# Synthesis of Prostate-Specific Membrane Antigen-Targeted Bimodal Conjugates of Cytotoxic Agents and Antiandrogens and Their Comparative Assessment with Monoconjugates

**DOI:** 10.3390/ijms241411327

**Published:** 2023-07-11

**Authors:** Nikolai Y. Zyk, Anastasiia S. Garanina, Ekaterina A. Plotnikova, Anton P. Ber, Ekaterina A. Nimenko, Natalia S. Dashkova, Anastasiia A. Uspenskaia, Radik R. Shafikov, Dmitry A. Skvortsov, Stanislav A. Petrov, Andrey A. Pankratov, Nikolai V. Zyk, Alexander G. Majouga, Elena K. Beloglazkina, Aleksei E. Machulkin

**Affiliations:** 1 Chemistry Department, Lomonosov Moscow State University, Leninskie Gory, Building 1/3, GSP-1, Moscow 119991, Russiaberantonmsu@gmail.com (A.P.B.); nata.dashkova1996@mail.ru (N.S.D.); uspenskaya.n@gmail.com (A.A.U.); iltarn@mail.ru (R.R.S.); skvorratd@mail.ru (D.A.S.); zyknikola@gmail.com (N.Y.Z.); 2 Laboratory of Biomedical Nanomaterials, National University of Science and Technology MISIS, 4 Leninskiy pr, Moscow 119049, Russia; anastasiacit@gmail.com; 3 National Medical Research Radiological Centre of the Ministry of Health of the Russian Federation, 2 Botkinskiy proezd, 3, Moscow 125284, Russia; plotnikovaekaterina62@gmail.com (E.A.P.);; 4 Shemyakin and Ovchinnikov Institute of Bioorganic Chemistry Russian Academy of Sciences, GSP-7, Ulitsa Miklukho-Maklaya, 16/10, Moscow 117997, Russia; 5 Dmitry Mendeleev University of Chemical Technology of Russia, Miusskaya sq. 9, Moscow 125047, Russia

**Keywords:** prostate cancer, prostate-specific membrane antigen, targeted drug delivery, bimodal conjugates, monomethyl auristatin E

## Abstract

Prostate cancer is the second most common cancer among men. We designed and synthesized new ligands targeting prostate-specific membrane antigen and suitable for bimodal conjugates with diagnostic and therapeutic agents. *In vitro* studies of the affinity of the synthesized compounds to the protein target have been carried out. Based on these ligands, a series of bimodal conjugates with a combination of different mitosis inhibitors and antiandrogenic drugs were synthesized. The cytotoxicity of the compounds obtained *in vitro* was investigated on three different cell lines. The efficacy of the two obtained conjugates was evaluated *in vivo* in xenograft models of prostate cancer. These compounds have been shown to be highly effective in inhibiting the growth of PSMA-expressing tumors.

## 1. Introduction

Prostate cancer (PCa) is currently the second most newly diagnosed cancer in men [1]. Current therapies for the disease have a number of serious side effects, such as impaired urinary and bowel function, possible erectile dysfunction, hair loss, allergic reactions, increased risk of infections, bleeding, diabetes, bone fractures and cardiovascular disease [2,3,4,5]. A potential solution to this problem is the development of drugs that deliver therapeutic agents directly to the tumor tissue. In the case of PCa, one of the most promising targets is prostate-specific membrane antigen (PSMA), which is hyperexpressed in tumor cells [6]. There are a number of therapeutic and diagnostic drugs targeting PSMA currently in various stages of clinical or pre-clinical trials [7,8].

Another promising approach in modern cancer therapy and diagnosis is the use of a combination of different agents. A number of examples of different combinations of therapeutic or diagnostic agents, are present in the literatures [9,10,11]. In the case of therapeutic drugs, the aim is to achieve a synergistic effect between agents of different nature, and in the case of diagnostic agents, the possibility of using different diagnostic methods with a single administration of a drug. In the case of PCa, of particular interest is the combination of mitosis-inhibiting drugs (e.g., Docetaxel, Monomethyl Auristatin E (MMAE) or others) and drugs with antiandrogenic activity (such as Abiraterone, Enzalutamide or others) [12,13,14]. It should be noted that a number of papers have presented platforms for co-delivery of various drug combinations for the treatment of PCa [15,16], but in most cases, the authors have used high-molecular-weight delivery platforms, which have certain disadvantages [11]. At the same time, a number of low-molecular-weight bimodal diagnostic and theranostic conjugates targeting PSMA have been reported in the literature [17,18,19]. The urea–vinyl derivatives DCL ((*S*)-2-(3-((*S*)-5-amino-1-carboxypentyl)ureido)pentanedioic acid) and DUPA ((2*S*, 2′*S*)-2,2′-(carbonylbis(azanediyl))dipentanedioic acid) are used as vector fragments in these studies.

The aim of this work was to synthesize and biologically investigate a series of low-molecular-weight PSMA ligands suitable for the development of bimodal conjugates with different actions. Such platforms have a number of advantages over high-molecular-weight delivery platforms: cheapness of production compared to antibodies, high reproducibility of synthesis compared to high-molecular-weight platforms, and no immune response. Also, these ligands may have a high affinity for PSMA, which could reduce the non-specific toxicity of their therapeutic products and achieve greater specificity of the conjugates to cancer cells. *In vitro* affinity studies of the resulting compounds have confirmed their ability to bind effectively to PSMA. In order to test the possibility of efficiently producing double conjugates, it was decided to synthesize a series of therapeutic conjugates with a combination of antiandrogenic agents and mitosis inhibitors based on the obtained PSMA ligands. The selection of this mix of drugs is based on a number of studies that have already tested such combinations [12,13,15]. According to the literature, Abiraterone and Enzalutamide, by inhibiting the androgen receptor (in case of Enzalutamide) or by inhibiting CYP17 enzyme and disrupting testosterone production (in case of Abiraterone), can induce increased levels of PSMA, which may further increase the specificity of the created drugs [20,21,22]. A series of biological studies on the resulting bimodal conjugates were subsequently carried out. *In vitro* studies determined cytotoxicity as well as qualitatively evaluated the non-specific toxicity of the compounds obtained on PSMA-positive and PSMA-negative cell lines. Further *in vivo* studies in xenograft models of PCa made it possible to more fully evaluate the efficacy of the obtained conjugates in comparison with some previously described drugs. By performing this experiment on PSMA-positive and PSMA-negative models, selectivity of these drugs was qualitatively evaluated.

## 2. Results and Discussion

**Figure 1:**
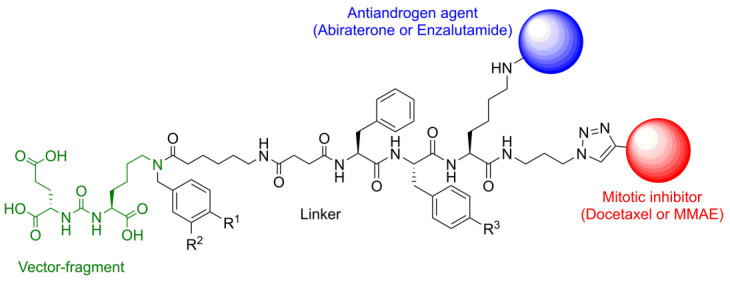
General structure of PSMA-targeted bimodal conjugates.

Analysis of literature data [11] on synthetic approaches for the design of low-molecular-weight bimodal theranostic and diagnostic conjugates showed that one of the most promising strategies is the separate synthesis of a modified vector molecule with an aliphatic linker fragment and the creation of a peptide linker fragment with a lysine residue using solid-phase peptide synthesis (SPPS) techniques. The next step involves obtaining the immobilized ligand on the resin, its subsequent removal from the solid carrier, modification with 3-azidopropylamine and removal of the protecting groups. Since, in contrast to the previously known ligands [23,24], a lysine residue was introduced into the structure of the peptide linker fragment as a second fragment for subsequent conjugation to one of the therapeutic agents, the affinity of the compounds obtained to PSMA had to be evaluated. The PSMA ligands thus obtained, suitable for bimodal conjugates, were subsequently reacted with modified therapeutic agents to produce target products with the general structural motif shown in Figure 1.

The following functional groups were selected as substituents in the benzyl fragment at the ε-amino group of lysine (Figure 1. R^1^ and R^2^): *meta*-Cl, *para*-Br and *para*-COOH. This choice is justified by previously published data about the affinity to PSMA of ligand analogues without a lysine fragment in the structure [23,24]. All amino acid residues in the peptide fragment of the linker are L-isomers. This is attributed to the better binding efficiency of ligands with this configuration of linker amino acid residues to a protein target as compared to analogous compounds with amino acid residues possessing D-configuration or mixed configuration [25]. Phenylalanine or tyrosine were chosen as one of the amino acid residues in the peptide sequence. This choice was justified by a number of studies that examined in detail the effect of the chosen amino acid residues on the affinity to PSMA [25,26,27]. Thus, in [25], a glycine residue was inserted into the linker structure instead of one of the two phenylalanine residues, and this resulted in a significant decrease in the affinity of the resulting ligands.

### 2.1. Synthesis of PSMA Ligands

**Scheme 1:**
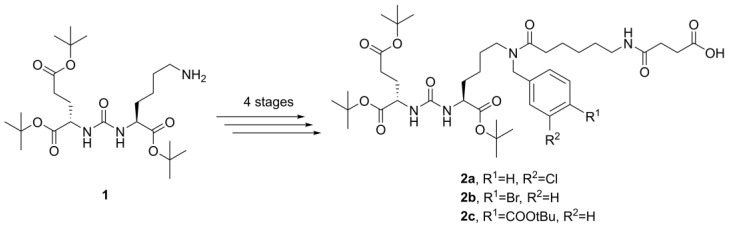
General scheme for obtaining compounds **2a**–**c**.

Synthesis of vector fragments with the aliphatic part of the linker was carried out according to the published protocols [23,24]. The tert-butyl protected urea derivative DCL (Scheme 1, compound **1**) was chosen as the starting compound because of its synthetic availability and its common use as a vector fragment of PSMA ligands and conjugates based on them [23,28,29,30]. Also, the ε-amino group of lysine can easily be modified to obtain more structurally complex PSMA ligands. A series of compounds **2a**–**c** was obtained by a four-step synthesis (Scheme 1).

**Scheme 2:**
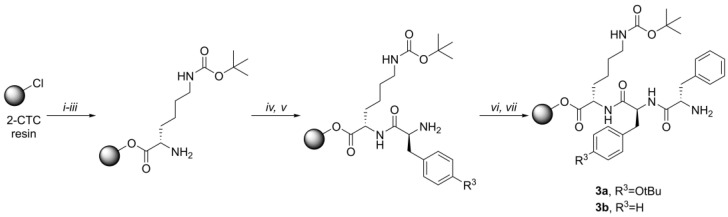
Scheme for the solid-phase synthesis of peptide fragments **3a**–**b**. Reagents and conditions: i, SOCl_2_, DMF, DCM, 40 °C; ii, Fmoc-Lys(Boc)-OH, HBTU, HOBt, DIPEA, DMF; iii, 20% solution of 4-methylpiperidine in DMF; iv, Fmoc-Phe-OH or Fmoc-Tyr(OtBu)-OH, HBTU, HOBt, DIPEA, DMF; v, 20% solution of 4-methylpiperidine in DMF; vi, Fmoc-Phe-OH, HBTU, HOBt, DIPEA, DMF; vii, 20% solution of 4-methylpiperidine in DMF.

To obtain the tripeptide fragment, a solid-phase synthesis method using 2-chlorotrityl chloride resin (2-CTC resin) was chosen. This approach was chosen because of the availability of established protocols for the synthesis of similar compounds [17,27]. Alternatively, liquid-phase synthesis can be used. However, the use of liquid-phase synthesis involves a greater number of stages with chromatographic separation, which is time-consuming. The general approach for creating such peptide fragments and their subsequent modification with PSMA ligands has been described in detail previously [17]. A general scheme for producing tripeptide fragments is shown in Scheme 2.

**Scheme 3:**
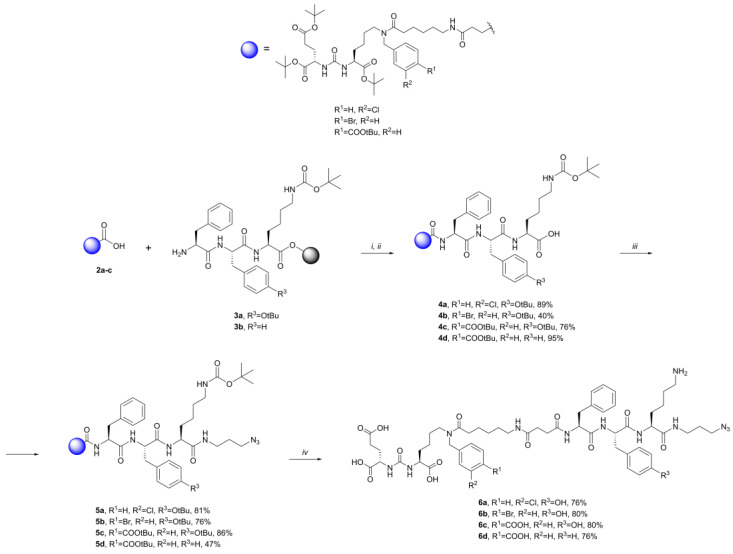
Scheme for producing PSMA ligands **6a**–**d**. Reagents and conditions: i, HBTU, HOBt, DIPEA, DMF; ii, 0.75% solution of TFA in DCM; iii, 3-azidopropylamine, HBTU, HOBt, DIPEA, DMF; iv, TFA/DCM/TIPS/H_2_O (50/40/5/5 *v*/*v*, %).

In the first step, the 2-CTC resin was activated with thionyl chloride in the presence of dimethylformamide (DMF). Dichloromethane (DCM) was used as solvent. In the next step, immobilization of lysine and subsequent removal of the Fmoc-protective group with a 20% solution of 4-methylpiperidine in DMF was carried out (Scheme 2, stages i–iii). The next step was to introduce a second amino acid residue in the presence of HBTU (2-(1H-benzotriazol-1-yl)-1,1,3,3-tetramethyluronium hexafluorophosphate), HOBt (hydroxybenzotriazole) and DIPEA (N,N-diisopropylethylamine) (Scheme 2, stage iv). This was followed by removal of the Fmoc-protective group, acylation of the dipeptide fragment with phenylalanine and removal of the protective group (Scheme 2, stages v–vii). Thus, tripeptides **3a** and **3b** immobilized on 2-CTC resin were obtained. Subsequently, these compounds were reacted by acylation with the previously obtained **2a**–**c** fragments in the presence of HBTU, HOBt and base. The compounds were then removed from the resin using a solution of trifluoroacetic acid (TFA) in DCM, and products **4a**–**d** were isolated (Scheme 3, stages i and ii). The products of this reaction were further introduced into the acylation reaction with 3-azidopropylamine in the presence of HBTU, HOBt and DIPEA. Thus, protected **5a**–**d** ligands with a terminal azido group in their structure were obtained. In the last step, the removal of the protecting groups was carried out by a TFA/triisopropyl silane (TIPS)/DCM/water mixture, and **6a**–**d** ligands were obtained using reverse phase preparative column chromatography. The purity of the obtained ligands was confirmed using HPLC-MS.

Thus, four new PSMA ligands with a lysine residue in their structures were obtained, which can be used as the basis for the synthesis of various therapeutic and diagnostic bimodal conjugates.

### 2.2. In Vitro Affinity Studies of Synthesized PSMA Ligands

**Figure 2:**
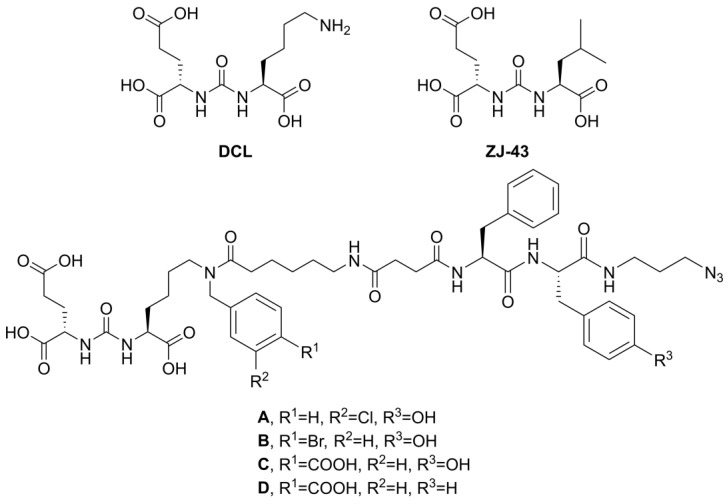
Structure of comparison ligands.

The affinity of the four newly obtained ligands to PSMA was evaluated *in vitro* by inhibiting the cleavage of N-acetylaspartylglutamic acid (NAAG). One function of PSMA, also known as glutamate carboxypeptidase II (GCPII), is the cleavage of NAAG into N-acetylaspartate and glutamate [31]. PSMA-specific ligands inhibit this reaction by interacting with the protein. Thus, the calculation of the values of half-maximum inhibition (IC_50_) allows evaluation of the binding efficiency of the synthesized compounds to the protein. The analogues of the obtained ligands **6a**–**d** without a lysine residue in their structure were chosen as comparison ligands. Also, some ligands previously described in the literature were chosen as references. The structures of the reference compounds are shown in Figure 2. The IC_50_ values obtained are shown in Table 1.

As can be seen from the IC_50_ values given in Table 1, the addition of a lysine residue into the structure does not result in a significant change in affinity. All the obtained ligands were found to be significantly superior to the parent DCL urea. The synthesized compounds **6a**–**d** have IC_50_ values comparable with those of the reference ligand ZJ-43. On the basis of these data, it is difficult to identify a single leader compound. Therefore, we decided to obtain a series of bimodal therapeutic conjugates with each of the ligands.

### 2.3. Synthesis of Bimodal Therapeutic Conjugates

To obtain the target bimodal conjugates, the selected therapeutic agents were further modified using both previously known technique [32,33] and the methods first proposed in this work. A detailed synthesis of the modified therapeutic agents is presented in the data sheet (Figures S1 and S2).

#### 2.3.1. Synthesis of Conjugates with the Drug Combination Docetaxel/Abiraterone

**Scheme 4:**
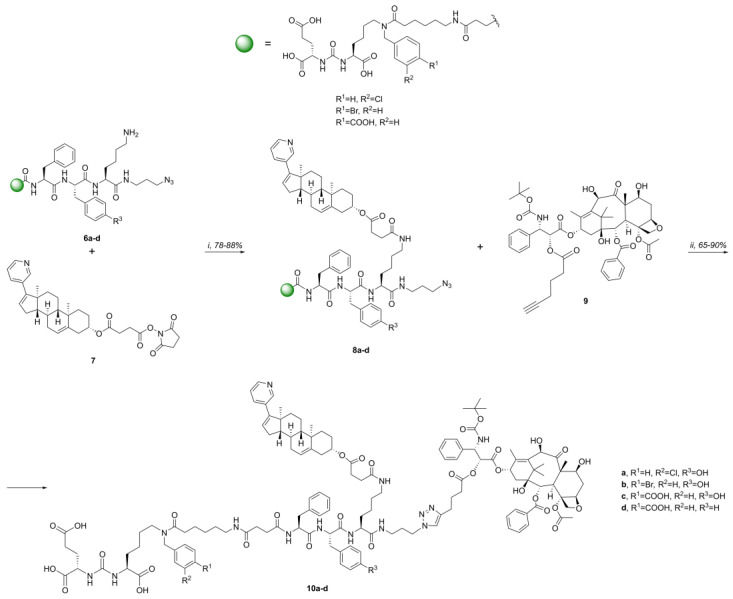
Scheme for the synthesis of bimodal conjugates **10a**–**d**. Reagents and conditions: i, DIPEA, DMF; ii, CuSO_4_*5H_2_O, sodium ascorbate, DMF/H_2_O (3/1).

To obtain bimodal conjugates with the Docetaxel/Abiraterone drug pair, ligands **6a**–**d** were reacted with modified drug **7** in the presence of a non-nucleophilic base (Scheme 4, stage i). Compounds **8a**–**d** were synthesized in this manner and further reacted with modified Docetaxel **9** in a copper-catalyzed azide–alkyne cycloaddition reaction (Scheme 4, stage ii). The obtained bimodal conjugates **10a**–**d** were isolated individually.

#### 2.3.2. Synthesis of Conjugates with MMAE/Abiraterone Drug Combination

**Scheme 5:**
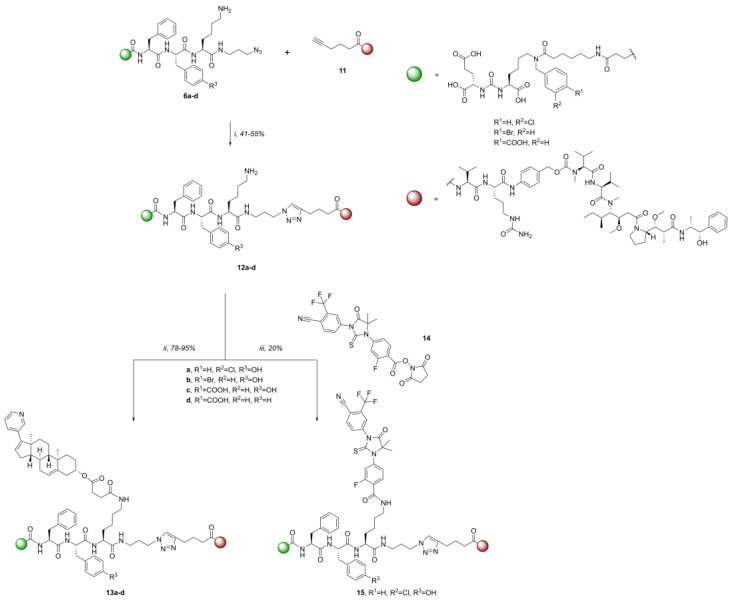
Scheme for the synthesis of bimodal conjugates **13a**–**d** and **15**. Reagents and conditions: i, CuSO_4_*5H_2_O, sodium ascorbate, DMF/H_2_O (3/1); ii, **7**, DIPEA, DMF; iii, **14**, DIPEA, DMF.

A model reaction was performed between Abiraterone **8a** monoconjugate and modified Monomethyl Auristatin E **11** (Figure S3) to obtain a series of conjugates with the drug pair MMAE and Abiraterone. However, it was not possible to isolate the target product individually (detailed data on this synthetic route are given in the electronic supplementary materials). The azide–alkyne cycloaddition reaction produced a stable impurity that could not be separated using reverse phase column chromatography (Figure S4). In this regard, the synthetic scheme was modified. In the first step, monomodal conjugates **12a**–**d** were obtained by reaction between modified MMAE (**11**) and ligands **6a**–**d** (Scheme 5, stage i). The acylation reaction of the monoconjugates was then carried out with compound **7** (Scheme 5, stage ii). Four bimodal **13a**–**d** conjugates were obtained this way.

#### 2.3.3. Synthesis of Conjugate with MMAE/Enzalutamide Drug Combination

Based on the presented scheme for the synthesis of compounds **13a**–**d**, the reaction between monoconjugate **12a** and modified Enzalutamide **14** was carried out (Scheme 5, stage iii). In contrast to the bimodal conjugates **13a**–**d**, compound **15** could not be isolated individually via precipitation with acetonitrile. Therefore, the product was purified from impurities using reverse phase column chromatography. Due to a more difficult method of purification the target conjugate **15** was obtained in moderate yield.

Nine new bimodal therapeutic conjugates with different structures were obtained. Of these, four compounds carried the therapeutic load of the drug pair Docetaxel/Abiraterone, differing from each other by the ligand structure (**10a**–**d**). Another four compounds (**13a**–**d**) have the MMAE/Abiraterone drug pair in their structure and also differ in the structure of the starting ligands. In addition, one bimodal conjugate was obtained with the MMAE/Enzalutamide drug pair (**15**) based on ligand **6a**.

### 2.4. In Vitro Cytotoxicity Studies of Bimodal Conjugates

The resulting bimodal conjugates were tested *in vitro* on three different PCa cell lines. We used LNCaP cells with high PSMA expression, 22Rv1 cells with moderate PSMA expression and PC-3 cells with extremely low PSMA expression. Different levels of PSMA expression in 22Rv1 and LNCaP cell lines allows evaluation of the effect of the level of expression on the toxicity of the target drugs. PSMA-negative cell line PC-3 allows a qualitative assessment of non-specific toxicity of the obtained compounds.

In order to be able to compare the cytotoxicity of the compounds in a biological study, various therapeutic agents were chosen as references.

#### 2.4.1. In Vitro Studies of Conjugates Containing Docetaxel

**Figure 3:**
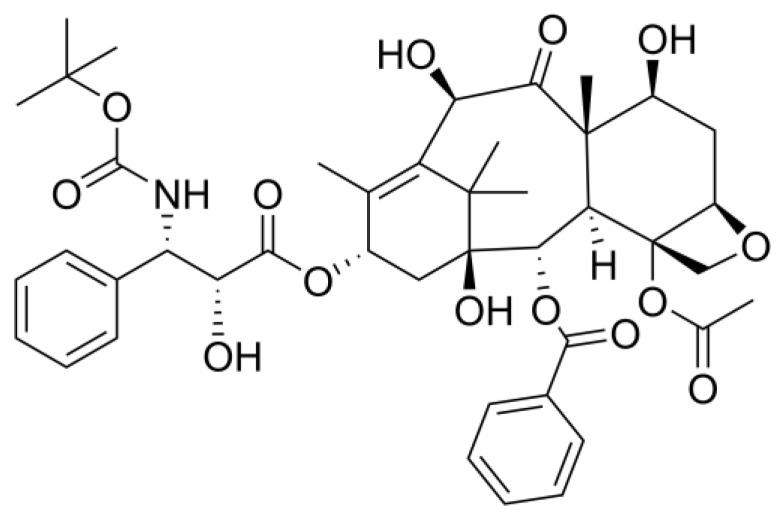
Structure of Docetaxel.

Docetaxel (**DOC**) was chosen as the reference compound in an *in vitro* experiment with **10a**–**d** conjugates (Figure 3).

**Figure 4:**
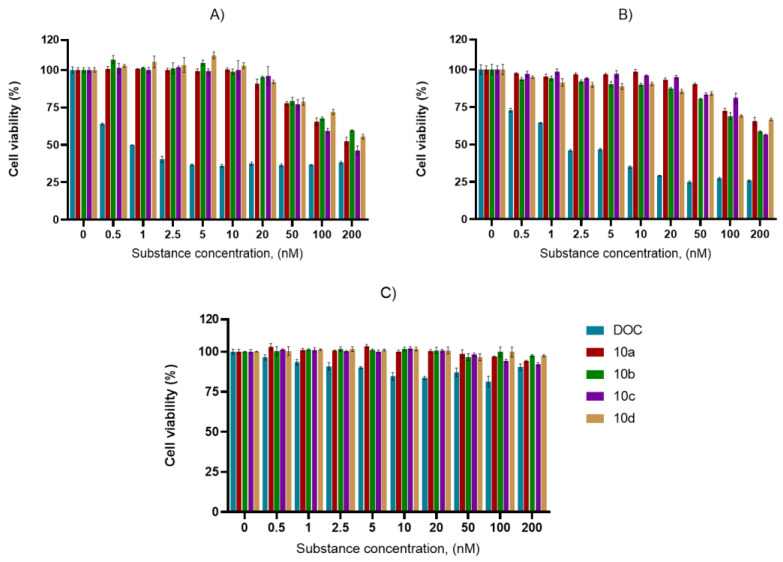
Graphical representation of the results of the *in vitro* experiment on prostate cancer cell lines: (**A**) LNCaP; (**B**) 22Rv1; (**C**) PC-3.

The obtained cytotoxicity data are shown in Figure 4.

As can be seen from the presented data for all three cell lines, the obtained compounds are inferior in toxicity to Docetaxel. However, in the case of the PSMA-negative PC-3 cell line (Figure 4C), this can be explained by the low non-specific toxicity of the synthesized conjugates. On the basis of cytotoxicity data on PSMA-expressing cell lines LNCaP and 22Rv1 (Figure 4A,B), it may be argued that all obtained compounds have very similar cytotoxicity profiles, and in spite of a slightly higher toxicity of compound **10c**, it is impossible to determine any leading compound. It is also noteworthy that the synthesized compounds are more cytotoxic for the LNCaP cell line, which has a higher level of PSMA expression than for the 22Rv1 cell line, which has a lower level of PSMA expression. This fact, combined with the low toxicity on PSMA-negative PC-3 cell lines, suggests that the cytotoxicity of the obtained **10a**–**d** conjugates is dependent on the level of PSMA expression in PCa cells.

Based on the rather low toxicity of the resulting series of compounds, another therapeutic agent, Monomethyl Auristatin E, which is also a mitosis inhibitor, was introduced into the structure instead of Docetaxel.

#### 2.4.2. In Vitro Studies of Conjugates Containing Monomethyl Auristatin E

**Figure 5:**
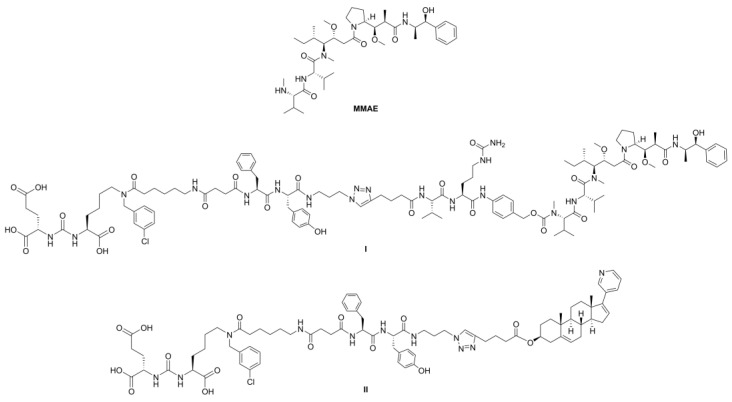
Structures of the reference compounds; **I**—monomodal conjugate with MMAE; **II**—monomodal conjugate with abiraterone.

*In vitro* studies of compounds with MMAE have evaluated the cytotoxicity of both bimodal **13a**–**d** and **15** conjugates and **12a**–**d** precursor compounds. The chosen reference compounds were the previously described monoconjugates with MMAE and Abiraterone (**I** and **II**) [33,34], their equimolar mixture (**Combo**) and the MMAE. The structures of the reference compounds are shown in Figure 5. The resulting CC_50_ values from the experiment are shown in Table 2.

The presented data show that the **13a**–**d** bimodal conjugates and their **12a**–**d** precursor compounds have similar cytotoxicity on LNCaP cell line with high expression of PSMA. However, on the 22Rv1 cell line, the bimodal conjugates with the therapeutic agent pair Abiraterone/MMAE show greater toxicity than the **12a**–**d** compounds. InPSMA-negative line PC-3, these 8 compounds show a much lower toxicity, indicating the selectivity of the obtained compounds. Compared with the previously described conjugate with MMAE **I**, this series of compounds shows slightly lower cytotoxicity on all cell lines. The CC_50_ values of all synthesized compounds are significantly lower than those of the PSMA ligand monoconjugate with Abiraterone **II**. An equimolar mixture of compounds **I** and **II** (**Combo**) shows greater cytotoxicity compared to the **13a**–**d** conjugates. On the PC-3 cell line, the CC_50_ value was not achieved for the synthesized compounds in the investigated concentration range (0.5 to 800 nM). However, in this concentration range, the conjugates **13a**–**d** showed significantly lower toxicity compared to the monoconjugate combination (electronic supplementary materials, Figure S5). This fact may indicate a greater non-specific toxicity of the monoconjugate combination compared to the synthesized bimodal conjugates.

The conjugate **15** with the Enzalutamide/MMAE drug combination showed slightly higher cytotoxicity than the series of compounds with the Abiraterone/MMAE pair. At the same time, this compound is inferior to conjugate **I**.

Monomethyl Auristatin E is superior in cytotoxicity to all presented conjugates. This fact is explained by the extremely high toxicity of this drug. For this reason, the compound is not used in its free form in therapy. In most cases, MMAE is used in the form of various antibody conjugates [14,35].

### 2.5. In Vivo Studies of Therapeutic Conjugates

We carried out *in vivo* studies of the efficacy of the obtained bimodal conjugates. Conjugates **13a** and **15** were chosen for this experiment due to the fact that these compounds share a common structural motif and Monomethyl Auristatin E as a reference compound. Also, conjugate **I** (Figure 5) was used as a reference compound. An isotonic (0.9%) sodium chloride solution was used as a negative control. Studies were carried out in xenograft models of 22Rv1 human prostate carcinoma and PC-3 prostate adenocarcinoma in immunodeficient Balb/c nude mice (male).

The results of the histological studies carried out on these xenografts have previously been presented in [33]. A detailed description of this experiment is given in the electronic supplementary materials (Figures S6 and S7).

Three injections of 132.3 nM/kg (dosage selection is justified by chronic toxicity studies for conjugate **I** [33]) had no effect on the general condition and weight of the animals in all groups and no mortality was observed in mice. Animal body weight data are presented in Table S2 in the electronic supplementary materials.

**Figure 6:**
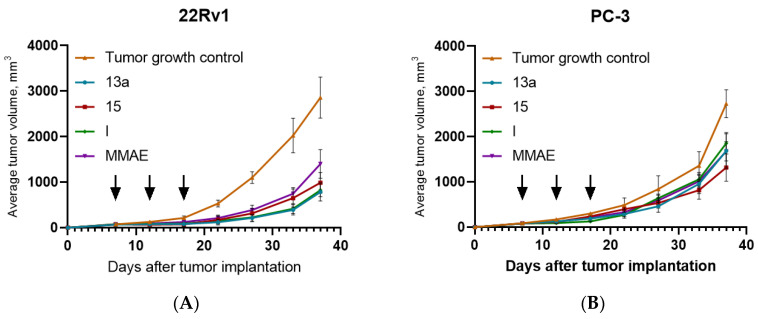
Tumor growth plots of 22Rv1 (**A**) and PC-3 (**B**) in mice with intravenous triple injection of **13a**, **15** and MMAE (single dose of 132.3 nM/kg). Arrows indicate days of injection.

**Figure 7:**
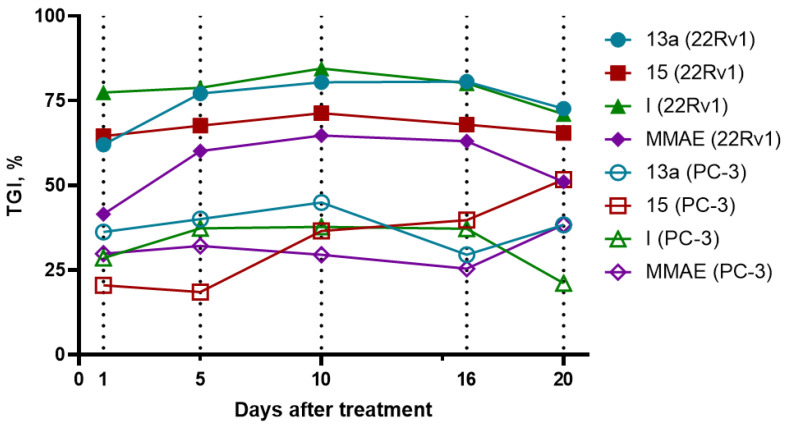
Dynamics of tumor growth inhibition ratio (TGI) in the 22Rv1 and PC-3 xenograft models.

The antitumor effect of treatment of 22Rv1-based xenograft was high, and the mean tumor volume increased slowly compared to the mean tumor volume of the control group (Figure 6A). The inhibition of tumor growth with conjugate **13a** treatment was 60.6–80.6%, conjugate **15** was 53.9–71.3%, conjugate **I** was 59.1–84.5% and MMAE was 43.0–64.7% (Figure 7). The antitumor effect was significantly weaker in mice with PC-3-derived tumor (Figure 6B), and the TGI of the **13a** conjugate treatment did not exceed 44.9%, **15**—51.7%, **I**—37.7% and MMAE—38.4% (Figure 7) (Numerical data are given in the electronic supplementary materials in Tables S3 and S4).

Although compound **15** showed slightly higher cytotoxicity in the *in vitro* model, in the *in vivo* model, this compound is slightly inferior to the bimodal conjugate with the Abiraterone/MMAE drug pair. Meanwhile, conjugate **13a** in xenograft models shows a similar, although slightly inferior, performance to that of monoconjugate **I** described in the literature [33].

Thus, a comparative study of the antitumor efficacy of the **13a**, **15** conjugates in mice with 22Rv1 and PC-3 prostate xenografts revealed a treatment advantage in mice with 22Rv1 tumors, possibly due to a higher level of PSMA expression. Both bimodal conjugates are slightly inferior to monoconjugate **I** in efficacy. The lowest efficacy was shown with the free MMAE. It should be noted that the addition of the PSMA ligand to the molecule contributes to the efficacy of antitumor therapy.

## 3. Materials and Methods

### 3.1. General

More detailed experimental data can be found in the electronic supplementary material.

All used solvents were purified according to procedures described in [36]. All starting compounds were commercially available reagents or were synthesized according to previously published papers [23,24].

### 3.2. Synthesis

General procedure for synthesis of ligands **6a**–**d**

The protected ligand (**5a**–**d**) was dissolved in a mixture of trifluoroacetic acid (50% *v*/*v*), dichloromethane (40% *v*/*v*), triisopropylsilane (5% *v*/*v*) and water (5% *v*/*v*) in a ratio of 5 mL mixture per 100 mg ligand. The reaction mixture was stirred for 4 h. Then, the solvent was removed under reduced pressure, after which the dry residue was precipitated with diethyl ether, decanted and the precipitate was washed three times with Et_2_O. The product was isolated individually using reverse phase column chromatography, and a mixture of 0.1% *v*/*v* trifluoroacetic acid in water and acetonitrile was used as eluent.

Synthesis of compound **6a**

From 795 mg (0.529 mmol) of compound **5a** and 15 mL mixture of trifluoroacetic acid, triisopropylsilane, water and dichloromethane, 517 mg (76% yield) of compound **6a** was isolated individually using reverse phase column chromatography (InterchimPuriflashC18 120 g, 15µ, gradient from 10% acetonitrile to 100% acetonitrile in 50 min, flow rate 40 mL/min) as a white amorphous powder.

^1^H NMR (400 MHz, DMSO-d6) δ, ppm: 8.30–8.36 (m, 1H, NH), 8.12 (d, J = 7.52 Hz, 1H, NH), 7.91–8.00 (m, 1H, NH), 7.74 (m, 1H, NH), 7.49–7.57 (m, 1H, NH), 7.24–7.39 (m, 2H, Ar), 7.07–7.24 (m, 7H, Ar), 7.03 (m, 2H, Ar), 6.65 (d, J = 8.38 Hz, 2H, Ar), 6.28–6.38 (m, 2H, NH), 4.38–4.59 (m, 2H, CH_2_), 4.22–4.35 (m, 2H, CH), 3.96–4.13 (m, 3H, CH), 3.25–3.33 (m, 2H, CH_2_), 3.11–3.22 (m, 2H, CH_2_), 3.07 (m, 2H, CH_2_), 2.86–3.04 (m, 4H, CH_2_), 2.76–2.86 (m, 1H, CH_2_), 2.59–2.76 (m, 3H, CH_2_), 2.11–2.39 (m, 8H, CH_2_), 1.84–1.95 (m, 1H, CH_2_), 1.55–1.75 (m, 5H, CH_2_), 1.32–1.55 (m, 10H, CH_2_), 1.07–1.32 (m, 7H, CH_2_).

^13^C NMR (101 MHz, DMSO-d6) δ, ppm: 174.87, 174.58, 174.15, 173.07, 172.56, 171.95, 171.62, 171.46, 157.70, 156.28, 141.58, 138.89, 138.32, 133.44, 130.98, 130.62, 130.42, 129.40, 128.47, 128.22, 127.57, 127.24, 126.67, 126.45, 125.34, 115.38, 58.52, 55.38, 52.99, 52.55, 52.06, 48.63, 47.29, 45.72, 39.07, 37.41, 37.28, 36.25, 32.16, 31.57, 31.16, 30.98, 30.31, 29.47, 29.37, 28.64, 27.95, 27.01, 26.67, 25.10, 22.68.

[α]^20^_D_ = −11.0°

HPLC-MS: target compound content—99.9%, t_R_ = 11.05 min.

ESI-HRMS: for C_56_H_77_ClN_12_O_14_: *m*/*z* calculated for [M+H]^+^ 1177.5371, found: 1177.5443; *m*/*z* calculated for [M+Na]^+^ 1199.5269, found: 1199.5263.

Synthesis of compound **6b**

From 199 mg (0.129 mmol) of compound **5b** and 7 mL of a mixture of trifluoroacetic acid, triisopropylsilane, water and dichloromethane, 126 mg (80% yield) of compound **6b** was isolated individually using reverse phase column chromatography (InterchimPuriflash C18 40 g, 15µ, gradient from 10% acetonitrile to 20% acetonitrile in 2 min, from 20% acetonitrile to 100% in 35 min, flow rate 20 mL/min) as a white amorphous powder.

^1^H NMR (400 MHz, DMSO-d6) δ, ppm: 12.45 (br. s, 3H, COOH), 9.22 (br. s, 1H, OH), 8.29 (d, J = 7.34 Hz, 1H, NH), 8.10 (d, J = 8.13 Hz, 1H, NH), 7.88–7.96 (m, 1H, NH), 7.75–7.82 (m, 1H, NH), 7.65 (br, s, 3H, NH^3+^), 7.55–7.59 (m, 1H, NH), 7.54 (d, J = 8.38 Hz, 1H, Ar), 7.48 (d, J = 8.31 Hz, 1H, Ar), 7.19–7.25 (m, 2H, Ar), 7.09–7.19 (m, 5H, Ar), 7.03 (m, 2H, Ar), 6.65 (d, J = 8.38 Hz, 2H, Ar), 6.26–6.37 (m, 2H, NH), 4.49–4.43 (m, 2H, CH_2_), 4.28–4.38 (m, 2H, CH), 4.08 (m, 3H, CH), 3.27–3.32 (m, 2H, CH_2_), 3.12–3.21 (m, 2H, CH_2_), 3.05–3.12 (m, 2H, CH_2_), 2.88–3.05 (m, 4H, CH_2_), 2.77–2.85 (m, 1H, CH_2_), 2.73 (m, 2H, CH_2_), 2.61–2.69 (m, 1H, CH_2_), 2.26–2.35 (m, 3H, CH_2_), 2.14–2.26 (m, 5H, CH_2_), 1.85–1.96 (m, 1H, CH_2_), 1.65–1.75 (m, 2H, CH_2_), 1.57–1.65 (m, 3H, CH_2_), 1.45–1.55 (m, 6H, CH_2_), 1.34–1.44 (m, 3H, CH_2_), 1.31 (m, 1H, CH_2_), 1.12–1.29 (m, 6H, CH_2_).

^13^C NMR (101 MHz, DMSO-d6) δ, ppm: 174.55, 174.26, 173.82, 172.71, 172.07, 171.77, 171.59, 171.24, 171.08, 157.32, 155.91, 138.02, 137.94, 131.55, 131.24, 130.03, 129.72, 129.02, 128.64, 128.09, 127.84, 126.29, 120.14, 119.92, 114.99, 55.00, 52.61, 52.17, 51.73, 51.24, 48.23, 46.74 38.66, 36.87, 35.85, 31.90, 31.17, 30.77, 30.58, 29.97, 29.07, 28.26, 27.59, 26.65, 26.29, 24.72, 24.59, 22.31.

[α]^20^_D_ = −9.0°

HPLC-MS: target compound content—99.9%, t_R_ = 10.8 min.

ESI-HRMS: for C_56_H_77_BrN_12_O_14_: *m*/*z* calculated for [M+H]^+^ 1221.49383, found: 1221.4937.

Synthesis of compound **6c**

From 282 mg (0.180 mmol) of compound **5c** and 11 mL mixture of trifluoroacetic acid, triisopropylsilane, water and dichloromethane, 126 mg (80% yield) of compound **6c** was isolated individually using reverse phase column chromatography (InterchimPuriflash C18 120 g, 15µ, gradient from 10% acetonitrile to 100% acetonitrile in 50 min, flow rate 40 mL/min) as a white amorphous powder.

^1^H NMR (400 MHz, DMSO-d6) δ, ppm: 12.56 (br. s, 3H, COOH), 9.22 (br. s, 1H, OH), 8.29 (d, J = 7.40 Hz, 1H, NH), 8.10 (d, J = 7.70 Hz, 1H, NH), 7.89–7.97 (m, 2H, NH+Ar), 7.87 (d, J = 8.25 Hz, 1H, Ar), 7.74–7.82 (m, 1H, NH), 7.65 (m, 3H, NH), 7.53–7.60 (m, 1H, NH), 7.25–7.32 (m, 2H, Ar), 7.18–7.25 (m, 2H, Ar), 7.12–7.18 (m, 3H, Ar), 7.03 (m, 2H, Ar), 6.60–6.69 (m, 2H, Ar), 6.25–6.38 (m, 2H, NH), 4.60–4.53 (m, 2H, CH_2_), 4.26–4.38 (m, 2H, CH), 3.98–4.16 (m, 3H, CH), 3.32 (t, J = 6.85 Hz, 2H, CH_2_), 3.17 (m, 2H, CH_2_), 3.05–3.13 (m, 2H, CH_2_), 2.88–3.05 (m, 4H, CH_2_), 2.69–2.86 (m, 3H, CH_2_), 2.60–2.69 (m, 1H, CH_2_), 2.30–2.39 (m, 2H, CH_2_), 2.24–2.30 (m, 2H, CH_2_), 2.20 (m, 4H, CH_2_), 1.84–1.96 (m, 1H, CH_2_), 1.56–1.74 (m, 5H, CH_2_), 1.37–1.55 (m, 9H, CH_2_), 1.32 (m, 1H, CH_2_), 1.14–1.29 (m, 6H, CH_2_).

^13^C NMR (101 MHz, DMSO-d6) δ, ppm: 174.50, 174.21, 173.78, 172.62, 171.73, 171.55, 171.23, 171.07, 167.21, 157.30, 155.89, 137.94, 130.04, 129.78, 129.47, 129.02, 128.09, 127.83, 127.38, 126.29, 114.98, 54.97, 52.56, 52.13, 51.67, 48.23, 38.68, 36.88, 35.85, 31.78, 29.91, 28.26, 27.52, 26.62, 24.71, 22.27.

[α]^20^_D_ = −9.7°

HPLC-MS: target compound content—99.0%, t_R_ = 10.25 min.

ESI-HRMS: for C_56_H_78_N_12_O_16_: *m*/*z* calculated for [M+H]^+^ 1187.57315, found: 1187.572.

Synthesis of compound **6d**

From 119 mg (79.6 µmol) of compound **5d** and 6 mL of a mixture of trifluoroacetic acid, triisopropylsilane, water and dichloromethane, 71 mg (76% yield) of compound **6d** was isolated individually using reverse phase column chromatography (InterchimPuriflash C18 20 g, 15µ, gradient from 10% acetonitrile to 20% acetonitrile in 2 min, from 20% acetonitrile to 100% in 23 min, flow rate 20 mL/min) as a white amorphous powder.

^1^H NMR (400 MHz, DMSO-d6) δ, ppm: 12.55 (br. s, 3H, COOH), 8.30 (d, J = 7.28 Hz, 1H, NH), 8.17 (d, J = 7.89 Hz, 1H, NH), 7.89–7.99 (m, 2H, NH+Ar), 7.87 (d, J = 8.19 Hz, 1H, Ar), 7.77–7.84 (m, 1H, NH), 7.56–7.68 (m, 3H, NH+Ar), 7.22–7.30 (m, 6H, Ar), 7.20 (m, 2H, Ar), 7.15 (m, 3H, Ar), 6.26–6.36 (m, 2H, NH), 4.60–4.53 (m, 2H, CH_2_), 4.39–4.48 (m, 1H, CH), 4.31 (m, 1H, CH), 3.98–4.17 (m, 3H, CH), 3.32 (t, J = 6.76 Hz, 2H, CH_2_), 3.12–3.25 (m, 2H, CH_2_), 3.07–3.12 (m, 2H, CH_2_), 2.97–3.07 (m, 3H, CH_2_), 2.87–2.97 (m, 3H, CH_2_), 2.73 (m, 2H, CH_2_), 2.59–2.69 (m, 1H, CH_2_), 2.30–2.38 (m, 2H, CH_2_), 2.13–2.30 (m, 6H, CH_2_), 1.85–1.96 (m, 1H, CH_2_), 1.64 (m, 5H, CH_2_), 1.50 (m, 6H, CH_2_), 1.41 (m, 3H, CH_2_), 1.12–1.34 (m, 7H, CH_2_).

[α]^20^_D_ = −7.9°

HPLC-MS: target compound content—99.9%, t_R_ = 10.7 min.

ESI-HRMS: for C56H78N12O15: *m*/*z* calculated for [M+H]^+^ 1171.57824, found: 1171.5782.

General procedure for the preparation of monoconjugates **8a**–**d**

1 eq. of compound **6a**–**d** was dissolved in DMF. Compound **7** (1.2 eq.) and DIPEA (6 eq.) were added to the solution. The reaction mixture was stirred until the reaction was completed (the reaction was monitored using TLC in a system of 10% methanol in dichloromethane + 1% trifluoroacetic acid). The solvent was removed under reduced pressure. The dry residue was precipitated with acetonitrile, then decanted and the precipitate was washed three times with acetonitrile.

Synthesis of compound **8a**

From 100 mg (77.4 µmol) of compound **6a**, 51 mg (92.9 µmol) of compound **7** in the presence of 81 µL (0.464 mmol) of DIPEA in 10 mL of DMF, 131 mg (85% yield) of compound **8a** was obtained as white powder.

^1^H NMR (400 MHz, DMSO-d6) δ, ppm: 8.53 (s, 1H, Py), 8.39 (d, J = 4.59 Hz, 1H, Py), 8.15 (d, J = 6.79 Hz, 1H, NH), 8.01 (m, 1H, NH), 7.84–7.92 (m, 1H, NH), 7.74 (d, J = 7.82 Hz, 1H, Py), 7.55–7.64 (m, 1H, NH), 7.47 (m, 1H, NH), 7.23–7.40 (m, 3H, Ar+Py), 7.08–7.23 (m, 7H, Ar), 6.98–7.08 (m, 2H, Ar), 6.65 (d, J = 8.01 Hz, 2H, Ar), 6.28–6.38 (m, 2H, NH), 6.08 (br. s, 1H, CH), 5.27–5.38 (m, 1H, CH), 4.36–4.52 (m, 3H, CH_2_+CH), 4.24 (m, 2H, CH), 3.95–4.12 (m, 3H, CH), 3.28 (t, J = 6.72 Hz, 2H, CH_2_), 3.14 (m, 2H, CH_2_), 2.99–3.10 (m, 3H, CH_2_), 2.97 (m, 3H, CH_2_), 2.74–2.93 (m, 4H, CH_2_), 2.58–2.69 (m, 1H, CH_2_), 2.42 (m, 2H, CH_2_), 2.08–2.38 (m, 13H, CH_2_), 1.92–2.01 (m, 3H, CH_2_), 1.89 (m, 1H, CH_2_), 1.78 (m, 2H, CH_2_), 1.54–1.73 (m, 9H, CH_2_), 1.26–1.53 (m, 14H, CH_2_), 1.08–1.26 (m, 7H, CH_2_+CH_3_), 0.84–1.08 (m, 9H, CH_2_+CH_3_).

HPLC-MS: target compound content—97%, t_R_ = 8.26 min.

Synthesis of compound **8b**

From 81 mg (60.6 µmol) of compound **6b**, 40 mg (72.7 µmol) of compound **7** in the presence of 63 µL (0.364 mmol) of DIPEA in 9 mL of DMF, 80 mg (80% yield) of compound **8b** was obtained as white powder.

^1^H NMR (400 MHz, DMSO-d6) δ, ppm: 8.54 (s, 1H, Py), 8.39 (d, J = 4.59 Hz, 1H, Py), 8.28–8.34 (m, 1H, NH), 8.15 (m, 1H, NH), 7.97–8.05 (m, 1H, NH), 7.92–7.97 (m, 1H, NH), 7.82–7.92 (m, 1H, NH), 7.74 (d, J = 8.07 Hz, 1H, Py), 7.61 (t, J = 8.34 Hz, 1H, NH), 7.41–7.55 (m, 3H, Ar+NH), 7.33 (m, 1H, Py), 6.98–7.24 (m, 9H, Ar), 6.65 (d, J = 7.58 Hz, 2H, Ar), 6.26–6.36 (m, 2H, NH), 6.08 (br. s, 1H, CH), 5.32 (m, 1H, CH), 4.33–4.53 (m, 3H, CH_2_+CH), 4.25 (m, 2H, CH), 3.94–4.10 (m, 3H, CH), 3.28 (t, J = 6.82 Hz, 2H, CH_2_), 2.74–3.22 (m, 13H, CH_2_), 2.58–2.70 (m, 1H, CH_2_), 2.38–2.45 (m, 2H, CH_2_), 2.08–2.38 (m, 14H, CH_2_), 1.83–2.08 (m, 5H, CH_2_), 1.26–1.82 (m, 26H, CH_2_), 1.08–1.26 (m, 7H, CH_2_+CH_3_), 0.84–1.07 (m, 9H, CH_2_+CH_3_).

HPLC-MS: target compound content—97%, t_R_ = 8.40 min.

Synthesis of compound **8c**

From 32 mg (24.6 µmol) of compound **6c**, 16 mg (29.5 µmol) of compound **7** in the presence of 30 µL (0.172 mmol) of DIPEA in 5 mL of DMF, 31 mg (78% yield) of compound **8c** was obtained as white powder.

HPLC-MS: target compound content—99.9%, t_R_ = 4.60 min.

ESI-HRMS: for C_85_H_111_N_13_O_19_: *m*/*z* calculated for [M+H]^+^ 1618.8119, found: 1618.82.

Synthesis of compound **8d**

From 48 mg (37.3 µmol) of compound **6d**, 24 mg (44.8 µmol) of compound **7** in the presence of 45 µL (0.261 mmol) of DIPEA in 7 mL of DMF, 53 mg (88% yield) of compound **8d** was obtained as white powder.

^1^H NMR (400 MHz, DMSO-d6) δ, ppm: 8.54 (s, 1H, Py), 8.39 (m, 1H, Py), 8.22 (m, 1H, NH), 8.05 (m, 1H, NH), 7.81–7.93 (m, 2H, NH), 7.74 (m, 1H, Py), 7.62 (m, 1H, NH), 7.53 (m, 1H, NH), 7.33 (m, 1H, Py), 7.21–7.29 (m, 6H, Ar), 7.06–7.21 (m, 6H, Ar), 6.31 (m, 1H, NH), 6.08 (m, 1H, CH), 5.32 (m, 1H, CH), 4.50–4.57 (m, 2H, CH_2_), 4.38 (m, 2H, CH), 4.23 (m, 1H, CH), 4.06 (m, 2H, CH), 4.01 (m, 1H, CH), 3.29 (m, 2H, CH_2_), 2.78–3.13 (m, 13H, CH_2_), 2.57–2.68 (m, 1H, CH_2_), 2.43 (m, 2H, CH_2_), 2.15–2.30 (m, 13H, CH_2_), 1.88–1.99 (m, 4H, CH_2_), 1.77 (m, 2H, CH_2_), 1.53–1.69 (m, 9H, CH_2_), 1.30–1.50 (m, 14H, CH_2_), 1.19 (m, 6H, CH_2_+CH_3_), 0.97 (m, 9H, CH_2_+CH_3_).

HPLC-MS: target compound content—99.9%, t_R_ = 6.89 min.

General procedure for the preparation of bimodal **10a**–**d** conjugates using an azide–alkyne cycloaddition reaction.

Monoconjugate **8a**–**d** (1 eq.) and Docetaxel-alkyne **9** (1.2 eq.) were dissolved in a mixture of DMF and water. The flask was filled with argon, then aqueous solutions of sodium ascorbate (1.2 eq.) and copper sulfate pentahydrate (0.4 eq.) were added to the system. The reaction mixture was stirred for 18 h. Afterwards, EDTA (0.8 eq.) was added and stirred for another three hours with access to oxygen in air. The solvent was removed under reduced pressure, the dry residue was precipitated with acetonitrile and decanted. Then, the product was isolated individually using reverse phase column chromatography using acetonitrile–water mixture as eluent.

Synthesis of compound **10a**

From 60 mg (37.3 µmol) of compound **8a**, 40 mg (44.8 µmol) of Docetaxel-alkyne **9**, 9 mg (44.8 µmol) of sodium ascorbate, 4 mg (14.9 µmol) of copper sulfate pentahydrate in 8 mL DMF/water mixture (3/1), followed by addition of 9 mg (29.8 µmol) EDTA was isolated individually using reverse phase column chromatography (InterchimPuriflashC18 20 g, 15µ, gradient from 15% acetonitrile to 100% acetonitrile in 20 min, flow rate 20 mL/min) 85 mg (90% yield) of compound **10a** as white powder.

^1^H NMR (400 MHz, DMSO-d6) δ, ppm: 8.76 (s, 1H, Py), 8.62 (d, J = 5.32 Hz, 1H, Py), 8.33 (d, J = 8.3 Hz, 1H, Py), 7.94 (d, J = 7.40 Hz, 2H, Ar), 7.82 (m, 1H, Py), 7.78 (s, 1H, Triazole), 7.69 (m, 1H, Ar), 7.56–7.66 (m, 2H, Ar), 7.21–7.44 (m, 6H, Ar), 6.97–7.21 (m, 10H, Ar), 6.65 (d, J = 8.01 Hz, 2H, Ar), 6.37 (br. s, 1H, CH), 5.75 (m, 1H, CH), 5.36 (d, J = 7.09 Hz, 1H, CH), 5.31–5.34 (m, 1H, CH), 4.98–5.09 (m, 3H, CH), 4.84–4.90 (m, 1H, CH), 4.36–4.52 (m, 3H, CH_2_+CH), 4.19–4.33 (m, 4H, CH_2_+CH), 4.03–4.11 (m, 2H, CH), 3.93–4.03 (m, 4H, CH_2_), 3.14 (m, 2H, CH_2_), 2.97 (m, 7H, CH_2_), 2.77–2.90 (m, 2H, CH_2_), 2.54–2–70 (m, 3H, CH_2_), 2.37–2.46 (m, 4H, CH_2_), 2.30 (d, J = 6.42 Hz, 6H, CH_2_), 2.20–2.26 (m, 6H, CH_2_), 2.19 (m, 4H, CH_2_), 2.15 (m, 2H, CH_2_), 2.04–2.08 (m, 1H, CH_2_), 1.97–2.02 (m, 1H, CH2), 1.72–1.97 (m, 9H, CH_2_), 1.68–1.72 (m, 2H, CH_2_) 1.66 (m, 4H, CH_3_+CH_2_), 1.54–1.64 (m, 5H, CH_2_), 1.47 (m, 11H, CH_3_+CH_2_), 1.29 (s, 10H, CH_3_+CH_2_), 0.98 (d, J = 3.24 Hz, 8H, CH_2_), 0.94 (br. s., 6H, CH_3_).

^13^C NMR (101 MHz, DMSO-d6) δ, ppm: 209.29, 174.39, 174.05, 173.76, 172.45, 172.18, 171.87, 171.81, 171.57, 171.24, 170.89, 169.66, 169.20, 165.40, 157.34, 155.64, 155.23, 148.53, 145.94, 141.01, 140.76, 140.59, 140.28, 139.83, 137.58, 136.76, 136.10, 134.62, 133.57, 133.35, 133.07, 130.62, 130.28, 129.99, 129.88, 129.58, 128.92, 128.74, 128.64, 128.13, 128.00, 127.39, 127.08, 126.88, 126.38, 126.20, 126.00, 124.92, 122.13, 121.86, 118.22, 114.94, 83.80, 80.22, 78.65, 76.75, 75.03, 74.68, 73.62, 73.24, 71.20, 70.67, 56.96, 56.88, 55.22, 52.86, 52.13, 51.51, 49.49, 46.88, 46.65, 42.84, 37.62, 36.25, 35.68, 34.57, 33.99, 32.62, 31.87, 31.52, 31.18, 30.85, 30.61, 29.75, 28.94, 28.73, 28.03, 27.32, 26.38, 26.21, 24.70, 24.25, 24.09, 22.81, 22.47, 20.76, 20.26, 18.87, 15.92, 13.66, 9.78.

HPLC-MS: target compound content—99.9%, t_R_ = 9.96 min.

ESI-HRMS: for C_133_H_169_ClN_14_O_32_: *m*/*z* calculated for [M+H]^+^ 2510.1716, found: 2510.18.

Synthesis of compound **10b**

From 40 mg (24.2 µmol) of compound **8b**, 26 mg (29 µmol) of Docetaxel-alkyne **9**, 6 mg (29 µmol) of sodium ascorbate, 3 mg (9.68 µmol) of copper sulfate pentahydrate in 8 mL DMF/water mixture (3/1), followed by addition of 6 mg (19.4 µmol) EDTA was isolated individually using reverse phase column chromatography (InterchimPuriflashC18 20 g, 15µ, gradient from 15% acetonitrile to 100% acetonitrile in 20 min, flow rate 20 mL/min) 40 mg (65% yield) of compound **10b** as white powder.

^1^H NMR (400 MHz, DMSO-d6) δ, ppm: 8.73 (s, 1H, Py), 8.60 (d, J = 5.01 Hz, 1H, Py), 8.26 (d, J = 7.82 Hz, 1H, Py), 7.94 (m, 2H, Ar), 7.78 (s, 1H, Triazole), 7.66–7.77 (m, 2H, Ar), 7.58–7.66 (m, 2H, Ar), 7.50 (d, J = 8.31 Hz, 1H, Ar), 7.45 (d, J = 8.31 Hz, 1H, Ar), 7.38 (m, 2H, Ar), 7.31 (m, 2H, Ar), 7.13–7.22 (m, 3H, Ar), 7.00–7.13 (m, 7H, Ar), 6.65 (d, J = 7.89 Hz, 2H, Ar), 6.33 (br. s, 1H, CH), 5.74 (t, J = 8.34 Hz, 1H, CH), 5.29–5.40 (m, 2H, CH), 4.97–5.10 (m, 3H, CH+OH), 4.87 (m, 1H, CH), 4.40–4.46 (m, 3H, CH+CH_2_), 4.18–4.33 (m, 4H, CH), 3.92–4.12 (m, 7H, CH_2_+CH), 3.06–3.22 (m, 3H, CH_2_), 2.90–3.06 (m, 8H, CH_2_), 2.80–2.88 (m, 2H, CH_2_), 2.66 (m, 1H, CH_2_), 2.58 (t, J = 7.37 Hz, 2H, CH_2_), 2.42 (m, 5H, CH_2_), 2.26–2.37 (m, 7H, CH_2_), 2.10–2.26 (m, 12H, CH_2_), 1.54–1.92 (m, 21H, CH_2_), 1.47 (m, 12H, CH_2_), 1.33–1.42 (m, 6H, CH_2_), 1.29 (m, 11H, CH_3_+CH_2_), 1.07–1.25 (m, 8H, CH_2_), 0.83–1.05 (m, 15H, CH_3_+CH_2_).

HPLC-MS: target compound content—99.9%, t_R_ = 10.06 min.

ESI-HRMS: for C_133_H_169_BrN_14_O_32_: *m*/*z* calculated for [M+H]^+^ 2554.1211, found: 2554.13.

Synthesis of compound **10c**

From 15 mg (9.26 µmol) of compound **8c**, 10 mg (11.1 µmol) of Docetaxel-alkyne **9**, 2.2 mg (11.1 µmol) of sodium ascorbate, 1 mg (3.70 µmol) of copper sulfate pentahydrate in 6.7 mL of DMF/water mixture (3/1), followed by the addition of 2.2 mg (7.41 µmol) EDTA was isolated individually using reverse phase column chromatography (InterchimPuriflashC18 20 g, 15µ gradient, 15% acetonitrile to 100% acetonitrile in 20 min, flow rate 20 mL/min) 14 mg (77% yield) of compound **10c** as white powder.

^1^H NMR (400 MHz, DMSO-d6) δ, ppm: 8.82 (br. s, 1H, NH), 8.69 (br. s, 1H, NH), 8.49 (d, J = 8.07 Hz, 1H, Py), 7.87–8.01 (m, 4H, Ar+Py), 7.85 (d, J = 8.07 Hz, 1H, Py), 7.78 (s, 1H, Triazole), 7.65–7.75 (m, 1H, Py), 7.57–7.65 (m, 2H, Ar), 7.34–7.47 (m, 2H, Ar), 7.31 (m, 2H, Ar), 7.25 (m, 2H, Ar), 7.13 (m, 2H, Ar), 7.17 (m, 2H, Ar), 6.98–7.11 (m, 4H, Ar), 6.65 (d, J = 6.42 Hz, 2H, Ar), 6.43 (br. s, 1H, CH), 5.69–5.79 (m, 1H, CH), 5.29–5.39 (m, 2H, CH+OH), 4.97–5.08 (m, 3H, CH), 4.87 (d, J = 9.96 Hz, 1H, CH), 4.45–4.57 (m, 2H, CH_2_), 4.40 (br. s, 1H, OH), 4.15–4.32 (m, 4H, CH+CH_2_), 4.05 (m, 2H, CH), 3.91–4.03 (m, 4H, CH+CH_2_), 3.58–3.60 (m, 1H, CH), 3.15 (m, 2H, CH_2_), 2.89–3.09 (m, 8H, CH_2_), 2.84 (m, 2H, CH_2_), 2.64 (m, 1H, CH_2_), 2.58 (m, 2H, CH_2_), 2.42 (d, J = 6.66 Hz, 4H, CH_2_), 2.30 (m, 7H, CH_2_), 2.10–2.26 (m, 11H, CH_2_), 2.03–2.10 (m, 2H, CH_2_), 1.97 (m, 1H, CH_2_), 1.72–1.93 (m, 8H, CH_2_), 1.69 (m, 2H, CH_2_), 1.66 (m, 4H, CH_2_), 1.58 (s, 2H), 1.61 (s, 3H, CH_2_), 1.47 (br. s., 12H, CH_2_), 1.31–1.41 (m, 6H, CH_2_), 1.29 (s, 9H, CH_2_), 1.19 (m, 6H, CH_2_+CH_3_), 1.08 (m, 2H, CH_2_), 0.83–1.06 (m, 15H, CH_2_+CH_3_).

HPLC-MS: target compound content—99.9%, t_R_ = 8.81 min.

ESI-HRMS: for C_134_H_170_N_14_O_34_: *m*/*z* calculated for [M+H]^+^ 2520.2004, found: 2520.21.

Synthesis of compound **10d**

From 48 mg (29.9 µmol) of compound **8d**, 32 mg (35.9 µmol) of Docetaxel-alkyne **9**, 7 mg (35.9 µmol) of sodium ascorbate, 3 mg (11.9 µmol) of copper sulfate pentahydrate in 10.6 mL of DMF/water mixture (3/1), followed by addition of 7 mg (23.9 µmol) EDTA was isolated individually using reverse phase column chromatography (InterchimPuriflash C18 20 g, 15µ, gradient from 15% acetonitrile to 100% acetonitrile in 20 min, flow rate 20 mL/min) 64 mg (85% yield) of compound **10d** as white powder.

^1^H NMR (400 MHz, DMSO-d6) δ, ppm: 8.82 (br. S, 1H, Py), 8.69 (d, J = 5.32 Hz, 1H, Py), 8.51 (d, J = 7.95 Hz, 1H, Py), 7.92–7.99 (m, 3H, Ar), 7.80–7.92 (m, 2H, Py+Ar), 7.78 (s, 1H, Triazole), 7.65–7.75 (m, 1H, Ar), 7.58–7.65 (m, 2H, Ar), 7.28–7.41 (m, 4H, Ar), 7.20–7.28 (m, 7H, Ar), 7.01–7.20 (m, 8H, Ar), 6.44 (br. S, 1H, CH), 5.67–5.79 (m, 1H, CH), 5.36 (d, J = 7.09 Hz, 1H, CH), 5.31 (m, 1H, CH), 4.97–5.08 (m, 3H, CH), 4.87 (d, J = 9.54 Hz, 1H, CH), 4.50–4.57 (m, 2H, CH_2_), 4.32–4.45 (m, 2H, CH), 4.17–4.31 (m, 3H, CH), 3.92–4.12 (m, 7H, CH+CH_2_), 3.59 (d, J = 6.36 Hz, 1H, CH_2_), 3.09–3.22 (m, 3H, CH_2_), 2.89–3.09 (m, 9H, CH_2_), 2.78–2.89 (m, 1H, CH_2_), 2.54–2.68 (m, 3H, CH_2_), 2.41 (d, J = 7.15 Hz, 4H, CH_2_), 2.10–2.36 (m, 18H, CH_2_), 1.77–2.10 (m, 11H, CH_2_), 1.54–1.74 (m, 12H, CH_2_), 1.47 (m, 12H, CH_2_), 1.24–1.41 (m, 16H, CH_3_+CH_2_), 1.06–1.24 (m, 8H, CH_2_), 0.98 (d, J = 5.87 Hz, 8H, CH_3_+CH_2_), 0.94 (br. S, 6H, CH_3_).

HPLC-MS: target compound content—99.9%, t_R_ = 9.49 min.

ESI-HRMS: for C_134_H_170_N_14_O_33_: *m*/*z* calculated for [M+H]^+^ 2504.2055, found: 2504.21.

General procedure for preparing monomodal conjugates **12a**–**d**

Ligand **6a**–**d** (1.1 eq.) and MMAE-alkyne (compound **11**, 1 eq.) were dissolved in a mixture of DMF and water. The flask was filled with argon, then aqueous solutions of sodium ascorbate (1.2 eq.) and copper sulfate pentahydrate (0.4 eq.) were added. The reaction mixture was stirred for 18 h. Afterwards, EDTA (0.8 eq.) was added and stirred for three hours with access to oxygen in air. The solvent was removed under reduced pressure, then the dry residue was precipitated with acetonitrile and decanted. Then, the product was isolated individually using reverse phase column chromatography using acetonitrile–water mixture as eluent.

Synthesis of compound **12a**

From 104 mg (80.5 µmol) of compound **6a**, 89 mg (73.2 µmol) of compound **11**, 17 mg (87.8 µmol) of sodium ascorbate, 6 mg (29.3 µmol) of copper sulfate pentahydrate in 13.5 mL DMF/water mixture (3/1), followed by addition of 17 mg (58.6 µmol) EDTA was isolated individually using reverse phase column chromatography (InterchimPuriflash C18 40 g, 15µ, gradient from 10% acetonitrile to 100% acetonitrile in 30 min, flow rate 20 mL/min) 86 mg (49% yield) of compound **12a** as white powder.

^1^H NMR (400 MHz, DMSO-d6) δ, ppm: 12.51 (br. s, 3H, COOH), 10.00 (m, 1H, NH), 9.22 (br. s, 1H, OH), 8.28 (m, 2H, NH), 8.13 (m, 3H, NH), 7.84–7.96 (m, 3H, NH), 7.82 (s, 2H, Triazole+NH), 7.52–7.74 (m, 6H, Ar+NH), 7.09–7.40 (m, 16H, Ar), 7.00–7.07 (m, 2H, Ar), 6.65 (d, J = 7.82Hz, 2H, Ar), 6.31 (m, 2H, NH), 5.99 (br. s, 1H, NH), 5.42 (br. s, 2H, NH_2_), 4.90–5.13 (m, 2H, CH_2_), 4.59–4.77 (m, 1H, CH), 4.44–4.59 (m, 3H, CH_2_+CH), 4.18–4.43 (m, 8H, CH+CH_2_), 3.90–4.17 (m, 5H, CH), 3.30 (m, 2H, CH_2_), 3.14–3.26 (m, 9H, CH_2_+CH_3_), 3.10 (s, 2H, CH_2_), 2.91–3.07 (m, 9H, CH_2_), 2.70–2.90 (m, 7H, CH_2_), 2.61–2.69 (m, 1H, CH_2_), 2.56 (t, J = 7.49 Hz, 2H, CH_2_), 2.30–2.44 (m, 3H, CH_2_), 2.14–2.29 (m, 9H, CH_2_), 2.11 (m, 1H, CH_2_), 1.84–2.01 (m, 5H, CH_2_), 1.56–1.84 (m, 10H, CH_2_), 1.34–1.56 (m, 13H, CH_2_+CH_3_), 1.10–1.34 (m, 8H, CH_2_), 0.94–1.07 (m, 6H, CH_2_), 0.67–0.89 (m, 23H, CH_2_+CH_3_).

^13^C NMR (101 MHz, DMSO-d6) δ, ppm: 174.50, 174.20, 173.77, 172.52, 172.17, 172.11, 171.71, 171.50, 171.34, 171.10, 170.62, 169.85, 168.75, 158.96, 157.29, 155.88, 146.46, 143.69, 141.22, 137.88, 133.41, 133.05, 130.62, 130.26, 130.05, 129.03, 128.18, 128.08, 127.83, 127.77, 127.19, 126.86, 126.68, 126.43, 126.29, 126.09, 124.97, 121.96, 118.81, 114.98, 114.84, 81.64, 74.82, 66.11, 63.29, 60.95, 58.18, 57.56, 57.14, 54.92, 53.18, 52.57, 52.15, 51.66, 50.28, 49.75, 49.18, 47.23, 46.86, 46.26, 43.77, 43.21, 38.71, 37.14, 36.97, 35.81, 35.06, 34.67, 32.28, 31.81, 31.57, 31.21, 30.79, 30.59, 30.47, 29.91, 29.84, 29.34, 29.10, 27.79, 27.54, 26.83, 26.65, 26.29, 25.43, 24.66, 24.37, 23.14, 22.52, 22.26, 19.28, 18.95, 18.77, 18.56, 18.40, 18.24, 15.91, 15.48, 15.33, 15.05, 10.32.

HPLC-MS: target compound content—99.9%, t_R_ = 11.47 min.

ESI-HRMS: for C_120_H_177_ClN_22_O_27_: *m*/*z* calculated for [M+2H]^2+^ 1197.64938, found: 1197.645.

Synthesis of compound **12b**

From 80 mg (65.5 µmol) of compound **6b**, 73 mg (59.5 µmol) of compound **11**, 14 mg (71.4 µmol) of sodium ascorbate, 6 mg (23.8 µmol) of copper sulfate pentahydrate in 10.8 mL DMF/water mixture (3/1), followed by addition of 14 mg (47.6 µmol) EDTA was isolated individually using reverse phase column chromatography (InterchimPuriflash C18 40 g, 15µ, gradient from 10% acetonitrile to 100% acetonitrile in 30 min, flow rate 20 mL/min) 60 mg (41% yield) of compound **12b** as white powder.

^1^H NMR (400 MHz, DMSO-d6) δ, ppm: 12.52 (br. s, 3H, COOH), 9.99 (m, 1H, NH), 9.20 (br. s, 1H, OH), 8.24–8.37 (m, 2H, NH), 8.11–8.15 (m, 3H, NH), 7.84–7.96 (m, 3H, NH), 7.82 (s, 2H, NH+Triazole), 7.59–7.70 (m, 4H, Ar), 7.51–7.59 (m, 3H, NH+Ar), 7.48 (d, J = 8.31 Hz, 1H, Ar), 7.08–7.35 (m, 13H, Ar), 7.03 (m, 2H, Ar), 6.65 (d, J = 7.76 Hz, 2H, Ar), 6.29–6.33 (m, 2H, NH), 6.00 (br. s, 1H, NH), 5.41 (br. s, 2H, NH), 4.95–5.14 (m, 2H, CH_2_), 4.62–4.73 (m, 1H, CH), 4.23–4.53 (m, 10H, CH+CH_2_), 3.38–4.23 (m, 27H, H_2_O+CH+CH_2_), 3.08–3.26 (m, 10H, CH_2_+CH_3_), 2.78–3.08 (m, 13H, CH_2_+CH_3_), 2.70–2.78 (m, 2H, CH_2_), 2.60–2.69 (m, 1H, CH_2_), 2.52–2.60 (m, 2H, CH_2_), 2.15–2.42 (m, 11H, CH_2_), 2.11 (m, 1H, CH_2_), 1.84–2.04 (m, 5H, CH_2_), 1.63–1.84 (m, 7H, CH_2_), 1.33–1.58 (m, 13H, CH_2_+CH_3_), 1.11–1.33 (m, 7H, CH_2_), 0.89–1.08 (m, 6H, CH_2_), 0.65–0.89 (m, 21H, CH_2_+CH_3_).

^13^C NMR (101 MHz, DMSO-d6) δ, ppm: 174.51, 174.22, 173.78, 172.37, 172.09, 171.71, 171.50, 171.35, 171.10, 168.74, 158.96, 157.28, 155.88, 146.45, 143.70, 138.03, 137.88, 131.57, 131.25, 130.05, 129.73, 129.03, 128.65, 128.19, 128.08, 127.83, 126.47, 121.97, 119.93, 114.98, 74.80, 60.95, 60.31, 57.18, 54.92, 54.15, 51.65, 46.86, 43.21, 38.70, 37.48, 36.99, 35.80, 34.67, 31.80, 30.48, 29.91, 29.02, 27.54, 26.65, 26.30, 25.43, 24.65, 24.36, 22.27, 19.28, 18.24, 15.49, 15.32, 10.31.

HPLC-MS: target compound content—99.9%, t_R_ = 11.68 min.

ESI-HRMS: for C_120_H_177_BrN_22_O_27_: *m*/*z* calculated for [M+2H]^2+^ 1219.62412, found: 1219.6244.

Synthesis of compound **12c**

From 100 mg (84.2 µmol) of compound **6c**, 93 mg (76.6 µmol) of compound **11**, 18 mg (91.9 µmol) sodium ascorbate, 8 mg (30.6 µmol) copper sulfate pentahydrate in 13.5 mL DMF/water mixture (3/1), followed by the addition of 18 mg (61.3 µmol) EDTA was isolated individually using reverse phase column chromatography (InterchimPuriflash C18 40 g, 15µ, gradient from 10% acetonitrile to 100% acetonitrile in 30 min, flow rate 20 mL/min) 101 mg (55% yield) of compound **12c** as white powder.

^1^H NMR (400 MHz, DMSO-d6) δ, ppm: 12.62 (br. s, 3H, COOH), 9.99 (d, J = 7.46 Hz, 1H, NH), 9.22 (br. s, 1H, OH), 8.24–8.37 (m, 2H, NH), 8.10–8.16 (m, 3H, NH), 7.84–7.96 (m, 5H, Ar+NH), 7.82 (s, 2H, NH+Triazole), 7.54–7.66 (m, 6H, Ar+NH), 7.10–7.35 (m, 15H, Ar), 7.03 (dd, J = 8.41, 3.82 Hz, 2H, Ar), 6.64 (dd, J = 8.44, 1.96 Hz, 2H, Ar), 6.23–6.40 (m, 2H, NH), 6.00 (br. s, 1H, NH), 5.41 (br. s, 2H, NH_2_), 4.91–5.13 (m, 2H, CH_2_), 4.73 (br. s, 1H, CH), 4.18–4.66 (m, 13H, CH+CH_2_+CH_3_), 4.03–4.18 (m, 4H, CH), 3.89–4.03 (m, 4H, CH), 3.13–3.27 (m, 10H, CH_2_+CH_3_), 2.91–3.10 (m, 12H, CH_2_), 2.79–2.91 (m, 5H, CH_2_), 2.54–2.79 (m, 6H, CH_2_), 2.13–2.39 (m, 12H, CH_2_), 2.11 (m, 1H, CH_2_), 1.84–2.01 (m, 5H, CH_2_), 1.34–1.84 (m, 24H, CH_2_+CH_3_), 1.10–1.34 (m, 9H, CH_2_), 0.93–1.08 (m, 7H, CH_2_), 0.64–0.90 (m, 26H, CH_2_+CH_3_).

^13^C NMR (101 MHz, DMSO-d6) δ, ppm: 174.50, 174.21, 173.77, 172.53, 172.31, 172.10, 171.71, 171.51, 171.34, 171.10, 170.60, 169.91, 169.85, 168.74, 167.21, 158.96, 157.89, 157.28, 155.88, 146.45, 143.77, 143.69, 137.88, 130.05, 129.78, 129.47, 129.02, 128.19, 128.08, 127.83, 127.77, 127.39, 126.67, 126.47, 126.42, 126.28, 121.96, 118.95, 114.98, 89.66, 74.80, 60.95, 60.31, 58.68, 57.57, 57.17, 54.95, 54.14, 53.15, 52.57, 52.12, 51.65, 46.87, 40.44, 38.71, 37.02, 35.81, 34.68, 32.32, 31.78, 31.54, 31.22, 30.80, 30.48, 29.91, 29.84, 29.35, 29.12, 27.53, 26.65, 26.31, 25.43, 25.37, 24.66, 24.36, 23.14, 22.52, 22.25, 19.28, 18.98, 18.24, 15.49, 15.32, 15.05, 10.33.

HPLC-MS: target compound content—99.9%, t_R_ = 11.32 min.

ESI-HRMS: for C_121_H_178_N_22_O_29_: *m*/*z* calculated for [M+2H]^2+^ 1202.66378, found: 1202.664.

Synthesis of compound **12d**

From 50 mg (42.7 µmol) of compound **6d**, 47 mg (38.8 µmol) of compound **11**, 9 mg (46.6 µmol) of sodium ascorbate, 4 mg (15.5 µmol) of copper sulfate pentahydrate in 9.5 mL DMF/water mixture (3/1), followed by addition of 9 mg (31.1 µmol) EDTA was isolated individually using reverse phase column chromatography (InterchimPuriflash C18 40 g, 15µ, gradient from 10% acetonitrile to 100% acetonitrile in 30 min, flow rate 20 mL/min) 43 mg (45% yield) of compound **12d** as white powder.

^1^H NMR (400 MHz, DMSO-d6) δ, ppm: 12.45 (br. s, 3H), 9.99 (br. s, 1H, NH), 8.23–8.39 (m, 2H, NH), 8.02–8.23 (m, 2H, NH), 7.79–8.01 (m, 6H, Ar+NH+Triazole), 7.56–7.74 (m, 5H, NH), 7.10–7.38 (m, 16H, Ar), 6.17–6.44 (m, 2H, NH), 6.00 (br. s, 1H, NH), 4.93–5.18 (m, 3H, CH_2_), 4.72 (br. s, 1H, CH), 4.24–4.66 (m, 15H, CH+CH_2_+CH_3_+H_2_O), 3.56 (d, J = 5.20 Hz, 1H, CH_2_), 3.28–3.58 (m, 4H, CH_2_), 3.08–3.26 (m, 10H, CH_3_+CH_2_), 2.79–3.08 (m, 12H, CH_2_), 2.75 (br. s, 2H, CH_2_), 2.53–2.61 (m, 2H, CH_2_), 2.08–2.40 (m, 10H, CH_2_), 1.85–2.00 (m, 4H, CH_2_), 1.62–1.84 (m, 7H, CH_2_), 1.34–1.62 (m, 12H, CH_2_+CH_3_), 1.09–1.34 (m, 7H, CH_2_), 0.94–1.08 (m, 5H, CH_2_), 0.66–0.92 (m, 19H, CH_2_+CH_3_).

^13^C NMR (101 MHz, DMSO-d6) δ, ppm: 174.55, 174.50, 174.21, 173.78, 172.54, 172.09, 171.75, 171.53, 171.33, 170.94, 168.74, 158.95, 157.28, 146.46, 143.70, 137.83, 129.78, 129.46, 129.37, 129.12, 129.01, 128.18, 128.08, 127.83, 127.77, 127.39, 126.76, 126.45, 121.98, 74.81, 57.14, 54.50, 52.60, 51.65, 46.87, 38.71, 35.83, 31.80, 30.55, 29.91, 29.12, 27.55, 26.65, 26.33, 25.44, 24.70, 23.14, 22.27, 19.28, 18.96, 18.24, 15.49, 15.32, 15.06, 10.32.

HPLC-MS: target compound content—99.9%, t_R_ = 11.46 min.

ESI-HRMS: for C_121_H_178_N_22_O_28_: *m*/*z* calculated for [M+2H]^2+^ 1194.66632, found: 1194.6669.

General methodology for producing bimodal conjugates **13a**–**d**

Monoconjugate **12a**–**d** (1 eq.) was dissolved in DMF to which NHS-ester of Abiraterone (compound **7**, 1.2 eq.) and DIPEA (6 eq.) were added. The reaction mixture was stirred for 20 h, after which the solvent was removed under reduced pressure. The dry residue was precipitated with acetonitrile, the resulting precipitate was decanted and washed three times with acetonitrile. The solvent residue was removed under reduced pressure.

Synthesis of compound **13a**

From 34 mg (14.2 µmol) of compound **12a**, 9 mg (17.0 µmol) of compound **7** and 15 µL (85.1 µmol) of DIPEA, 38 mg (95% yield) of compound **13a** was obtained as white powder.

^1^H NMR (400 MHz, DMSO-d6) δ, ppm: 12.46 (br. s, 3H, COOH), 9.99 (br. s, 1H, NH), 9.20 (m, 1H, OH), 8.58 (br. s, 1H, Py), 8.42 (m, 1H, Py), 8.33 (d, J = 6.58 Hz, 1H, Py), 8.03–8.21 (m, 3H, NH), 7.83–8.01 (m, 4H, NH), 7.81 (s, 1H, Triazole), 7.68–7.78 (m, 2H, Ar), 7.51–7.67 (m, 4H, Ar), 7.08–7.40 (m, 18H, Ar), 7.04 (d, J = 8.50 Hz, 2H, Ar), 6.64 (d, J = 7.95 Hz, 2H, Ar), 6.23–6.37 (m, 2H, NH), 6.10 (s, 1H, CH), 5.98 (m, 1H, NH), 5.42 (br. s, 2H, NH_2_), 5.35 (m, 1H, CH), 4.91–5.15 (m, 2H, CH_2_), 4.59–4.77 (m, 1H, CH), 4.16–4.59 (m, 12H, CH_2_+CH), 4.03–4.16 (m, 3H, CH), 3.88–4.03 (m, 2H, CH), 3.57 (m, 1H, CH), 3.13–3.26 (m, 9H, CH_2_+CH_3_), 3.11 (s, 2H, CH_2_), 2.90–3.08 (m, 11H, CH_2_+CH), 2.77–2.89 (m, 5H, CH_2_), 2.60–2.70 (m, 1H, CH_2_), 2.53–2.60 (m, 2H, CH_2_), 2.43 (m, 2H, CH_2_), 2.13–2.40 (m, 7H, CH_2_), 1.85–2.13 (m, 4H, CH_2_), 1.74–1.84 (m, 2H, CH_2_), 1.56–1.74 (m, 4H, CH_2_), 1.44–1.56 (m, 4H, CH_2_+CH_3_), 1.11–1.43 (m, 7H, CH_2_+CH_3_), 0.92–1.11 (m, 6H, CH_2_), 0.67–0.92 (m, 10H, CH_2_+CH_3_).

^13^C NMR (101 MHz, DMSO-d6) δ, ppm: 174.50, 174.20, 173.77, 172.38, 172.31, 172.10, 171.79, 171.58, 171.48, 171.34, 171.09, 170.56, 169.90, 169.84, 168.75, 158.92, 157.27, 155.86, 151.01, 147.84, 147.20, 146.40, 143.69, 141.22, 140.81, 139.87, 137.86, 133.33, 133.05, 132.16, 130.59, 130.24, 130.00, 129.01, 128.17, 128.05, 127.94, 127.76, 127.21, 126.75, 126.42, 126.25, 126.08, 124.96, 123.43, 121.98, 121.92, 118.98, 114.98, 85.43, 74.81, 73.17, 60.94, 60.30, 58.67, 58.19, 57.59, 57.14, 56.99, 54.99, 54.17, 53.17, 52.88, 52.17, 51.68, 49.63, 49.18, 47.16, 46.84, 46.66, 46.27, 43.78, 43.22, 38.44, 37.71, 36.42, 36.31, 35.77, 34.67, 34.55, 32.29, 31.87, 31.54, 31.33, 30.96, 30.75, 30.57, 30.45, 29.95, 29.89, 29.36, 29.10, 28.91, 27.80, 27.60, 27.35, 26.84, 26.30, 25.41, 24.66, 24.36, 23.14, 22.91, 22.35, 20.40, 19.27, 18.93, 18.58, 18.24, 16.28, 15.87, 15.48, 15.30, 15.04, 10.43.

HPLC-MS: target compound content—99%, t_R_ = 1.64 min.

ESI-HRMS: for C_148_H_210_ClN_23_O_30_: *m*/*z* calculated for [M+2Na]^2+^ 1435.25435, found: 1435.2559.

Synthesis of compound **13b**

From 35 mg (14.4 µmol) of compound **12b**, 10 mg (17.2 µmol) of compound **7** and 16 µL (86.1 µmol) of DIPEA, 33 mg (80% yield) of compound **13b** was obtained as white powder.

^1^H NMR (400 MHz, DMSO-d6) δ, ppm: 12.40 (br. s, 3H, COOH), 9.98 (br. s, 1H, NH), 9.21 (br. s, 1H, OH), 8.58 (br. s, 1H, Py), 8.39–8.46 (m, 1H, Py), 8.32 (d, J = 6.9 Hz, 1H, Ar), 8.10–8.19 (m, 2H, Ar+NH), 7.96–8.10, (m, 1H, NH), 7.82–7.96, (m, 4H, Ar+NH+Py), 7.78–7.82 (s, 1H, Triazole), 7.74 (d, J = 8.7 Hz, 1H, Ar), 7.50–7.65 (m, 4H, Ar+NH), 7.47 (d, J = 6.1 Hz, 1H, Ar), 7.08–7.41 (m, 16H, Ar), 7.04 (d, J = 6.7 Hz, 2H, Ar), 6.64 (d, J = 8.3 Hz, 2H, Ar), 6.24–6.36 (m, 2H, NH), 6.10 (br. s, 1H, CH), 5.97 (br. s, 1H, NH), 5.32–5.45 (m, 3H, NH_2_+CH), 4.92–5.12 (m, 2H, CH_2_), 4.56–4.79 (m, 1H, CH_2_), 4.18–4.51 (m, 12H, CH+CH_2_), 3.90–4.14 (m, 5H, CH+CH_2_), 3.51–3.60 (m, 1H, CH_2_), 3.08–3.24 (m, 12H, CH_2_), 2.90–3.06 (m, 12H, CH_2_+CH_3_), 2.80–2.90 (m, 5H, CH_2_), 2.52–2.70 (m, 3H, CH_2_), 2.15–2.35 (m, 16H, CH_2_), 1.85–2.13 (m, 10H, CH_2_), 1.74–1.84 (m, 5H, CH_2_), 1.63–1.74 (m, 6H, CH_2_), 1.55–1.63 (m, 4H, CH_2_), 1.44–1.55 (m, 9H, CH_2_), 1.29–1.44 (m, 9H, CH_2_), 1.12–1.28 (m, 7H, CH_2_), 0.94–1.05 (m, 13H, CH_2_), 0.70–0.88 (m, 25H, CH_2_+CH_3_).

^13^C NMR (101 MHz, DMSO-d6) δ, ppm: 174.53, 174.24, 173.81, 172.38, 172.11, 171.80, 171.60, 171.49, 171.35, 171.12, 170.57, 169.85, 168.76, 158.93, 157.28, 155.87, 151.00, 147.84, 147.19, 146.40, 143.69, 139.87, 137.85, 133.33, 132.14, 131.56, 131.24, 130.01, 129.73, 129.01, 128.66, 128.18, 128.06, 127.94, 127.83, 127.77, 126.74, 126.47, 126.27, 123.45, 121.99, 120.15, 119.93, 118.96, 114.99, 81.64, 74.80, 73.17, 60.95, 60.31, 57.59, 57.17, 56.99, 55.02, 54.17, 53.17, 52.89, 52.13, 51.69, 49.63, 46.83, 46.65, 46.28, 43.78, 43.23, 38.44, 37.71, 36.41, 36.31, 35.83, 34.66, 34.54, 31.80, 31.33, 30.96, 30.45, 29.89, 29.36, 29.10, 28.91, 27.74, 27.62, 27.35, 26.84, 26.32, 25.41, 24.73, 24.65, 24.36, 22.92, 22.36, 20.40, 19.27, 18.94, 18.55, 18.25, 16.28, 15.86, 15.65, 15.48, 15.31, 15.05, 10.32.

HPLC-MS: target compound content—99.9%, t_R_ = 9.27 min.

ESI-HRMS: for C_148_H_210_BrN_23_O_30_: *m*/*z* calculated for [M+2Na]^2+^ 1457.22909, found: 1457.2319.

Synthesis of compound **13c**

From 50 mg (20.8 µmol) of compound **12c**, 14 mg (25.0 µmol) of compound **7** and 22 µL (0.125 mmol) of DIPEA, 49 mg (83% yield) of compound **13c** was obtained as white powder.

^1^H NMR (400 MHz, DMSO-d6) δ, ppm: 12.47 (br. s, 4H, COOH), 9.98 (br. s, 1H, NH), 9.21 (br. s, 1H, OH), 8.58 (m, 1H, Py), 8.42 (m, 1H, Py), 8.24–8.36 (m, 2H, NH), 8.10–8.17 (m, 2H, NH+Ar), 8.02–8.10 (m, 1H, NH) 7.82–8.01 (m, 5H, Py+Ar+NH), 7.81 (s, 1H, Tetrazole), 7.67–7.77 (m, 2H, Ar+NH), 7.53–7.64 (m, 3H, Ar), 7.22–7.33 (m, 9H, Ar), 7.16–7.22 (m, 2H, Ar), 7.10–7.16 (m, 4H, Ar), 7.00–7.07 (m, 2H, Ar), 6.64 (d, J = 8.5 Hz, 2H, Ar), 6.25–6.34 (m, 2H, NH), 6.10 (s, 1H, CH), 5.97 (br. s, 1H, NH), 5.30–5.44 (m, 4H, NH_2_+CH_2_), 4.91–5.10 (m, 2H, CH), 4.68–4.87 (m, 1H, CH), 6.50–4.61 (m, 2H, CH_2_), 4.39–4.50 (m, 3H, CH+CH_2_), 4.30–4.39 (m, 3H, CH+CH_2_), 4.16–4.30 (m, 5H, CH_2_+CH), 4.04–4.14 (m, 3H, CH), 3.89–4.03 (m, 3H, CH), 3.50–3.60 (m, 1H, CH), 3.12–3.24 (m, 11H, CH_2_+CH_3_), 3.10 (br. s, 2H, CH_2_), 2.90–3.07 (m, 13H, CH_2_), 2.80–2.89 (m, 5H, CH_2_), 2.61–2.69 (m, 1H, CH_2_), 2.56 (t, J = 7.4 Hz, 2H, CH_2_), 2.41–2.46 (m, 3H, CH_2_), 2.16–2.34 (m, 16H, CH_2_), 1.84–2.07 (m, 10H, CH_2_), 1.74–1.83 (m, 5H, CH_2_), 1.56–1.74 (m, 11H, CH_2_), 1.29–1.56 (m, 10H, CH_2_), 1.11–1.29 (m, 8H, CH_2_), 0.95–1.05 (m, 14H, CH_2_), 0.70–0.89 (m, 25H, CH_2_+CH_3_).

^13^C NMR (101 MHz, DMSO-d6) δ, ppm: 174.55, 174.22, 173.79, 172.37, 172.31, 172.12, 171.79, 171.60, 171.49, 171.35, 171.11, 170.57, 169.84, 168.75, 167.22, 158.93, 157.28, 155.86, 151.01, 147.81, 147.16, 146.40, 143.76, 143.69, 139.86, 137.85, 133.30, 130.01, 129.77, 129.46, 129.01, 128.18, 128.06, 127.94, 127.82, 127.77, 127.39, 126.67, 126.47, 126.27, 121.98, 121.92, 118.98, 114.98, 85.42, 74.80, 73.17, 60.95, 60.31, 58.18, 57.60, 57.14, 56.98, 55.07, 53.18, 52.89, 52.15, 51.68, 49.62, 49.18, 46.83, 46.65, 46.27, 43.22, 38.43, 37.71, 36.41, 36.31, 35.86, 34.67, 34.53, 31.82, 31.50, 31.33, 30.96, 30.74, 30.46, 29.94, 29.88, 29.35, 28.90, 27.58, 27.34, 26.84, 26.23, 25.41, 24.65, 24.38, 23.14, 22.91, 22.37, 20.39, 19.27, 18.93, 18.41, 18.25, 16.27, 15.87, 15.65, 15.48, 15.31, 15.05, 10.32.

HPLC-MS: target compound content—99.9%, t_R_ = 8.91 min.

ESI-HRMS: for C_149_H_211_N_23_O_32_: *m*/*z* calculated for [M+2Na]^2+^ 1440.26875, found: 1440.2652.

Synthesis of compound **13d**

From 38 mg (15.9 µmol) of compound **12d**, 11 mg (19.1 µmol) of compound **7** and 17 µL (95.5 µmol) of DIPEA, 35 mg (78% yield) of compound **13d** was obtained as white powder.

^1^H NMR (400 MHz, DMSO-d6) δ, ppm: 12.53 (br. s, 3H, COOH), 9.98 (br. s, 1H, NH), 8.57 (br. s, 1H, Py), 8.42 (br. s, 1H, Py), 8.05–8.36 (m, 4H, NH), 7.79–8.01 (m, 7H, Ar+Py+Triazole+NH), 7.74 (d, J = 7.46 Hz, 2H, NH), 7.53–7.70 (m, 3H, NH+Ar), 7.02–7.40 (m, 21H, Ar), 6.30 (m, 2H, NH), 6.10 (br. s, 1H, CH), 5.98 (br. s, 1H, NH), 5.35–5.42 (m, 3H, NH+CH), 4.93–5.14 (m, 2H, CH_2_), 4.72 (br. s, 1H, CH), 4.34–4.66 (m, 8H, CH_2_+CH), 4.16–4.33 (m, 5H, CH_2_+CH), 3.88–4.16 (m, 5H, CH), 3.14–3.25 (m, 11H, CH_2_), 2.79–3.14 (m, 20H, CH_2_+CH_3_), 2.53–2.70 (m, 4H, CH_2_), 2.43 (d, J = 5.93 Hz, 2H, CH_2_), 1.84–2.40 (m, 29H, CH_2_), 1.10–1.84 (m, 43H, CH+CH_2_+CH_3_), 0.93–1.10 (m, 15H, CH_2_), 0.68–0.93 (m, 26H, CH_2_+CH_3_).

^13^C NMR (101 MHz, DMSO-d6) δ, ppm: 174.53, 174.23, 173.80, 172.10, 171.80, 171.62, 171.35, 170.96, 170.57, 169.85, 168.74, 156.93, 150.99, 147.18, 146.40, 143.69, 139.86, 137.93, 137.82, 133.34, 129.76, 129.46, 129.08, 129.00, 128.18, 128.07, 127.83, 127.38, 126.68, 126.46, 123.45, 122.00, 121.92, 118.93, 81.75, 77.71, 74.80, 73.17, 60.95, 60.31, 57.59, 57.14, 56.98, 54.99, 54.67, 53.17, 52.92, 49.62, 46.84, 46.64, 46.26, 43.24, 37.70, 36.40, 36.31, 35.79, 34.54, 31.81, 31.54, 31.33, 30.96, 30.46, 29.87, 29.34, 29.12, 28.91, 27.63, 27.34, 26.85, 26.32, 25.42, 24.65, 24.37, 23.14 CH2 (), 22.91, 20.40, 19.27, 18.93, 18.42, 18.25, 16.27, 15.48, 15.31, 15.05, 10.31.

HPLC-MS: target compound content—96%, t_R_ = 8.39 min.

ESI-HRMS: for C_149_H_211_N_23_O_31_: *m*/*z* calculated for [M+2Na]^2+^ 1432.27129, found: 1432.2719.

Synthesis of a bimodal conjugate with the MMAE/Enzalutamide drug pair **15**

86 mg (35.9 µmol, 1 equiv.) of compound **12a** and 24 mg (43.1 µmol, 1.2 equiv.) of compound **14** were dissolved in 20 mL of DMF. To the resulting solution, 38 μL (0.215 mmol, 6 eq.) of DIPEA was added. The reaction mixture was stirred for 24 h, after which the solvent was removed under reduced pressure. Then, the product was precipitated with acetonitrile, decanted and the resulting precipitate was washed with acetonitrile. The target product was isolated individually using reverse phase column chromatography using a mixture of acetonitrile and water (InterchimPuriflash C18 20 g, 15 µ, gradient from 10% acetonitrile to 100% acetonitrile in 30 min, flow rate 20 mL/min) as eluent. 20 mg (20% yield) of compound **15** was obtained as white powder.

^1^H NMR (400 MHz, DMSO-d6) δ, ppm: 12.49 (br. s., 3H, COOH), 9.99 (br. s., 1H, NH), 9.20 (br. s, 1H, OH), 8.35–8.62 (m, 1H, NH), 8.28 (br. s., 2H, NH), 8.02–8.20 (m, 3H, Ar+NH), 7.70–7.98 (m, 6H, Ar), 7.49–7.69 (m, 3H, Ar), 6.94–7.45 (m, 21H, Ar), 6.63 (br. s., 2H, Ar), 6.29 (br. s., 2H, NH), 5.97 (br. s., 1H, NH), 5.29–5.47 (m, 2H, NH), 5.03 (br. s., 1H, CH_2_), 4.72 (br. s., 1H, CH), 4.18–4.65 (m, 13H, CH_2_+CH), 3.87–4.15 (m, 6H, CH_2_+CH), 3.50–3.80 (m, 3H, CH_2_+CH), 3.08–3.26 (m, 14H, CH_2_), 2.75–3.07 (m, 18H, CH_2_), 2.62–2.74 (m, 3H, CH_2_), 2.03–2.36 (m, 16H, CH_2_), 1.90 (m, 6H, CH_2_), 1.63–1.83 (m, 10H, CH_2_), 1.09–1.61 (m, 28H, CH_2_+CH_3_), 0.99 (d, J = 5.14 Hz, 8H, CH_2_+CH_3_), 0.64–0.89 (m, 26H, CH_2_+CH_3_).

HPLC-MS: target compound content—99.9%, t_R_ = 9.20 min.

ESI-HRMS: for C_140_H_188_ClF_4_N_25_O_29_S: *m*/*z* calculated for [M+2Na]^2+^ 1436.15673, found: 1436.1587.

### 3.3. In Vitro Affinity Studies of the Synthesized Ligands ***6a**–**d***

LNCaP cells were cultured in RPMI medium with 10% FBS (Gibco, Billings, MT, USA), 1xGlutamax (Gibco) and 1x penicillin-streptomycin mixture (Gibco, USA). To suspend the cells, growth medium was removed from the 25 cm^2^ vial, the cells were washed with PBS buffer and incubated for 5 min with 0.25% trypsin (1 mL). Trypsin was inactivated with complete growth medium (2 mL), washed with PBS, transferred 10^6^ cells to a test tube, and 500 μL of lysis buffer (0.5% Triton X-100, 50 mM Tris-HCl (pH 7.5) and 1× Proteinase Inhibition cocktail) was added. The suspension was incubated for 30 min on ice and sonicated (seven times 7 sec at 20–30 s intervals) on ice to avoid overheating. The suspension was centrifuged for 10 min at 1000g at 4 °C, and the supernatant was used in further analysis.

The inhibition by PSMA ligands was analyzed using a non-radioactive protocol with detection of the glutamate released during the reaction. LNCaP cell line extract (10 μL) was mixed with 2 μL of the appropriate compound preparation. A series of dilutions of 2 nM-100 μM compounds was used for initial testing, with dilution steps of 3–5 times. To the resulting mixture, 1 μL of 100 μM NAAG solution was added. The mixture was incubated for 2 h at 37 °C. After incubation, the mixture was doubled with reaction buffer (13 μL) from the Amplex Red Glutamic Acid Kit (Molecular Probes Inc., Eugene, OR, USA), and the multi-component glutamate detection reaction mixture prepared according to the manufacturer’s protocol (26 μL) was added. Amplex Red stock solution: 5 μL Amplex^®^ Red reagent stock solution, 1.25 μL HRP stock solution, 8 μL L-glutamate oxidase, 2.5 μL L-glutamate pyruvate transaminase, 0.5 μL L-alanine, 483 μL 1× reaction buffer. Incubation was performed for 1 h at 37 °C. The fluorescence of resorufin produced by conjugated glutamate detection kit reactions was detected on a VICTORX5 multidetector plate (Perkin Elmer Inc., Waltham, MA, USA) at an excitation length of 555 nm and detection at 580 nm. As a control for endogenous glutamate levels with replacement of the NAAG solution with water.

### 3.4. In Vitro Cytotoxicity Studies of the Obtained Compounds

The cytotoxicity of the investigated substances was tested using the MTS assay [37] with some modifications. Cells were seeded in the 96-well plates (Corning, New York, NY, USA) at concentration of 7000 cells per well for PC-3 culture, 12,000 cells per well for LNCaP cells and 20,000 cells per well for 22Rv1 line. The cells were counted using the automatic cell counter EVE. All cell lines were purchased from the American Type Culture Collection (ATCC, Manassas, VA, USA). After 1 day, serial dilutions of tested substances were added to the cells. Cell culture medium with addition of DMSO in appropriate concentrations was used as a negative control. DMSO (30%) was added in culture medium as a positive control. Cells were then incubated during 72 h at 37 °C and 5% CO_2_. Later, the culture medium from each well was removed and 20 μL of MTS reagent (CellTiter 96 AQueous Non-Radioactive Cell Proliferation Assay, Promega) were added to each well with 100 μL of new culture medium. After 4 h incubation at 37 °C in darkness, the absorbance of the solution was measured at 490 nm wavelength using Thermo Scientific Multiskan GO spectrometer. Cell viability was calculated as a percentage compared to control cells. The absorbance of MTS reagent in culture medium without cells was taken as zero. MTS assay revealed 100% cell death after incubation with 30% DMSO. Experiments were performed in triplicates. Data were analyzed using Prism 9—GraphPad version 9.2.0 software. *p* values < 0.05 were considered significant.

### 3.5. Human Tumor Xenograft Mouse Model

#### 3.5.1. Animals

All animal procedures were conducted in strict adherence to the European Convention for the Protection of Vertebrate Animals Used for Experimental and other Scientific Purposes (ETS 123), Strasbourg, 1986. The procedures with animals were reviewed and approved by the bioethical committee of the National Medical Research Radiological Centre of the Ministry of Health of the Russian Federation, Protocol No. 23 from 22 June 2021.

BALB/c nu/nu (nude) mice, males with body weights of 21–25 g (aged 8−10 weeks) were used. Animals were obtained from SPF-vivarium of the Center for Collective Use in Novosibirsk. All animals came with a veterinary passport and a certificate of quality.

#### 3.5.2. Human Tumor Xenograft Mouse Model

Two cell lines were used: human prostate carcinoma 22Rv1 and prostate adenocarcinoma PC-3 (ATCC collection). Cells were cultured in 75 cm^2^ vials (Costar, Washington, DC, USA) in RPMI-1640 medium (PanEco, Russia) with the addition of 2 mM L-glutamine (PanEco, Russia) and 10% fetal calf serum (HyClone, Logan, UT, USA) under standard conditions of cultivation (in a humidified atmosphere at 37 °C and 5% CO_2_). Cells of 6–10 passages were used to inoculate the animals. Cell suspension was prepared with a concentration of 5 × 10^6^ cells in 0.05 mL of the 22Rv1 medium and in 0.1 mL of the PC-3 medium. 22Rv1 cells were inoculated into animals in Matrigel (BD Matrigel Basement Membrane Matrix, BD Biosciences, San Jose, CA, USA). Manipulations with Matrigel were performed on a refrigerated plate using precooled materials, tubes and instrumentation. For animal administration, 50 μL of the cell suspension (5 × 10^6^ cells) was transferred to a chilled tube containing 50 μL of Matrigel; after mixing, the cellular material (final volume of 100 μL) was injected subcutaneously into the groin area of the animal. To obtain subcutaneous xenograft PC-3, a 5 × 10^6^ suspension of tumor cells was inoculated subcutaneously into the inguinal cavity of male mice in a volume of 0.1 mL.

#### 3.5.3. Preparation of Material for Histological and Immunohistochemical Testing

Tissue samples obtained by the autopsy of animals on 14–21 days after cell inoculation were fixed in neutral buffered 10% formalin and encased in paraffin after a standard histological examination. Serial tissue sections with a 4 μm thickness were prepared. For histological analysis, the sections were stained with hematoxylin and eosin (H&E) according to the standard technique.

#### 3.5.4. Immunohistochemical Staining

Polyclonal rabbit antibodies Ab58779 (Abcam, United Kingdom) were used to detect PSMA expression at a dilution of 1:100. Sections were stained using a common indirect immunoperoxidase assay technique. To demask the antigen, glasses with deparaffinized sections were incubated in 0.1 M sodium citrate buffer (pH 6.0) at 95 °C for 20 min. The system of secondary reagents included biotinylated polyclonal antibodies to rabbit immunoglobulins (Santa Cruz Biotechnology, Inc., Santa Cruz, CA, USA), streptavidin conjugate with horseradish peroxidase (Dako, Denmark) and the chromogenic substrate Liquid DAB + Substrate Chromogen System (Dako, Denmark). Non-specific rabbit immunoglobulins (Santa Cruz Biotechnology, Inc.) were applied to control sections instead of primary antibodies. After the completion of the reaction, cells and sections were stained with hematoxylin and encapsulated in Canada balsam. Micropreparations were analyzed under an Olympus BX51 microscope equipped with an image documentation system.

#### 3.5.5. Evaluation of In Vivo Antitumor Efficacy

To study the antitumor efficacy, conjugates were administered intravenously at a dose of 132.3 nM/kg to tumor-bearing mice three times at five-day intervals; administration was started 7−8 days after cell inoculation, and the tumor size was 70−90 mm^3^. As a control substance, 0.9% sodium chloride solution was used. Experimental groups included five animals each. Conjugates **13a**, **15**, **I** and MMAE were formulated with Pluronic F-127 (the compound/Pluronic F-127 F weight ratio was 1:5). The mixture was dissolved in DMSO (5% of final solution) and diluted with hemodesum (30% low-molecular-weight polyvinylpyrrolidone (M_w_ = 8000 ± 200) saline infusion solution). The presence of tumors and their sizes, as well as the body weights of the mice, were recorded every five days during the observation of the animals. The antitumor effect was assessed by comparing the tumor volumes in the experimental and control groups as well as the amount of tumor growth inhibition (TGI, %).

In line with humane principles, animals were euthanized by placing mice in a CO_2_ inhalation chamber (ZOONLAB GmbH, Germany). All animals were euthanized either once the tumor volumes in the control group reached 2500 mm^3^ or due to the presence of necrosis or ulceration unrelated to tumor growth.

The data were statistically processed using the Statistica 8.0 software package (StatSoft, Inc., Tulsa, OK, USA). Group arithmetic mean (M) and standard error of the mean (m) as well as median (Me) were calculated for all quantitative data. Student’s *t*-test or Mann–Whitney U-test were used to assess the reliability of differences between the groups, depending on the nature of the distribution of the trait in the groups.

Tumor volume was calculated using Formula (1):

V = d_1_ × d_2_ × d_3_ × 0.52 (1)

where d_1_, d_2_ and d_3_ are the three mutually perpendicular dimensions of the tumor.

TGI was calculated according to Formula (2):

TGI = [(V_K_ − Vo)/V_K_] × 100% (2)

where V_k_ and V_o_ are average tumor volumes in the experimental and control groups, respectively.

## 4. Conclusions

In this work, we synthesized four new PSMA ligands (**6a**–**d**), suitable for the creation of various bimodal conjugates targeting PCa cells. The introduction of a lysine residue into the structure of the linker peptide fragment did not lead to a significant decrease in the affinity to the protein target. We proposed a new bimodal conjugate preparation technique and used it to synthesize a series of bimodal conjugates based on ligands **6a**–**d** with Docetaxel/Abiraterone drug pairs. *In vitro* cytotoxicity studies of these compounds showed the selective effect of these drugs on PCa cells with a range of PSMA expression levels. However, the cytotoxicity values were rather moderate. Subsequently, we performed the synthesis of four bimodal conjugates with the Abiraterone/MMAE drug pair and one conjugate with the Enzalutamide/MMAE drug pair. A further optimization of the synthetic route was carried out to obtain these five compounds. Subsequent *in vitro* studies showed that the obtained compounds show toxicity comparable to that of the Monomethyl Auristatin E conjugate previously described in the literature. Further *in vivo* studies in xenograft models of PCa demonstrated that the two bimodal conjugates **13a** and **15** had efficacies comparable to the previously described conjugate with MMAE and were superior to the free MMAE. By using PSMA expressing and PSMA non-expressing models, we confirmed a further significant increase in the efficacy of such compounds on PSMA-expressing PCa cells.

## Figures and Tables

**Table 1:** IC_50_ values from *in vitro* studies of inhibition of the NAAG cleavage reaction.

Compound	Substitute			IC_50_ ± SD, nM
	R^1^	R^2^	R^3^	
**6a**	H	Cl	OH	13 ± 3
**6b**	Br	H	OH	11 ± 2
**6c**	COOH	H	OH	10 ± 3
**6d**	COOH	H	H	5.5 ± 1.7
Reference ligands				
ZJ-43				11 ± 3
DCL				547 ± 389
**A** [23]	H	Cl	OH	9 ± 3
**B** [23]	Br	H	OH	18 ± 6
**C** [24]	COOH	H	OH	19 ± 5
**D** [24]	COOH	H	H	6.6 ± 2.4

**Table 2:** CC_50_ values obtained from *in vitro* cytotoxicity studies of synthesized compounds on LNCaP, 22Rv1 and PC-3 cell lines.

Compound	CC_50_, nM		
	LNCaP	22Rv1	PC-3
**12a**	157.4 ± 2.0	267.9 ± 3.8	>800
**12b**	182.6 ± 2.2	247.8 ± 3.3	>800
**12c**	177.8 ± 2.2	453.7 ± 9.8	>800
**12d**	197.8 ± 2.7	463.3 ± 8.0	>800
**13a**	172.6 ± 2.9	155.8 ± 2.4	>800
**13b**	129.7 ± 1.7	128.5 ± 2.0	>800
**13c**	159.0 ± 2.9	192.2 ± 2.7	>800
**13d**	212.6 ± 3.8	191.8 ± 2.4	>800
**15**	139.4± 19.3	170.2 ± 15.3	>800
**MMAE**	0.69 ± 0.1	0.42 ± 0.2	>800
**I** [33]	77.7 ± 1.3	78.5 ± 0.8	>800
**II** [34]	>800	>800	>800
**Combo (I + II)**	73.3 ± 1.0	93.3 ± 1.1	>800

## Data Availability

The data are available from the authors upon request.

## Supplementary Materials

The following supporting information can be downloaded at: https://www.mdpi.com/article/10.3390/ijms241411327/s1, experimental data [38].
