# Supplementary material for: Synthesis of Prostate-Specific Membrane Antigen-Targeted Bimodal Conjugates of Cytotoxic Agents and Antiandrogens and Their Comparative Assessment with Monoconjugates
Source: Int J Mol Sci. 2023 Jul 11;24(14):11327. doi: 10.3390/ijms241411327 (PMC10380083; doi:10.3390/ijms241411327)
Supplement: Supplementary file 1 [file ijms-24-11327-s001.zip › ijms-2450544-supplementary.pdf]

Synthesis of bimodal conjugates of cytotoxic agents and anti-androgenes, comparative assessment with monoconjugates

Nikolai Y. Zyk [1], Anastasiia S. Garanina [2], Ekaterina A. Plotnikova [3], Anton P. Ber [1], Ekaterina A. Nimenko [1], Natalia S. Dashkova [1], Anastasiia A. Uspenskaia [1], Radik R. Shafikov [1,4], Dmitry A. Skvortsov [1], Stanislav A. Petrov [1], Andrei A. Pankratov [3], Nikolai V. Zyk [1], Alexander G. Majouga [5], Elena K. Beloglazkina [1,*] and Aleksei E. Machulkin [1,*]

[1] Lomonosov Moscow State University, Chemistry Dept., Leninskie Gory, Building 1/3, GSP-1, Moscow, 119991, Russian Federation

[2] National University of Science and Technology MISiS, 9 Leninskiy pr, Moscow, 119049, Russian Federa-tion.

[3] National Medical Research Radiological Centre of the Ministry of Health of the Russian Federation, 2 Botkinskiy proezd, 3, Moscow, 125284, Russian Federation.

[4] Shemyakin and Ovchinnikov Institute of Bioorganic Chemistry Russian Academy of Sciences, GSP-7, Ulitsa Miklukho-Maklaya, 16/10, Moscow 117997, Russian Federation

[5] Dmitry Mendeleev University of Chemical Technology of Russia, Miusskaya sq. 9, Moscow, 125047, Russian Federation

* *Correspondence: beloglazki@mail.ru, alekseymachulkin@rambler.ru*

Supplementary Information

Table of contents

1. **Synthesis** ... 3

1.1. **General information** ... 3

1.2. **PSMA ligands synthesis** ... 4

1.2.1. **Solid-phase synthesis of tripeptide fragments 3a and 3b** ... 4

1.2.2. **Synthesis of compounds 4a-d** ... 5

1.2.3. **Synthesis of *tert*-butyl-protected ligands 5a-d** ... 8

1.2.4. **Synthesis of ligands 6a-d** ... 11

1.3. **Synthesis of bimodal conjugates** ... 14

1.3.1. **Synthesis of modified therapeutic agents 7, 9, 11 and 14** ... 14

1.3.2. **Synthesis of bimodal conjugates with the drug pair Docetaxel/Abiraterone** ... 17

1.3.3. **Synthesis of bimodal conjugates with the MMAE/Abiraterone drug pair** ... 23

1.3.4. **Synthesis of a bimodal conjugate with the MMAE/Enzalutamide drug pair 15** ... 33

2. ***In vitro* evaluation of compounds 13a-d and Combo on PC-3 cell line** ... 34

3. ***In vivo* evaluation of synthesized conjugates** ... 34

3.1. **Characteristics of the test conjugates and control substances** ... 34

3.2. **Immunohistochemical Studies** ... 34

3.2.1. ***In vivo* evaluation of antitumor efficacy** ... 35

3.3. **Tumor Histology** ... 35

3.4. ***In vivo* Efficacy Evaluation** ... 37

4. **Spectral data of the synthesised compounds** ... 39

5. **References** ... 103

1. Synthesis

1.1. General information

All used solvents were purified according to procedures described in [1]. All starting compounds were commercially available reagents or were synthesized according to previously published papers [2, 3].

Characterization

^{1}H and ^{13}C NMR spectra were registered on Bruker Avance 400 spectrometer (400 MHz for ^{1}H and 101 MHz for ^{13}C) in $CDCl_3$ or DMSO-d6. Preparative column chromatography was performed on INTERCHIM puriFlash 430. For purification and analysis of samples we used Shimadzu Prominence LC-20 system with Phenomenex Luna 3µm C18 100A (150 x 4.6 mm) column in column oven at 40ºC and fraction collector coupled to single quadrupole mass-spectrometer Shimadzu LCMS-2020 with dual DUIS-ESI-APCI ionization source. Mobile phases: A - 0.1% formic acid in water, B - 10 mM ammonium formate in water, D - acetonitrile. LC parameters for analyses were: gradient flow of 1 ml/min (0-0.5 min - 5% D, 0.5 -10.5 min - 5% to 100% D, 10.5-12 min - 100% D, 12-14.5 min - 100% to 5% D) with optional UV detection for some compounds. MS parameters: drying gas 15.0 L/min, nebulizing gas 1.5 L/min, desolvation line temperature 250ºC, heat block temperature 400ºC, interface voltage -3.5 kV, corona needle voltage -3.5 kV. Positive (mass range 250-2000 Da, in some cases 155-2000 Da) and negative ions (mass range 100-2000 Da) were registered simultaneously. For purification we used identical LC parameters except gradient which was tailored for each compound (in some cases we used mobile phase B instead of A). Fractionation was based on UV detection only; fractions were collected based on UV signal level and slope. High resolution mass spectra were registered on Orbitrap Elite mass spectrometer (Thermo Scientific) with ESI ionization source. Compounds with concentration of 0.1-10 µg/ml (in 1% formic acid in acetonitrile) were directly infused into the ion source with syringe pump (5 µl/min). We didn't use auxiliary and sheath gases, spray voltage was +-3.5 kV, capillary temperature was set to 275ºC. MS spectra were registered by Orbitrap analyzer with 480000 resolution (1 microscan, AGC target value of 1×106, max inject time 1000 ms, averaged on 10 spectra, MS range 100-2000 Da, in some cases 200-4000 Da). We used DMSO and di-iso-octyl phthalate as internal calibration signals (m/z 157.03515 and 413.26623) in positive mode and dodecylsulfate (m/z 265.14790) in negative mode. HPLC MS study of conjugate 13a was performed using an ONYX MONOLITHIC, C18 50x3mm column; 1.8ml/min; Columns Reg Valve. Gradient: From 100% Phase A to 100% Phase B in 2 min followed by 100% Phase B in 0.6 min. Phase A: 0.1% TFA, 2.5% acetonitrile in water. Phase B: 0.1% TFA in acetonitrile. The optical rotation angles were measured on an automatic A.KRUSS Optronic P8000 high-speed polarimeter.

1.2. PSMA ligands synthesis

The synthesis of the **2a-c** vector fragments was carried out from *tert*-butyl-protected urea DCL (**1**) according to procedures presented in the literature.[2, 3]

1.2.1. Solid-phase synthesis of tripeptide fragments 3a and 3b

General methodology for the preparation of resin-immobilized tripeptide fragments

1) Activation of 2-chlorotrityl chloride resin (2-CTC): resin (1 equiv., capacity 1.0-1.5 mmol/g) was stirred in DCM (10 ml per 1 g resin) for 10 min. Then $SOCl_2$ (3 eq.) was added dropwise, followed by DMF (5% v/v, relative to $SOCl_2$). The resulting mixture was stirred at 40 °C for 4 h. Next, the resin was filtered off, transferred to a polypropylene reactor and washed with DMF (3 times for 1 min, 10 ml per 1 g resin) and DCM (3 times for 1 min, 10 ml per 1 g resin).

2) Immobilization of Fmoc-Lys-(*L*)-(NHBoc)-OH on 2-CTC resin: Fmoc-Lys-(*L*)-(NHBoc)-OH (2 eq.) and diisopropylethylamine (DIPEA, 10 eq.) were added to a mixture of activated 2-CTC resin (1 eq., capacity 1.0-1.5 mmol/g) in DMF (10 ml per 1 g resin). The resulting mixture was stirred for 2 h, after which the resin was filtered and successively washed with methanol (3 times for 5 min, 10 ml per 1 g resin), DCM (3 times for 1 min, 10 ml per 1 g resin), DMF (3 times for 1 min, 10 ml per 1 g resin) and DCM (3 times for 1 min, 10 ml per1 g resin).

3) General methodology for removing the Fmoc-protective group: Fmoc-protected amino acid immobilized on 2-CTC resin was washed with DMF (2 times for 1 min, 10 ml per 1 g resin), then a solution of 4-methylpiperidine in DMF (20% v/v, 10 ml per 1 g resin) was added and stirred for 15 min. The resin was filtered, washed with DMF (3 times for 1 min, 10 ml per 1 g of resin), a solution of 4-methylpiperidine in DMF (20% v/v, 10 ml per 1 g of resin) was added and stirred for 15 min. Then the resin was filtered off, washed with DMF (3 times for 1 min, 10 ml per 1 g of resin) and dichloromethane (3 times for 1 min, 10 ml per 1 g of resin).

4) General procedure for the acylation reaction with HBTU and HOBt: to the amino acid immobilized on the resin (1 eq.) was added DMF (10 ml per 1 g resin). Fmoc-protected amino acid (2 eq.), HOBt (0.5 eq.), HBTU (2 eq.) and DIPEA (3 eq.) were added to the resulting mixture and the mixture was stirred for 2 h. The resin was then filtered off and washed with DMF (3 times for 1 min, 10 ml per 1 g resin) and dichloromethane (3 times for 1 min, 10 ml per 1 g resin).

Synthesis of *N*^{2}-((*S*)-2-((*S*)-2-amino-3-phenylpropanamido)-3-(4-(*tert*-butoxy)phenyl)propanoyl)-*N*^{6}-(*tert*-butoxycarbonyl)-*L*-lysine on 2-CTC resin (3a)

From 1000 mg of resin, successive steps 1) 20 ml DCM, 276 μl $SOCl_2$, 24 μl DMF; 2) 1378 mg Fmoc-Lys(Boc)-OH, 2617 μl DIPEA; 3) 8 ml 20% 4- methylpiperidine in DMF; 4) 1351 mg Fmoc-Tyr(o^{t}Bu)-OH, 768 μl DIPEA, 1115 mg HBTU, 99 mg HOBt, 3) 8 ml 20% 4-methylpiperidine in DMF, 4) 1139 mg Fmoc-Phe-OH, 768 μl DIPEA, 1115 mg HBTU, 99 mg

HOBt, 3) 9 ml 20% 4- methylpiperidine in DMF; 2050 mg of resin with immobilized tripeptide **3a** were obtained.

Synthesis of *N*²-*L*-phenylalanyl-*L*-phenylalanyl-*N*⁶-(*tert*-butoxycarbonyl)-*L*-lysine on 2-CTC resin (3b)

From 713 мг mg of resin, successive steps 1) 15 ml DCM, 197 μl $SOCl_2$, 17 μl DMF; 2) 935 mg Fmoc-Lys(Boc)-OH, 1739 μl DIPEA; 3) 6 ml 20% 4- methylpiperidine in DMF; 4) 772 mg Fmoc-Phe-OH, 522 μl DIPEA, 750 mg HBTU, 68 mg HOBt, 3) 7 ml 20% 4-methylpiperidine in DMF, 4)772 mg Fmoc-Phe-OH, 522 μl DIPEA, 750 mg HBTU, 68 mg HOBt, 3) 7 ml 20% 4-methylpiperidine in DMF; 968 mg of resin with immobilized tripeptide **3b** were obtained.

1.2.2. Synthesis of compounds 4a-d

General procedure for obtaining compounds 4a-d

Vector fragment **2a-c** (1.25 eq.) was added to a mixture of tripeptide immobilized on 2-CTC resin (3a or 3b, 1 eq.) with DMF (10 ml per 1 g resin). HBTU (2 eq.), HOBt (0.5 eq.) and DIPEA (3 eq.) were added to the resulting mixture and stirred for 16 h. The resin was then filtered and washed with DMF (4 times for 1 min, 10 ml per 1 g of resin) and dichloromethane (4 times for 1 min, 10 ml per 1 g of resin). Then, 0.75% v/v solution of trifluoroacetic acid in dichloromethane (10 ml per 1 g of resin) was added to the resin, stirred for 15 min, then filtered and the resin washed with DCM (3 times for 1 min, 10 ml per 1 g of resin). From the obtained filtrate the solvent was removed under reduced pressure. The product was further isolated from the mixture by reverse phase column chromatography using a mixture of 0.1% v/v trifluoroacetic acid in water and acetonitrile as eluent.

Synthesis of *N*²-((*S*)-2-((*S*)-2-((7*S*,11*S*)-7,11-bis(*tert*-butoxycarbonyl)-16-(3-chlorobenzyl)-2,2-dimethyl-4,9,17,24-tetraoxo-3-oxa-8,10,16,23-tetraazaheptacosan-27-amido)-3-phenylpropanamido)-3-(4-(*tert*-butoxy)phenyl)propanoyl)-*N*⁶-(*tert*-butoxycarbonyl)-*L*-lysine (4a)

From 758 mg of compound **2a** (0.918 mmol), 1.025 g resin with immobilised tripeptide **3a**, 557 mg HBTU, 50 mg HOBt and 384 μl DIPEA from the resin was removed and further isolated individually by reverse phase column chromatography (InterchimPuriflash C18 120 g, 15μ, gradient from 30% acetonitrile to 60% acetonitrile in 11 min, then from 60% acetonitrile to 100% in 15 min, flow rate 40 ml/min) 937 mg (89% yield) of compound **4a** as a yellow oily solid.

¹H NMR (400 MHz, DMSO-d6) δ, ppm: 8,15 (m, 2H, NH), 7,87 (m, 2H, NH), 7,24-7,33 (m, 2H, Ar), 7,20 (m, 3H, Ar), 7,07-7,18 (m, 6H, Ar), 6,84 (d, J=7,95 Hz, 2H, Ar), 6,76 (m, 1H, NH), 6,19-6,34 (m, 2H, NH), 4,43-4,58 (m, 3H, CH_2+CH), 4,31 (m, 1H, CH), 4,11 (m, 1H, CH), 3,88-4,06 (m, 3H, CH), 3,12-3,19 (m, 2H, CH_2), 2,84-3,09 (m, 9H, CH_2), 2,54-2,63 (m, 1H, CH_2),

2,30-2,38 (m, 2H, CH_2), 2,08-2,30 (m, 7H, CH_2), 1,78-1,91 (m, 1H, CH_2), 1,66 (m, 2H, CH_2), 1,40-1,62 (m, 8H, CH_2), 1,30-1,40 (m, 42H, CH_3+CH_2), 1,12-1,30 (m, 18H, CH_3+CH_2).

Synthesis of *N*²-((*S*)-2-((*S*)-2-((7*S*,11*S*)-16-(4-bromobenzyl)-7,11-bis(*tert*-butoxycarbonyl)-2,2-dimethyl-4,9,17,24-tetraoxo-3-oxa-8,10,16,23-tetraazaheptacosan-27-amido)-3-phenylpropanamido)-3-(4-(*tert*-butoxy)phenyl)propanoyl)-*N*⁶-(*tert*-butoxycarbonyl)-*L*-lysine (4b)

Based on 413 mg of compound **2b** (0.475 mmol), 576 mg of resin with immobilized tripeptide **3a**, 288 mg HBTU, 26 mg HOBt and 195 μl DIPEA from the resin was removed and further isolated individually by reverse phase column chromatography (InterchimPuriflash C18 120 g, 15μ, gradient from 30% acetonitrile to 60% acetonitrile in 11 min, then from 60% acetonitrile to 100% in 15 min, flow rate 40 ml/min) 223 mg (40% yield) of compound **4b** as a yellow oily solid.

^{1}H NMR (400 MHz, DMSO-d6) δ, ppm: 12.51 (br. s, 1H, COOH), 8.15 (d, J = 7,4 Hz, 2H, NH), 7.79-7.94 (m, 2H, NH), 7.44-7.56 (m, 2H, Ar), 7.08-7.24 (m, 9H, Ar), 6.84 (d, J = 7.89 Hz, 2H, Ar), 6.78 (t, J = 5.26 Hz, 1H, NH), 6.21-6.32 (m, 2H, NH), 4.41-4.53 (m, 2H, CH_2+CH), 4.29-4.37 (m, 1H, CH), 4.12 (m, 1H, CH), 4,02 (m, 1H, CH), 3.94 (m, 1H, CH), 3.15 (m, 2H, CH_2), 2.91-3.08 (m, 4H, CH_2), 2.77-2.90 (m, 4H, CH_2), 2.55-2.66 (m, 1H, CH_2), 2.11-2.36 (m, 8H, CH_2), 1.82-1.90 (m, 1H, CH_2), 1.66 (m, 2H, CH_2), 1.53-1.63 (m, 2H, CH_2), 1.41-1.53 (m, 4H, CH_2), 1.31-1.40 (m, 42H, CH_2+ CH_3), 1.13-1.31 (m, 18H, CH_2+ CH_3).

^{13}C NMR (101 MHz, DMSO-d6) δ, ppm: 173.32, 172.24, 172.20, 172.07, 172.00, 171.92, 171.45, 171.38, 171.04, 157.13, 155.59, 153.39, 138.13, 138.00, 137.51, 135.54, 132.64, 131.56, 131.24, 129.72, 129.66, 129.03, 128.66, 128.04, 126.20,, 123.44, 120.15, 119.93, 115.04, 80.59, 80.42, 80.33, 79.78, 77.60, 77.38, 54.52, 53.81, 52.97, 52.84, 52.18, 51.98, 49.56, 46.92, 46.65, 45.11, 38.59, 37.02, 36.44, 32.34, 31.97, 31.80, 30.91, 30.77, 30.64, 29.18, 29.07, 28.57, 28.29, 27.75, 27.66, 27.64, 26.70, 26.32, 26.23, 24.73, 24.60, 22.71, 22.54, 22.45, 22.26.

Synthesis of *N*²-((*S*)-2-((*S*)-2-((7*S*,11*S*)-7,11-bis(*tert*-butoxycarbonyl)-16-(4-(*tert*-butoxycarbonyl)benzyl)-2,2-dimethyl-4,9,17,24-tetraoxo-3-oxa-8,10,16,23-tetraazaheptacosan-27-amido)-3-phenylpropanamido)-3-(4-(*tert*-butoxy)phenyl)propanoyl)-*N*⁶-(*tert*-butoxycarbonyl)-*L*-lysine (4c)

From 496 mg of compound **2c** (0.557 mmol), 621 mg of resin with immobilized tripeptide **3a**, 338 mg HBTU, 30 mg HOBt and 233 μl DIPEA from resin was removed and further isolated individually by reverse phase column chromatography (InterchimPuriflash C18 120 g, 15μ, gradient from 30% acetonitrile to 60% acetonitrile in 11 min, then from 60% acetonitrile to 100% in 15 min, flow rate 40 ml/min) 523 mg (76% yield) of compound **4c** as a yellow oily solid.

^{1}**H NMR** (400 MHz, DMSO-d6) δ, ppm: 12.54 (br. s, 1H, COOH), 8.15 (d, J=6.17 Hz, 2H, NH), 7.80-7.90 (m, 4H, Ar+NH), 7.27 (d, J=8.19 Hz, 2H, Ar), 7.11-7.24 (m, 7H, Ar), 6.84 (d, J=8.4 Hz, 2H, Ar), 6.77 (m, 1H, NH), 6.21-6.31 (m, 2H, NH), 4.60 (m, 1H, CH), 4.44-4.56 (m, 2H, CH_2), 4.33 (m, 1H, CH), 4.12 (m, 1H, CH), 3.89-4.05 (m, 2H, CH), 3.11-3.23 (m, 2H, CH_2), 2.91-3.08 (m, 4H, CH_2), 2.75-2.84 (m, 1H, CH_2), 2.54-2.64 (m, 1H, CH_2), 2.29-2.37 (m, 2H, CH_2), 2.22-2.29 (m, 2H, CH_2), 2.12-2.22 (m, 4H, CH_2), 1.82-1.90 (m, 1H, CH_2), 1.60-1.73 (m, 2H, CH_2), 1.40-1.60 (m, 13H, CH_2+CH_3), 1.39-1.48 (m, 4H, CH_2), 1.35 (m, 41H, CH_2+CH_3), 1.12-1.31 (m, 18H, CH_2+CH_3).

^{13}**C NMR** (101 MHz, DMSO-d6) δ, ppm: 173,32, 172,19, 172,12, 172,05, 171,92, 171,45, 171,39, 171,04, 164,81, 164,73, 157,13, 155,59, 153,38, 145,42, 143,77, 143,31, 138,13, 135,53, 132,79, 132,65, 130,25, 129,98, 129,65, 129,47, 129,17, 129,03, 128,03, 127,45, 126,51, 126,20, 123,44, 115,04, 87,97, 85,65, 80,68, 80,57, 80,41, 80,32, 79,78, 77,59, 77,37, 54,53, 53,81, 52,96, 52,81, 52,18, 51,97, 50,05, 47,41, 46,78, 45,30, 40,90, 38,60, 38,54, 37,01, 36,42, 35,83, 32,37, 31,99, 31,79, 30,91, 30,77, 30,64, 29,18, 29,07, 28,56, 28,28, 27,81, 27,75, 27,65, 27,62, 26,71, 26,32, 26,24, 24,74, 24,59, 22,71, 22,46, 22,24.

Synthesis of *N*^{2}-((7*S*,11*S*)-7,11-bis(*tert*-butoxycarbonyl)-16-(4-(*tert*-butoxycarbonyl)benzyl)-2,2-dimethyl-4,9,17,24-tetraoxo-3-oxa-8,10,16,23-tetraazaheptacosan-27-oyl)-*L*-phenylalanyl-*L*-phenylalanyl-*N*^{6}-(*tert*-butoxycarbonyl)-*L*-lysine (4d)

From 379 mg of compound **2c** (0.425 mmol), 572 mg of resin with immobilized tripeptide 3b, 258 mg HBTU, 23 mg HOBt and 178 µl DIPEA from resin was removed and further isolated individually by reverse phase column chromatography (InterchimPuriflash C18 120 g, 15µ, gradient from 30% acetonitrile to 60% acetonitrile in 11 min, then from 60% acetonitrile to 100% in 15 min, flow rate 40 ml/min) 457 mg (95% yield) of compound **2d** as a yellow oily solid.

^{1}**H NMR** (400 MHz, DMSO-d6) δ, ppm: 12,56 (br. s, 1H, COOH), 8,15 (d, J = 7,89 Hz, 2H, NH), 7,90-8,01 (m, 1H, NH), 7,79-7,89 (m, 3H, Ar+NH), 7,11-7,30 (m, 12H, Ar), 6,78 (m, 1H, NH), 6,21-6,31 (m, 2H, NH), 4,47-4,63 (m, 3H, CH+CH_2), 4,35 (m, 1H, CH), 4,13 (m, 1H, CH), 3,89-4,05 (m, 2H, CH), 3,04-3,23 (m, 3H, CH_2), 2,82-3,04 (m, 6H, CH_2), 2,56-2,67 (m, 1H, CH_2), 2,31-2,37 (m, 1H, CH_2), 2,10-2,30 (m, 6H, CH_2), 1,79-1,91 (m, 1H, CH_2), 1,61-1,72 (m, 2H, CH_2), 1,42-1,61 (m, 16H, CH_2+$C(CH_3)_3$), 1,35 (m, 42H, $C(CH_3)_3$+CH_2), 1,10-1,30 (m, 8H, CH_2).

^{13}**C NMR** (101 MHz, DMSO-d6) δ, ppm: 173,34, 172,23, 172,19, 172,13, 172,06, 171,92, 171,45, 171,38, 171,05, 171,00, 164,82, 164,74, 157,13, 155,60, 143,77, 143,31, 138,12, 137,97, 135,53, 130,26, 129,98, 129,47, 129,17, 129,06, 128,09, 128,02, 127,45, 126,51, 126,25, 126,19, 115,04, 80,69, 80,57, 80,41, 80,33, 79,78, 77,38, 54,39, 53,77, 52,97, 52,82, 52,19, 51,97, 50,07,

47,42, 46,80 45,30, 38,60, 38,53, 37,06, 36,95, 31,99, 31,78, 30,91, 30,76, 30,63, 29,18, 29,07, 28,29, 27,81, 27,75, 27,65, 27,63, 26,71, 26,32, 26,24, 24,75, 24,59, 22,54, 22,24, 22,73.

1.2.3. Synthesis of *tert*-butyl-protected ligands 5a-d

General procedure for the acylation reaction of 3-azidopropylamine.

Compound **4a-d** (1 eq.) was dissolved in DMF. The mixture was cooled down to 0 °C and 3-azidopropylamine (2 eq.), HOBt (1.5 eq.), DIPEA (2 eq.) and HBTU (1.5 eq.) were added successively. The reaction mixture was stirred for 16 hours. The DMF was removed under reduced pressure, the dry residue was dissolved in dichloromethane and washed twice with water, twice with saturated sodium hydrogen carbonate solution and once with saturated sodium chloride solution. Then the organic fraction was dried over anhydrous sodium sulfate and the solvent was removed under reduced pressure. The product was isolated individually by column chromatography, the eluent used was dichloromethane-methanol mixture.

Synthesis of tri-*tert*-butyl (10*S*,13*S*,16*S*,34*S*,38*S*)-10-((3-azidopropyl)carbamoyl)-16-benzyl-13-(4-(*tert*-butoxy)benzyl)-29-(3-chlorobenzyl)-2,2-dimethyl-4,12,15,18,21,28,36-heptaoxo-3-oxa-5,11,14,17,22,29,35,37-octaazatetracontane-34,38,40-tricarboxylate (5a)

From 927 mg (0.653 mmol) of compound **4a**, 131 mg (1.306 mmol) 3-azidopropylamine, 227 µl (1.306 mmol) DIPEA, 132 mg (0.979 mmol) HOBt and 371 mg (0.979 mmol) HBTU in 30 ml DMF, after purification by column chromatography (InterchimPuriflash 120 g, 50µ, gradient from 0% methanol to 5% methanol in 30 min, then from 5% methanol to 10% in 15 min, then from 10% methanol to 100% in 10 min flow rate - 40 ml/min) 796 mg (81% yield) of compound **5a** was obtained as a yellow oily solid.

^{1}H NMR (400 MHz, $CDCl_3$) δ, ppm: 7.97 (t, J=6.11 Hz, 1H, NH), 7.79 (m, 1H, NH), 7.29-7.35 (m, 2H, Ar), 6.87-7.26 (m, 11H, Ar), 6.08-6.23 (m, 1H, NH), 4.41-4.57 (m, 3H, CH_2+CH), 4.21-4.41 (m, 4H, CH), 3.20-3.47 (m, 7H, CH_2), 2.85-3.20 (m, 6H, CH_2), 2.80 (m, 1H, CH_2), 2.53 (m, 1H, CH_2), 2.43 (m, 1H, CH_2), 2.13-2.39 (m, 6H, CH_2), 1.99-2.13 (m, 2H, CH_2), 1.79-1.91 (m, 3H, CH_2), 1.50-1.74 (m, 9H, CH_2), 1.36-1.50 (m, 40H, CH_2+CH_3), 1.19-1.36 (m, 17H, CH_2+CH_3).

^{13}C NMR (101 MHz, DMSO-d6) δ, ppm: 174.83, 172.85, 172.39, 172.11, 172.05, 172.00, 171.86, 171.67, 171.63, 156.58, 156.10, 154.94, 153.57, 139.58, 138.75, 135.18, 134.45, 133.92, 132.78, 129.82, 129.40, 128.99, 128.54, 128.24, 127.25, 126.96, 125.76, 125.51, 123.84, 81.46, 81.33, 81.06, 80.11, 80.04, 78.98, 77.95, 77.91, 76.89, 56.72, 56.64, 56.39, 53.49, 52.91, 52.70, 52.48, 52.38, 50.02, 48.33, 47.46, 46.79, 45.35, 40.29, 39.13, 36.49, 36.17, 34.62, 32.52, 32.22, 31.78, 31.17, 30.84, 30.35, 28.79, 28.43, 28.15, 27.98, 27.95, 27.76, 27.64, 27.59, 27.56, 26.28, 24.39, 23.78, 22.05.

Synthesis of tri-*tert*-butyl (10*S*,13*S*,16*S*,34*S*,38*S*)-10-((3-azidopropyl)carbamoyl)-16-benzyl-29-(4-bromobenzyl)-13-(4-(*tert*-butoxy)benzyl)-2,2-dimethyl-4,12,15,18,21,28,36-heptaoxo-3-oxa-5,11,14,17,22,29,35,37-octaazatetracontane-34,38,40-tricarboxylate (5b)

From 270 mg (0.1844 mmol) of compound **4b**, 37 mg (0.369 mmol) 3-azidopropylamine, 64 µl (0.369 mmol) DIPEA, 37 mg (0.277 mmol) HOBt and 105 mg (0.277 mmol) HBTU in 23 ml DMF, after purification by column chromatography (InterchimPuriflash 25 g, 50µ, gradient from 0% methanol to 5% methanol in 25 min, then from 5% methanol to 10% in 11 min, then from 10% methanol to 100% in 8 min flow rate - 20 ml/min) 218 mg (76% yield) of compound **5b** as a yellow oily solid was obtained.

^{1}H NMR (400 MHz, DMSO-d6) δ, ppm: 8.32 (d, J=7.09 Hz, 1H, NH), 8.16 (d, J=7.83 Hz, 1H, NH), 7.90-7.98 (m, 1H, NH), 7.67 (d, J=8.13 Hz, 1H, NH), 7.60 (m, 1H, NH), 7.53 (d, J=8.31 Hz, 1H, Ar), 7.47 (d, J=8.38 Hz, 1H, Ar), 7.18-7.23 (m, 2H, Ar), 7.09-7.18 (m, 7H, Ar), 6.85 (d, J=8.25 Hz, 2H, Ar), 6.76 (m, 1H, NH), 6.27-6.31 (m, 1H, NH), 6.24 (m, 1H, NH), 4.49-4.43 (m, 2H, CH_2), 4.35-4.41 (m, 1H, CH), 4.28 (m, 1H, CH), 4.10 (m, 1H, CH), 3.98-4.05 (m, 1H, CH), 3.91-3.98 (m, 1H, CH), 3.30 (m, 2H, CH_2), 2.93-3.19 (m, 8H, CH_2), 2.82-2.92 (m, 4H, CH_2), 2.59-2.67 (m, 1H, CH_2), 2.31 (t, J=7.06 Hz, 3H, CH_2), 2.23-2.28 (m, 1H, CH_2), 2.13-2.23 (m, 4H, CH_2), 1.80-1.90 (m, 1H, CH_2), 1.58-1.70 (m, 5H, CH_2), 1.40-1.58 (m, 8H, CH_2), 1.31-1.39 (m, 43H, CH_3+CH_2), 1.13-1.27 (m, 17H, CH_3+CH_2).

^{13}C NMR (101 MHz, DMSO-d6) δ, ppm: 172.77, 172.24, 172.20, 172.04, 171.97, 171.92, 171.70, 171.62, 171.45, 171.29, 170.87, 157.12, 155.59, 153.45, 137.99, 137.89, 137.49, 132.61, 131.56, 131.24, 129.72, 129.58, 128.98, 128.63, 128.08, 126.29, 123.51, 120.15, 119.94, 80.59, 80.41, 80.33, 79.78, 77.64, 77.38, 55.10, 54.70, 52.97, 52.83, 52.17, 49.56, 48.23, 46.93, 46.64, 45.13, 38.69, 36.92, 35.81, 32.33, 31.97, 31.80, 31.60, 30.91, 30.73, 30.58, 29.25, 29.07, 29.00, 28.55, 28.28, 27.75, 27.65, 27.63, 26.70, 26.33, 26.24, 24.72, 24.60, 22.80, 22.45, 22.26.

Synthesis of tri-*tert*-butyl (10*S*,13*S*,16*S*,34*S*,38*S*)-10-((3-azidopropyl)carbamoyl)-16-benzyl-13-(4-(*tert*-butoxy)benzyl)-29-(4-(*tert*-butoxycarbonyl)benzyl)-2,2-dimethyl-4,12,15,18,21,28,36-heptaoxo-3-oxa-5,11,14,17,22,29,35,37-octaazatetracontane-34,38,40-tricarboxylate (5c)

From 329 mg (0.2214 mmol) of compound **4c**, 44 mg (0.443 mmol) 3-azidopropylamine, 77 µl (0.443 mmol) DIPEA, 45 mg (0.332 mmol) HOBt and 126 mg (0.332 mmol) HBTU in 25 ml DMF, after purification by column chromatography (InterchimPuriflash 25 g, 50µ, gradient from 0% methanol to 5% methanol in 25 min, then from 5% methanol to 10% in 11 min, then from 10% methanol to 100% in 8 min flow rate - 20 ml/min) 300 mg (86% yield) of compound **5c** was obtained as a yellow oily solid.

^{1}H NMR (400 MHz, DMSO-d6) δ, ppm: 8.33 (d, J=7.03 Гц, 1H, NH), 8.17 (d, J=7.76 Гц, 1H, NH), 7.91-8.00 (m, 1H, NH), 7.87 (d, J=8.38 Гц, 1H, Ar), 7.82 (d, J=8.31 Гц, 1H, Ar), 7.66 (m, 1H, Ar), 7.59 (m, 1H, Ar), 7.24-7.31 (m, 2H, Ar), 7.18-7.24 (m, 2H, Ar), 7.10-7.18 (m, 5H, Ar), 6.85 (m, 2H, Ar), 6.76 (m, 1H, NH), 6.21-6.30 (m, 2H, NH), 4.59 (m, 1H, CH_2), 4.53 (m, 1H, CH_2), 4.34-4.42 (m, 1H, CH), 4.28 (m, 1H, CH), 4.09 (m, 1H, CH), 3.98-4.05 (m, 1H, CH), 3.89-3.98 (m, 1H, CH), 3.30 (m, 2H, CH_2) 3.12-3.22 (m, 2H, CH_2), 3.05-3.12 (m, 2H, CH_2), 2.93-3.05 (m, 3H, CH_2), 2.81-2.93 (m, 4H, CH_2), 2.59-2.68 (m, 1H, CH_2), 2.28-2.36 (m, 3H, CH_2), 2.12-2.28 (m, 5H, CH_2), 1.79-1.90 (m, 1H, CH_2), 1.58-1.70 (m, 4H, CH_2), 1.52 (m, 10H, CH_2+CH_3), 1.39-1.49 (m, 5H, CH_2), 1.31-1.39 (m, 39H, CH_2+CH_3), 1.24-1.31 (m, 3H, CH_2), 1.12-1.24 (m, 15H, CH_2+CH_3).

^{13}C NMR (101 MHz, DMSO-d6) δ, ppm: 172.79, 172.18, 172.09, 172.02, 171.92, 171.71, 171.63, 171.45, 171.29, 170.87, 164.81, 164.73, 157.12, 155.58, 153.45, 143.76, 143.29, 137.88, 132.61, 130.25, 129.98, 129.57, 129.47, 129.16, 128.98, 128.08, 127.45, 126.48, 126.28, 123.51, 80.68, 80.57, 80.40, 80.32, 79.78, 77.63, 77.37, 55.13, 54.71, 52.87, 52.18, 48.23, 38.69, 36.90, 35.81, 32.35, 31.98, 31.79, 31.59, 30.91, 30.72, 30.58, 29.24, 29.07, 28.54, 28.28, 27.80, 27.75, 27.64, 26.71, 26.34, 24.73, 24.58, 22.80, 22.46, 22.24.

Synthesis of tri-*tert*-butyl (10*S*,13*S*,16*S*,34*S*,38*S*)-10-((3-azidopropyl)carbamoyl)-13,16-dibenzyl-29-(4-(*tert*-butoxycarbonyl)benzyl)-2,2-dimethyl-4,12,15,18,21,28,36-heptaoxo-3-oxa-5,11,14,17,22,29,35,37-octaazatetracontane-34,38,40-tricarboxylate (5d)

From 179 mg (0.127 mmol) of compound **4d**, 25 mg (0.253 mmol) of 3-azidopropylamine, 44 μl (0.253 mmol) of DIPEA, 26 mg (0.19 mmol) of HOBt and 72 mg (0.19 mmol) of HBTU in 15 ml of DMF, after purification by column chromatography (InterchimPuriflash 25 g, 50μ, gradient from 0% methanol to 5% methanol in 25 min, then from 5% methanol to 10% in 11 min, then from 10% methanol to 100% in 8 min flow rate - 20 ml/min) 129 mg (47% yield) of compound **5d** was obtained as a yellow oily solid.

^{1}H NMR (400 MHz, DMSO-d6) δ, ppm: 8.33 (d, J=7.21 Hz, 1H, NH), 8.19 (d, J=7.64 Hz, 1H, NH), 7.90-7.99 (m, 1H, NH), 7.87 (d, J=8.19 Hz, 1H, Ar), 7.82 (d, J=8.19 Hz, 1H, Ar), 7.66-7.75 (m, 1H, NH), 7.55-7.64 (m, 1H, NH), 7.22-7.29 (m, 6H, Ar), 7.11-7.22 (m, 6H, Ar), 6.76 (m, 1H, NH), 6.21-6.32 (m, 2H, NH), 4.59 (m, 1H), 4.53 (m, 1H, CH), 4.38-4.47 (m, 1H, CH_2), 4.30 (m, 1H, CH_2), 4.10 (m, 1H, CH), 3.89-4.05 (m, 2H, CH), 3.16-3.23 (m, 1H, CH_2), 3.11-3.16 (m, 1H, CH_2), 3.03-3.11 (m, 3H, CH_2), 2.91-3.03 (m, 3H, CH_2), 2.82-2.91 (m, 3H, CH_2), 2.27-2.35 (m, 3H, CH_2), 2.19-2.25 (m, 2H, CH_2), 2.12-2.19 (m, 2H, CH_2), 1.77-1.91 (m, 1H, CH_2), 1.58-1.71 (m, 4H, CH_2), 1.52 (m, 11H, CH_2+CH_3), 1.29-1.50 (m, 49H, CH_2+CH_3), 1.25 (m, 2H, CH_2), 1.11-1.23 (m, 6H, CH_2).

^{13}C NMR (101 MHz, DMSO-d6) δ, ppm: 172.74, 172.23, 172.18, 172.11, 172.03, 171.92, 171.75, 171.61, 171.45, 171.30, 170.84, 164.82, 164.73, 157.13, 155.59, 143.76, 143.29, 137.95, 137.92, 130.26, 129.98, 129.47, 129.16, 129.08, 129.00, 128.16, 128.07, 127.45, 126.49, 126.32, 126.27, 80.69, 80.57, 80.41, 80.32, 79.78, 77.38, 55.00, 54.62, 52.96, 52.90, 52.81, 52.18, 50.06, 48.23, 47.41, 46.79, 45.33, 38.68, 36.81, 36.61, 35.82, 32.36, 31.98, 31.78, 31.58, 30.91, 30.70, 30.55, 29.25, 29.07, 28.28, 27.81, 27.75, 27.65, 27.62, 26.72, 26.34, 26.25, 24.74, 24.58, 22.81, 22.45, 22.25.

1.2.4. Synthesis of ligands 6a-d

General procedure for removing protective groups

The protected ligand (5a-d) was dissolved in a mixture of trifluoroacetic acid (50% v/v), dichloromethane (40% v/v), triisopropylsilane (5% v/v) and water (5% v/v) at a ratio of 5 ml mixture per 100 mg ligand. The reaction mixture was stirred for 4 h. Then the solvent was removed under reduced pressure, after which the dry residue was precipitated with diethyl ether, decanted and the precipitate was washed three times with Et_2O. The product was isolated individually by reverse phase column chromatography, a mixture of 0.1% v/v trifluoroacetic acid in water and acetonitrile was used as eluent.

Synthesis of (3*S*,7*S*,25*S*,28*S*,31*S*)-31-(4-aminobutyl)-36-azido-25-benzyl-12-(3-chlorobenzyl)-28-(4-hydroxybenzyl)-5,13,20,23,26,29,32-heptaoxo-4,6,12,19,24,27,30,33-octaazahexatriacontane-1,3,7-tricarboxylic acid (6a)

From 795 mg (0.529 mmol) of compound **5a** and 15 ml mixture of trifluoroacetic acid, triisopropylsilane, water and dichloromethane, 517 mg (76% yield) of compound **6a** was isolated individually by reverse phase column chromatography (InterchimPuriflashC18 120 g, 15μ, gradient from 10% acetonitrile to 100% acetonitrile in 50 min, flow rate 40 ml/min) as a white amorphous powder.

^{1}H NMR (400 MHz, DMSO-d6) δ, ppm: 8.30-8.36 (m, 1H, NH), 8.12 (d, J=7.52 Hz, 1H, NH), 7.91-8.00 (m, 1H, NH), 7.74 (m, 1H, NH), 7.49-7.57 (m, 1H, NH), 7.24-7.39 (m, 2H, Ar), 7.07-7.24 (m, 7H, Ar), 7.03 (m, 2H, Ar), 6.65 (d, J=8.38 Hz, 2H, Ar), 6.28-6.38 (m, 2H, NH), 4.38-4.59 (m, 2H, CH_2), 4.22-4.35 (m, 2H, CH), 3.96-4.13 (m, 3H, CH), 3.25-3.33 (m, 2H, CH_2), 3.11-3.22 (m, 2H,), 3.07 (m, 2H, CH_2), 2.86-3.04 (m, 4H, CH_2), 2.76-2.86 (m, 1H, CH_2), 2.59-2.76 (m, 3H, CH_2), 2.11-2.39 (m, 8H, CH_2), 1.84-1.95 (m, 1H, CH_2), 1.55-1.75 (m, 5H, CH_2), 1.32-1.55 (m, 10H, CH_2), 1.07-1.32 (m, 7H, CH_2).

^{13}C NMR (101 MHz, DMSO-d6) δ, ppm: 174.87, 174.58, 174.15, 173.07, 172.56, 171.95, 171.62, 171.46, 157.70, 156.28, 141.58, 138.89, 138.32, 133.44, 130.98, 130.62, 130.42, 129.40, 128.47, 128.22, 127.57, 127.24, 126.67, 126.45, 125.34, 115.38, 58.52, 55.38, 52.99, 52.55, 52.06,

48.63, 47.29, 45.72, 39.07, 37.41, 37.28, 36.25, 32.16, 31.57, 31.16, 30.98, 30.31, 29.47, 29.37, 28.64, 27.95, 27.01, 26.67, 25.10, 22.68.

$[\alpha]_{20}^{D}$ = -11.0°

HPLC-MS: target compound content – 99.9%, t_R=11.05 min.

ESI-HRMS: for $C_{56}H_{77}ClN_{12}O_{14}$: m/z calculated for $[M+H]^+$ 1177.5371, found: 1177.5443; m/z calculated for $[M+Na]^+$ 1199.5269, found: 1199.5263.

Synthesis of (3*S*,7*S*,25*S*,28*S*,31*S*)-31-(4-aminobutyl)-36-azido-25-benzyl-12-(4-bromobenzyl)-28-(4-hydroxybenzyl)-5,13,20,23,26,29,32-heptaoxo-4,6,12,19,24,27,30,33-octaazahexatriacontane-1,3,7-tricarboxylic acid (6b)

From 199 mg (128.7 mmol) of compound **5b** and 7 mL of a mixture of trifluoroacetic acid, triisopropylsilane, water and dichloromethane 126 mg (80% yield) of compound **6b** was isolated individually by reverse phase column chromatography (InterchimPuriflash C18 40 g, 15μ, gradient from 10% acetonitrile to 20% acetonitrile in 2 min, from 20% acetonitrile to 100% in 35 min, flow rate 20 ml/min) as a white amorphous powder.

^{1}H NMR (400 MHz, DMSO-d6) δ, ppm: 12.45 (br. s, 3H, COOH), 9.22 (br. s, 1H, OH), 8.29 (d, J=7.34 Hz, 1H, NH), 8.10 (d, J=8.13 Hz, 1H, NH), 7.88-7.96 (m, 1H, NH), 7.75-7.82 (m, 1H, NH), 7.65 (br, s, 3H, NH_3^+), 7.55-7.59 (m, 1H, NH), 7.54 (d, J=8.38 Hz, 1H, Ar), 7.48 (d, J=8.31 Hz, 1H, Ar), 7.19-7.25 (m, 2H, Ar), 7.09-7.19 (m, 5H, Ar), 7.03 (m, 2H, Ar), 6.65 (d, J=8.38 Hz, 2H, Ar), 6.26-6.37 (m, 2H, NH), 4.49-4.43 (m, 2H, CH_2), 4.28-4.38 (m, 2H, CH), 4.08 (m, 3H, CH), 3.27-3.32 (m, 2H, CH_2), 3.12-3.21 (m, 2H, CH_2), 3.05-3.12 (m, 2H, CH_2), 2.88-3.05 (m, 4H, CH_2), 2.77-2.85 (m, 1H, CH_2), 2.73 (m, 2H, CH_2), 2.61-2.69 (m, 1H, CH_2), 2.26-2.35 (m, 3H, CH_2), 2.14-2.26 (m, 5H, CH_2), 1.85-1.96 (m, 1H, CH_2), 1.65-1.75 (m, 2H, CH_2), 1.57-1.65 (m, 3H, CH_2), 1.45-1.55 (m, 6H, CH_2), 1.34-1.44 (m, 3H, CH_2), 1.31 (m, 1H, CH_2), 1.12-1.29 (m, 6H, CH_2).

^{13}C NMR (101 MHz, DMSO-d6) δ, ppm: 174.55, 174.26, 173.82, 172.71, 172.07, 171.77, 171.59, 171.24, 171.08, 157.32, 155.91, 138.02, 137.94, 131.55, 131.24, 130.03, 129.72, 129.02, 128.64, 128.09, 127.84, 126.29, 120.14, 119.92, 114.99, 55.00, 52.61, 52.17, 51.73, 51.24, 48.23, 46.74 38.66, 36.87, 35.85, 31.90, 31.17, 30.77, 30.58, 29.97, 29.07, 28.26, 27.59, 26.65, 26.29, 24.72, 24.59, 22.31.

$[\alpha]_{20}^{D}$ = -9,0°

HPLC-MS: target compound content – 99.9%, t_R=10.8 min.

ESI-HRMS: for $C_{56}H_{77}BrN_{12}O_{14}$: m/z calculated for $[M+H]^+$ 1221.49383, found: 1221.4937.

Synthesis of (3*S*,7*S*,25*S*,28*S*,31*S*)-31-(4-aminobutyl)-36-azido-25-benzyl-12-(4-carboxybenzyl)-28-(4-hydroxybenzyl)-5,13,20,23,26,29,32-heptaoxo-4,6,12,19,24,27,30,33-octaazahexatriacontane-1,3,7-tricarboxylic acid (6c)

From 282 mg (179.9 mmol) of compound **5c** and 11 ml mixture of trifluoroacetic acid, triisopropylsilane, water and dichloromethane, 126 mg (80% yield) of compound **6c** was isolated individually by reverse phase column chromatography (InterchimPuriflash C18 120 g, 15μ, gradient from 10% acetonitrile to 100% acetonitrile in 50 min, flow rate 40 ml/min) as a white amorphous powder.

^{1}H NMR (400 MHz, DMSO-d6) δ, ppm: 12.56 (br. s, 3H, COOH), 9.22 (br. s, 1H, OH), 8.29 (d, J=7.40 Hz, 1H, NH), 8.10 (d, J=7.70 Hz, 1H, NH), 7.89-7.97 (m, 2H, NH+Ar), 7.87 (d, J=8.25 Hz, 1H, Ar), 7.74-7.82 (m, 1H, NH), 7.65 (m, 3H, NH), 7.53-7.60 (m, 1H, NH), 7.25-7.32 (m, 2H, Ar), 7.18-7.25 (m, 2H, Ar), 7.12-7.18 (m, 3H, Ar), 7.03 (m, 2H, Ar), 6.60-6.69 (m, 2H, Ar), 6.25-6.38 (m, 2H, NH), 4.60-4.53 (m, 2H, CH_2), 4.26-4.38 (m, 2H, CH), 3.98-4.16 (m, 3H, CH), 3.32 (t, J=6.85 Hz, 2H, CH_2), 3.17 (m, 2H, CH_2), 3.05-3.13 (m, 2H, CH_2), 2.88-3.05 (m, 4H, CH_2), 2.69-2.86 (m, 3H, CH_2), 2.60-2.69 (m, 1H, CH_2), 2.30-2.39 (m, 2H, CH_2), 2.24-2.30 (m, 2H, CH_2), 2.20 (m, 4H, CH_2), 1.84-1.96 (m, 1H, CH_2), 1.56-1.74 (m, 5H, CH_2), 1.37-1.55 (m, 9H, CH_2), 1.32 (m, 1H, CH_2), 1.14-1.29 (m, 6H, CH_2).

^{13}C NMR (101 MHz, DMSO-d6) δ, ppm: 174.50, 174.21, 173.78, 172.62, 171.73, 171.55, 171.23, 171.07, 167.21, 157.30, 155.89, 137.94, 130.04, 129.78, 129.47, 129.02, 128.09, 127.83, 127.38, 126.29, 114.98, 54.97, 52.56, 52.13, 51.67, 48.23, 38.68, 36.88, 35.85, 31.78, 29.91, 28.26, 27.52, 26.62, 24.71, 22.27.

$[\alpha]_{20}^{D}$ = -9.7°

HPLC-MS: target compound content – 99.0%, t_R=10.25 min.

ESI-HRMS: for $C_{56}H_{78}N_{12}O_{16}$: m/z calculated for $[M+H]^+$ 1187.57315, found: 1187.572.

Synthesis of (3*S*,7*S*,25*S*,28*S*,31*S*)-31-(4-aminobutyl)-36-azido-25,28-dibenzyl-12-(4-carboxybenzyl)-5,13,20,23,26,29,32-heptaoxo-4,6,12,19,24,27,30,33-octaazahexatriacontane-1,3,7-tricarboxylic acid (6d)

From 119 mg (0.0796 mmol) of compound **5d** and 6 ml of a mixture of trifluoroacetic acid, triisopropylsilane, water and dichloromethane 71 mg (76% yield) of compound **6d** was isolated individually by reverse phase column chromatography (InterchimPuriflash C18 20 g, 15μ, gradient from 10% acetonitrile to 20% acetonitrile in 2 min, from 20% acetonitrile to 100% in 23 min, flow rate 20 ml/min) as a white amorphous powder.

^{1}H NMR (400 MHz, DMSO-d6) δ, ppm: 12.55 (br. s, 3H, COOH), 8.30 (d, J=7.28 Hz, 1H, NH), 8.17 (d, J=7.89 Hz, 1H, NH), 7.89-7.99 (m, 2H, NH+Ar), 7.87 (d, J=8.19 Hz, 1H, Ar), 7.77-7.84 (m, 1H, NH), 7.56-7.68 (m, 3H, NH+Ar), 7.22-7.30 (m, 6H, Ar), 7.20 (m, 2H, Ar), 7.15 (m,

3H, Ar), 6.26-6.36 (m, 2H, NH), 4.60-4.53 (m, 2H, CH_2), 4.39-4.48 (m, 1H, CH), 4.31 (m, 1H, CH), 3.98-4.17 (m, 3H, CH), 3.32 (t, J=6.76 Hz, 2H, CH_2), 3.12-3.25 (m, 2H, CH_2), 3.07-3.12 (m, 2H, CH_2), 2.97-3.07 (m, 3H, CH_2), 2.87-2.97 (m, 3H, CH_2), 2.73 (m, 2H, CH_2), 2.59-2.69 (m, 1H, CH_2), 2.30-2.38 (m, 2H, CH_2), 2.13-2.30 (m, 6H, CH_2), 1.85-1.96 (m, 1H, CH_2), 1.64 (m, 5H, CH_2), 1.50 (m, 6H, CH_2), 1.41 (m, 3H, CH_2), 1.12-1.34 (m, 7H, CH_2).

$[\alpha]_{20}^{D}$ = -7.9°

HPLC-MS: target compound content – 99.9%, t_R=10.7 min.

ESI-HRMS: for $C_{56}H_{78}N_{12}O_{15}$: m/z calculated for $[M+H]^+$ 1171.57824, found: 1171.5782.

1.3. Synthesis of bimodal conjugates

1.3.1. Synthesis of modified therapeutic agents 7, 9, 11 and 14

The preparation of modified docetaxel (**9**) and MMAE (**11**) was carried out according to previously described procedures.[4, 5]

The synthesis of modified abiraterone **7** was carried out according to the following synthetic scheme.

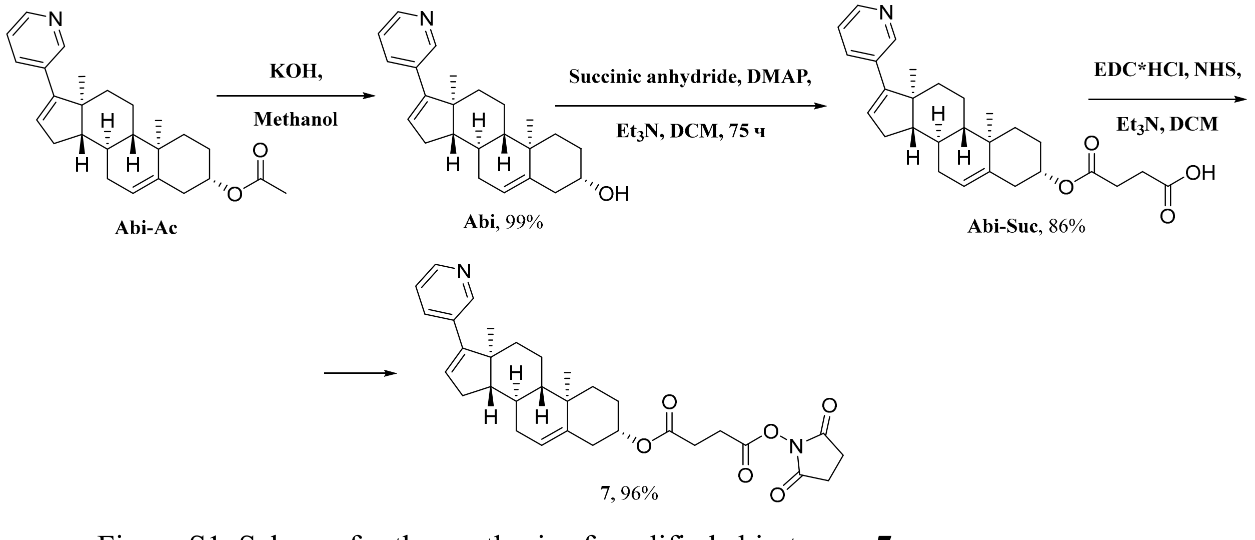

Figure S1. Scheme for the synthesis of modified abiraterone **7**

Abiraterone acetate (**Abi-Ac**) was used as the starting compound. In the first step an alkaline hydrolysis was performed to give the product **Abi**. At the second stage the acylation of the free hydroxyl group of Abiraterone with succinic anhydride was performed. In the last step, the obtained **Abi-Suc** was reacted with N-hydroxysuccinimide to give activated NHS ester **7**.

Synthesis of (3*S*,8*R*,9*S*,10*R*,13*S*,14*S*)-10,13-dimethyl-17-(pyridin-3-yl)-2,3,4,7,8,9,10,11,12,13,14,15-dodecahydro-1*H*-cyclopenta[*a*]phenanthren-3-ol (Abi)

1000 mg (2.554 mmol) of abiraterone acetate (**Abi-Ac**) was dissolved in 12.5 mL of methanol, after which 20 mL of 10% potassium hydroxide solution in methanol was added. The reaction mixture was stirred until the reaction was finished (the reaction was monitored by TLC in DCM/methanol = 15/1). The solvent was removed under reduced pressure, the dry residue was

dissolved in DCM, water was added to the solution and stirred for one hour. The organic fraction was separated, washed twice with water, dried over anhydrous sodium sulfate and the solvent was removed under reduced pressure, giving 892 mg (99% yield) of compound **Abi** as white powder.

^{1}**H NMR** (400 MHz, $CDCl_3$) δ, ppm: 8.62 (d, J=1.65 Hz, 1H, Py), 8.46 (dd, J=4.80, 1.56 Hz, 1H, Py), 7.65 (dt, J=7.93, 1.93 Hz, 1H, Py), 7.22 (dd, J=7.92, 4.86 Hz, 1H, Py), 6.00 (dd, J=3.21, 1.74 Hz, 1H, CH), 5.37-5.42 (m, 1H, CH), 3.47-3.61 (m, 1H, CH), 2.21-2.37 (m, 3H, CH_2), 2.01-2.12 (m, 3H, CH_2), 1.82-1.90 (m, 2H, CH_2), 1.74-1.82 (m, 1H, CH_2), 1.70-1.74 (m, 2H, CH_2), 1.64-1.70 (m, 3H, CH+CH_2), 1.44-1.64 (m, 4H, CH_2), 1.01-1.17 (m, 8H, CH_2+CH_3).

Synthesis of 4-(((3*S*,8*R*,9*S*,10*R*,13*S*,14*S*)-10,13-dimethyl-17-(pyridin-3-yl)-2,3,4,7,8,9,10,11,12,13,14,15-dodecahydro-1*H*-cyclopenta[*a*]phenanthren-3-yl)oxy)-4-oxobutanoic acid (Abi-Suc)

300 mg (0.858 mmol) of compound **Abi**, 129 mg (1.287 mmol, 1.5 eq) of succinic anhydride, 10 mg of 4-dimethylaminopyridine (0.0858 mmol, 0.1 eq) were dissolved in 20 ml dichloromethane. 180 μl (1.287 mmol, 1.5 eq.) of triethylamine was added to the mixture and stirred for 75 h under reflux. The solvent was removed under reduced pressure, after which the dry residue was precipitated with 0.1M hydrochloric acid solution. The resulting solid residue was washed with methanol. The methanol residue was removed under reduced pressure. 311 mg (86% yield) of **Abi-Suc** was obtained as white powder.

^{1}**H NMR** (400 MHz, DMSO-d6) δ, ppm: 8.75 (s, 1H, Py), 8.61 (d, J=5.38 Hz, 1H, Py), 8.42 (d, J=8.62 Hz, 1H, Py), 7.86 (t, J=7.03 Hz, 1H, Py), 6.39 (br. s, 1H, CH), 5.35 (d, J=3.55 Hz, 1H, CH), 4.42 (d, J=7.46 Hz, 1H, CH), 2.44 (m, 4H, CH_2), 2.24 (m, 3H, CH_2), 2.00-2.14 (m, 2H, CH_2), 1.98 (m, 1H, CH_2), 1.81 (m, 1H, CH_2), 1.57-1.76 (m, 4H, CH), 1.44-1.57 (m, 3H, CH+CH_2), 1.37 (m, 1H, CH_2), 1.18 (s, 1H, CH_2), 0.92-1.09 (m, 8H, CH+CH_3+CH_2).

Synthesis of (3*S*,8*R*,9*S*,10*R*,13*S*,14*S*)-10,13-dimethyl-17-(pyridin-3-yl)-2,3,4,7,8,9,10,11,12,13,14,15-dodecahydro-1*H*-cyclopenta[*a*]phenanthren-3-yl (2,5-dioxopyrrolidin-1-yl) succinate (7)

303 mg (0.623 mmol, 1 eq.) of **Abi-Suc**, 188 mg (0.981 mmol, 1.45 eq.) of N-(3-dimethylaminopropyl)-N'-ethylcarbodiimide hydrochloride, 113 mg (0.981 mmol, 1.45 eq.) of N-hydroxysuccinimide were dissolved in 15 ml of DCM. To the resulting mixture 245 μl (1.758 mmol, 2.8 eq.) of triethylamine was added. The reaction mixture was stirred for 20 hours, after which the solvent was removed under reduced pressure, the dry residue was dissolved in dichloromethane and extracted twice with water, once with saturated sodium bicarbonate solution and once with saturated sodium chloride solution. The organic fraction was dried over anhydrous sodium sulfate, after which the solvent was removed under reduced pressure. Compound **7** was obtained as white powder in an amount of 354 mg (96% yield).

^{1}H NMR (400 MHz, $CDCl_3$) δ, ppm: 8.59 (d, *J*=1.96 Hz, 1H, Ar), 8.44 (dd, *J*=5.09, 1.57 Hz, 1H, Ar), 7.72 (dt, *J*=7.92, 1.91 Hz, 1H, Ar), 7.28 (dd, *J*=7.83, 5.09 Hz, 1H, Ar), 6.01 (dd, *J*=2.93, 1.76 Hz, 1H, CH), 5.38 (d, *J*=4.30 Hz, 1H, CH), 4.57-4.69 (m, 1H, CH), 2.88-2.95 (m, 2H, CH_2), 2.80 (s, 4H, CH_2), 2.66-2.72 (m, 2H, CH_2), 2.32 (d, *J*=7.43 Hz, 2H, CH_2), 2.25 (ddd, *J*=15.94, 6.55, 3.33 Hz, 1H, CH), 1.97-2.09 (m, 3H, CH_2+CH), 1.79-1.88 (m, 2H, CH_2), 1.70-1.79 (m, 1H, CH), 1.51-1.70 (m, 6H, CH_3), 1.40-1.50 (m, 1H, CH), 1.06-1.19 (m, 2H, CH_2), 1.05 (s, 3H, CH_3), 1.01 (s, 3H, CH_3).

^{13}C NMR (101 MHz, $CDCl_3$) δ, ppm: 170.27, 168.88, 167.75, 151.02, 146.30, 139.85, 134.94, 133.55, 130.22, 123.52, 122.30, 74.66, 57.39, 50.12, 47.30, 37.89, 36.81, 36.72, 35.09, 31.82, 31.43, 30.31, 28.99, 27.52, 26.30, 25.54, 20.74, 19.19, 16.53.

The synthesis of the modified enzalutamide **14** was carried out according to the following scheme.

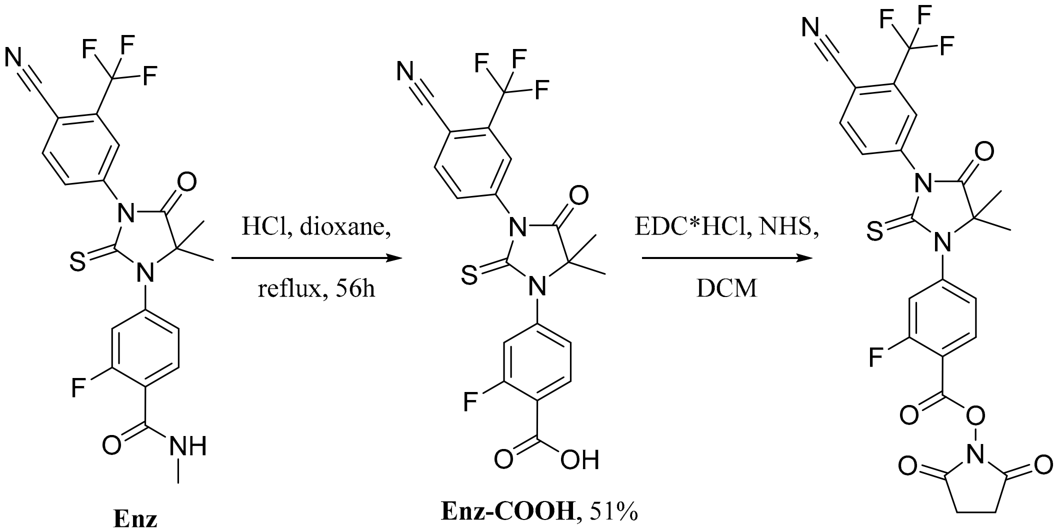

Enz **Enz-COOH**, 51% **14**, 84%

Figure S2. Scheme for the synthesis of modified enzalutamide **14**

In the first stage, acid hydrolysis of Enzalutamide (**Enz**) was carried out, and the product obtained (**Enz-COOH**) was converted into NHS-ether **14**.

Synthesis of 4-(3-(4-cyano-3-(trifluoromethyl)phenyl)-5,5-dimethyl-4-oxo-2-thioxoimidazolidin-1-yl)-2-fluorobenzoic acid (Enz-COOH)

200 mg **Enz** (0.431 mmol) was dissolved in 1,4-dioxane. Concentrated HCl was added to the mixture and brought to a HCl/dioxane ratio of 16/3 (v/v). The resulting mixture was boiled for 60 hours. Then the mixture was extracted with dichloromethane, the organic phase was washed with brine and dried over anhydrous sodium sulfate. The solvent was removed under reduced pressure. In this way 100 mg of the compound **Enz-COOH** (51% yield) as a white amorphous powder was obtained.

^{1}**H NMR** (400 MHz, DMSO-d6) δ, ppm: 13.55 (br. s., 1H, COOH) 8.40 (d, *J*=8.25 Hz, 1H, Ar) 8.28 (d, *J*=1.28 Hz, 1H, Ar) 7.99-8.11 (m, 2H, Ar) 7.45 (dd, *J*=11.10, 1.62 Hz, 1H, Ar) 7.36 (dd, *J*=8.28, 1.68 Hz, 1H, Ar) 1.54 (s, 6H, CH_3).

Synthesis of 2,5-dioxopyrrolidin-1-yl 4-(3-(4-cyano-3-(trifluoromethyl)phenyl)-5,5-dimethyl-4-oxo-2-thioxoimidazolidin-1-yl)-2-fluorobenzoate (14)

94 mg (0.208 mmol, 1 eq.) of compound **Enz-COOH**, 50 mg (0.26 mmol, 1.25 eq.) of EDC*HCl and 30 mg (0.26 mmol, 1.25 eq.) of N-hydroxysuccinimide were dissolved in 10 ml dichloromethane. The reaction mixture was stirred until the reaction was completed. The completeness of the reaction was monitored by TLC (eluent was DCM-methanol = 15/1). The solvent was removed under reduced pressure. The dry residue was dissolved in 20 mL dichloromethane and extracted with water, saturated sodium bicarbonate solution and saturated sodium chloride solution. The organic fraction was dried over anhydrous sodium sulfate. The solvent was removed under reduced pressure. 96 mg (yield 84%) of compound **14** was obtained as white powder.

^{1}**H NMR** (400 MHz, DMSO-d6) δ, ppm: 8.41 (d, *J*=8.25 Hz, 1H, Ar), 8.24-8.32 (m, 2H, Ar), 8.06-8.11 (m, 1H, Ar), 7,69 (dd, *J*=11.19, 1.71 Hz, 1H, Ar), 7.54 (dd, *J*=8.38, 1.71 Hz, 1H, Ar), 2.90 (m, 4H, CH_2), 1.57 (s, 6H, CH_3).

1.3.2. Synthesis of bimodal conjugates with the drug pair Docetaxel/Abiraterone

General procedure for the preparation of monoconjugates 8a-d

1 eq. of compound **6a-d** was dissolved in DMF. Compound **7** (1.2 eq.) and DIPEA (6 eq.) were added to the solution. The reaction mixture was stirred until the reaction was completed (the reaction was monitored by TLC in a system of 10% methanol in dichloromethane + 1% trifluoroacetic acid). The solvent was removed under reduced pressure. The dry residue was precipitated with acetonitrile, then decanted and the precipitate was washed three times with acetonitrile.

Synthesis of (3*S*,7*S*,25*S*,28*S*,31*S*)-31-((3-azidopropyl)carbamoyl)-25-benzyl-12-(3-chlorobenzyl)-40-(((3*S*,8*R*,9*S*,10*R*,13*S*,14*S*)-10,13-dimethyl-17-(pyridin-3-yl)-2,3,4,7,8,9,10,11,12,13,14,15-dodecahydro-1*H*-cyclopenta[*a*]phenanthren-3-yl)oxy)-28-(4-hydroxybenzyl)-5,13,20,23,26,29,37,40-octaoxo-4,6,12,19,24,27,30,36-octaazatetracontane-1,3,7-tricarboxylic acid (8a)

From 100 mg (0.0774 mmol) of compound **6a**, 51 mg (0.0929 mmol) of compound **7** in the presence of 81 µl (0.4644 mmol) of DIPEA in 10 ml of DMF 131 mg (85% yield) of compound **8a** was obtained as white powder.

^{1}**H NMR** (400 MHz, DMSO-d6) δ, ppm: 8.53 (s, 1H, Py), 8.39 (d, J=4.59 Hz, 1H, Py), 8.15 (d, J=6.79 Hz, 1H, NH), 8.01 (m, 1H, NH), 7.84-7.92 (m, 1H, NH), 7.74 (d, J=7.82 Hz, 1H,

Py), 7.55-7.64 (m, 1H, NH), 7.47 (m, 1H, NH), 7.23-7.40 (m, 3H, Ar+Py), 7.08-7.23 (m, 7H, Ar), 6.98-7.08 (m, 2H, Ar), 6.65 (d, J=8.01 Hz, 2H, Ar), 6.28-6.38 (m, 2H, NH), 6.08 (br. s, 1H, CH), 5.27-5.38 (m, 1H, CH), 4.36-4.52 (m, 3H, CH_2+CH), 4.24 (m, 2H, CH), 3.95-4.12 (m, 3H, CH), 3.28 (t, J=6.72 Hz, 2H, CH_2), 3.14 (m, 2H, CH_2), 2.99-3.10 (m, 3H, CH_2), 2.97 (m, 3H, CH_2), 2.74-2.93 (m, 4H, CH_2), 2.58-2.69 (m, 1H, CH_2), 2.42 (m, 2H, CH_2), 2.08-2.38 (m, 13H,), 1.92-2.01 (m, 3H, CH_2), 1.89 (m, 1H, CH_2), 1.78 (m, 2H, CH_2), 1.54-1.73 (m, 9H, CH_2), 1.26-1.53 (m, 14H, CH_2), 1.08-1.26 (m, 7H, CH_2+CH_3), 0.84-1.08 (m, 9H, CH_2+CH_3).

HPLC-MS: target compound content – 97%, t_R=8.26 min.

Synthesis of (3*S*,7*S*,25*S*,28*S*,31*S*)-31-((3-azidopropyl)carbamoyl)-25-benzyl-12-(4-bromobenzyl)-40-(((3*S*,8*R*,9*S*,10*R*,13*S*,14*S*)-10,13-dimethyl-17-(pyridin-3-yl)-2,3,4,7,8,9,10,11,12,13,14,15-dodecahydro-1*H*-cyclopenta[*a*]phenanthren-3-yl)oxy)-28-(4-hydroxybenzyl)-5,13,20,23,26,29,37,40-octaoxo-4,6,12,19,24,27,30,36-octaazatetracontane-1,3,7-tricarboxylic acid (8b)

From 81 mg (0.0606 mmol) of compound **6b**, 40 mg (0.0727 mmol) of compound **7** in the presence of 63 μl (0.3637 mmol) of DIPEA in 9 ml of DMF, 80 mg (80% yield) of compound **8b** was obtained as white powder.

^{1}H NMR (400 MHz, DMSO-d6) δ, ppm: 8.54 (s, 1H, Py), 8.39 (d, J=4.59 Hz, 1H, Py), 8.28-8.34 (m, 1H, NH), 8.15 (m, 1H, NH), 7.97-8.05 (m, 1H, NH), 7.92-7.97 (m, 1H, NH), 7.82-7.92 (m, 1H, NH), 7.74 (d, J=8.07 Hz, 1H, Py), 7.61 (t, J=8.34 Hz, 1H, NH), 7.41-7.55 (m, 3H, Ar+NH), 7.33 (m, 1H, Py), 6.98-7.24 (m, 9H, Ar), 6.65 (d, J=7.58 Hz, 2H, Ar), 6.26-6.36 (m, 2H, NH), 6.08 (br. s, 1H, CH), 5.32 (m, 1H, CH), 4.33-4.53 (m, 3H, CH_2+CH), 4.25 (m, 2H, CH), 3.94-4.10 (m, 3H, CH), 3.28 (t, J=6.82 Hz, 2H, CH_2), 2.74-3.22 (m, 13H, CH_2), 2.58-2.70 (m, 1H, CH_2), 2.38-2.45 (m, 2H, CH_2), 2.08-2.38 (m, 14H, CH_2), 1.83-2.08 (m, 5H, CH_2), 1.26-1.82 (m, 26H, CH_2), 1.08-1.26 (m, 7H, CH_2+CH_3), 0.84-1.07 (m, 9H, CH_2+CH_3).

HPLC-MS: target compound content – 97%, t_R=8.40 min.

Synthesis of (3*S*,7*S*,25*S*,28*S*,31*S*)-31-((3-azidopropyl)carbamoyl)-25-benzyl-12-(4-carboxybenzyl)-40-(((3*S*,8*R*,9*S*,10*R*,13*S*,14*S*)-10,13-dimethyl-17-(pyridin-3-yl)-2,3,4,7,8,9,10,11,12,13,14,15-dodecahydro-1*H*-cyclopenta[*a*]phenanthren-3-yl)oxy)-28-(4-hydroxybenzyl)-5,13,20,23,26,29,37,40-octaoxo-4,6,12,19,24,27,30,36-octaazatetracontane-1,3,7-tricarboxylic acid (8c)

From 32 mg (0.0246 mmol) of compound **6c**, 16 mg (0.0295 mmol) of compound **7** in the presence of 30 μl (0.1722 mmol) of DIPEA in 5 ml of DMF, 31 mg (78% yield) of compound **8c** was obtained as white powder.

HPLC-MS: target compound content – 99.9%, t_R=4.60 min.

ESI-HRMS: for $C_{85}H_{111}N_{13}O_{19}$: m/z calculated for $[M+H]^+$ 1618.8119, found: 1618.82.

Synthesis of (3*S*,7*S*,25*S*,28*S*,31*S*)-31-((3-azidopropyl)carbamoyl)-25,28-dibenzyl-12-(4-carboxybenzyl)-40-(((3*S*,8*R*,9*S*,10*R*,13*S*,14*S*)-10,13-dimethyl-17-(pyridin-3-yl)-2,3,4,7,8,9,10,11,12,13,14,15-dodecahydro-1*H*-cyclopenta[*a*]phenanthren-3-yl)oxy)-5,13,20,23,26,29,37,40-octaoxo-4,6,12,19,24,27,30,36-octaazatetracontane-1,3,7-tricarboxylic acid (8d)

From 48 mg (0.0373 mmol) of compound **6d**, 24 mg (0.0448 mmol) of compound **7** in the presence of 45 µl (0.2611 mmol) of DIPEA in 7 ml of DMF, 53 mg (88% yield) of compound **8d** was obtained as white powder.

^{1}H NMR (400 MHz, DMSO-d6) δ, ppm: 8.54 (s, 1H, Py), 8.39 (m, 1H, Py), 8.22 (m, 1H, NH), 8.05 (m, 1H, NH), 7.81-7.93 (m, 2H, NH), 7.74 (m, 1H, Py), 7.62 (m, 1H, NH), 7.53 (m, 1H, NH), 7.33 (m, 1H, Py), 7.21-7.29 (m, 6H, Ar), 7.06-7.21 (m, 6H, Ar), 6.31 (m, 1H, NH), 6.08 (m, 1H, CH), 5.32 (m, 1H, CH), 4.50-4.57 (m, 2H, CH_2), 4.38 (m, 2H, CH), 4.23 (m, 1H, CH), 4.06 (m, 2H, CH), 4.01 (m, 1H, CH), 3.29 (m, 2H, CH_2), 2.78-3.13 (m, 13H, CH_2), 2.57-2.68 (m, 1H, CH_2), 2.43 (m, 2H, CH_2), 2.15-2.30 (m, 13H, CH_2), 1.88-1.99 (m, 4H, CH_2), 1.77 (m, 2H, CH_2), 1.53-1.69 (m, 9H, CH_2), 1.30-1.50 (m, 14H, CH_2), 1.19 (m, 6H, CH_2+CH_3), 0.97 (m, 9H, CH_2+CH_3).

HPLC-MS: target compound content – 99.9%, t_R=6.89 min.

General procedure for the preparation of bimodal 10a-d conjugates using an azide-alkyne cycloaddition reaction.

Monoconjugate **8a-d** (1 eq.) and docetaxel-alkyne **9** (1.2 eq.) were dissolved in a mixture of DMF and water. The flask was filled with argon, then aqueous solutions of sodium ascorbate (1.2 eq.) and copper sulfate pentahydrate (0.4 eq.) were added to the system. The reaction mixture was stirred for 18 hours. Afterwards EDTA (0.8 eq.) was added and stirred for another three hours with access of air oxygen. The solvent was removed under reduced pressure, the dry residue was precipitated with acetonitrile and decanted. Then the product was isolated individually by reverse phase column chromatography using acetonitrile-water mixture as eluent.

Synthesis of (3*S*,7*S*,25*S*,28*S*,31*S*)-31-((3-(4-(4-(((2*R*,3*S*)-1-(((2a*R*,4*S*,4a*S*,6*R*,9*S*,11*S*,12*S*,12a*R*,12b*S*)-12b-acetoxy-12-(benzoyloxy)-4,6,11-trihydroxy-4a,8,13,13-tetramethyl-5-oxo-2a,3,4,4a,5,6,9,10,11,12,12a,12b-dodecahydro-1*H*-7,11-methanocyclodeca[3,4]benzo[1,2-*b*]oxet-9-yl)oxy)-3-((*tert*-butoxycarbonyl)amino)-1-oxo-3-phenylpropan-2-yl)oxy)-4-oxobutyl)-1*H*-1,2,3-triazol-1-yl)propyl)carbamoyl)-25-benzyl-12-(3-chlorobenzyl)-40-(((3*S*,8*R*,9*S*,10*R*,13*S*,14*S*)-10,13-dimethyl-17-(pyridin-3-yl)-2,3,4,7,8,9,10,11,12,13,14,15-dodecahydro-1*H*-cyclopenta[*a*]phenanthren-3-yl)oxy)-28-(4-hydroxybenzyl)-5,13,20,23,26,29,37,40-octaoxo-4,6,12,19,24,27,30,36-octaazatetracontane-1,3,7-tricarboxylic acid (10a)

From 60 mg (0.0373 mmol) of compound **8a**, 37 mg (0.040 mmol) of docetaxel-alkine **9**, 9 mg (0.04476 mmol) of sodium ascorbate, 4 mg (0.01492 mmol) of copper sulfate pentahydrate in 8 ml DMPA/water mixture (3/1), followed by addition of 9 mg (0, 02984 mmol) EDTA was isolated individually by reverse phase column chromatography (InterchimPuriflashC18 20 g, 15μ, gradient from 15% acetonitrile to 100% acetonitrile in 20 min, flow rate 20 ml/min) 85 mg (90% yield) of compound **10a** as white powder.

^{1}H NMR (400 MHz, DMSO-d6) δ, ppm: 8.76 (s, 1H, Py), 8.62 (d, J=5.32 Hz, 1H, Py), 8.33 (d, J=8.3 Hz, 1H, Py), 7.94 (d, J=7.40 Hz, 2H, Ar), 7.82 (m, 1H, Py), 7.78 (s, 1H, Triazole), 7.69 (m, 1H, Ar), 7.56-7.66 (m, 2H, Ar), 7.21-7.44 (m, 6H, Ar), 6.97-7.21 (m, 10H, Ar), 6.65 (d, J=8.01 Hz, 2H, Ar), 6.37 (br. s, 1H, CH), 5.75 (m, 1H, CH), 5.36 (d, J=7.09 Hz, 1H, CH), 5.31-5.34 (m, 1H, CH), 4.98-5.09 (m, 3H, CH), 4.84-4.90 (m, 1H, CH), 4.36-4.52 (m, 3H, CH_2+CH), 4.19-4.33 (m, 4H, CH_2+CH), 4.03-4.11 (m, 2H, CH), 3.93-4.03 (m, 4H, CH_2), 3.14 (m, 2H, CH_2), 2.97 (m, 7H, CH_2), 2.77-2.90 (m, 2H, CH_2), 2.54-2-70 (m, 3H, CH_2), 2.37-2.46 (m, 4H, CH_2), 2.30 (d, J=6.42 Hz, 6H, CH_2), 2.20-2.26 (m, 6H, CH_2), 2.19 (m, 4H, CH_2), 2.15 (m, 2H, CH_2), 2.04-2.08 (m, 1H, CH_2), 1.97-2.02 (m, 1H, CH_2), 1.72-1.97 (m, 9H, CH_2), 1.68-1.72 (m, 2H, CH_2) 1.66 (m, 4H, CH_3+CH_2), 1.54-1.64 (m, 5H, CH_2), 1.47 (m, 11H, CH_3+CH_2), 1.29 (s, 10H, CH_3+CH_2), 0.98 (d, J=3.24 Hz, 8H, CH_2), 0.94 (br. s., 6H, CH_3).

^{13}C NMR (101 MHz, DMSO-d6) δ, ppm: 209.29, 174.39, 174.05, 173.76, 172.45, 172.18, 171.87, 171.81, 171.57, 171.24, 170.89, 169.66, 169.20, 165.40, 157.34, 155.64, 155.23, 148.53, 145.94, 141.01, 140.76, 140.59, 140.28, 139.83, 137.58, 136.76, 136.10, 134.62, 133.57, 133.35, 133.07, 130.62, 130.28, 129.99, 129.88, 129.58, 128.92, 128.74, 128.64, 128.13, 128.00, 127.39, 127.08, 126.88, 126.38, 126.20, 126.00, 124.92, 122.13, 121.86, 118.22, 114.94, 83.80, 80.22, 78.65, 76.75, 75.03, 74.68, 73.62, 73.24, 71.20, 70.67, 56.96, 56.88, 55.22, 52.86, 52.13, 51.51, 49.49, 46.88, 46.65, 42.84, 37.62, 36.25, 35.68, 34.57, 33.99, 32.62, 31.87, 31.52, 31.18, 30.85, 30.61, 29.75, 28.94, 28.73, 28.03, 27.32, 26.38, 26.21, 24.70, 24.25, 24.09, 22.81, 22.47, 20.76, 20.26, 18.87, 15.92, 13.66, 9.78.

HPLC-MS: target compound content – 99.9%, t_R=9.96 min.

ESI-HRMS: for $C_{133}H_{169}ClN_{14}O_{32}$: m/z calculated for $[M+H]^+$ 2510.1716, found: 2510.18.

Synthesis of (3*S*,7*S*,25*S*,28*S*,31*S*)-31-((3-(4-(4-(((2*R*,3*S*)-1-(((2a*R*,4*S*,4a*S*,6*R*,9*S*,11*S*,12*S*,12a*R*,12b*S*)-12b-acetoxy-12-(benzoyloxy)-4,6,11-trihydroxy-4a,8,13,13-tetramethyl-5-oxo-2a,3,4,4a,5,6,9,10,11,12,12a,12b-dodecahydro-1*H*-7,11-methanocyclodeca[3,4]benzo[1,2-*b*]oxet-9-yl)oxy)-3-((*tert*-butoxycarbonyl)amino)-1-oxo-3-phenylpropan-2-yl)oxy)-4-oxobutyl)-1*H*-1,2,3-triazol-1-yl)propyl)carbamoyl)-25-benzyl-12-(4-bromobenzyl)-40-(((3*S*,8*R*,9*S*,10*R*,13*S*,14*S*)-10,13-dimethyl-17-(pyridin-3-yl)-

2,3,4,7,8,9,10,11,12,13,14,15-dodecahydro-1*H*-cyclopenta[*a*]phenanthren-3-yl)oxy)-28-(4-hydroxybenzyl)-5,13,20,23,26,29,37,40-octaoxo-4,6,12,19,24,27,30,36-octaazatetracontane-1,3,7-tricarboxylic acid (10b)

From 40 mg (0.0242 mmol) of compound **8b**, 24 mg (0.0266 mmol) of docetaxel-alkine **9**, 6 mg (0.029 mmol) of sodium ascorbate, 3 mg (0.0097 mmol) of copper sulfate pentahydrate in 8 ml DMPA/water mixture (3/1), followed by addition of 6 mg (0, 0194 mmol) EDTA was isolated individually by reverse phase column chromatography (InterchimPuriflashC18 20 g, 15μ, gradient from 15% acetonitrile to 100% acetonitrile in 20 min, flow rate 20 ml/min) 40 mg (65% yield) of compound **10b** as white powder.

^{1}H NMR (400 MHz, DMSO-d6) δ, ppm: 8.73 (s, 1H, Py), 8.60 (d, J=5.01 Hz, 1H, Py), 8.26 (d, J=7.82 Hz, 1H, Py), 7.94 (m, 2H, Ar), 7.78 (s, 1H, Triazole), 7.66-7.77 (m, 2H, Ar), 7.58-7.66 (m, 2H, Ar), 7.50 (d, J=8.31 Hz, 1H, Ar), 7.45 (d, J=8.31 Hz, 1H, Ar), 7.38 (m, 2H, Ar), 7.31 (m, 2H, Ar), 7.13-7.22 (m, 3H, Ar), 7.00-7.13 (m, 7H, Ar), 6.65 (d, J=7.89 Hz, 2H, Ar), 6.33 (br. s, 1H, CH), 5.74 (t, J=8.34 Hz, 1H, CH), 5.29-5.40 (m, 2H, CH), 4.97-5.10 (m, 3H, CH+OH), 4.87 (m, 1H, CH), 4.40-4.46 (m, 3H, CH+CH_2), 4.18-4.33 (m, 4H, CH), 3.92-4.12 (m, 7H, CH_2+CH), 3.06-3.22 (m, 3H, CH_2), 2.90-3.06 (m, 8H, CH_2), 2.80-2.88 (m, 2H, CH_2), 2.66 (m, 1H, CH_2), 2.58 (t, J=7.37 Hz, 2H, CH_2), 2.42 (m, 5H, CH_2), 2.26-2.37 (m, 7H, CH_2), 2.10-2.26 (m, 12H, CH_2), 1.54-1.92 (m, 21H, CH_2), 1.47 (m, 12H, CH_2), 1.33-1.42 (m, 6H, CH_2), 1.29 (m, 11H, CH_3+CH_2), 1.07-1.25 (m, 8H, CH_2), 0.83-1.05 (m, 15H, CH_3+CH_2).

HPLC-MS: target compound content – 99.9%, t_R=10.06 min.

ESI-HRMS: for $C_{133}H_{169}BrN_{14}O_{32}$: m/z calculated for $[M+H]^+$ 2554.1211, found: 2554.13.

Synthesis of (3*S*,7*S*,25*S*,28*S*,31*S*)-31-((3-(4-(4-(((2*R*,3*S*)-1-(((2a*R*,4*S*,4a*S*,6*R*,9*S*,11*S*,12*S*,12a*R*,12b*S*)-12b-acetoxy-12-(benzoyloxy)-4,6,11-trihydroxy-4a,8,13,13-tetramethyl-5-oxo-2a,3,4,4a,5,6,9,10,11,12,12a,12b-dodecahydro-1*H*-7,11-methanocyclodeca[3,4]benzo[1,2-*b*]oxet-9-yl)oxy)-3-((*tert*-butoxycarbonyl)amino)-1-oxo-3-phenylpropan-2-yl)oxy)-4-oxobutyl)-1*H*-1,2,3-triazol-1-yl)propyl)carbamoyl)-25-benzyl-12-(4-carboxybenzyl)-40-(((3*S*,8*R*,9*S*,10*R*,13*S*,14*S*)-10,13-dimethyl-17-(pyridin-3-yl)-2,3,4,7,8,9,10,11,12,13,14,15-dodecahydro-1*H*-cyclopenta[*a*]phenanthren-3-yl)oxy)-28-(4-hydroxybenzyl)-5,13,20,23,26,29,37,40-octaoxo-4,6,12,19,24,27,30,36-octaazatetracontane-1,3,7-tricarboxylic acid (10c)

From 15 mg (0.00926 mmol) of compound **8c**, 10 mg (0.0111 mmol) of docetaxel-alkine **9**, 2.2 mg (0.0111 mmol) of sodium ascorbate, 1 mg (0.0037 mmol) of copper sulfate pentahydrate in 6.7 ml of DMPA/water mixture (3/1), followed by the addition of 2.2 mg (0, 00741 mmol) EDTA was isolated individually by reverse phase column chromatography (InterchimPuriflashC18

20 g, 15μ gradient, 15% acetonitrile to 100% acetonitrile in 20 min, flow rate 20 ml/min) 14 mg (77% yield) of compound **10c** as white powder.

^{1}H NMR (400 MHz, DMSO-d6) δ, ppm: 8.82 (br. s, 1H, NH), 8.69 (br. s, 1H, NH), 8.49 (d, J=8.07 Hz, 1H, Py), 7.87-8.01 (m, 4H, Ar+Py), 7.85 (d, J=8.07 Hz, 1H, Py), 7.78 (s, 1H, Triazole), 7.65-7.75 (m, 1H, Py), 7.57-7.65 (m, 2H, Ar), 7.34-7.47 (m, 2H, Ar), 7.31 (m, 2H, Ar), 7.25 (m, 2H, Ar), 7.13 (m, 2H, Ar), 7.17 (m, 2H, Ar), 6.98-7.11 (m, 4H, Ar), 6.65 (d, J=6.42 Hz, 2H, Ar), 6.43 (br. s, 1H, CH), 5.69-5.79 (m, 1H, CH), 5.29-5.39 (m, 2H, CH+OH), 4.97-5.08 (m, 3H, CH), 4.87 (d, J=9.96 Hz, 1H, CH), 4.45-4.57 (m, 2H, CH_2), 4.40 (br. s, 1H, OH), 4.15-4.32 (m, 4H, CH+CH_2), 4.05 (m, 2H, CH), 3.91-4.03 (m, 4H, CH+CH_2), 3.58-3.60 (m, 1H, CH), 3.15 (m, 2H, CH_2), 2.89-3.09 (m, 8H, CH_2), 2.84 (m, 2H, CH_2), 2.64 (m, 1H, CH_2), 2.58 (m, 2H, CH_2), 2.42 (d, J=6.66 Hz, 4H, CH_2), 2.30 (m, 7H, CH_2), 2.10-2.26 (m, 11H, CH_2), 2.03-2.10 (m, 2H, CH_2), 1.97 (m, 1H, CH_2), 1.72-1.93 (m, 8H, CH_2), 1.69 (m, 2H, CH_2), 1.66 (m, 4H, CH_2), 1.58 (s, 2H,), 1.61 (s, 3H,), 1.47 (br. s., 12H,), 1.31-1.41 (m, 6H,), 1.29 (s, 9H, CH_2), 1.19 (m, 6H, CH_2+CH_3), 1.08 (m, 2H, CH_2), 0.83-1.06 (m, 15H, CH_2+CH_3).

HPLC-MS: target compound content – 99.9%, t_R=8.81 min.

ESI-HRMS: for $C_{134}H_{170}N_{14}O_{34}$: m/z calculated for $[M+H]^+$ 2520.2004, found: 2520.21.

Synthesis of (3*S*,7*S*,25*S*,28*S*,31*S*)-31-((3-(4-(4-(((2*R*,3*S*)-1-(((2a*R*,4*S*,4a*S*,6*R*,9*S*,11*S*,12*S*,12a*R*,12b*S*)-12b-acetoxy-12-(benzoyloxy)-4,6,11-trihydroxy-4a,8,13,13-tetramethyl-5-oxo-2a,3,4,4a,5,6,9,10,11,12,12a,12b-dodecahydro-1*H*-7,11-methanocyclodeca[3,4]benzo[1,2-*b*]oxet-9-yl)oxy)-3-((*tert*-butoxycarbonyl)amino)-1-oxo-3-phenylpropan-2-yl)oxy)-4-oxobutyl)-1*H*-1,2,3-triazol-1-yl)propyl)carbamoyl)-25,28-dibenzyl-12-(4-carboxybenzyl)-40-(((3*S*,8*R*,9*S*,10*R*,13*S*,14*S*)-10,13-dimethyl-17-(pyridin-3-yl)-2,3,4,7,8,9,10,11,12,13,14,15-dodecahydro-1*H*-cyclopenta[*a*]phenanthren-3-yl)oxy)-5,13,20,23,26,29,37,40-octaoxo-4,6,12,19,24,27,30,36-octaazatetracontane-1,3,7-tricarboxylic acid (10d)

From 48 mg (0.0299 mmol) of compound **8d**, 30 mg (0.0329 mmol) of docetaxel-alkine **9**, 7 mg (0.0359 mmol) of sodium ascorbate, 3 mg (0.0119 mmol) of copper sulfate pentahydrate in 10.6 ml of DMPA/water mixture (3/1), followed by addition of 7 mg (0, 0238 mmol) EDTA was isolated individually by reverse phase column chromatography (InterchimPuriflash C18 20 g, 15μ, gradient from 15% acetonitrile to 100% acetonitrile in 20 min, flow rate 20 ml/min) 64 mg (85% yield) of compound **10d** as white powder.

^{1}H NMR (400 MHz, DMSO-d6) δ, ppm: 8.82 (br. s, 1H, Py), 8.69 (d, J=5.32 Hz, 1H, Py), 8.51 (d, J=7.95 Hz, 1H, Py), 7.92-7.99 (m, 3H, Ar), 7.80-7.92 (m, 2H, Py+Ar), 7.78 (s, 1H, Triazole), 7.65-7.75 (m, 1H, Ar), 7.58-7.65 (m, 2H, Ar), 7.28-7.41 (m, 4H, Ar), 7.20-7.28 (m, 7H, Ar), 7.01-7.20 (m, 8H, Ar), 6.44 (br. s, 1H, CH), 5.67-5.79 (m, 1H, CH), 5.36 (d, J=7.09 Hz, 1H,

CH), 5.31 (m, 1H, CH), 4.97-5.08 (m, 3H, CH), 4.87 (d, J=9.54 Hz, 1H, CH), 4.50-4.57 (m, 2H, CH_2), 4.32-4.45 (m, 2H, CH), 4.17-4.31 (m, 3H, CH), 3.92-4.12 (m, 7H, CH+ CH_2), 3.59 (d, J=6.36 Hz, 1H, CH_2), 3.09-3.22 (m, 3H, CH_2), 2.89-3.09 (m, 9H, CH_2), 2.78-2.89 (m, 1H, CH_2), 2.54-2.68 (m, 3H, CH_2), 2.41 (d, J=7.15 Hz, 4H, CH_2), 2.10-2.36 (m, 18H, CH_2), 1.77-2.10 (m, 11H, CH_2), 1.54-1.74 (m, 12H, CH_2), 1.47 (m, 12H, CH_2), 1.24-1.41 (m, 16H, CH_3+CH_2), 1.06-1.24 (m, 8H, CH_2), 0.98 (d, J=5.87 Hz, 8H, CH_3+CH_2), 0.94 (br. s, 6H, CH_3).

HPLC-MS: target compound content – 99.9%, t_R=9.49 min.

ESI-HRMS: for $C_{134}H_{170}N_{14}O_{33}$: m/z calculated for $[M+H]^+$ 2504.2055, found: 2504.21.

1.3.3. Synthesis of bimodal conjugates with the MMAE/Abiraterone drug pair

Synthetic procedure for producing bimodal conjugate 13a from compound 8a.

Initially, a synthesis scheme involving an azide-alkyne cycloaddition reaction of monoconjugate **9a** and modified monomethyl auristatin E **11** was proposed. The methodology was validated using the *m*-Cl substituted monoconjugate **9a**.

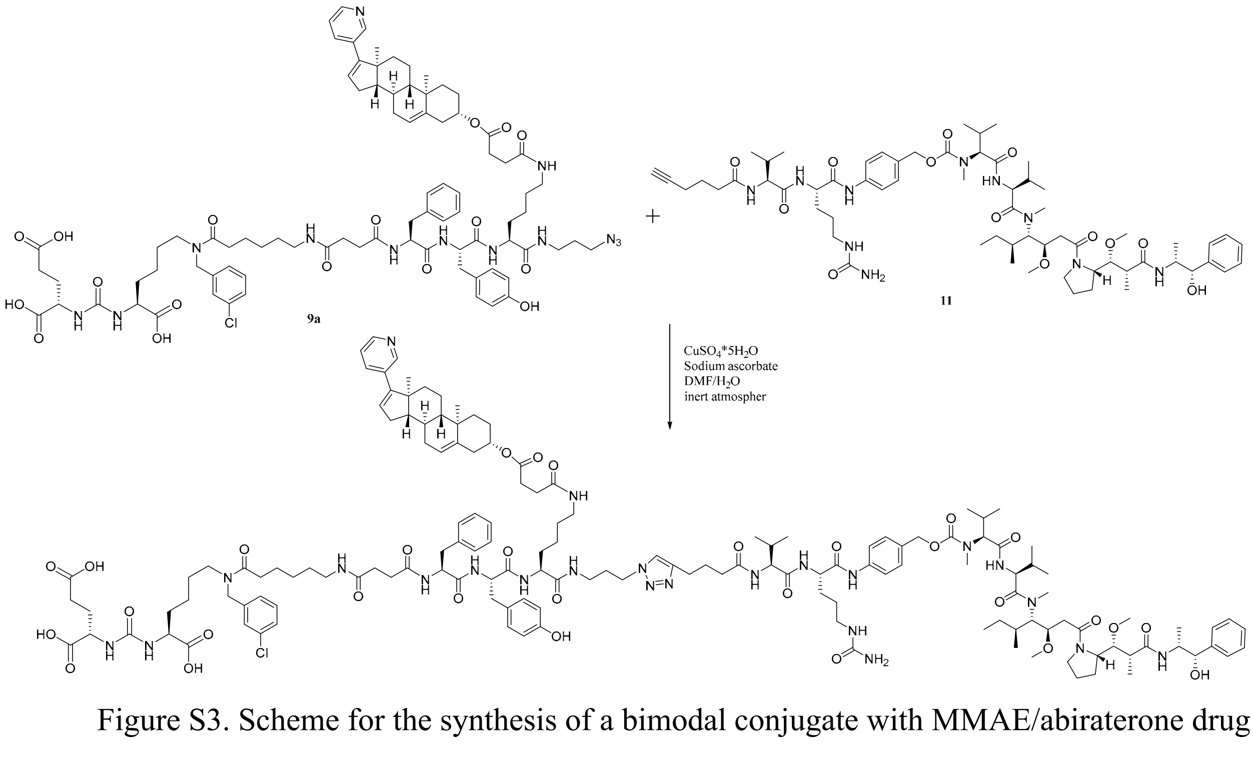

Figure S3. Scheme for the synthesis of a bimodal conjugate with MMAE/abiraterone drug pair based on compound **9a**

Monoconjugate **9a** (1.1 eq.) and MMAE-alkyne (compound **11**, 1 eq.) were dissolved in a mixture of DMF and water. The flask was filled with argon, then aqueous solutions of sodium ascorbate (1.2 eq.) and copper sulfate pentahydrate (0.4 eq.) were added to the system. The reaction mixture was stirred for 18 hours. Afterwards EDTA (0.8 eq.) was added and stirred for three hours with access of air oxygen. The solvent was removed under reduced pressure, then the dry residue was precipitated with acetonitrile and decanted. The product was then isolated individually by reverse phase column chromatography using acetonitrile-water mixture as eluent.

However, it was not possible to isolate the product individually by the available preparative methods. An impurity was observed in the separated mixture by column chromatography. Further analysis of the HPLC-MS data showed a content of 73% of the target product and 26% of the impurity. Based on the m/z difference of the target product and the impurity equal to 175, it can be assumed that the byproduct is a stable copper complex with the bimodal conjugate obtained, containing also trifluoroacetate as ligand.

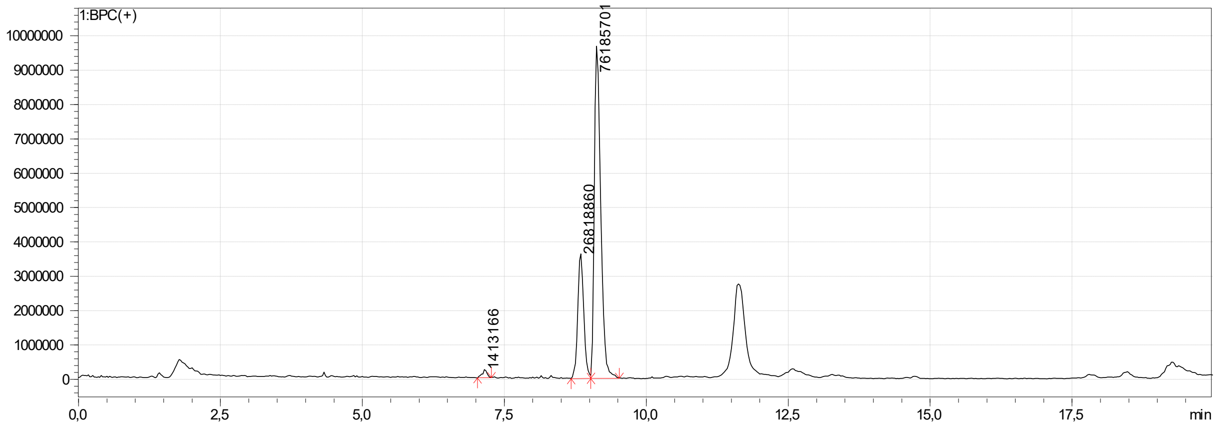

(a)

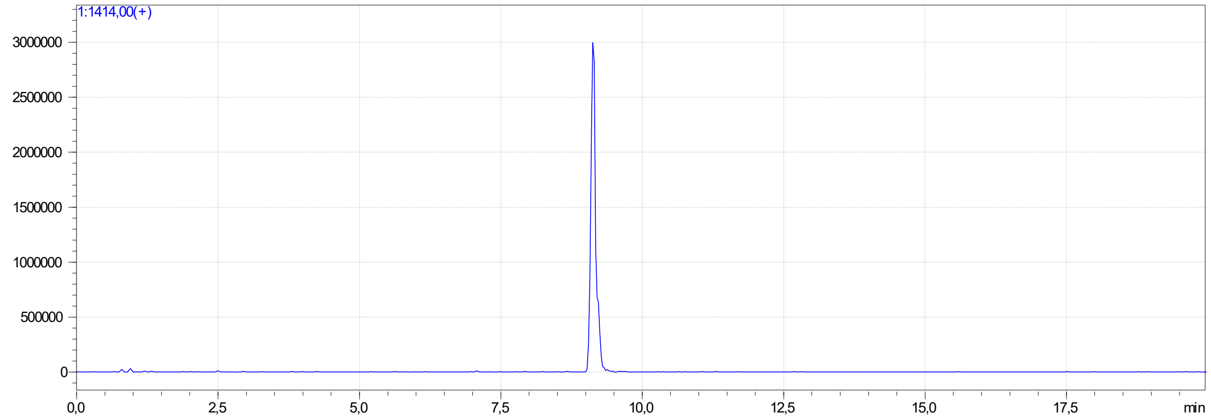

(b)

Figure S4. Results of HPLC-MS study of the conjugate preparation reaction: (a) total ionic current, in positive ions, 73% target compound content, 26% by-product content; (b) "extraction" of the $[M+2H]^{2+}$ target compound ion from the total ionic current.

General procedure for preparing monomodal conjugates 12a-d

Ligand **6a-d** (1.1 eq.) and MMAE-alkyne (compound **11**, 1 eq.) were dissolved in a mixture of DMF and water. The flask was filled with argon, then aqueous solutions of sodium ascorbate (1.2 eq.) and copper sulfate pentahydrate (0.4 eq.) were added. The reaction mixture was stirred for 18 hours. Afterwards EDTA (0.8 eq.) was added and stirred for three hours with access of air oxygen. The solvent was removed under reduced pressure, then the dry residue was precipitated

with acetonitrile and decanted. Then the product was isolated individually by reverse phase column chromatography using acetonitrile-water mixture as eluent.

Synthesis of (3*S*,7*S*,25*S*,28*S*,31*S*)-31-(4-aminobutyl)-25-benzyl-36-(4-(4-(((*S*)-1-(((*S*)-1-((4-((5*S*,8*S*,11*S*,12*R*)-11-((*S*)-*sec*-butyl)-12-(2-((*S*)-2-((1*R*,2*R*)-3-(((1*S*,2*R*)-1-hydroxy-1-phenylpropan-2-yl)amino)-1-methoxy-2-methyl-3-oxopropyl)pyrrolidin-1-yl)-2-oxoethyl)-5,8-diisopropyl-4,10-dimethyl-3,6,9-trioxo-2,13-dioxa-4,7,10-triazatetradecyl)phenyl)amino)-1-oxo-5-ureidopentan-2-yl)amino)-3-methyl-1-oxobutan-2-yl)amino)-4-oxobutyl)-1*H*-1,2,3-triazol-1-yl)-12-(3-chlorobenzyl)-28-(4-hydroxybenzyl)-5,13,20,23,26,29,32-heptaoxo-4,6,12,19,24,27,30,33-octaazahexatriacontane-1,3,7-tricarboxylic acid (12a)

From 104 mg (0.0805 mmol) of compound **6a**, 89 mg (0.0732 mmol) of compound **11**, 17 mg (0.08784 mmol) of sodium ascorbate, 6 mg (0.02928 mmol) of copper sulfate pentahydrate in 13.5 ml DMF/water mixture (3/1), followed by addition of 17 mg (0.05856 mmol) EDTA was isolated individually by reverse phase column chromatography (InterchimPuriflash C18 40 g, 15μ, gradient from 10% acetonitrile to 100% acetonitrile in 30 min, flow rate 20 ml/min) 86 mg (49% yield) of compound **12a** as white powder.

^{1}H NMR (400 MHz, DMSO-d6) δ, ppm: 12.51 (br. s, 3H, COOH), 10.00 (m, 1H, NH), 9.22 (br. s, 1H, OH), 8.28 (m, 2H, NH), 8.13 (m, 3H, NH), 7.84-7.96 (m, 3H, NH), 7.82 (s, 2H, Triazole+NH), 7.52-7.74 (m, 6H, Ar+NH), 7.09-7.40 (m, 16H, Ar), 7.00-7.07 (m, 2H, Ar), 6.65 (d, J=7.82Hz, 2H, Ar), 6.31 (m, 2H, NH), 5.99 (br. s, 1H, NH), 5.42 (br. s, 2H, NH_2), 4.90-5.13 (m, 2H, CH_2), 4.59-4.77 (m, 1H, CH), 4.44-4.59 (m, 3H, CH_2+CH), 4.18-4.43 (m, 8H, CH+CH_2), 3.90-4.17 (m, 5H, CH), 3.30 (m, 2H, CH_2), 3.14-3.26 (m, 9H, CH_2+CH_3), 3.10 (s, 2H, CH_2), 2.91-3.07 (m, 9H, CH_2), 2.70-2.90 (m, 7H, CH_2), 2.61-2.69 (m, 1H, CH_2), 2.56 (t, J=7.49 Hz, 2H, CH_2), 2.30-2.44 (m, 3H, CH_2), 2.14-2.29 (m, 9H, CH_2), 2.11 (m, 1H, CH_2), 1.84-2.01 (m, 5H, CH_2), 1.56-1.84 (m, 10H, CH_2), 1.34-1.56 (m, 13H, CH_2+CH_3), 1.10-1.34 (m, 8H, CH_2), 0.94-1.07 (m, 6H, CH_2), 0.67-0.89 (m, 23H, CH_2+CH_3).

^{13}C NMR (101 MHz, DMSO-d6) δ, ppm: 174.50, 174.20, 173.77, 172.52, 172.17, 172.11, 171.71, 171.50, 171.34, 171.10, 170.62, 169.85, 168.75, 158.96, 157.29, 155.88, 146.46, 143.69, 141.22, 137.88, 133.41, 133.05, 130.62, 130.26, 130.05, 129.03, 128.18, 128.08, 127.83, 127.77, 127.19, 126.86, 126.68, 126.43, 126.29, 126.09, 124.97, 121.96, 118.81, 114.98, 114.84, 81.64, 74.82, 66.11, 63.29, 60.95, 58.18, 57.56, 57.14, 54.92, 53.18, 52.57, 52.15, 51.66, 50.28, 49.75, 49.18, 47.23, 46.86, 46.26, 43.77, 43.21, 38.71, 37.14, 36.97, 35.81, 35.06, 34.67, 32.28, 31.81, 31.57, 31.21, 30.79, 30.59, 30.47, 29.91, 29.84, 29.34, 29.10, 27.79, 27.54, 26.83, 26.65, 26.29, 25.43, 24.66, 24.37, 23.14, 22.52, 22.26, 19.28, 18.95, 18.77, 18.56, 18.40, 18.24, 15.91, 15.48, 15.33, 15.05, 10.32.

HPLC-MS: target compound content – 99.9%, t_R=11.47 min.

ESI-HRMS: for $C_{120}H_{177}ClN_{22}O_{27}$: m/z calculated for $[M+2H]^{2+}$ 1197.64938, found: 1197.645.

Synthesis of (3*S*,7*S*,25*S*,28*S*,31*S*)-31-(4-aminobutyl)-25-benzyl-12-(4-bromobenzyl)-36-(4-(4-(((*S*)-1-(((*S*)-1-((4-((5*S*,8*S*,11*S*,12*R*)-11-((*S*)-*sec*-butyl)-12-(2-((*S*)-2-((1*R*,2*R*)-3-(((1*S*,2*R*)-1-hydroxy-1-phenylpropan-2-yl)amino)-1-methoxy-2-methyl-3-oxopropyl)pyrrolidin-1-yl)-2-oxoethyl)-5,8-diisopropyl-4,10-dimethyl-3,6,9-trioxo-2,13-dioxa-4,7,10-triazatetradecyl)phenyl)amino)-1-oxo-5-ureidopentan-2-yl)amino)-3-methyl-1-oxobutan-2-yl)amino)-4-oxobutyl)-1*H*-1,2,3-triazol-1-yl)-28-(4-hydroxybenzyl)-5,13,20,23,26,29,32-heptaoxo-4,6,12,19,24,27,30,33-octaazahexatriacontane-1,3,7-tricarboxylic acid (12b)

From 80 mg (0.06546 mmol) of compound **6b**, 73 mg (0.05951 mmol) of compound **11**, 14 mg (0.07141 mmol) of sodium ascorbate, 6 mg (0.0238 mmol) of copper sulfate pentahydrate in 10.8 ml DMF/water mixture (3/1), followed by addition of 14 mg (0.04761 mmol) EDTA was isolated individually by reverse phase column chromatography (InterchimPuriflash C18 40 g, 15μ, gradient from 10% acetonitrile to 100% acetonitrile in 30 min, flow rate 20 ml/min) 60 mg (41% yield) of compound **12b** as white powder.

^{1}H NMR (400 MHz, DMSO-d6) δ, ppm: 12.52 (br. s, 3H, COOH), 9.99 (m, 1H, NH), 9.20 (br. s, 1H, OH), 8.24-8.37 (m, 2H, NH), 8.11-8.15 (m, 3H, NH), 7.84-7.96 (m, 3H, NH), 7.82 (s, 2H, NH+Triazole), 7.59-7.70 (m, 4H, Ar), 7.51-7.59 (m, 3H, NH+Ar), 7.48 (d, J=8.31 Hz, 1H, Ar), 7.08-7.35 (m, 13H, Ar), 7.03 (m, 2H, Ar), 6.65 (d, J=7.76 Hz, 2H, Ar), 6.29-6.33 (m, 2H, NH), 6.00 (br. s, 1H, NH), 5.41 (br. s, 2H, NH), 4.95-5.14 (m, 2H, CH_2), 4.62-4.73 (m, 1H, CH), 4.23-4.53 (m, 10H, CH+CH_2), 3.38-4.23 (m, 27H, H_2O+CH+CH_2), 3.08-3.26 (m, 10H, CH_2+CH_3), 2.78-3.08 (m, 13H, CH_2+CH_3), 2.70-2.78 (m, 2H, CH_2), 2.60-2.69 (m, 1H, CH_2), 2.52-2.60 (m, 2H, CH_2), 2.15-2.42 (m, 11H, CH_2), 2.11 (m, 1H, CH_2), 1.84-2.04 (m, 5H, CH_2), 1.63-1.84 (m, 7H, CH_2), 1.33-1.58 (m, 13H, CH_2+CH_3), 1.11-1.33 (m, 7H, CH_2), 0.89-1.08 (m, 6H, CH_2), 0.65-0.89 (m, 21H, CH_2+CH_3).

^{13}C NMR (101 MHz, DMSO-d6) δ, ppm: 174.51, 174.22, 173.78, 172.37, 172.09, 171.71, 171.50, 171.35, 171.10, 168.74, 158.96, 157.28, 155.88, 146.45, 143.70, 138.03, 137.88, 131.57, 131.25, 130.05, 129.73, 129.03, 128.65, 128.19, 128.08, 127.83, 126.47, 121.97, 119.93, 114.98, 74.80, 60.95, 60.31, 57.18, 54.92, 54.15, 51.65, 46.86, 43.21, 38.70, 37.48, 36.99, 35.80, 34.67, 31.80, 30.48, 29.91, 29.02, 27.54, 26.65, 26.30, 25.43, 24.65, 24.36, 22.27, 19.28, 18.24, 15.49, 15.32, 10.31.

HPLC-MS: target compound content – 99.9%, t_R=11.68 min.

ESI-HRMS: for $C_{120}H_{177}BrN_{22}O_{27}$: m/z calculated for $[M+2H]^{2+}$ 1219.62412, found: 1219.6244.

Synthesis of (3*S*,7*S*,25*S*,28*S*,31*S*)-31-(4-aminobutyl)-25-benzyl-36-(4-(4-(((*S*)-1-(((*S*)-1-((4-((5*S*,8*S*,11*S*,12*R*)-11-((*S*)-*sec*-butyl)-12-(2-((*S*)-2-((1*R*,2*R*)-3-(((1*S*,2*R*)-1-hydroxy-1-phenylpropan-2-yl)amino)-1-methoxy-2-methyl-3-oxopropyl)pyrrolidin-1-yl)-2-oxoethyl)-5,8-diisopropyl-4,10-dimethyl-3,6,9-trioxo-2,13-dioxa-4,7,10-triazatetradecyl)phenyl)amino)-1-oxo-5-ureidopentan-2-yl)amino)-3-methyl-1-oxobutan-2-yl)amino)-4-oxobutyl)-1*H*-1,2,3-triazol-1-yl)-12-(4-carboxybenzyl)-28-(4-hydroxybenzyl)-5,13,20,23,26,29,32-heptaoxo-4,6,12,19,24,27,30,33-octaazahexatriacontane-1,3,7-tricarboxylic acid (12c)

From 100 mg (0.08423 mmol) of compound **6c**, 93 mg (0.07657 mmol) of compound **11**, 18 mg (0.09188 mmol) sodium ascorbate, 8 mg (0.03063 mmol) copper sulfate pentahydrate in 13.5 ml DMF/water mixture (3/1), followed by the addition of 18 mg (0.06126 mmol) EDTA was isolated individually by reverse phase column chromatography (InterchimPuriflash C18 40 g, 15μ, gradient from 10% acetonitrile to 100% acetonitrile in 30 min, flow rate 20 ml/min) 101 mg (55% yield) of compound **12c** as white powder.

^{1}H NMR (400 MHz, DMSO-d6) δ, ppm: 12.62 (br. s, 3H, COOH), 9.99 (d, J=7.46 Hz, 1H, NH), 9.22 (br. s, 1H, OH), 8.24-8.37 (m, 2H, NH), 8.10-8.16 (m, 3H, NH), 7.84-7.96 (m, 5H, Ar+NH), 7.82 (s, 2H, NH+Triazole), 7.54-7.66 (m, 6H, Ar+NH), 7.10-7.35 (m, 15H, Ar), 7.03 (dd, J=8.41, 3.82 Hz, 2H, Ar), 6.64 (dd, J=8.44, 1.96 Hz, 2H, Ar), 6.23-6.40 (m, 2H, NH), 6.00 (br. s, 1H, NH), 5.41 (br. s, 2H, NH_2), 4.91-5.13 (m, 2H, CH_2), 4.73 (br. s, 1H, CH), 4.18-4.66 (m, 13H, CH+CH_2+CH_3), 4.03-4.18 (m, 4H, CH), 3.89-4.03 (m, 4H, CH+H_2O), 3.13-3.27 (m, 10H, CH_2+CH_3), 2.91-3.10 (m, 12H, CH_2), 2.79-2.91 (m, 5H, CH_2), 2.54-2.79 (m, 6H, CH_2), 2.13-2.39 (m, 12H, CH_2), 2.11 (m, 1H, CH_2), 1.84-2.01 (m, 5H, CH_2), 1.34-1.84 (m, 24H, CH_2+CH_3), 1.10-1.34 (m, 9H, CH_2), 0.93-1.08 (m, 7H, CH_2), 0.64-0.90 (m, 26H, CH_2+CH_3).

^{13}C NMR (101 MHz, DMSO-d6) δ, ppm: 174.50, 174.21, 173.77, 172.53, 172.31, 172.10, 171.71, 171.51, 171.34, 171.10, 170.60, 169.91, 169.85, 168.74, 167.21, 158.96, 157.89, 157.28, 155.88, 146.45, 143.77, 143.69, 137.88, 130.05, 129.78, 129.47, 129.02, 128.19, 128.08, 127.83, 127.77, 127.39, 126.67, 126.47, 126.42, 126.28, 121.96, 118.95, 114.98, 89.66, 74.80, 60.95, 60.31, 58.68, 57.57, 57.17, 54.95, 54.14, 53.15, 52.57, 52.12, 51.65, 46.87, 40.44, 38.71, 37.02, 35.81, 34.68, 32.32, 31.78, 31.54, 31.22, 30.80, 30.48, 29.91, 29.84, 29.35, 29.12, 27.53, 26.65, 26.31, 25.43, 25.37, 24.66, 24.36, 23.14, 22.52, 22.25, 19.28, 18.98, 18.24, 15.49, 15.32, 15.05, 10.33.

HPLC-MS: target compound content – 99.9%, t_R=11.32 min.

ESI-HRMS: for $C_{121}H_{178}N_{22}O_{29}$: m/z calculated for $[M+2H]^{2+}$ 1202.66378, found: 1202.664.

Synthesis of (3*S*,7*S*,25*S*,28*S*,31*S*)-31-(4-aminobutyl)-25,28-dibenzyl-36-(4-(4-(((*S*)-1-(((*S*)-1-((4-((5*S*,8*S*,11*S*,12*R*)-11-((*S*)-*sec*-butyl)-12-(2-((*S*)-2-((1*R*,2*R*)-3-(((1*S*,2*R*)-1-hydroxy-1-phenylpropan-2-yl)amino)-1-methoxy-2-methyl-3-oxopropyl)pyrrolidin-1-yl)-2-oxoethyl)-5,8-diisopropyl-4,10-dimethyl-3,6,9-trioxo-2,13-dioxa-4,7,10-triazatetradecyl)phenyl)amino)-1-oxo-5-ureidopentan-2-yl)amino)-3-methyl-1-oxobutan-2-yl)amino)-4-oxobutyl)-1*H*-1,2,3-triazol-1-yl)-12-(4-carboxybenzyl)-5,13,20,23,26,29,32-heptaoxo-4,6,12,19,24,27,30,33-octaazahexatriacontane-1,3,7-tricarboxylic acid (12d)

From 50 mg (0.04269 mmol) of compound **6d**, 47 mg (0.03881 mmol) of compound **11**, 9 mg (0.04657 mmol) of sodium ascorbate, 4 mg (0.01552 mmol) of copper sulfate pentahydrate in 9.5 ml DMF/water mixture (3/1), followed by addition of 9 mg (0.03105 mmol) EDTA was isolated individually by reverse phase column chromatography (InterchimPuriflash C18 40 g, 15μ, gradient from 10% acetonitrile to 100% acetonitrile in 30 min, flow rate 20 ml/min) 43 mg (45% yield) of compound **12d** as white powder.

^{1}H NMR (400 MHz, DMSO-d6) δ, ppm: 12.45 (br. s, 3H), 9.99 (br. s, 1H, NH), 8.23-8.39 (m, 2H, NH), 8.02-8.23 (m, 2H, NH), 7.79-8.01 (m, 6H, Ar+NH+Triazole), 7.56-7.74 (m, 5H, NH), 7.10-7.38 (m, 16H, Ar), 6.17-6.44 (m, 2H, NH), 6.00 (br. s, 1H, NH), 4.93-5.18 (m, 3H, CH_2), 4.72 (br. s, 1H, CH), 4.24-4.66 (m, 15H, CH+CH_2+CH_3+H_2O), 3.56 (d, *J*=5.20 Hz, 1H,), 3.28-3.58 (m, 4H, CH_2), 3.08-3.26 (m, 10H, CH_3+CH_2), 2.79-3.08 (m, 12H, CH_2), 2.75 (br. s, 2H, CH_2), 2.53-2.61 (m, 2H, CH_2), 2.08-2.40 (m, 10H, CH_2), 1.85-2.00 (m, 4H, CH_2), 1.62-1.84 (m, 7H, CH_2), 1.34-1.62 (m, 12H, CH_2+CH_3), 1.09-1.34 (m, 7H, CH_2).), 0.94-1.08 (m, 5H, CH_2), 0.66-0.92 (m, 19H, CH_2+CH_3).

^{13}C NMR (101 MHz, DMSO-d6) δ, ppm: 174.55, 174.50, 174.21, 173.78, 172.54, 172.09, 171.75, 171.53, 171.33, 170.94, 168.74, 158.95, 157.28, 146.46, 143.70, 137.83, 129.78, 129.46, 129.37, 129.12, 129.01, 128.18, 128.08, 127.83, 127.77, 127.39, 126.76, 126.45, 121.98, 74.81, 57.14, 54.50, 52.60, 51.65, 46.87, 38.71, 35.83, 31.80, 30.55, 29.91, 29.12, 27.55, 26.65, 26.33, 25.44, 24.70, 23.14, 22.27, 19.28, 18.96, 18.24, 15.49, 15.32, 15.06, 10.32.

HPLC-MS: target compound content – 99.9%, t_R=11.46 min.

ESI-HRMS: for $C_{121}H_{178}N_{22}O_{28}$: m/z calculated for $[M+2H]^{2+}$ 1194.66632, found: 1194.6669.

General methodology for producing bimodal conjugates 13a-d

Monoconjugate **12a-d** (1 eq.) was dissolved in DMF, NHS-ester of abiraterone (compound **7**, 1.2 eq.) and DIPEA (6 eq.) were added. The reaction mixture was stirred for 20 hours, after which the solvent was removed under reduced pressure. The dry residue was precipitated with

acetonitrile, the resulting precipitate was decanted and washed three times with acetonitrile. The solvent residue was removed under reduced pressure.

Synthesis of (3*S*,7*S*,25*S*,28*S*,31*S*)-25-benzyl-31-((3-(4-(4-(((*S*)-1-(((*S*)-1-((4-((5*S*,8*S*,11*S*,12*R*)-11-((*S*)-*sec*-butyl)-12-(2-((*S*)-2-((1*R*,2*R*)-3-(((1*S*,2*R*)-1-hydroxy-1-phenylpropan-2-yl)amino)-1-methoxy-2-methyl-3-oxopropyl)pyrrolidin-1-yl)-2-oxoethyl)-5,8-diisopropyl-4,10-dimethyl-3,6,9-trioxo-2,13-dioxa-4,7,10-triazatetradecyl)phenyl)amino)-1-oxo-5-ureidopentan-2-yl)amino)-3-methyl-1-oxobutan-2-yl)amino)-4-oxobutyl)-1*H*-1,2,3-triazol-1-yl)propyl)carbamoyl)-12-(3-chlorobenzyl)-40-(((3*S*,8*R*,9*S*,10*R*,13*S*,14*S*)-10,13-dimethyl-17-(pyridin-3-yl)-2,3,4,7,8,9,10,11,12,13,14,15-dodecahydro-1*H*-cyclopenta[*a*]phenanthren-3-yl)oxy)-28-(4-hydroxybenzyl)-5,13,20,23,26,29,37,40-octaoxo-4,6,12,19,24,27,30,36-octaazatetracontane-1,3,7-tricarboxylic acid (13a)

From 34 mg (0.0142 mmol) of compound **12a**, 9 mg (0.01703 mmol) of compound **7** and 15 μl (0.08514 mmol) of DIPEA, 38 mg (95% yield) of compound **13a** was obtained as white powder.

^{1}H NMR (400 MHz, DMSO-d6) δ, ppm: 12.46 (br. s, 3H, COOH), 9.99 (br. s, 1H, NH), 9.20 (m, 1H, OH), 8.58 (br. s, 1H, Py), 8.42 (m, 1H, Py), 8.33 (d, *J*=6.58 Hz, 1H, Py), 8.03-8.21 (m, 3H, NH), 7.83-8.01 (m, 4H, NH), 7.81 (s, 1H, Triazole), 7.68-7.78 (m, 2H, Ar), 7.51-7.67 (m, 4H, Ar), 7.08-7.40 (m, 18H, Ar), 7.04 (d, *J*=8.50 Hz, 2H, Ar), 6.64 (d, *J*=7.95 Hz, 2H, Ar), 6.23-6.37 (m, 2H, NH), 6.10 (s, 1H, CH), 5.98 (m, 1H, NH), 5.42 (br. s, 2H, NH_2), 5.35 (m, 1H, CH), 4.91-5.15 (m, 2H, CH_2), 4.59-4.77 (m, 1H, CH), 4.16-4.59 (m, 12H, CH_2+CH), 4.03-4.16 (m, 3H, CH), 3.88-4.03 (m, 2H, CH), 3.57 (m, 1H, CH), 3.13-3.26 (m, 9H, CH_2+CH_3), 3.11 (s, 2H, CH_2), 2.90-3.08 (m, 11H, CH_2+CH), 2.77-2.89 (m, 5H, CH_2), 2.60-2.70 (m, 1H, CH_2), 2.53-2.60 (m, 2H, CH_2), 2.43 (m, 2H, CH_2), 2.13-2.40 (m, 7H, CH_2), 1.85-2.13 (m, 4H, CH_2), 1.74-1.84 (m, 2H, CH_2), 1.56-1.74 (m, 4H, CH_2), 1.44-1.56 (m, 4H, CH_2+CH_3), 1.11-1.43 (m, 7H, CH_2+CH_3), 0.92-1.11 (m, 6H, CH_2), 0.67-0.92 (m, 10H, CH_2+CH_3).

^{13}C NMR (101 MHz, DMSO-d6) δ, ppm: 174.50, 174.20, 173.77, 172.38, 172.31, 172.10, 171.79, 171.58, 171.48, 171.34, 171.09, 170.56, 169.90, 169.84, 168.75, 158.92, 157.27, 155.86, 151.01, 147.84, 147.20, 146.40, 143.69, 141.22, 140.81, 139.87, 137.86, 133.33, 133.05, 132.16, 130.59, 130.24, 130.00, 129.01, 128.17, 128.05, 127.94, 127.76, 127.21, 126.75, 126.42, 126.25, 126.08, 124.96, 123.43, 121.98, 121.92, 118.98, 114.98, 85.43, 74.81, 73.17, 60.94, 60.30, 58.67, 58.19, 57.59, 57.14, 56.99, 54.99, 54.17, 53.17, 52.88, 52.17, 51.68, 49.63, 49.18, 47.16, 46.84, 46.66, 46.27, 43.78, 43.22, 38.44, 37.71, 36.42, 36.31, 35.77, 34.67, 34.55, 32.29, 31.87, 31.54, 31.33, 30.96, 30.75, 30.57, 30.45, 29.95, 29.89, 29.36, 29.10, 28.91, 27.80, 27.60, 27.35, 26.84,

26.30, 25.41, 24.66, 24.36, 23.14, 22.91, 22.35, 20.40, 19.27, 18.93, 18.58, 18.24, 16.28, 15.87, 15.48, 15.30, 15.04, 10.43.

HPLC-MS: target compound content – 99%, t_R=8.78 min.

ESI-HRMS: for $C_{148}H_{210}ClN_{23}O_{30}$: m/z calculated for $[M+2Na]^{2+}$ 1435.25435, found: 1435.2559.

Synthesis of (3*S*,7*S*,25*S*,28*S*,31*S*)-25-benzyl-12-(4-bromobenzyl)-31-((3-(4-(4-(((*S*)-1-(((*S*)-1-((4-((5*S*,8*S*,11*S*,12*R*)-11-((*S*)-*sec*-butyl)-12-(2-((*S*)-2-((1*R*,2*R*)-3-(((1*S*,2*R*)-1-hydroxy-1-phenylpropan-2-yl)amino)-1-methoxy-2-methyl-3-oxopropyl)pyrrolidin-1-yl)-2-oxoethyl)-5,8-diisopropyl-4,10-dimethyl-3,6,9-trioxo-2,13-dioxa-4,7,10-triazatetradecyl)phenyl)amino)-1-oxo-5-ureidopentan-2-yl)amino)-3-methyl-1-oxobutan-2-yl)amino)-4-oxobutyl)-1*H*-1,2,3-triazol-1-yl)propyl)carbamoyl)-40-(((3*S*,8*R*,9*S*,10*R*,13*S*,14*S*)-10,13-dimethyl-17-(pyridin-3-yl)-2,3,4,7,8,9,10,11,12,13,14,15-dodecahydro-1*H*-cyclopenta[*a*]phenanthren-3-yl)oxy)-28-(4-hydroxybenzyl)-5,13,20,23,26,29,37,40-octaoxo-4,6,12,19,24,27,30,36-octaazatetracontane-1,3,7-tricarboxylic acid (13b)

From 35 mg (0.01435 mmol) of compound **12b**, 10 mg (0.01722 mmol) of compound **7** and 16 μl (0.0861 mmol) of DIPEA, 33 mg (80% yield) of compound **13b** was obtained as white powder.

^{1}H NMR (400 MHz, DMSO-d6) δ, ppm: 12.40 (br. s, 3H, COOH), 9.98 (br. s, 1H, NH), 9.21 (br. s, 1H, OH), 8.58 (br. s, 1H, Py), 8.39-8.46 (m, 1H, Py), 8.32 (d, J=6.9 Hz, 1H, Ar), 8.10-8.19 (m, 2H, Ar+NH), 7.96-8.10, (m, 1H, NH), 7.82-7.96, (m, 4H, Ar+NH+Py), 7.78-7.82 (s, 1H, Triazole), 7.74 (d, J=8.7 Hz, 1H, Ar), 7.50-7.65 (m, 4H, Ar+NH), 7.47 (d, J=6.1 Hz, 1H, Ar), 7.08-7.41 (m, 16H, Ar), 7.04 (d, J=6.7 Hz, 2H, Ar), 6.64 (d, J=8.3 Hz, 2H, Ar), 6.24-6.36 (m, 2H, NH), 6.10 (br. s, 1H, CH), 5.97 (br. s, 1H, NH), 5.32-5.45 (m, 3H, NH_2+CH), 4.92-5.12 (m, 2H, CH_2), 4.56-4.79 (m, 1H, CH_2), 4.18-4.51 (m, 12H, CH+CH_2), 3.90-4.14 (m, 5H, CH+CH_2), 3.51-3.60 (m, 1H, CH_2), 3.08-3.24 (m, 12H, CH_2), 2.90-3.06 (m, 12H, CH_2+CH_3), 2.80-2.90 (m, 5H, CH_2), 2.52-2.70 (m, 3H, CH_2), 2.15-2.35 (m, 16H, CH_2), 1.85-2.13 (m, 10H, CH_2), 1.74-1.84 (m, 5H, CH_2), 1.63-1.74 (m, 6H, CH_2), 1.55-1.63 (m, 4H, CH_2), 1.44-1.55 (m, 9H, CH_2), 1.29-1.44 (m, 9H, CH_2), 1.12-1.28 (m, 7H, CH_2), 0.94-1.05 (m, 13H, CH_2), 0.70-0.88 (m, 25H, CH_2+CH_3).

^{13}C NMR (101 MHz, DMSO-d6) δ, ppm: 174.53, 174.24, 173.81, 172.38, 172.11, 171.80, 171.60, 171.49, 171.35, 171.12, 170.57, 169.85, 168.76, 158.93, 157.28, 155.87, 151.00, 147.84, 147.19, 146.40, 143.69, 139.87, 137.85, 133.33, 132.14, 131.56, 131.24, 130.01, 129.73, 129.01, 128.66, 128.18, 128.06, 127.94, 127.83, 127.77, 126.74, 126.47, 126.27, 123.45, 121.99, 120.15, 119.93, 118.96, 114.99, 81.64, 74.80, 73.17, 60.95, 60.31, 57.59, 57.17, 56.99, 55.02, 54.17, 53.17, 52.89, 52.13, 51.69, 49.63, 46.83, 46.65, 46.28, 43.78, 43.23, 38.44, 37.71, 36.41, 36.31, 35.83,

34.66, 34.54, 31.80, 31.33, 30.96, 30.45, 29.89, 29.36, 29.10, 28.91, 27.74, 27.62, 27.35, 26.84, 26.32, 25.41, 24.73, 24.65, 24.36, 22.92, 22.36, 20.40, 19.27, 18.94, 18.55, 18.25, 16.28, 15.86, 15.65, 15.48, 15.31, 15.05, 10.32.

HPLC-MS: target compound content – 99.9%, t_R=9.27 min.

ESI-HRMS: for $C_{148}H_{210}BrN_{23}O_{30}$: m/z calculated for $[M+2Na]^{2+}$ 1457.22909, found: 1457.2319.

Synthesis of (3*S*,7*S*,25*S*,28*S*,31*S*)-25-benzyl-31-((3-(4-(4-(((*S*)-1-(((*S*)-1-((4-((5*S*,8*S*,11*S*,12*R*)-11-((*S*)-*sec*-butyl)-12-(2-((*S*)-2-((1*R*,2*R*)-3-(((1*S*,2*R*)-1-hydroxy-1-phenylpropan-2-yl)amino)-1-methoxy-2-methyl-3-oxopropyl)pyrrolidin-1-yl)-2-oxoethyl)-5,8-diisopropyl-4,10-dimethyl-3,6,9-trioxo-2,13-dioxa-4,7,10-triazatetradecyl)phenyl)amino)-1-oxo-5-ureidopentan-2-yl)amino)-3-methyl-1-oxobutan-2-yl)amino)-4-oxobutyl)-1*H*-1,2,3-triazol-1-yl)propyl)carbamoyl)-12-(4-carboxybenzyl)-40-(((3*S*,8*R*,9*S*,10*R*,13*S*,14*S*)-10,13-dimethyl-17-(pyridin-3-yl)-2,3,4,7,8,9,10,11,12,13,14,15-dodecahydro-1*H*-cyclopenta[*a*]phenanthren-3-yl)oxy)-28-(4-hydroxybenzyl)-5,13,20,23,26,29,37,40-octaoxo-4,6,12,19,24,27,30,36-octaazatetracontane-1,3,7-tricarboxylic acid (13c)

From 50 mg (0.02079 mmol) of compound **12c**, 14 mg (0.02495 mmol) of compound **7** and 22 µl (0.1247 mmol) of DIPEA, 49 mg (83% yield) of compound **13c** was obtained as white powder.

^{1}H NMR (400 MHz, DMSO-d6) δ, ppm: 12.47 (br. s, 4H, COOH), 9.98 (br. s, 1H, NH), 9.21 (br. s, 1H, OH), 8.58 (m, 1H, Py), 8.42 (m, 1H, Py), 8.24-8.36 (m, 2H, NH), 8.10-8.17 (m, 2H, NH+Ar), 8.02-8.10 (m, 1H, NH) 7.82-8.01 (m, 5H, Py+Ar+NH), 7.81 (s, 1H, Tetrazole), 7.67-7.77 (m, 2H, Ar+NH), 7.53-7.64 (m, 3H, Ar), 7.22-7.33 (m, 9H, Ar), 7.16-7.22 (m, 2H, Ar), 7.10-7.16 (m, 4H, Ar), 7.00-7.07 (m, 2H, Ar), 6.64 (d, J=8.5 Hz, 2H, Ar), 6.25-6.34 (m, 2H, NH), 6.10 (s, 1H, CH), 5.97 (br. s, 1H, NH), 5.30-5.44 (m, 4H, NH_2+CH_2), 4.91-5.10 (m, 2H, CH), 4.68-4.87 (m, 1H, CH), 6.50-4.61 (m, 2H, CH_2), 4.39-4.50 (m, 3H, CH+CH_2), 4.30-4.39 (m, 3H, CH+CH_2), 4.16-4.30 (m, 5H, CH_2+CH), 4.04-4.14 (m, 3H, CH), 3.89-4.03 (m, 3H, CH), 3.50-3.60 (m, 1H, CH), 3.12-3.24 (m, 11H, CH_2+CH_3), 3.10 (br. s, 2H, CH_2), 2.90-3.07 (m, 13H, CH_2), 2.80-2.89 (m, 5H, CH_2), 2.61-2.69 (m, 1H, CH_2), 2.56 (t, J=7.4 Hz, 2H, CH_2), 2.41-2.46 (m, 3H, CH_2), 2.16-2.34 (m, 16H, CH_2), 1.84-2.07 (m, 10H, CH_2), 1.74-1.83 (m, 5H, CH_2), 1.56-1.74 (m, 11H, CH_2), 1.29-1.56 (m, 10H, CH_2), 1.11-1.29 (m, 8H, CH_2), 0.95-1.05 (m, 14H, CH_2), 0.70-0.89 (m, 25H, CH_2+CH_3).

^{13}C NMR (101 MHz, DMSO-d6) δ, ppm: 174.55, 174.22, 173.79, 172.37, 172.31, 172.12, 171.79, 171.60, 171.49, 171.35, 171.11, 170.57, 169.84, 168.75, 167.22, 158.93, 157.28, 155.86, 151.01, 147.81, 147.16, 146.40, 143.76, 143.69, 139.86, 137.85, 133.30, 130.01, 129.77, 129.46,

129.01, 128.18, 128.06, 127.94, 127.82, 127.77, 127.39, 126.67, 126.47, 126.27, 121.98, 121.92, 118.98, 114.98, 85.42, 74.80, 73.17, 60.95, 60.31, 58.18, 57.60, 57.14, 56.98, 55.07, 53.18, 52.89, 52.15, 51.68, 49.62, 49.18, 46.83, 46.65, 46.27, 43.22, 38.43, 37.71, 36.41, 36.31, 35.86, 34.67, 34.53, 31.82, 31.50, 31.33, 30.96, 30.74, 30.46, 29.94, 29.88, 29.35, 28.90, 27.58, 27.34, 26.84, 26.23, 25.41, 24.65, 24.38, 23.14, 22.91, 22.37, 20.39, 19.27, 18.93, 18.41, 18.25, 16.27, 15.87, 15.65, 15.48, 15.31, 15.05, 10.32.

HPLC-MS: target compound content – 99.9%, t_R=7.53 min.

ESI-HRMS: for $C_{149}H_{211}N_{23}O_{32}$: m/z calculated for $[M+2Na]^{2+}$ 1440.26875, found: 1440.2652.

Synthesis of (3*S*,7*S*,25*S*,28*S*,31*S*)-25,28-dibenzyl-31-((3-(4-(4-(((*S*)-1-(((*S*)-1-((4-((5*S*,8*S*,11*S*,12*R*)-11-((*S*)-*sec*-butyl)-12-(2-((*S*)-2-((1*R*,2*R*)-3-(((1*S*,2*R*)-1-hydroxy-1-phenylpropan-2-yl)amino)-1-methoxy-2-methyl-3-oxopropyl)pyrrolidin-1-yl)-2-oxoethyl)-5,8-diisopropyl-4,10-dimethyl-3,6,9-trioxo-2,13-dioxa-4,7,10-triazatetradecyl)phenyl)amino)-1-oxo-5-ureidopentan-2-yl)amino)-3-methyl-1-oxobutan-2-yl)amino)-4-oxobutyl)-1*H*-1,2,3-triazol-1-yl)propyl)carbamoyl)-12-(4-carboxybenzyl)-40-(((3*S*,8*R*,9*S*,10*R*,13*S*,14*S*)-10,13-dimethyl-17-(pyridin-3-yl)-2,3,4,7,8,9,10,11,12,13,14,15-dodecahydro-1*H*-cyclopenta[*a*]phenanthren-3-yl)oxy)-5,13,20,23,26,29,37,40-octaoxo-4,6,12,19,24,27,30,36-octaazatetracontane-1,3,7-tricarboxylic acid (13d)

From 38 mg (0.0159 mmol) of compound **12d**, 11 mg (0.01909 mmol) of compound **7** and 17 µl (0.0955 mmol) of DIPEA, 35 mg (78% yield) of compound **13d** was obtained as white powder.

^{1}H NMR (400 MHz, DMSO-d6) δ, ppm: 12.53 (br. s, 3H, COOH), 9.98 (br. s, 1H, NH), 8.57 (br. s, 1H, Py), 8.42 (br. s, 1H, Py), 8.05-8.36 (m, 4H, NH), 7.79-8.01 (m, 7H, Ar+Py+Triazole+NH), 7.74 (d, J=7.46 Hz, 2H, NH), 7.53-7.70 (m, 3H, NH+Ar), 7.02-7.40 (m, 21H, Ar), 6.30 (m, 2H, NH), 6.10 (br. s, 1H, CH), 5.98 (br. s, 1H, NH), 5.35-5.42 (m, 3H, NH+CH), 4.93-5.14 (m, 2H, CH_2), 4.72 (br. s, 1H, CH), 4.34-4.66 (m, 8H, CH_2+CH), 4.16-4.33 (m, 5H, CH_2+CH), 3.88-4.16 (m, 5H, CH), 3.14-3.25 (m, 11H, CH_2), 2.79-3.14 (m, 20H, CH_2+CH_3), 2.53-2.70 (m, 4H, CH_2), 2.43 (d, J=5.93 Hz, 2H, CH_2), 1.84-2.40 (m, 29H, CH_2), 1.10-1.84 (m, 43H, CH+CH_2+CH_3), 0.93-1.10 (m, 15H, CH_2), 0.68-0.93 (m, 26H, CH_2+CH_3).

^{13}C NMR (101 MHz, DMSO-d6) δ, ppm: 174.53, 174.23, 173.80, 172.10, 171.80, 171.62, 171.35, 170.96, 170.57, 169.85, 168.74, 156.93, 150.99, 147.18, 146.40, 143.69, 139.86, 137.93, 137.82, 133.34, 129.76, 129.46, 129.08, 129.00, 128.18, 128.07, 127.83, 127.38, 126.68, 126.46, 123.45, 122.00, 121.92, 118.93, 81.75, 77.71, 74.80, 73.17, 60.95, 60.31, 57.59, 57.14, 56.98, 54.99, 54.67, 53.17, 52.92, 49.62, 46.84, 46.64, 46.26, 43.24, 37.70, 36.40, 36.31, 35.79, 34.54,

31.81, 31.54, 31.33, 30.96, 30.46, 29.87, 29.34, 29.12, 28.91, 27.63, 27.34, 26.85, 26.32, 25.42, 24.65, 24.37, 23.14, 22.91, 20.40, 19.27, 18.93, 18.42, 18.25, 16.27, 15.48, 15.31, 15.05, 10.31.

HPLC-MS: target compound content – 96%, t_R=8.41 min.

ESI-HRMS: for $C_{149}H_{211}N_{23}O_{31}$: m/z calculated for $[M+2Na]^{2+}$ 1432.27129, found: 1432.2719.

1.3.4. Synthesis of a bimodal conjugate with the MMAE/Enzalutamide drug pair 15

Synthesis of (7*S*,10*S*,13*S*,31*S*,35*S*)-13-benzyl-7-((3-(4-(4-(((*S*)-1-(((*S*)-1-((4-((5*S*,8*S*,11*S*,12*R*)-11-((*S*)-*sec*-butyl)-12-(2-((*S*)-2-((1*R*,2*R*)-3-(((1S,2*R*)-1-hydroxy-1-phenylpropan-2-yl)amino)-1-methoxy-2-methyl-3-oxopropyl)pyrrolidin-1-yl)-2-oxoethyl)-5,8-diisopropyl-4,10-dimethyl-3,6,9-trioxo-2,13-dioxa-4,7,10-triazatetradecyl)phenyl)amino)-1-oxo-5-ureidopentan-2-yl)amino)-3-methyl-1-oxobutan-2-yl)amino)-4-oxobutyl)-1*H*-1,2,3-triazol-1-yl)propyl)carbamoyl)-26-(3-chlorobenzyl)-1-(4-(3-(4-cyano-3-(trifluoromethyl)phenyl)-5,5-dimethyl-4-oxo-2-thioxoimidazolidin-1-yl)-2-fluorophenyl)-10-(4-hydroxybenzyl)-1,9,12,15,18,25,33-heptaoxo-2,8,11,14,19,26,32,34-octaazaheptatriacontane-31,35,37-tricarboxylic acid (15)

86 mg (0.0359 mmol, 1 equiv.) of compound **12a**, 24 mg (0.0431 mmol, 1.2 equiv.) of compound **14** were dissolved in 20 mL of DMF. To the resulting solution, 38 µl (0.2154 mmol, 6 eq.) of DIPEA was added. The reaction mixture was stirred for 24 h, after which the solvent was removed under reduced pressure. Then the product was precipitated with acetonitrile, decanted and the resulting precipitate was washed with acetonitrile. The target product was isolated individually by reverse phase column chromatography using a mixture of acetonitrile and water (InterchimPuriflash C18 20 g, 15µ, gradient from 10% acetonitrile to 100% acetonitrile in 30 min, flow rate 20 ml/min) as eluent. A 20 mg (20 % yield) of compound **15** as white powder was obtained.

^{1}H NMR (400 MHz, DMSO-d6) δ, ppm: 12.49 (br. s., 3H, COOH), 9.99 (br. s., 1H, NH), 9.20 (br. s, 1H, OH), 8.35-8.62 (m, 1H, NH), 8.28 (br. s., 2H, NH), 8.02-8.20 (m, 3H, Ar+NH), 7.70-7.98 (m, 6H, Ar), 7.49-7.69 (m, 3H, Ar), 6.94-7.45 (m, 21H, Ar), 6.63 (br. s., 2H, Ar), 6.29 (br. s., 2H, NH), 5.97 (br. s., 1H, NH), 5.29-5.47 (m, 2H, NH), 5.03 (br. s., 1H, CH_2), 4.72 (br. s., 1H, CH), 4.18-4.65 (m, 13H, CH_2+CH), 3.87-4.15 (m, 6H, CH_2+CH), 3.50-3.80 (m, 3H, CH_2+CH), 3.08-3.26 (m, 14H, CH_2), 2.75-3.07 (m, 18H, CH_2), 2.62-2.74 (m, 3H, CH_2), 2.03-2.36 (m, 16H, CH_2), 1.90 (m, 6H, CH_2), 1.63-1.83 (m, 10H, CH_2), 1.09-1.61 (m, 28H, CH_2+CH_3), 0.99 (d, *J*=5.14 Hz, 8H, CH_2+CH_3), 0.64-0.89 (m, 26H, CH_2+CH_3).

HPLC-MS: target compound content – 99.9%, t_R=9.20 min.

ESI-HRMS: for $C_{140}H_{188}ClF_4N_{25}O_{29}S$: m/z calculated for $[M+2Na]^{2+}$ 1436.15673, found: 1436.1587.

2. *In vitro* evaluation of compounds 13a-d and Combo on PC-3 cell line

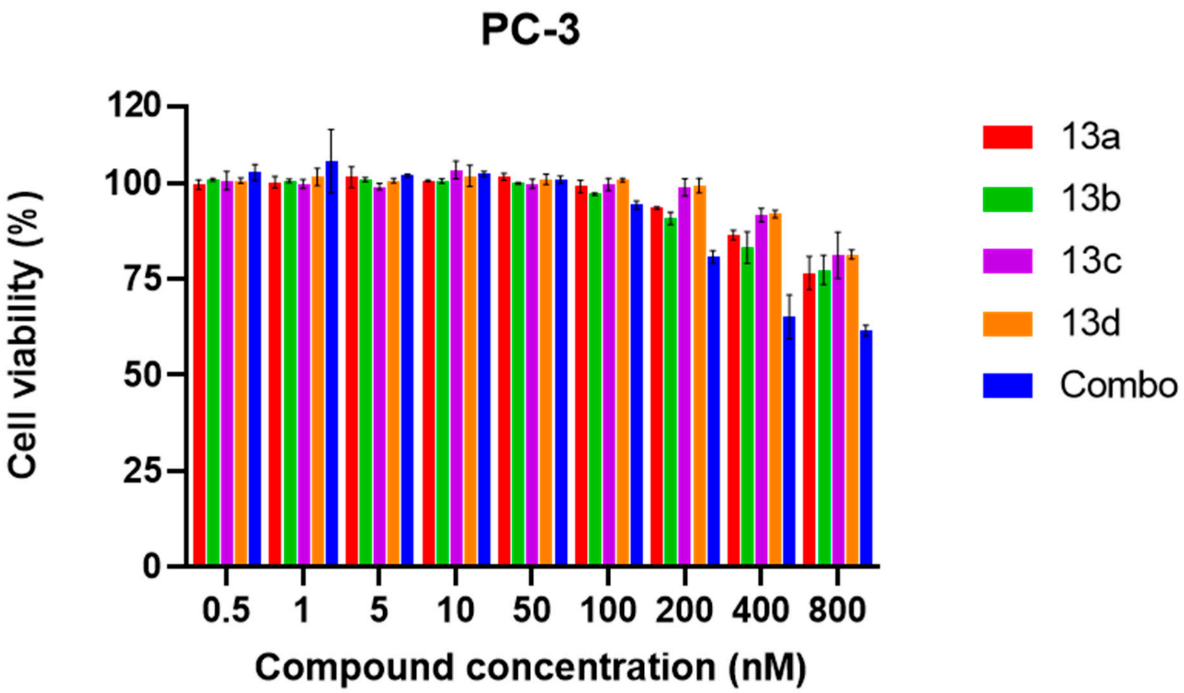

Figure S5. Graphical representation of the results of the *in vitro* experiment on PC-3 prostate cancer cell line.

3. *In vivo* evaluation of synthesized conjugates

3.1. Characteristics of the test conjugates and control substances

Substances:

- **I**, concentration 0.221 µmole/ml; 0.5 mg/ml;
- **13a**, concentration 0.221 µmole/ml; 0.623 mg/ml;
- **15**, concentration 0.221 µmole/ml; 0.624 mg/ml
- **MMAE**, concentration 0.221 µmole/ml; 0.158 mg/ml.

All tested compounds were stored in the freezer at minus 20 ± 2ºC.

An isotonic (0.9%) sodium chloride solution for injection in ampoules was used as a control substance (negative control).

3.2. Immunohistochemical Studies

Tissue samples obtained by the autopsy of animals on 14–21 days after cell inoculation were fixed in neutral buffered 10% formalin and encased in paraffin after a standard histological examination. Serial tissue sections with a 4 µm thickness were prepared. For histological analysis, the sections were stained with hematoxylin and eosin (H&E) according to the standard technique. Polyclonal rabbit antibodies Ab58779 (Abcam, United Kingdom) were used to detect PSMA expression at a dilution of 1:100. Sections were stained using a common indirect immunoperoxidase assay technique. To demask the antigen, glasses with deparaffinized sections

were incubated in 0.1 M sodium citrate buffer (pH 6.0) at 95 °C for 20 min. The system of secondary reagents included biotinylated polyclonal antibodies to rabbit immunoglobulins (Santa Cruz Biotechnology, Inc.), streptavidin conjugate with horseradish peroxidase (Dako, Denmark), and the chromogenic substrate Liquid DAB+ Substrate Chromogen System (Dako, Denmark). Nonspecific rabbit immunoglobulins (Santa Cruz Biotechnology, Inc.) were applied to control sections instead of primary antibodies. After the completion of the reaction, cells and sections were stained with hematoxylin and encapsulated in Canada balsam. Micropreparations were analyzed under an Olympus BX51 microscope equipped with an image documentation system.

3.2.1. *In vivo* evaluation of antitumor efficacy

To study the antitumor efficacy of conjugates **13a**, **15**, **I** and **MMAE** were administered intravenously at a dose of 132.3 nM/kg to tumor-bearing mice three times at 5-day intervals, the administration was started 7-8 days after cell inoculation to animals, tumor size was 70-90 mm^3. A 0.9% sodium chloride solution was used as a control substance. The experimental groups consisted of 4 animals.

The presence of tumor masses and their size, as well as the body weight of the mice, were recorded every 5 days during observation. In accordance with humane principles, animals were euthanized by placing the mice in a CO_2 inhalation chamber (ZOONLAB GmbH, Germany).

The design of the **I**, **13a**, **15** and **MMAE** antitumor efficacy studies is shown in Table S1.

Table S1. Design study of the efficacy of **I**, **13a**, **15** and **MMAE** in 22Rv1 and PC-3 prostate cancer xenograft mice.

**Conjugate**	**Method of injection**	**Dose**	**Number of animals**	
			**22Rv1**	**PC-3**
**I**	Intravenous	132.3 nM/kg, three times	4	4
**13a**	Intravenous		4	4
**15**	Intravenous		4	4
**MMAE**	Intravenous		4	4
**Control (0.9% NaCl)**	Intravenous		4+3	4+3
				**Total: 46 mice**

3.3. Tumor Histology

The proliferation rates of 22Rv1 and PC-3 cells when cultured in vitro were similar, which was also confirmed by the subcutaneous inoculation of nude mice. A 100% yield of 22Rv1 and PC-3 xenograft tumors was achieved when 5 × 106 cells of tumor material were inoculated into animals. According to the results of the histological examination, the subcutaneous xenografts of 22Rv1 and PC-3 tumors had a solid glandular structure, and a large number of mitoses were

determined in the tumor tissues (Figure S6 A and B; Figure S7 A and B, respectively). Both tumors were well vascularized. At 21 days, the tissue of the 22Rv1 tumor showed foci of necrosis. There was an inflammatory infiltrate in the surrounding connective tissue of both tumors.

According to the results of the immunohistological reaction evaluation, PSMA expression was detected in both 22Rv1 (Figure S6, C) and PC-3 (Figure S7, C) subcutaneous prostate cancer xenograft tumors. In 22Rv1 and PC-3 cells, both cytoplasm staining and surface membrane staining were observed. It should be noted that the total staining intensity of the 22Rv1 xenografts in all cases studied was significantly higher than that of the PC-3 xenografts; thus, the level of PSMA expression in PC-3 cells is much lower than that in 22Rv1 cells.

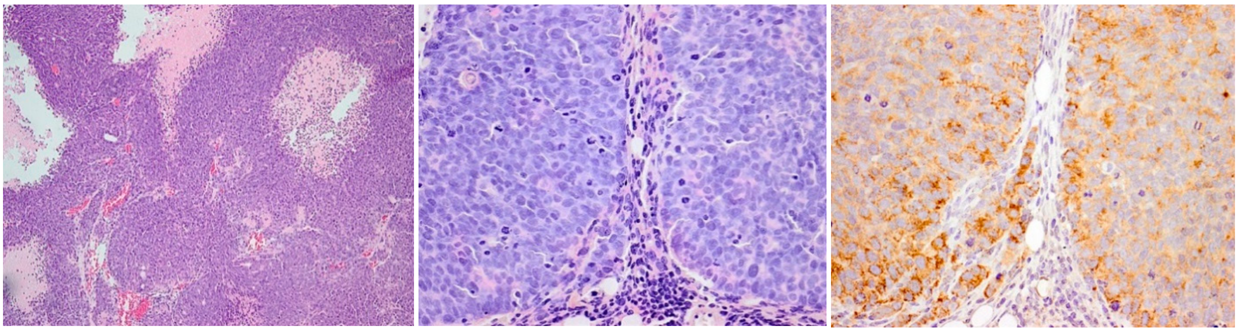

(A) (B) (C)

Figure S6. Histological preparations of a subcutaneous xenograft of human prostate carcinoma 22Rv1 in nude mice. Taken on day 21 after the tumor cell transplantation procedure. Microphotographs of formalin-fixed paraffin sections of tissue: (A) The general morphological picture (H&E) with x100 zoom; (B) The general morphological picture (H&E) with x400 zoom; (C) the expression of PSMA in tumor cells, the immunoperoxidase reaction with antibodies to PSMA (Ab58779, dilution 1:100), and (c) additional staining with hematoxylin are shown.

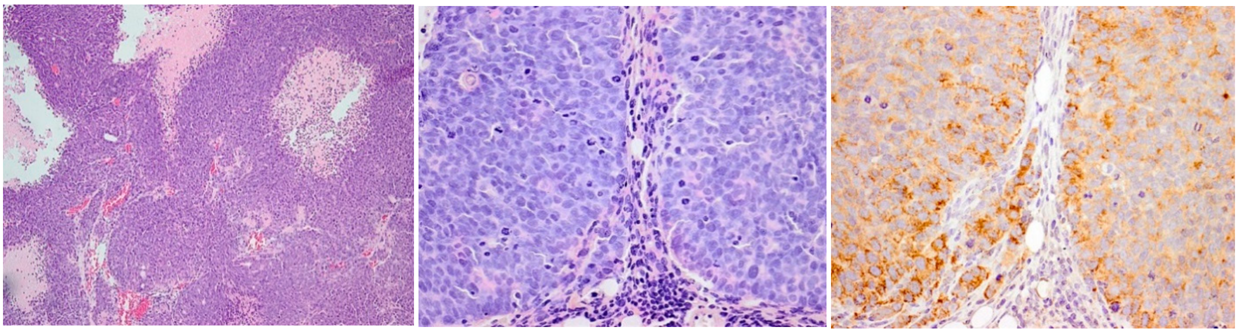

(A) (B) (C)

Figure S7. Histological preparations of a subcutaneous xenograft of human prostate adenocarcinoma PC-3 in nude mice. Taken on day 21 after the tumor cell transplantation procedure. Microphotographs of formalin-fixed paraffin sections of tissue: (A) The general morphological picture (H&E) with x100 zoom; (B) The general morphological picture (H&E) with x400 zoom; (C) the expression of PSMA in tumor cells, the immunoperoxidase reaction with

antibodies to PSMA (Ab58779, dilution 1:100), and (c) additional staining with hematoxylin are shown.

3.4. *In vivo* Efficacy Evaluation

Three times administration of the substances at a dose of 132.3 nM/kg had no effect on the general condition and weight of the animals in all groups, and no death of mice was observed. Data on the body weight of the animals are presented in Table S2.

Table S2. Body weight of mice in a study of the antitumor efficacy of **13a**, **15**, **I** conjugates and **MMAE**.

Conjugate	Days after tumor implantation / mass± error, g						
	7 day (1st injection)	12 day (2nd injection)	17 day (3rd injection)	22 day	27 day	33 day	37 day
Human prostate carcinoma 22Rv1							
**13a**	22.3±1.3	22.5±1.4	22.2±1.4	22.2±1.4	22.5±1.3	22.7±1.3	22.7±1.3
**15**	25.2±1.2	25.0±1.2	25.0±1.2	25.2±1.3	25.1±1.3	25.2±1.3	25.5±1.5
**I**	23.4±0.8	23.9±1.2	23.5±1.7	23.4±1.5	23.2±1.4	23.2±1.6	23.4±1.5
**MMAE**	24.0±1.3	23.7±1.3	23.4±1.3	23.5±1.3	23.8±1.3	24.0±1.3	24.2±1.4
0.9% NaCl	25.5±2.1	25.7±1.7	26.0±1.7	26.3±1.5	26.5±1.5	26.9±1.4	26.9±1.4
Human prostate adenocarcinoma PC-3							
**13a**	24.9±1.2	24.7±1.5	24.8±1.4	25.0±1.4	25.1±1.3	25.2±1.2	25.3±1.2
**15**	23.9±2.1	23.8±2.1	24.0±2.2	24.4±2.3	24.8±2.1	24.9±1.8	25.1±1.9
**I**	22.4±2.1	22.8±1.8	22.4±1.6	22.8±1.4	23.0±1.3	23.2±1.4	23.1±1.3
**MMAE**	22.4±1.2	22.7±1.5	22.4±1.4	23.0±1.4	23.1±1.3	23.2±1.2	23.3±1.2
0.9% NaCl	24.1±1.2	24.7±1.5	24.8±1.4	25.0±1.4	25.1±1.3	25.2±1.2	25.5±1.2

Table S3. Tumor growth dynamics of 22Rv1 and PC-3 in mice with intravenous triple injection of **13a**, **15**, **I** and **MMAE** (single dose 132.3 nM/kg).

**Conjugate**	**Days after tumor implantation / V±m, mm^3**						
	**7 day (1st injection)**	**12 day (2nd injection)**	**17 day (3rd injection)**	**22 day**	**27 day**	**33 day**	**37 day**
Human prostate carcinoma 22Rv1							
**13a**	69.6±17.0	77.2±9.3	81.4±25.8	121.7±58.5	215.9±74.3	391.6±112.4	784.0±201.9
**15**	73.3±8.8	64.7±15.3	76.1±46.5	171.6±85.6	315.5±108.6	650.1±199.9	988.7±225.8
**I**	78.3±16.3	85.4±25.5	92.6±34.8	147.7±56.3	227.7±98.3	419.5±112.6	824.4±145.3
**MMAE**	73.7±14.3	93.6±5.7	125.3±28.8	211.7±64.5	388.2±103.6	749.3±134.3	1402.3±313.3
**0.9% NaCl**	73.4±15.5	129.3±21.8	214.4±42.6	530.2±77.1	1100.7±128.6	2023.7±381.9	2859.3±451.3
Human prostate adenocarcinoma PC-3							
**13a**	82.2±5.9	127.5±48.2	192.2±72.2	292.5±92.8	463.1±135.4	958.0±212.3	1684.0 ±380.1
**15**	83.1±6.8	117.6±16.2	239.5±60.3	397.0±98.4	534.3±113.8	819.8±198.7	1318.4±300.3
**I**	80.3±4.5	92.6±14.8	128.9±19.5	271.7±42.6	641.6±91.7	1059.1±152.2	1855.5±236.6

**MMAE**	85.9±8.7	117.9±29.4	211.3±59.6	330.9±104.5	592.6±116.2	1014.3±137.2	1679.9±215.0
**0.9% NaCl**	88.0±7.1	170.7±25.3	301.1±36.2	487.2±162.6	840.8±294.5	1359.4±311.1	2728.7±309.4

Table S4. Antitumor efficacy of **I**, **13a**, **15** and **MMAE** in 22Rv1 and PC-3 xenograft mice.

Tumor growth inhibition, %					
Conjugate	Days after the start of treatment				
	1	5	10	16	20
Human prostate carcinoma 22Rv1					
**I**	77.4	78.8	84.5	80.1	71.0
**13a**	62.0	77.1	80.4	80.6	72.6
**15**	64.5	67.6	71.3	67.9	65.4
**MMAE**	41.5	60.1	64.7	63.0	51.0
Human prostate adenocarcinoma PC-3					
**I**	28.5	37.3	37.7	37.2	21.1
**13a**	36.2	40.0	44.9	29.5	38.3
**15**	20.5	18.5	36.5	39.7	51.7
**MMAE**	29.8	32.1	29.5	25.4	38.4

4. Spectral data of the synthesised compounds

^{1}H NMR spectra for compound **4a**

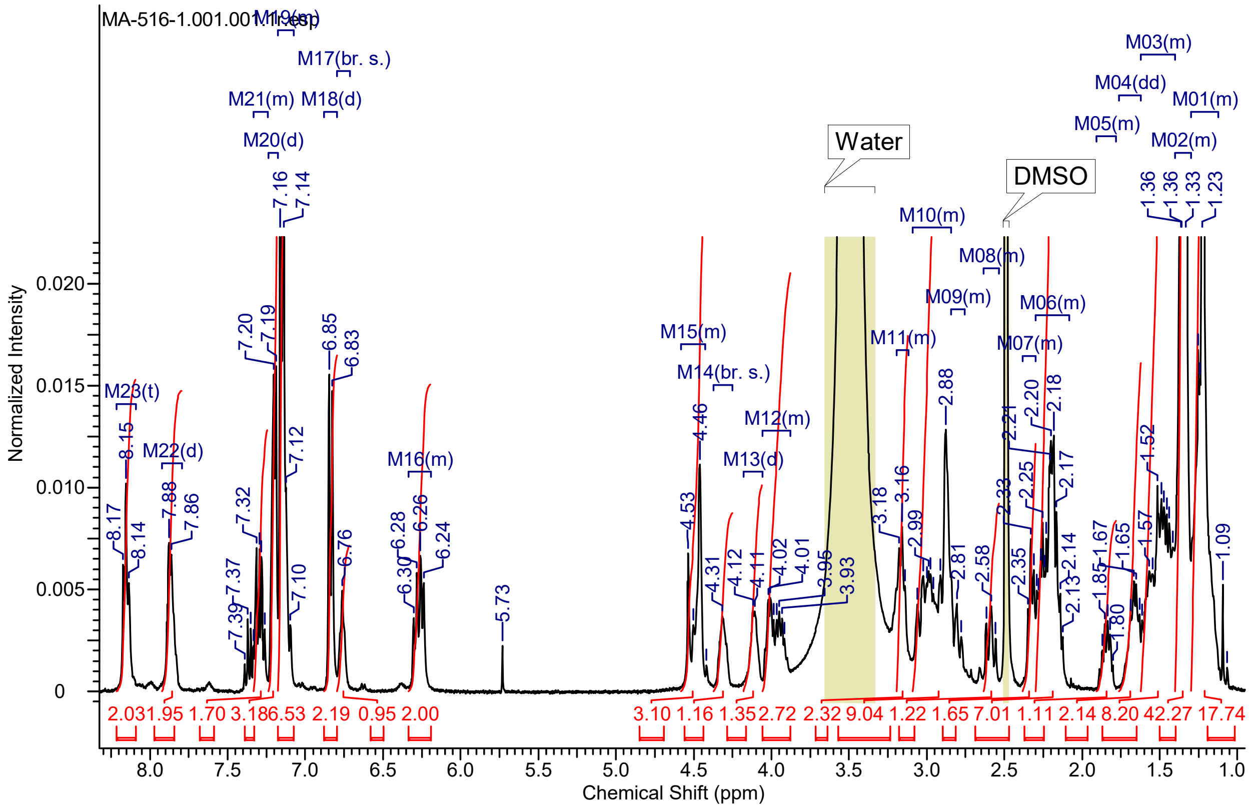

^{1}H NMR spectra for compound **4b**

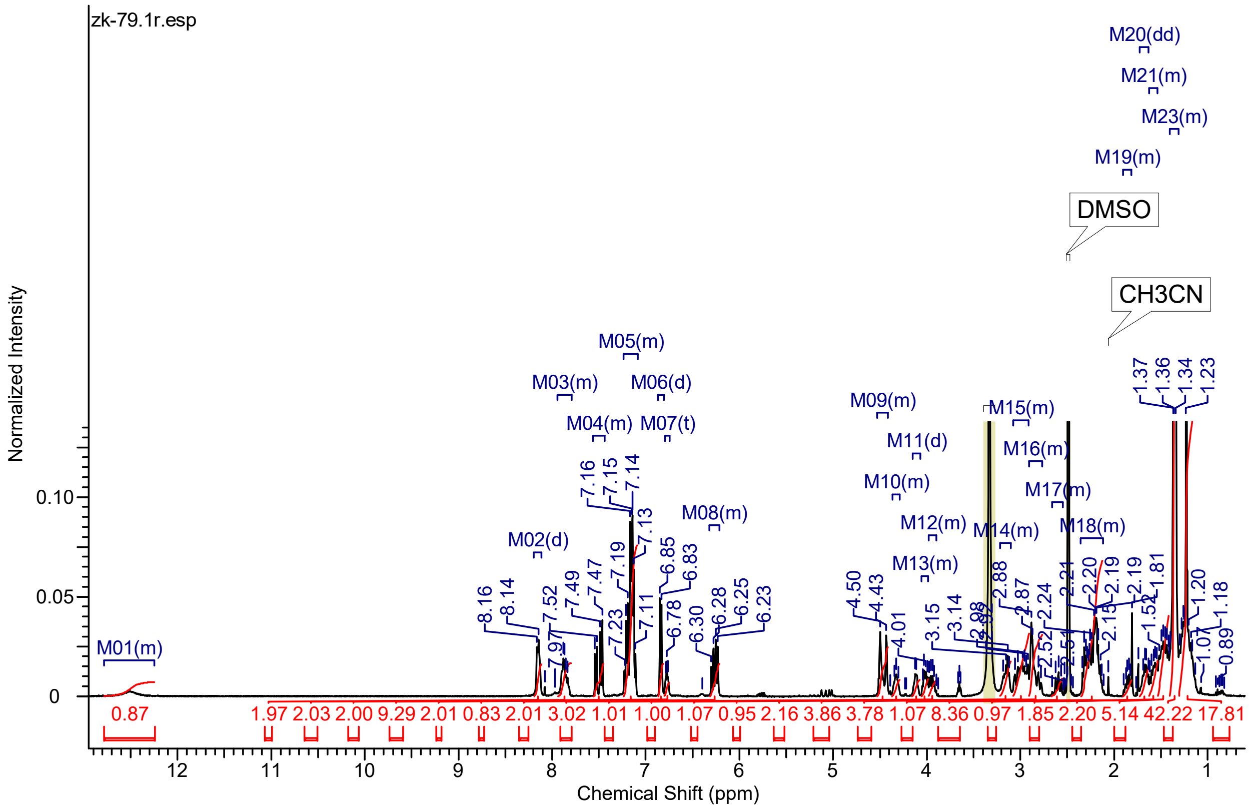

^{13}C NMR spectra for compound **4b**

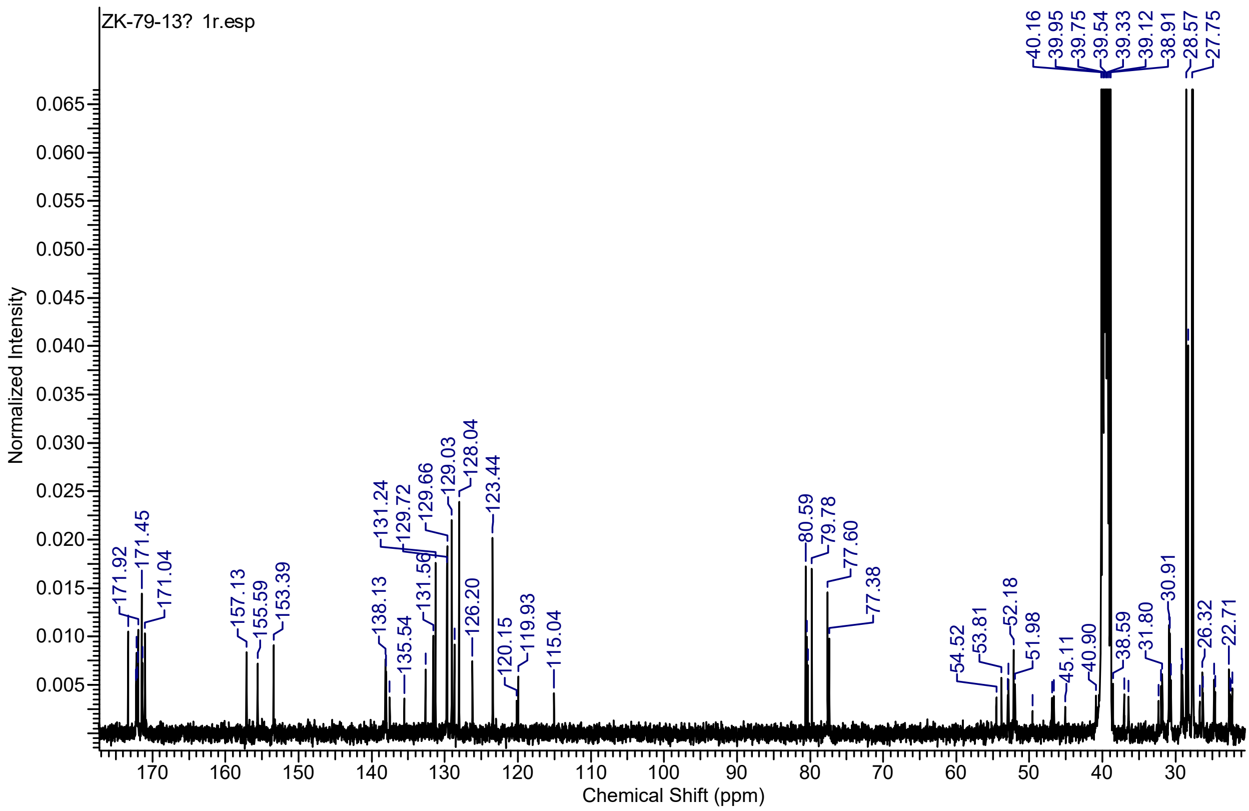

^{1}H NMR spectra for compound **4c**

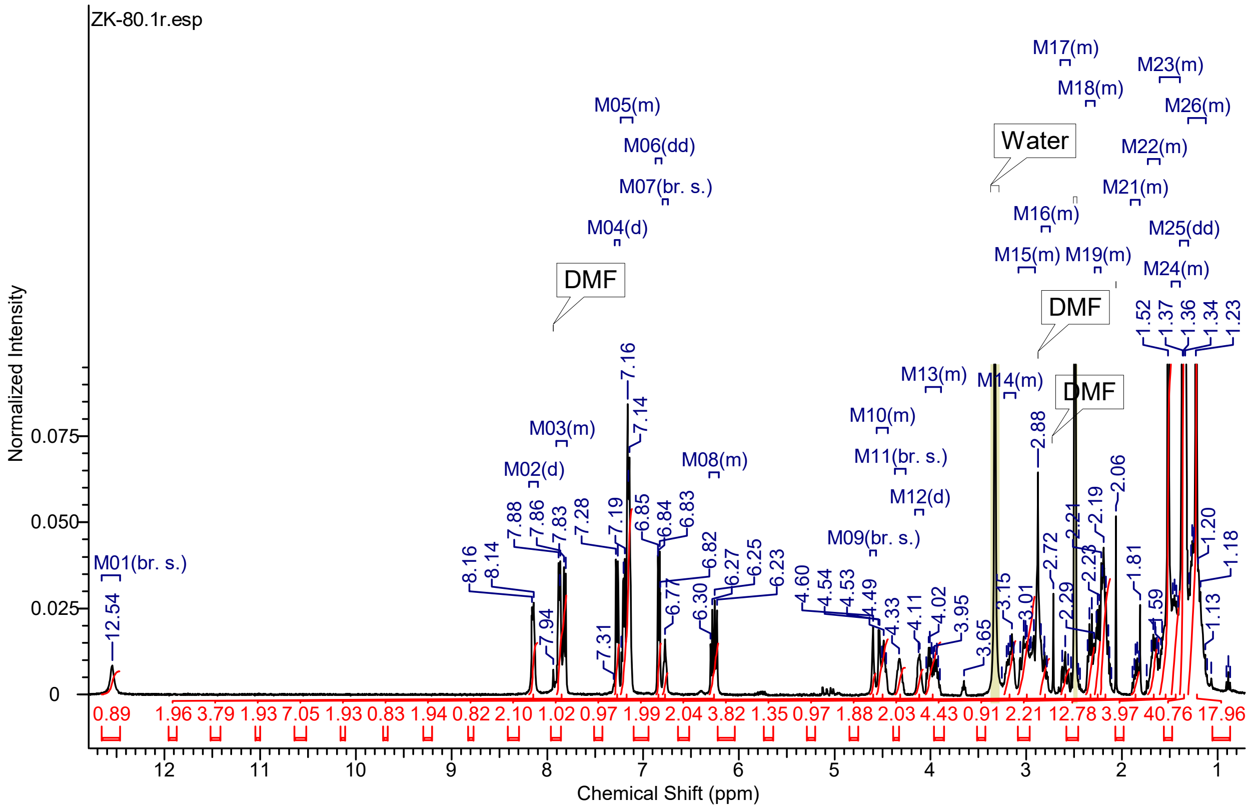

^{13}C NMR spectra for compound **4c**

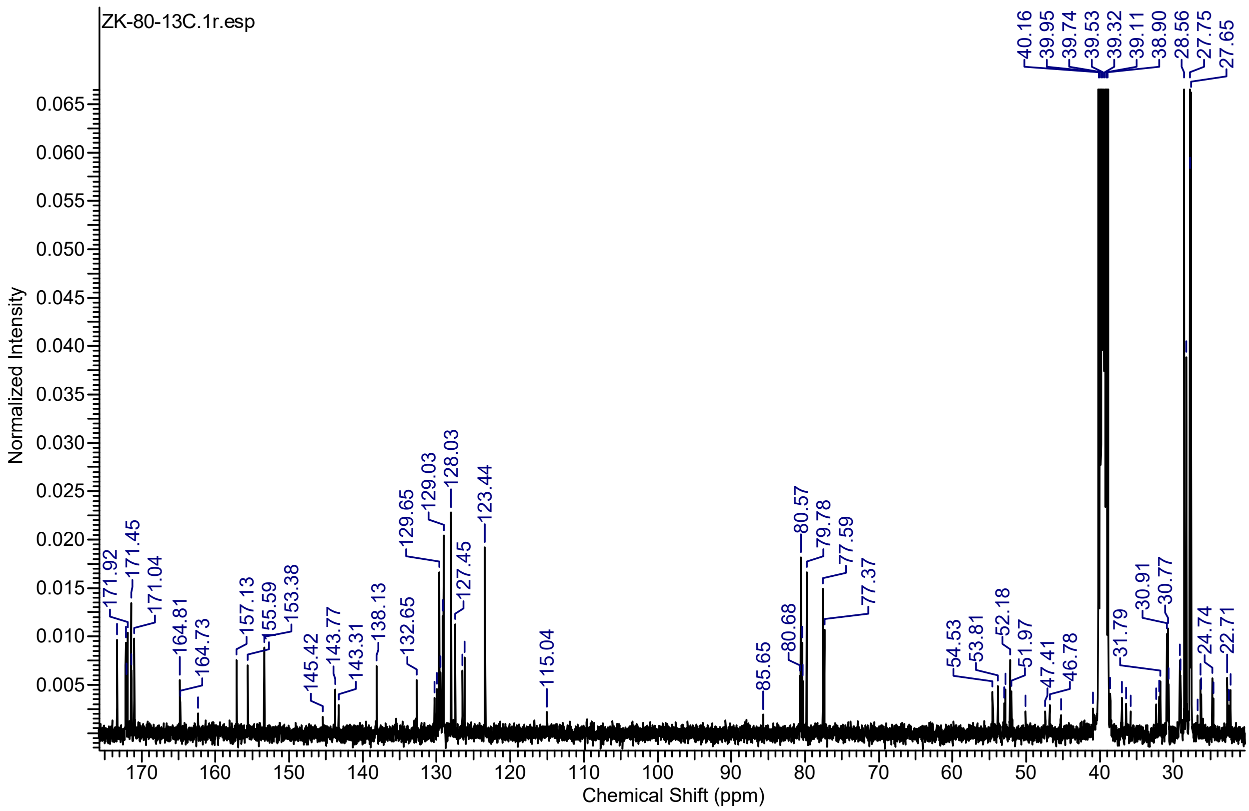

^{1}H NMR spectra for compound **4d**

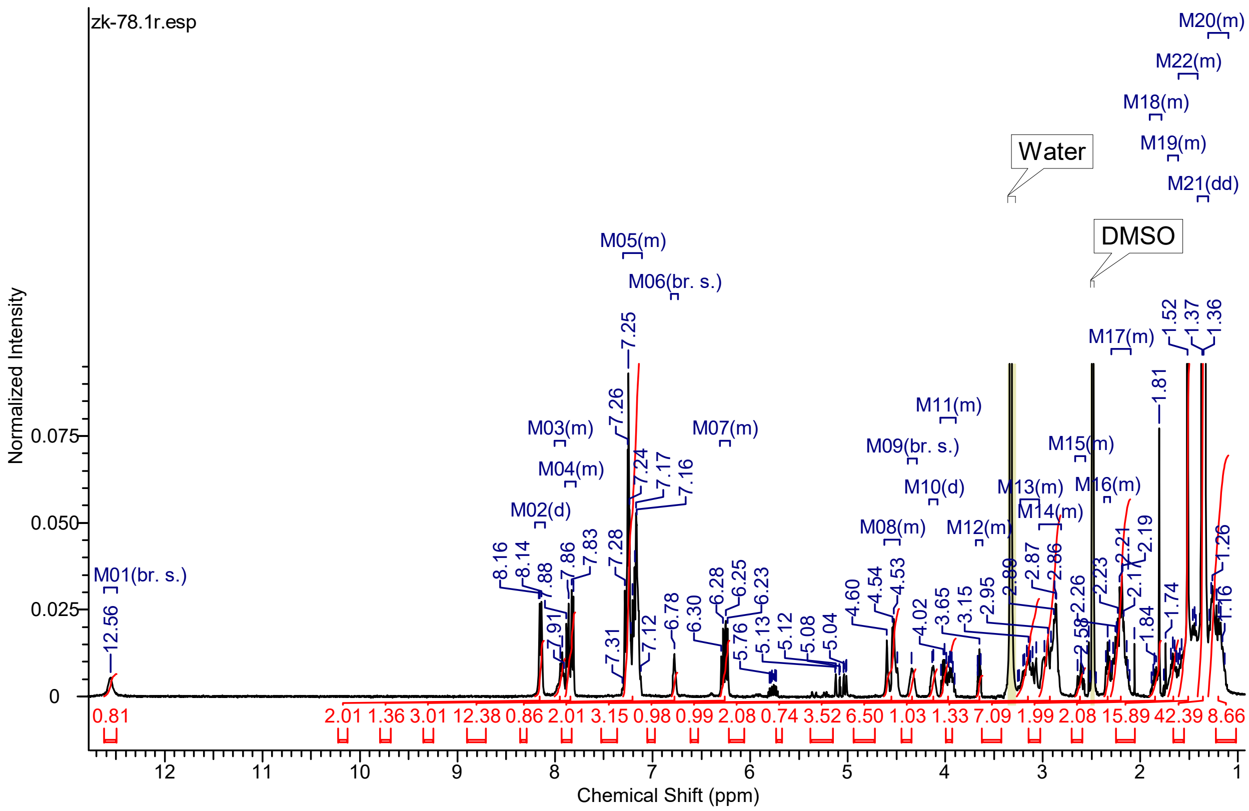

^{13}C NMR spectra for compound **4d**

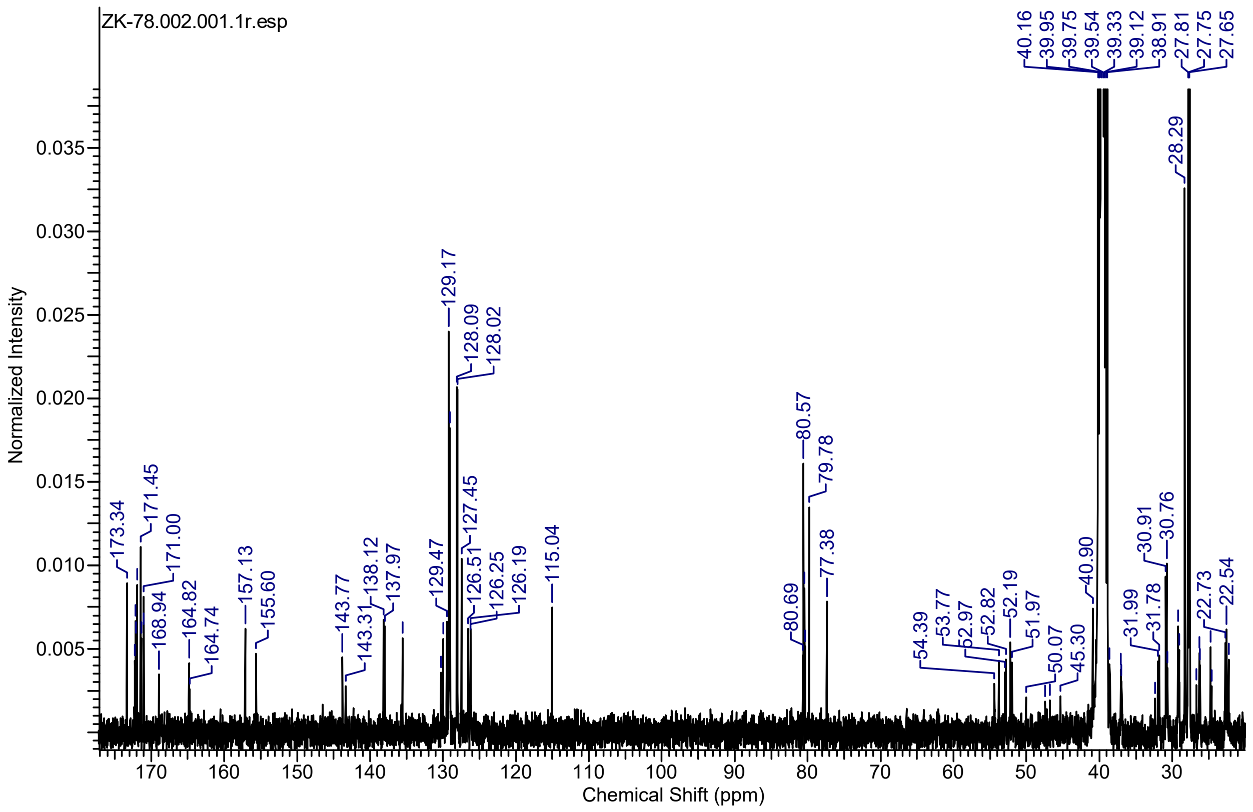

^{1}H NMR spectra for compound **5a**

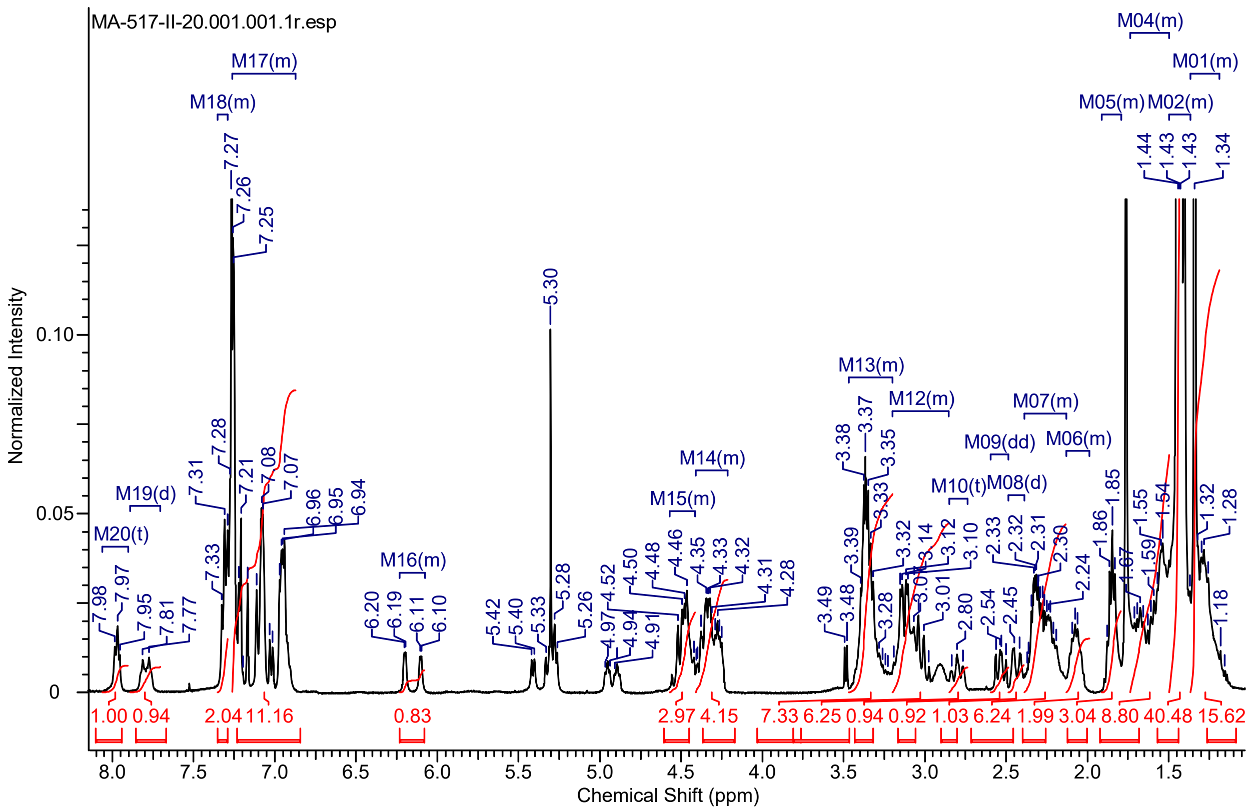

^{13}C NMR spectra for compound **5a**

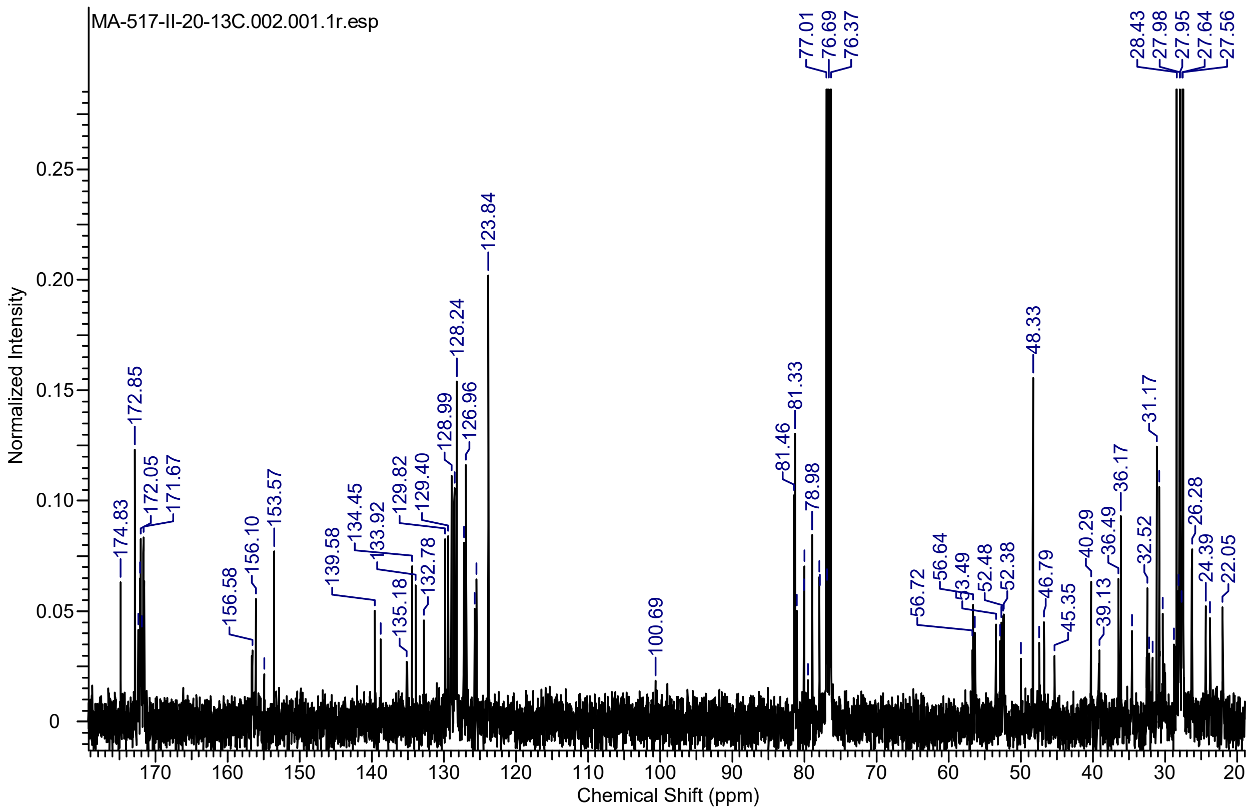

^{1}H NMR spectra for compound **5b**

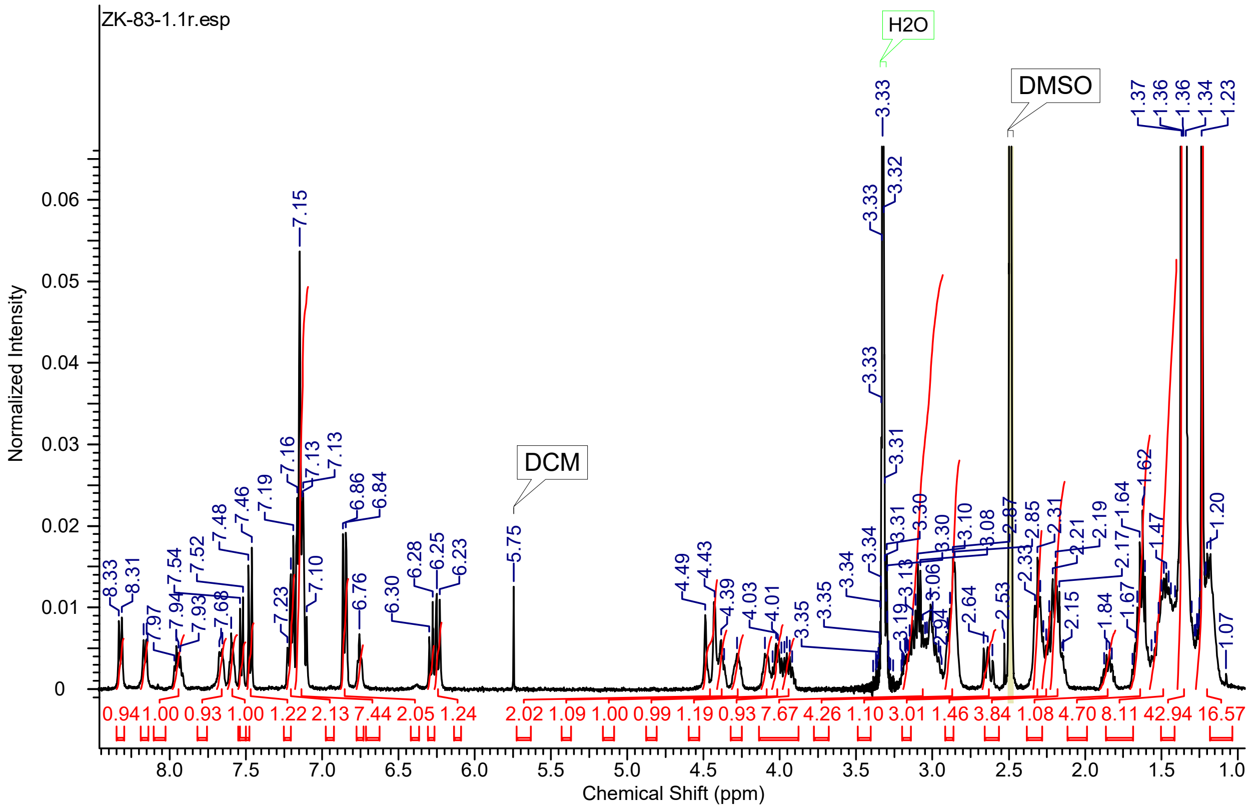

^{13}C NMR spectra for compound **5b**

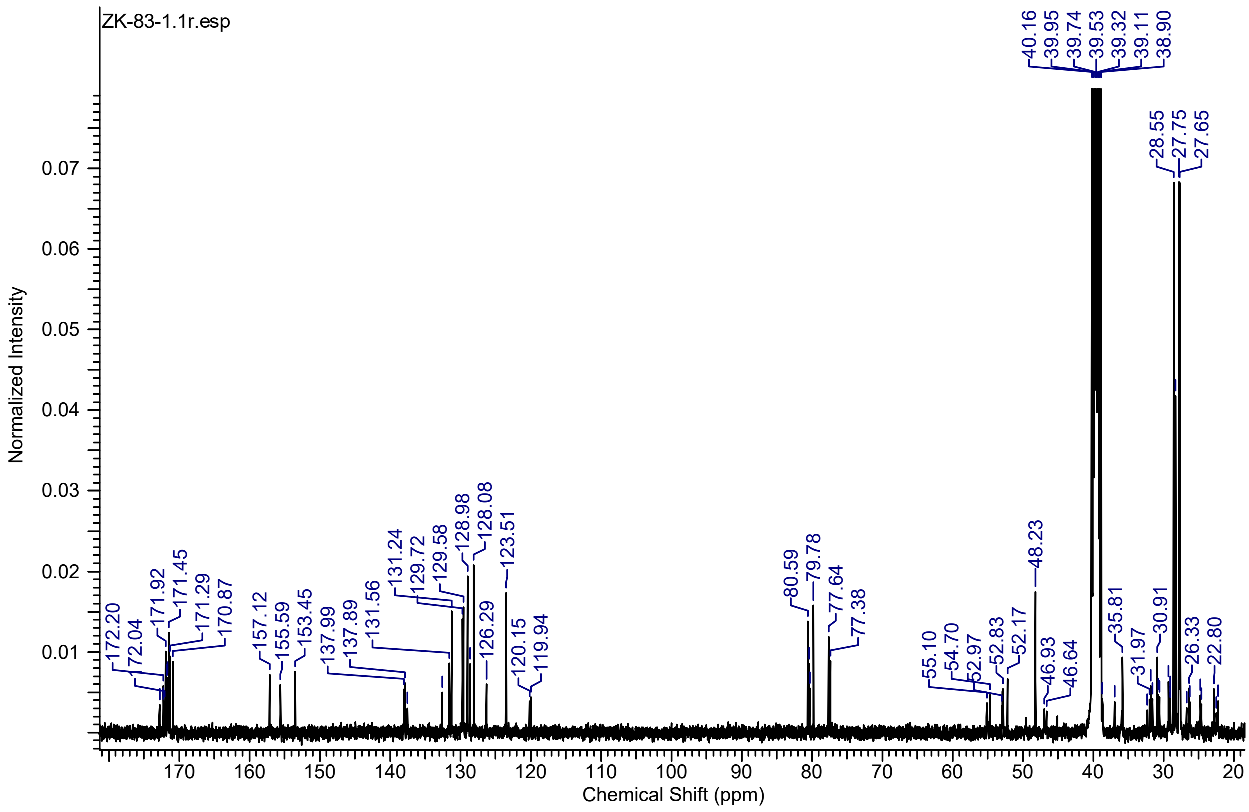

^{1}H NMR spectra for compound **5c**

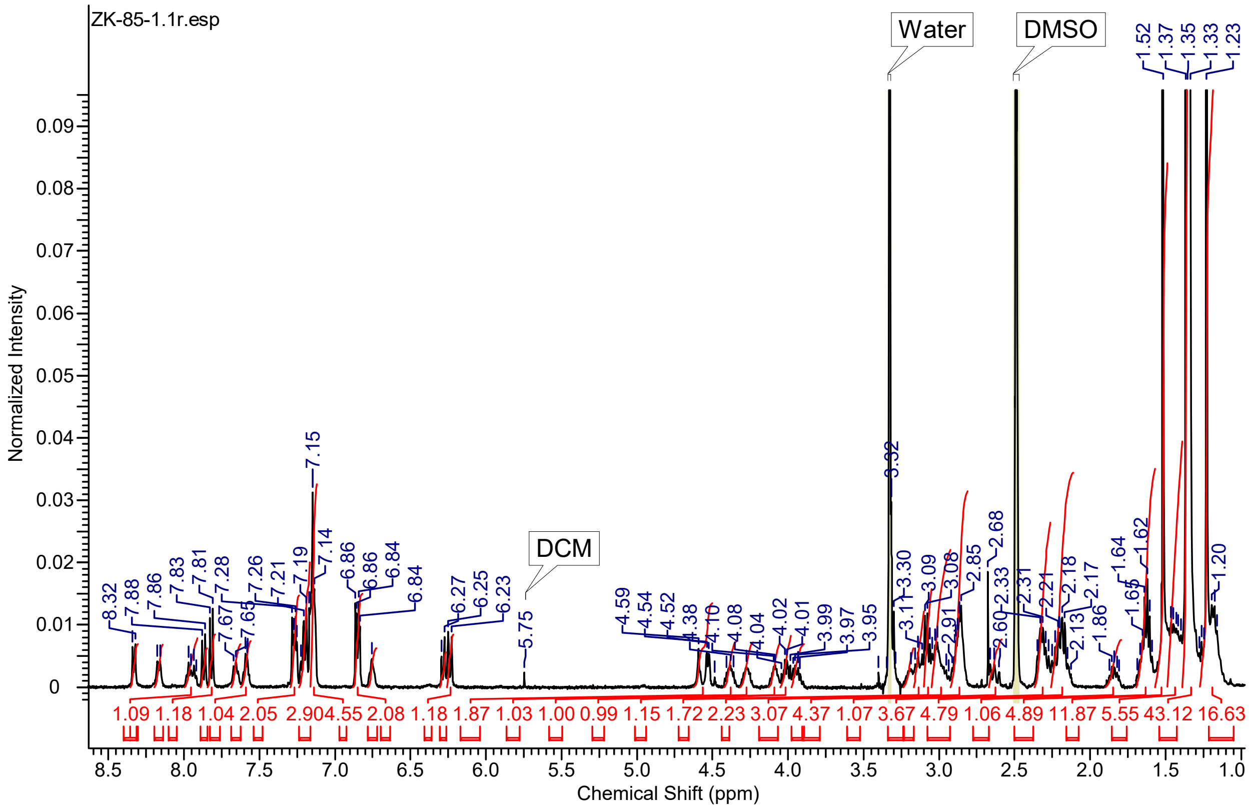

^{13}C NMR spectra for compound **5c**

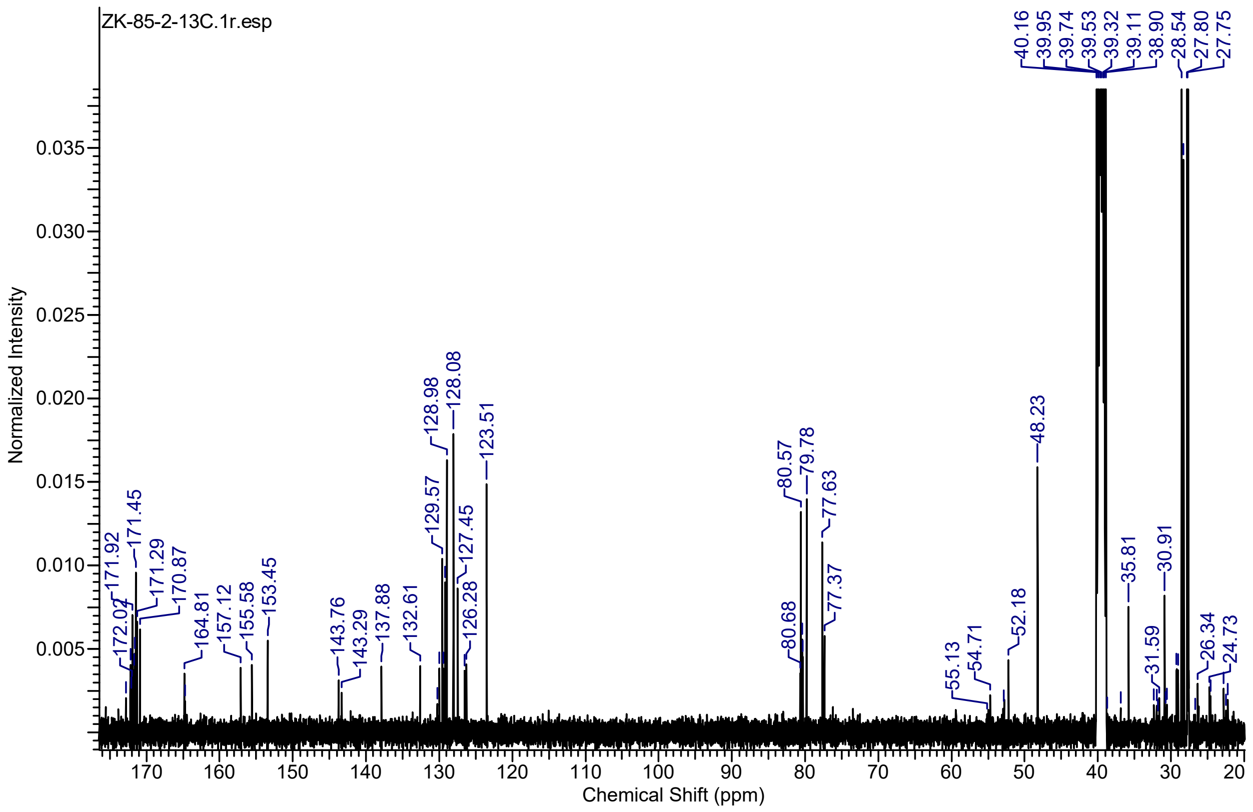

^{1}H NMR spectra for compound **5d**

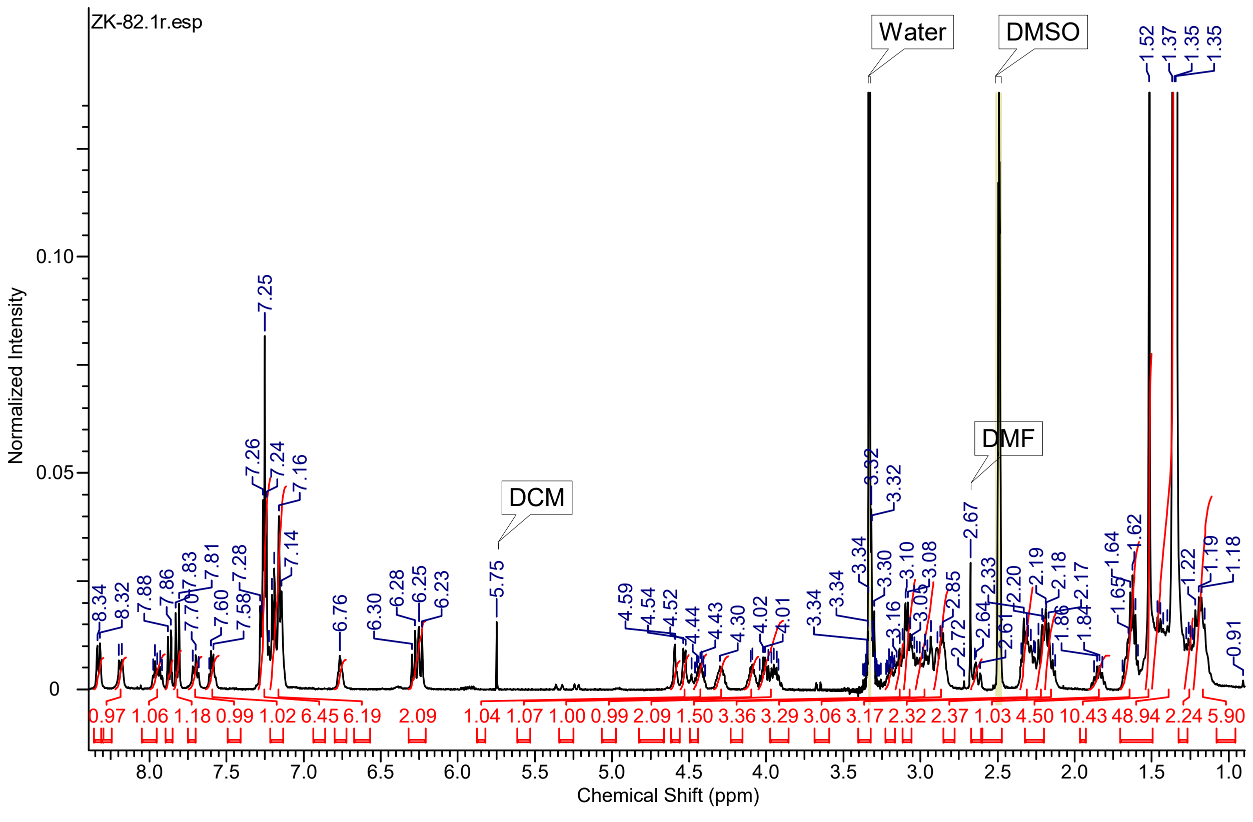

^{13}C NMR spectra for compound **5d**

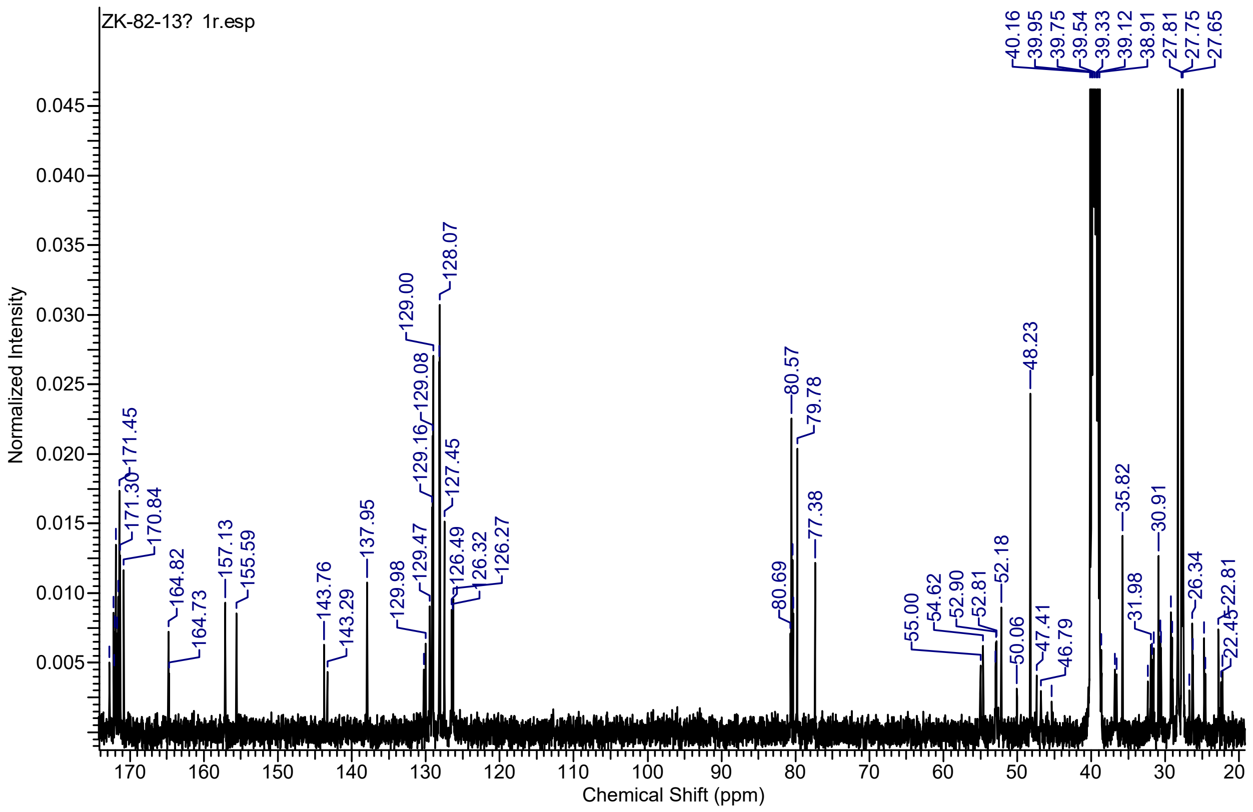

^{1}H NMR spectra for compound **6a**

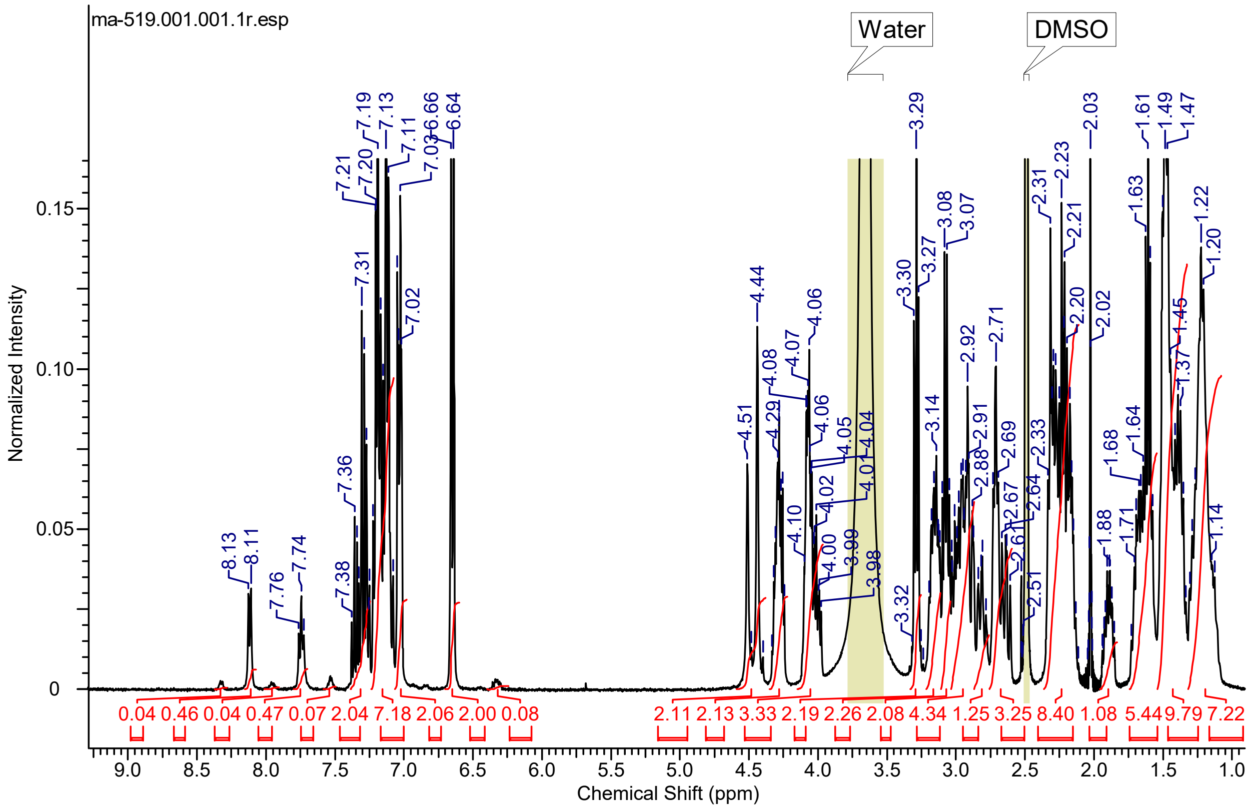

^{13}C NMR spectra for compound **6a**

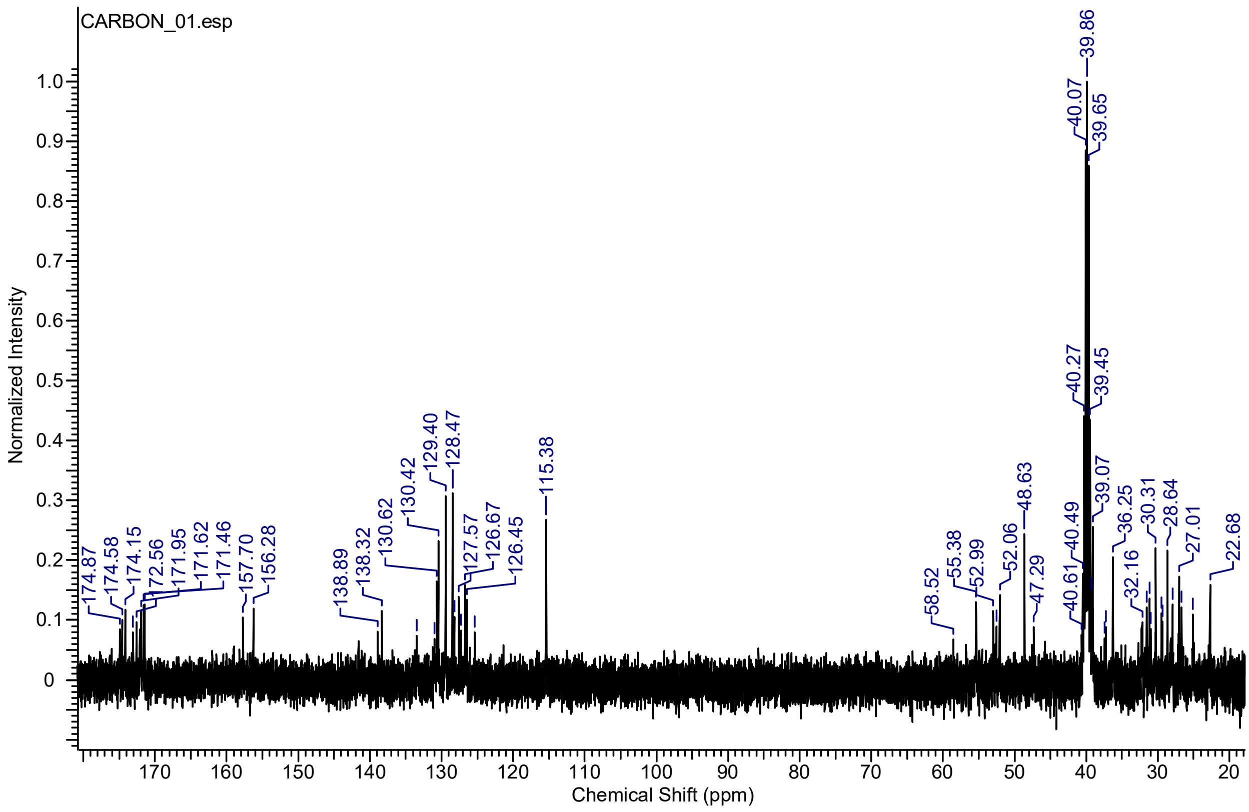

ESI-HRMS for compound **6a**

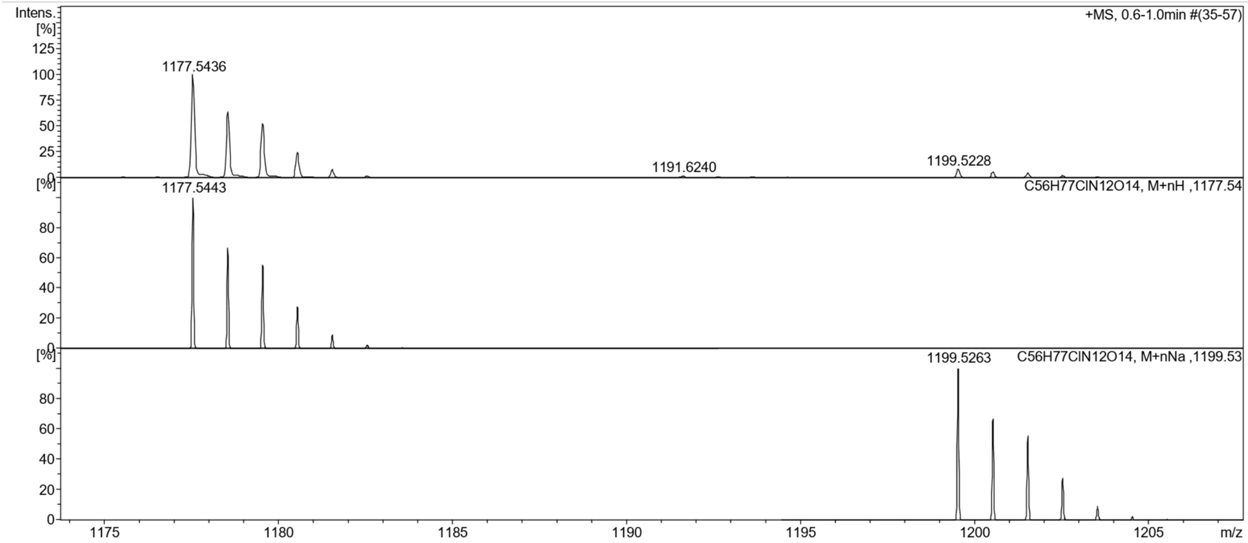

HPLC chromatogram for compound **6a**

Positive ions (chromatogram and blank)

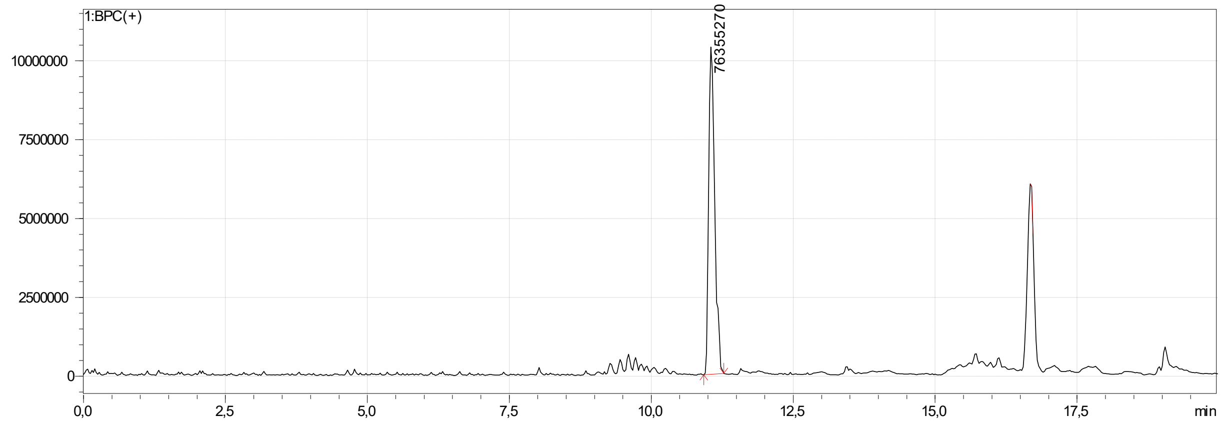

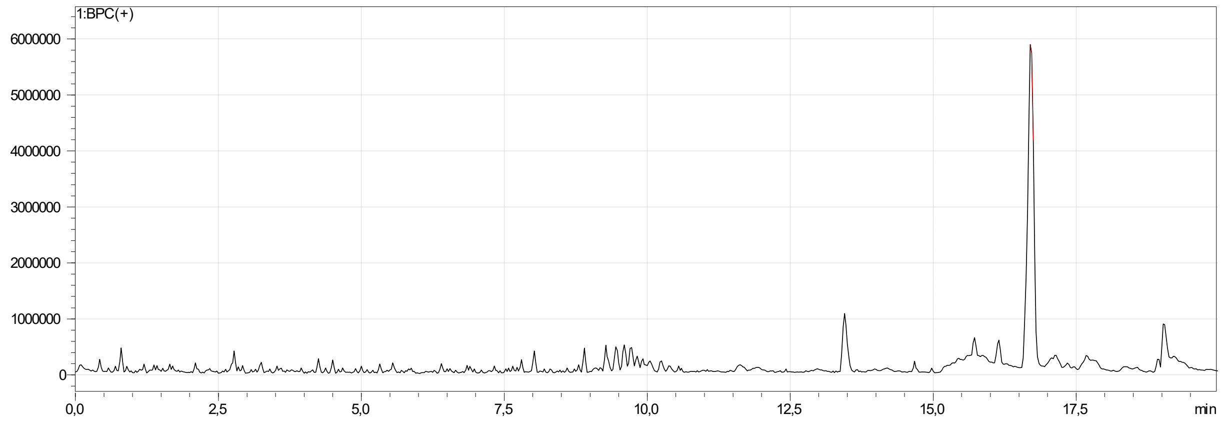

Negative ions (chromatogram and blank)

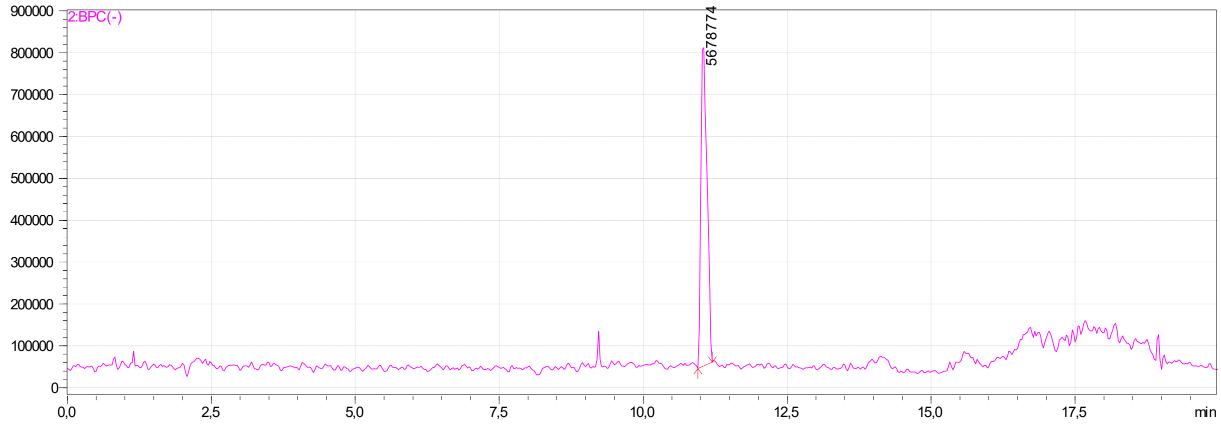

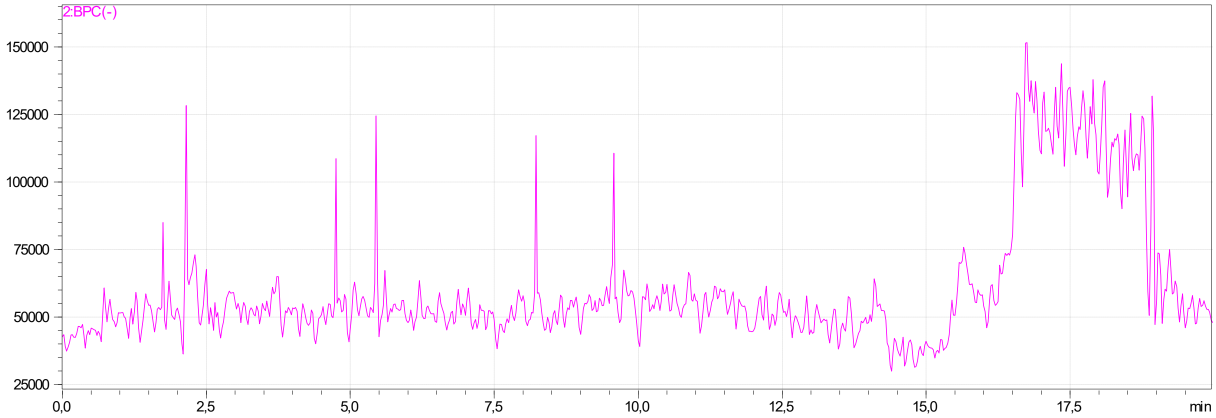

LCMS spectra for compound **6a**

Positive ions spectra for compound **6a**

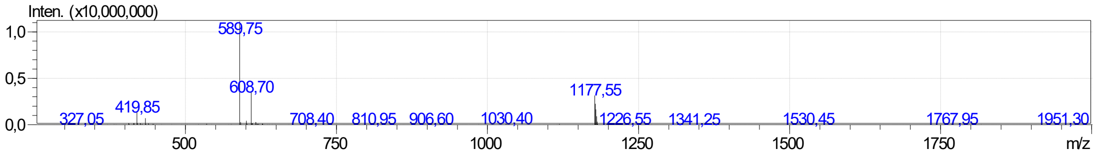

Negative ions spectra for compound **6a**

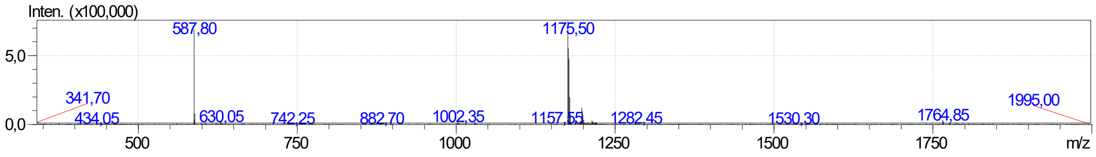

^{1}H NMR spectra for compound **6b**

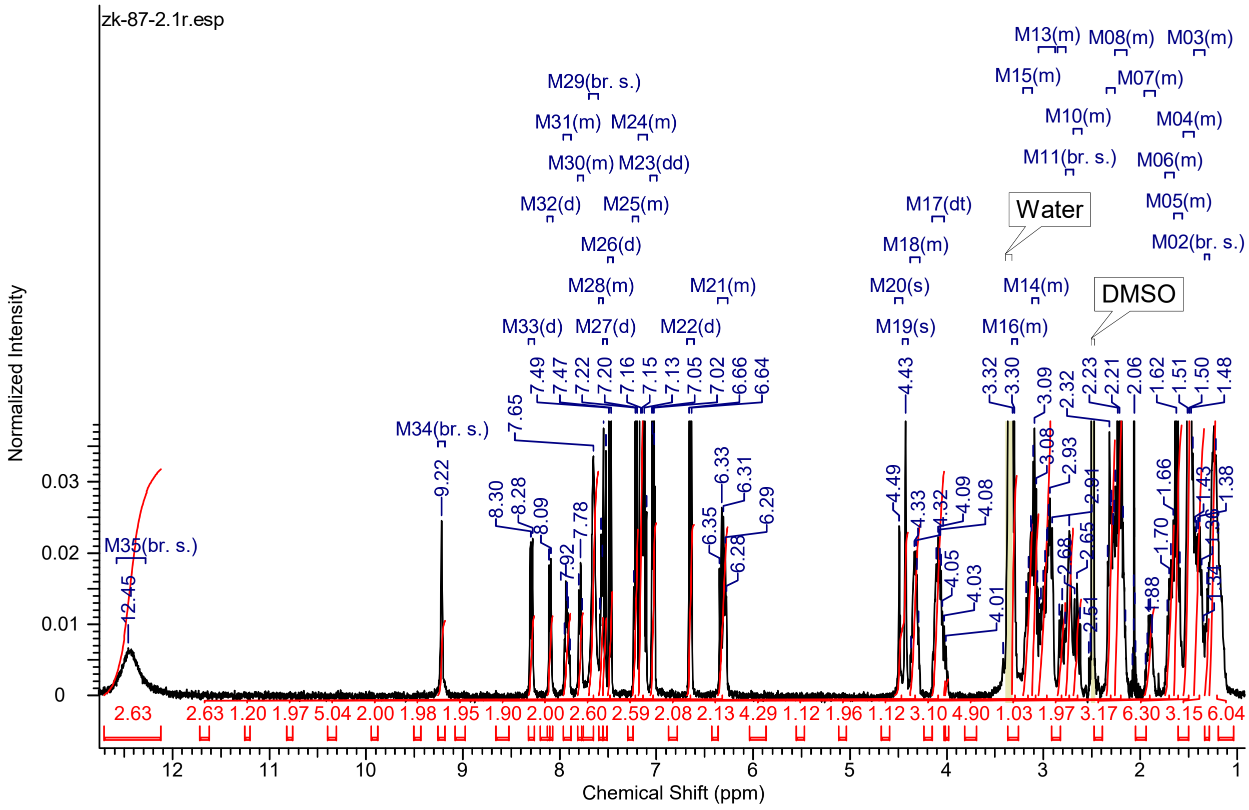

^{13}C NMR spectra for compound **6b**

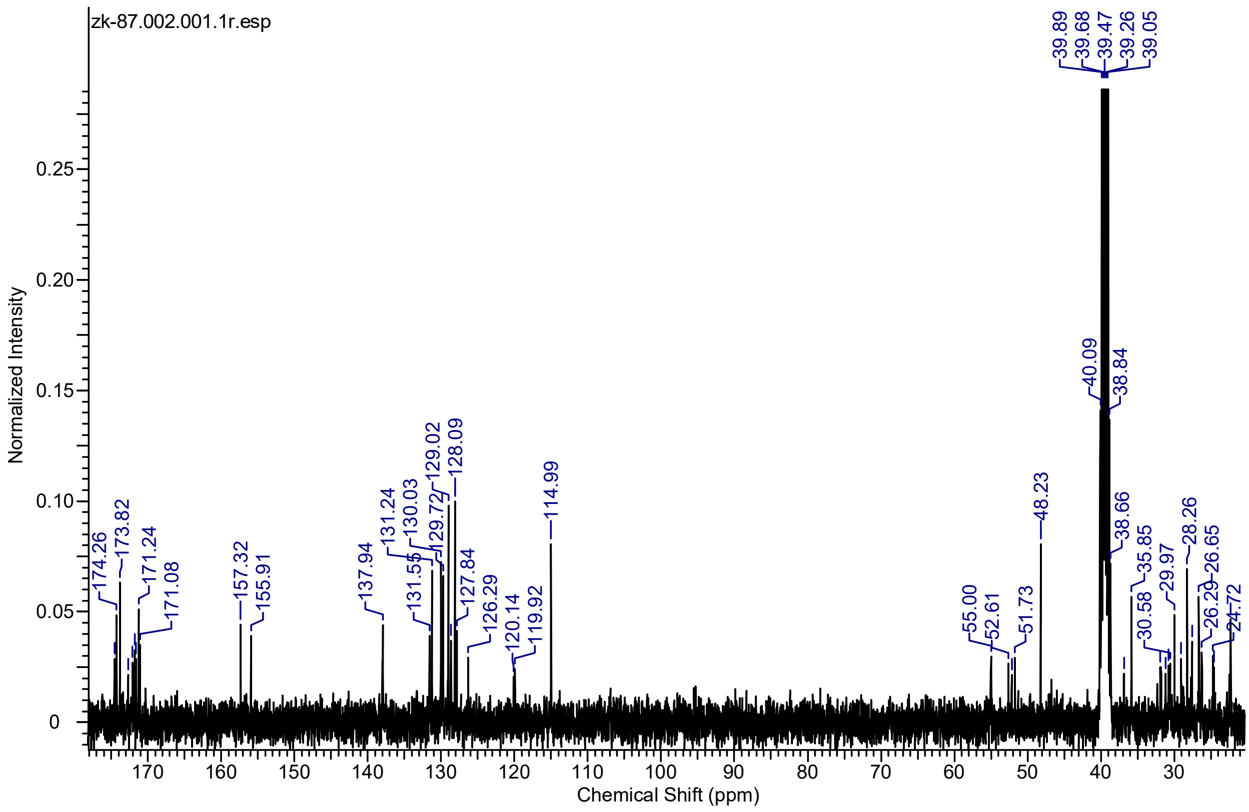

ESI-HRMS for compound **6b**

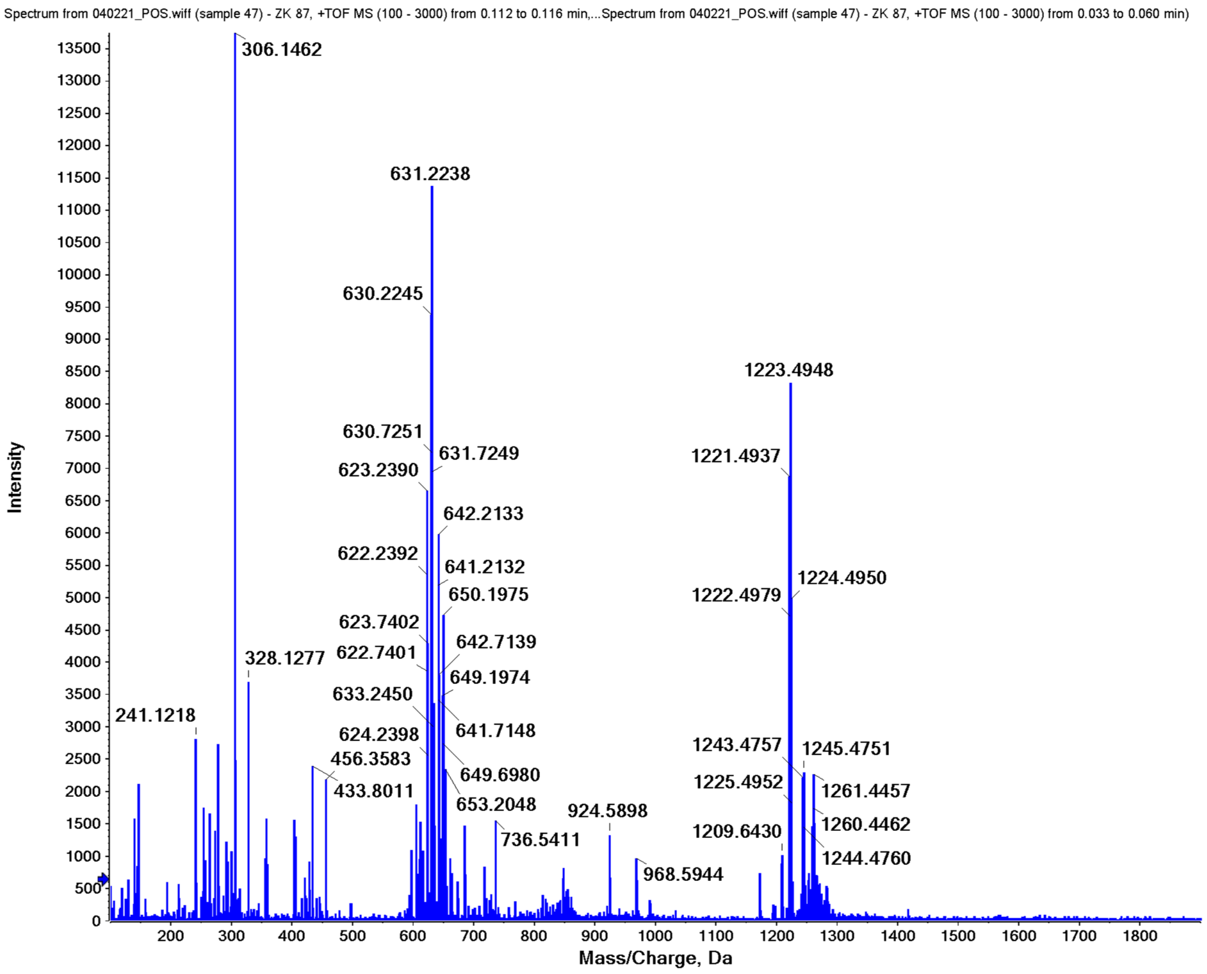

HPLC chromatogram for compound **6b**

Positive ions (chromatogram and blank)

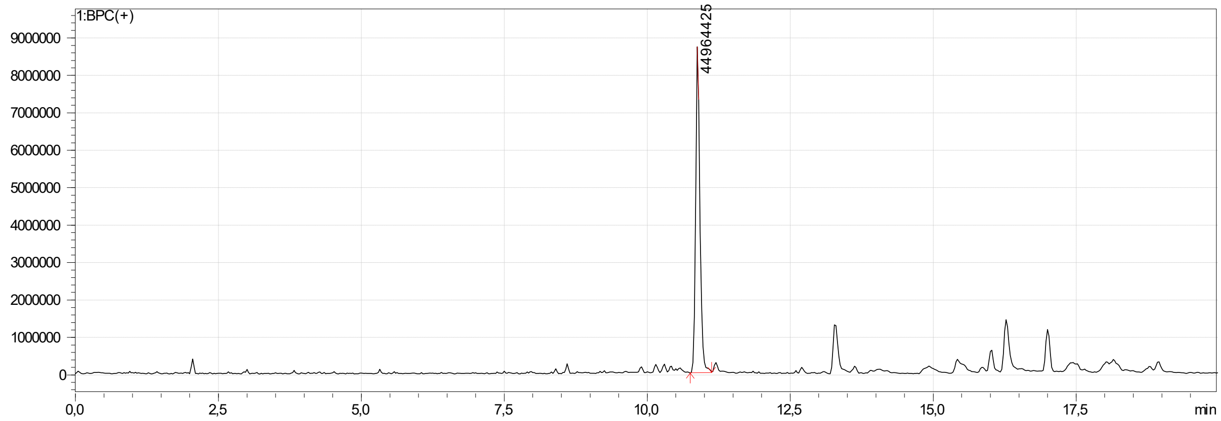

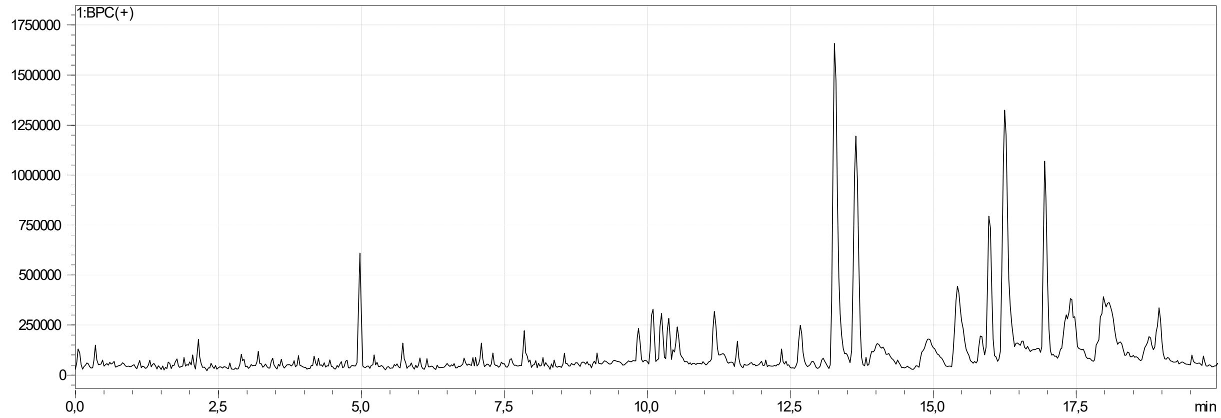

Negative ions (chromatogram and blank)

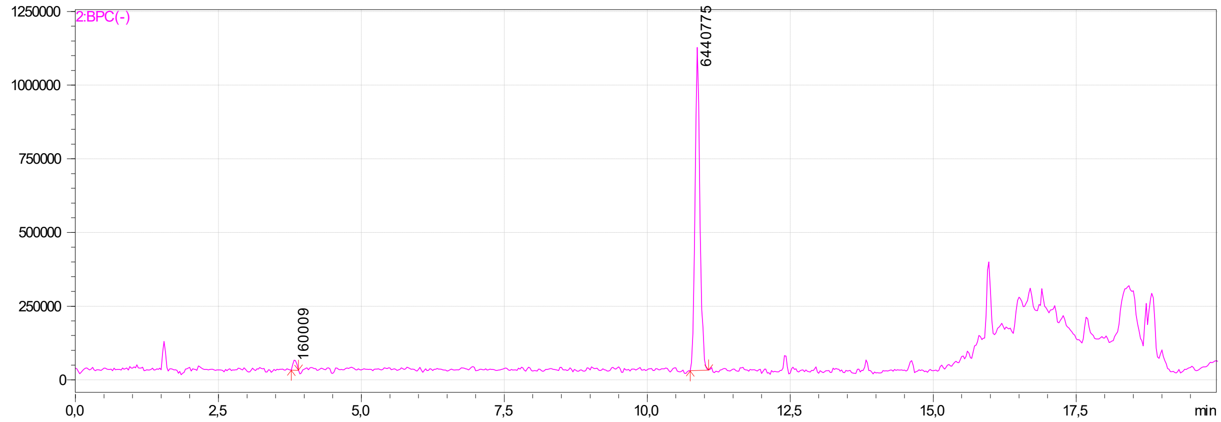

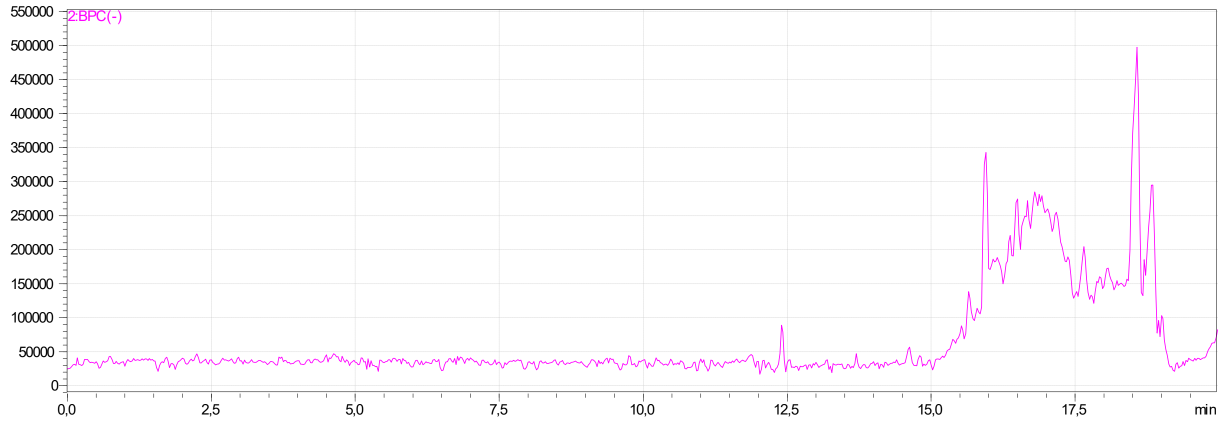

LCMS spectra for compound **6b**

Positive ions spectra for compound **6b**

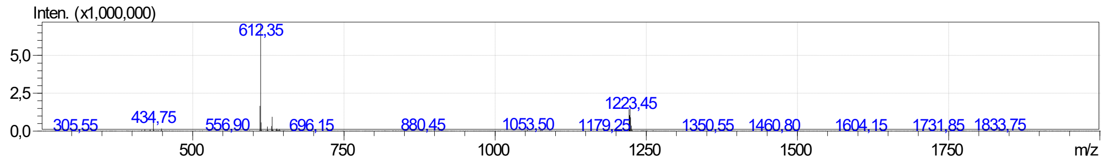

Negative ions spectra for compound **6b**

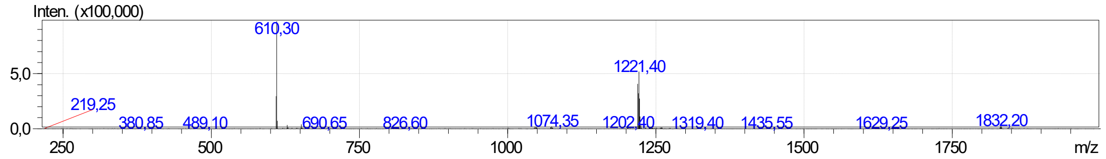

^{1}H NMR spectra for compound **6c**

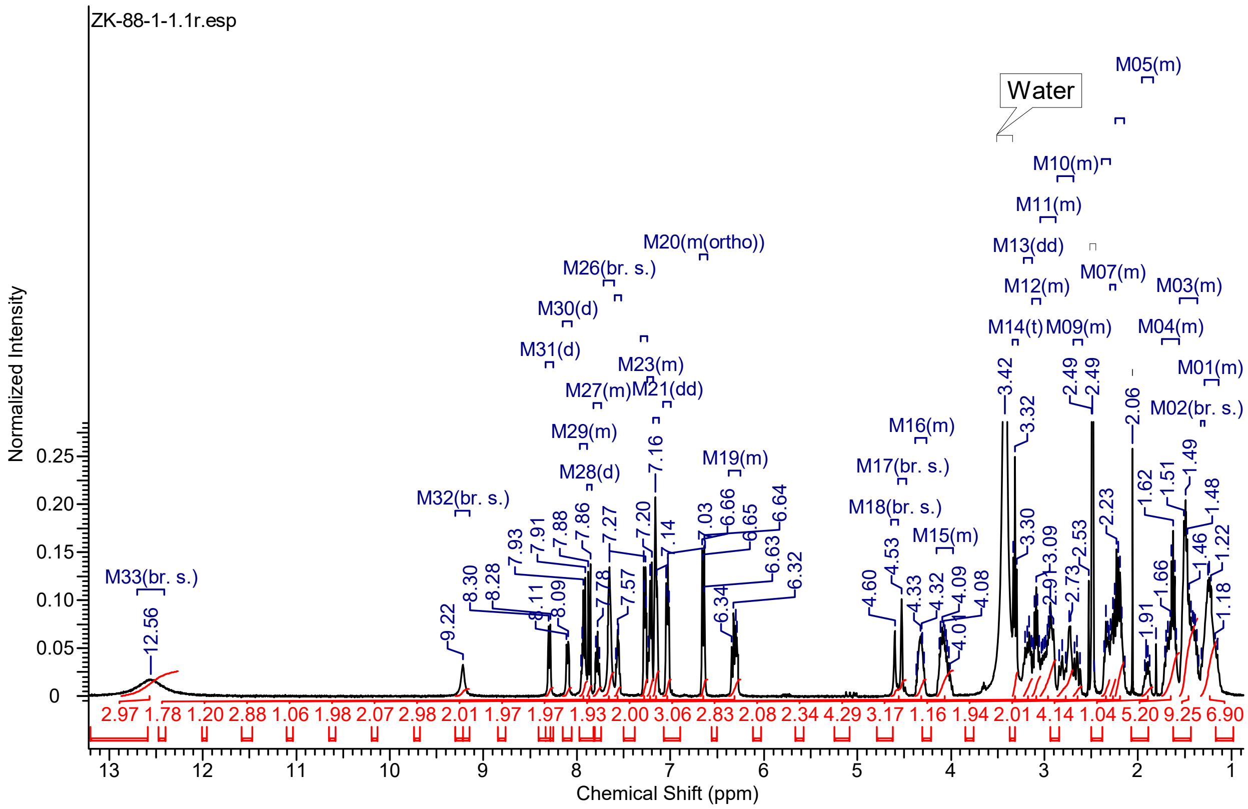

^{13}C NMR spectra for compound **6c**

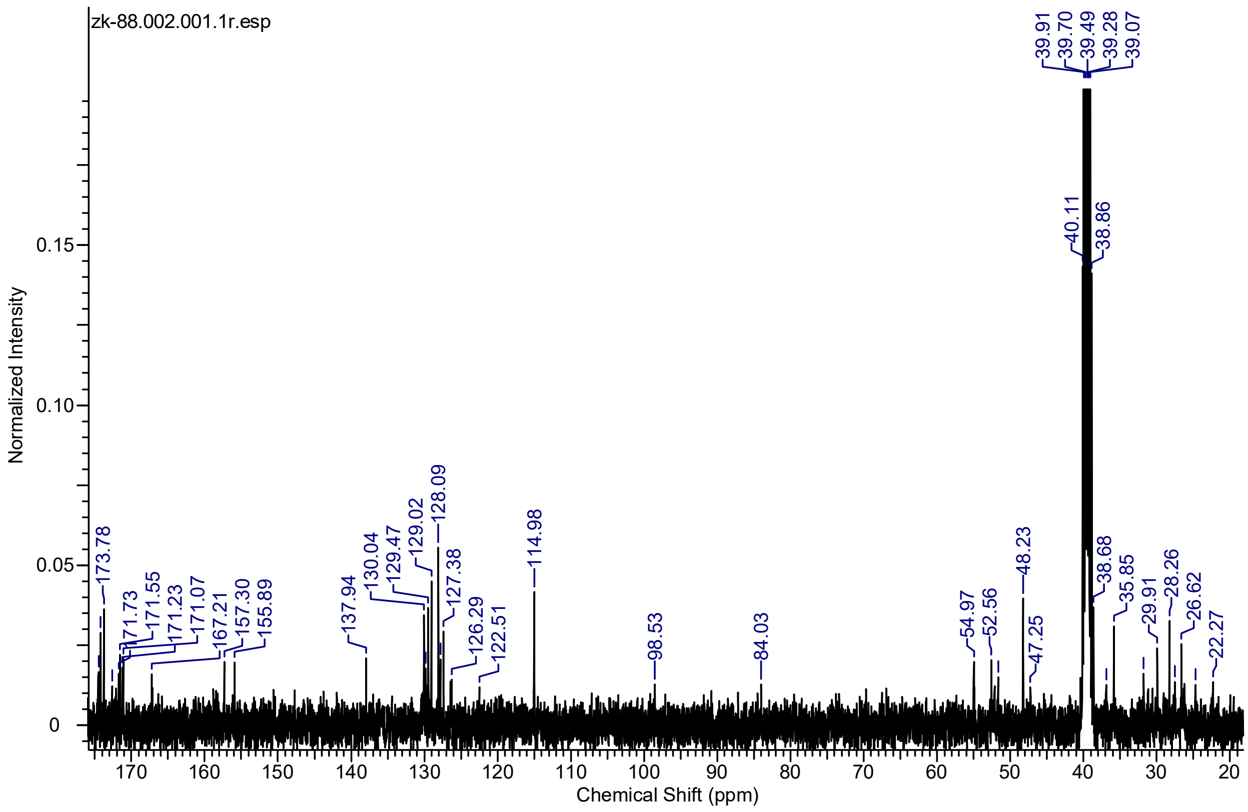

ESI-HRMS for compound **6c**

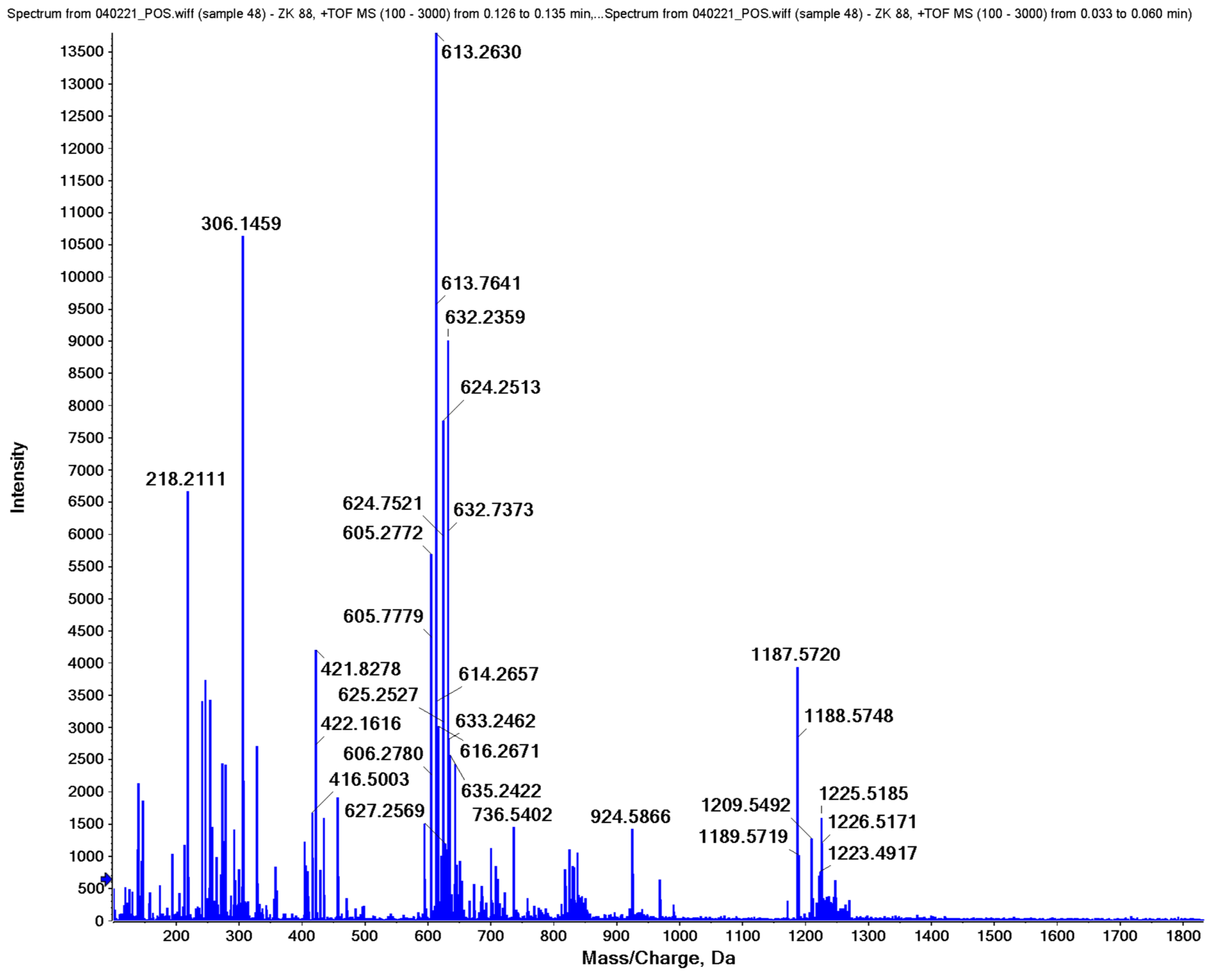

HPLC chromatogram for compound **6c**

Positive ions (chromatogram and blank)

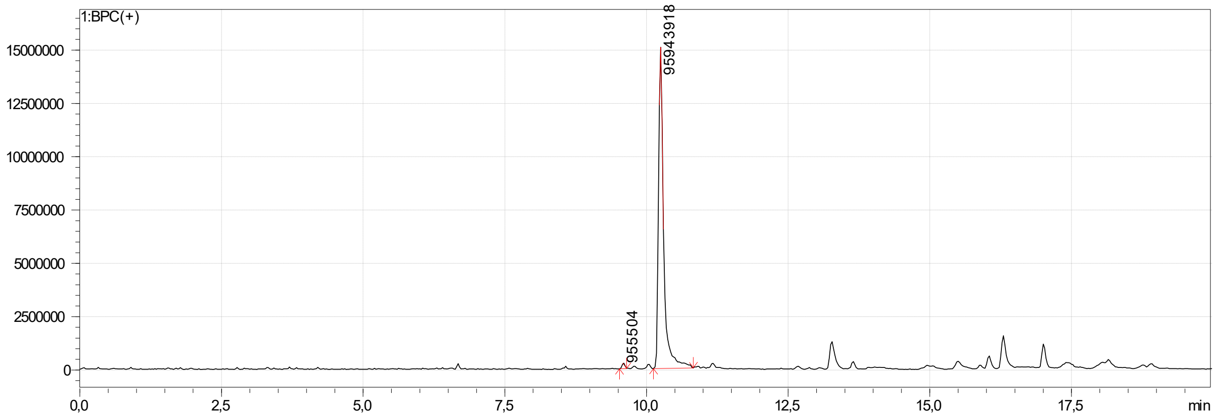

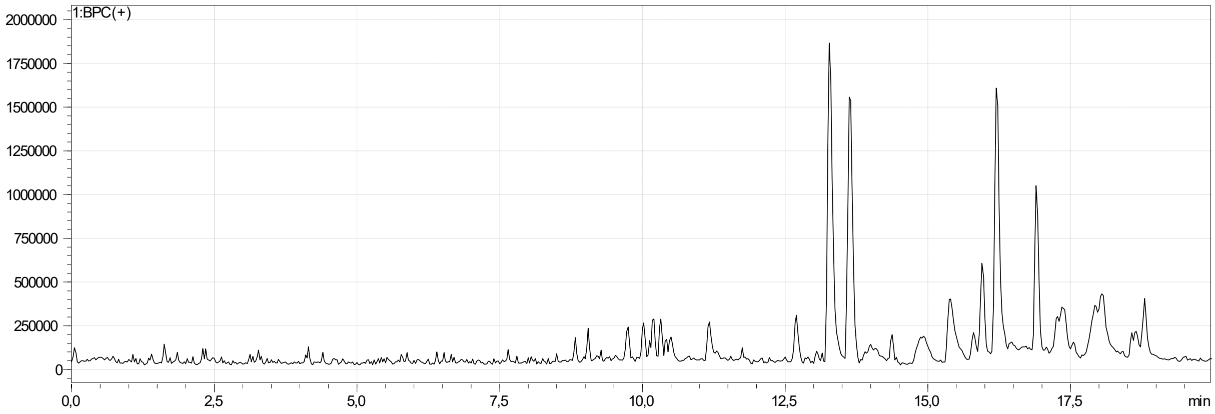

Negative ions (chromatogram and blank)

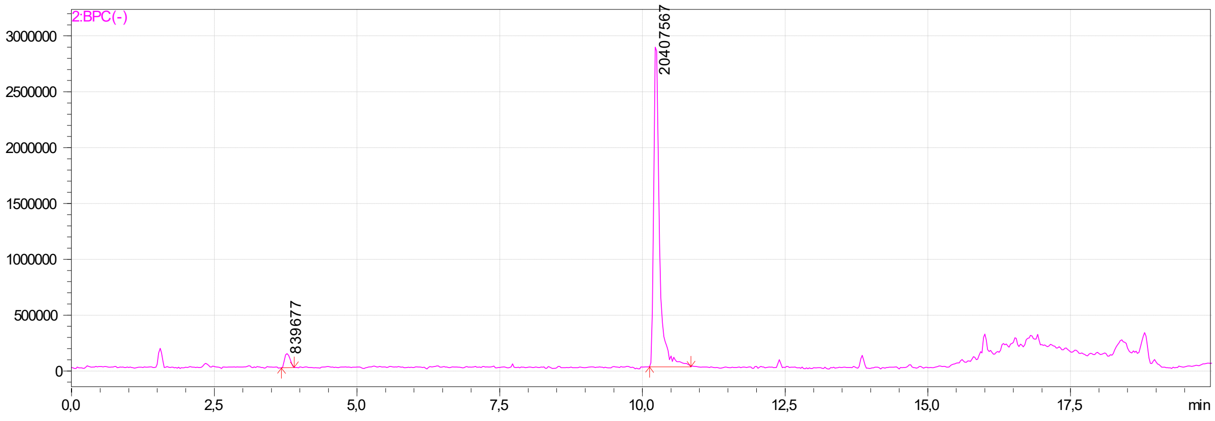

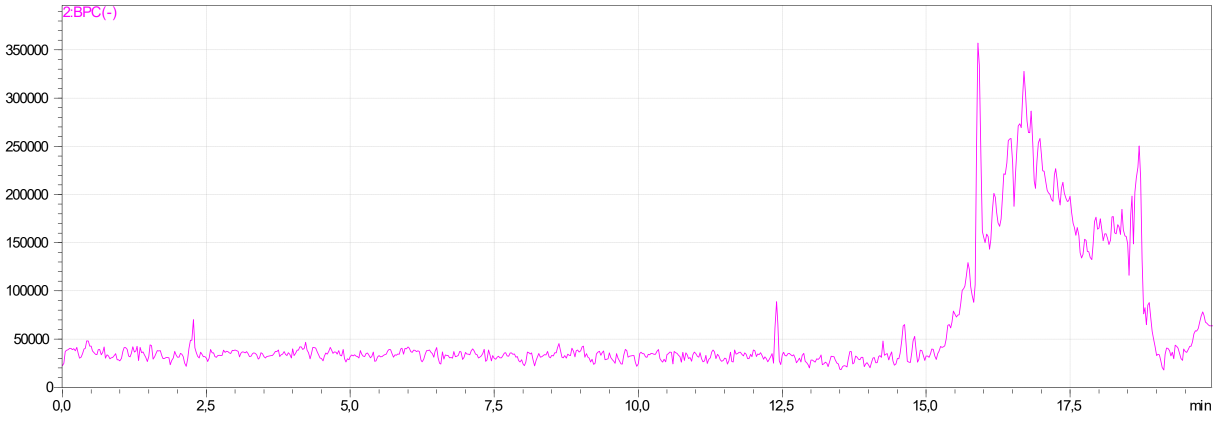

LCMS spectra for compound **6c**

Positive ions spectra for compound **6c**

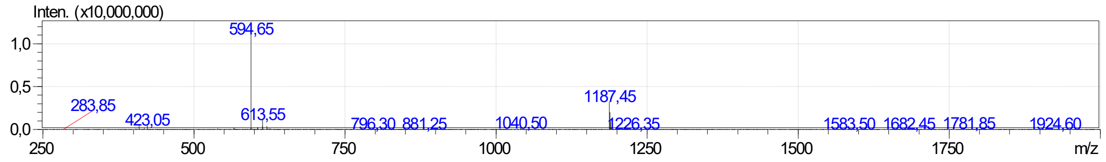

Negative ions spectra for compound **6c**

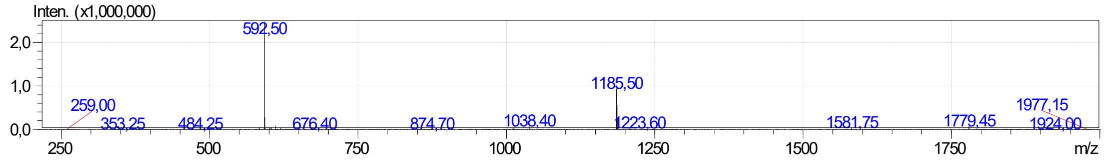

^{1}H NMR spectra for compound **6d**

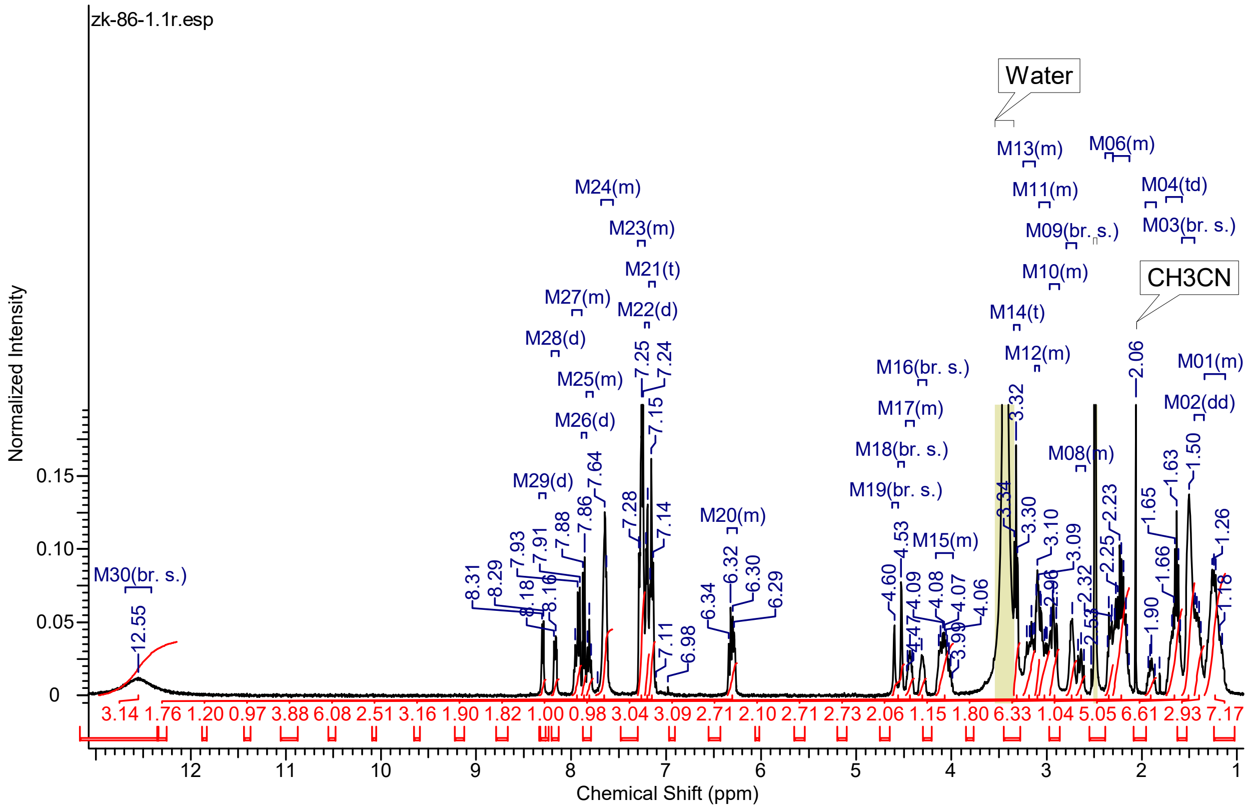

ESI-HRMS for compound **6d**

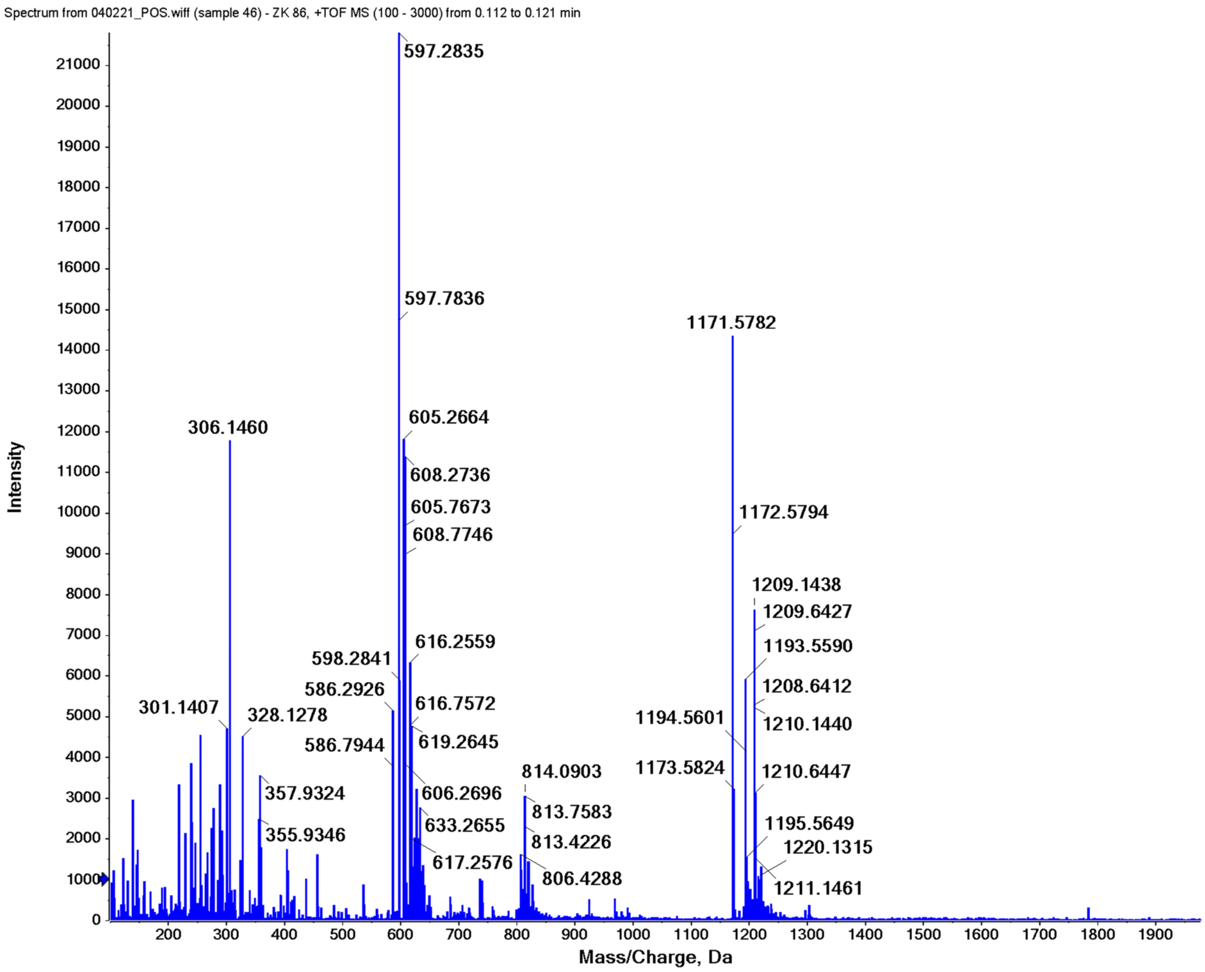

HPLC chromatogram for compound **6d**

Positive ions (chromatogram and blank)

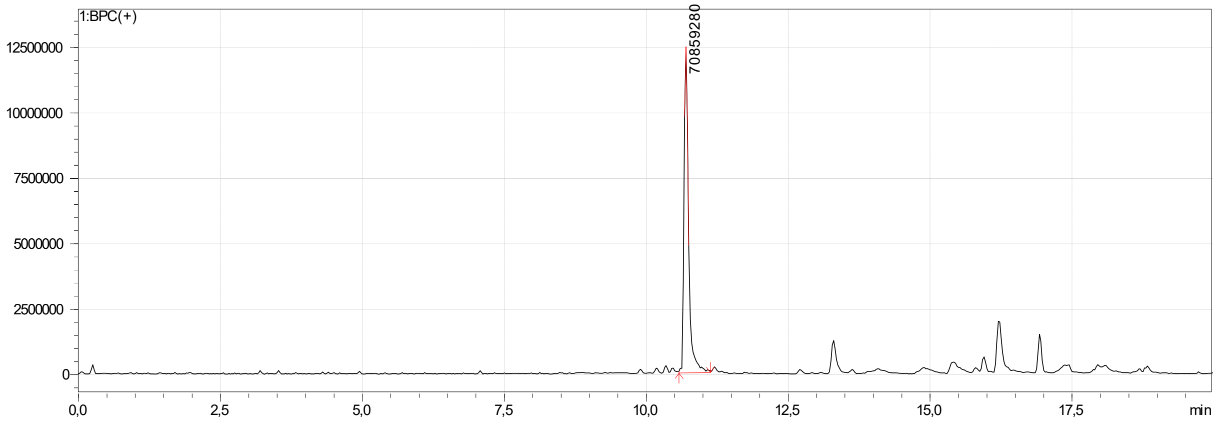

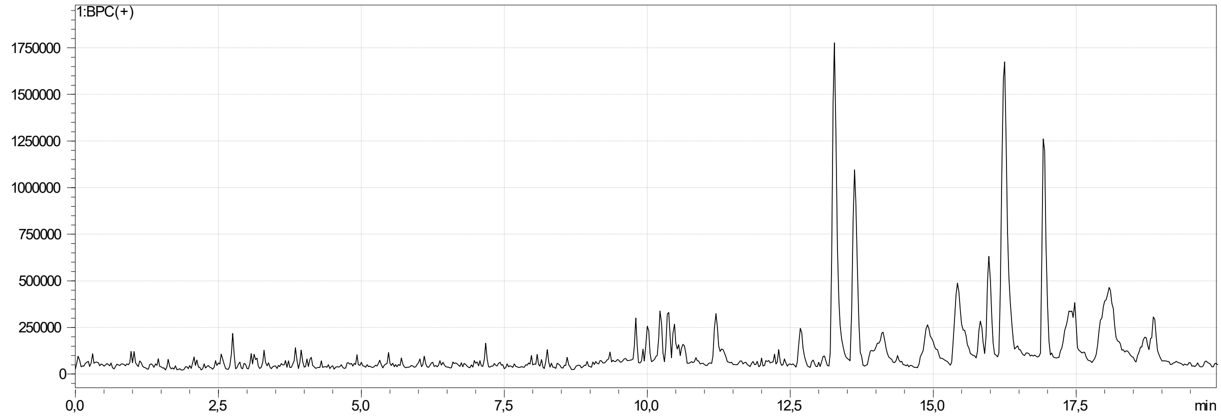

Negative ions (chromatogram and blank)

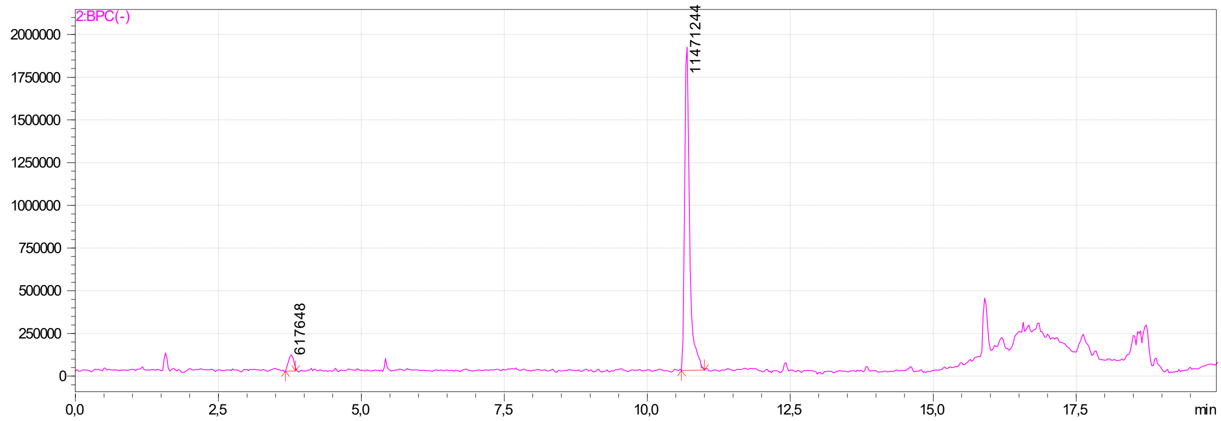

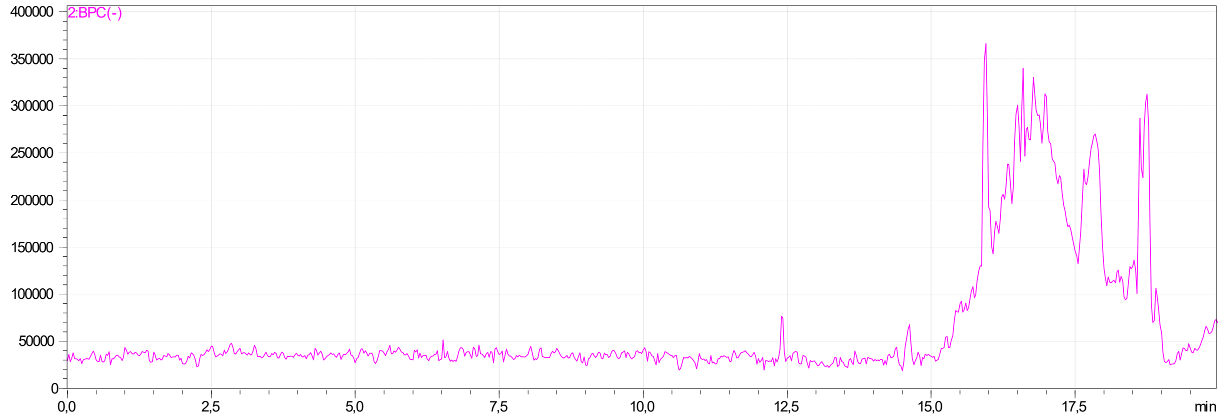

LCMS spectra for compound **6d**

Positive ions spectra for compound **6d**

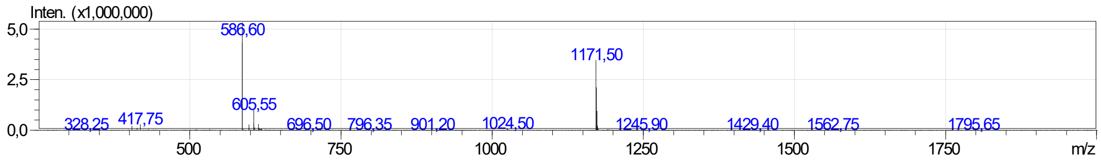

Negative ions spectra for compound **6d**

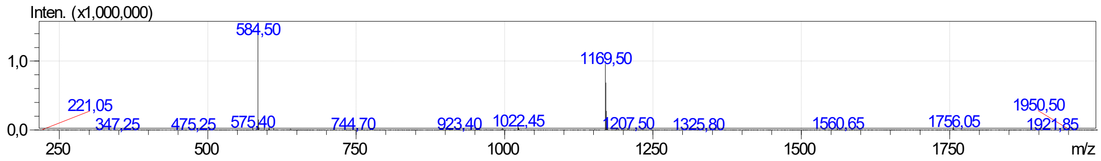

^{1}H NMR spectra for compound **Abi**

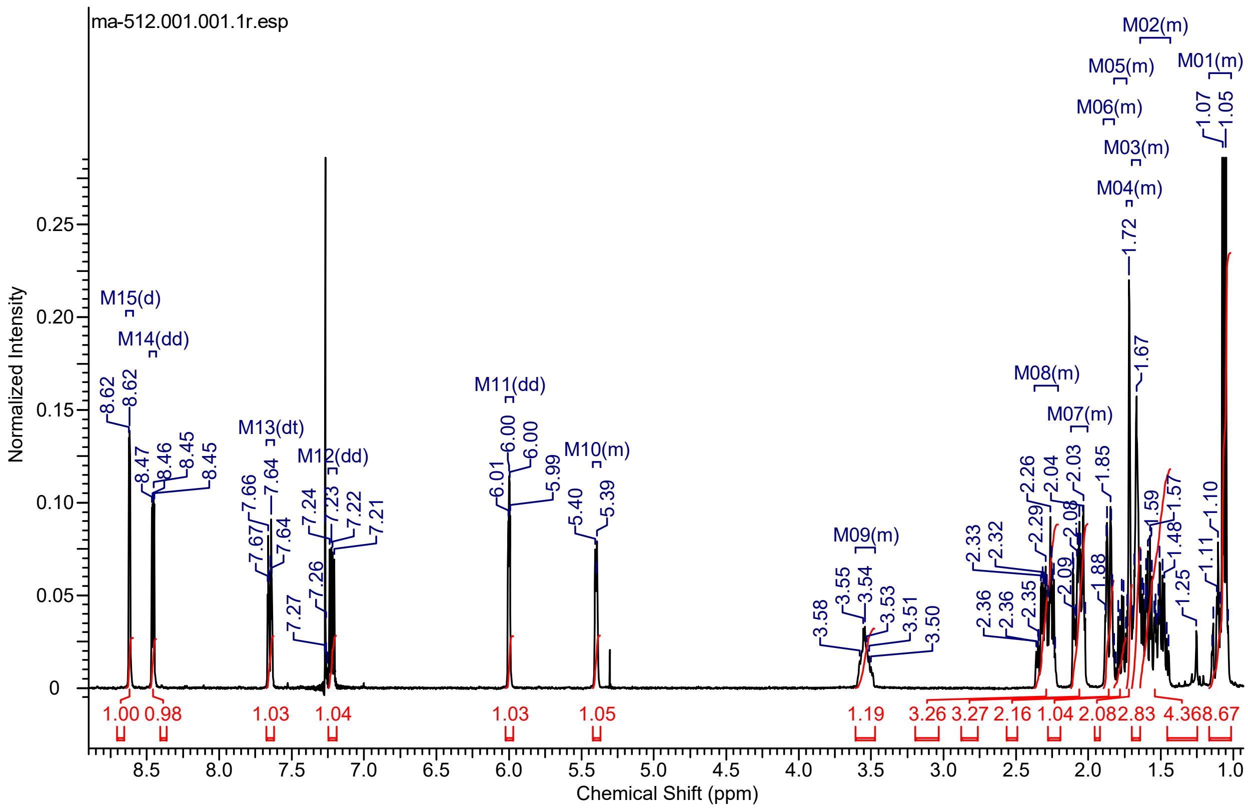

^{1}H NMR spectra for compound **Abi-Suc**

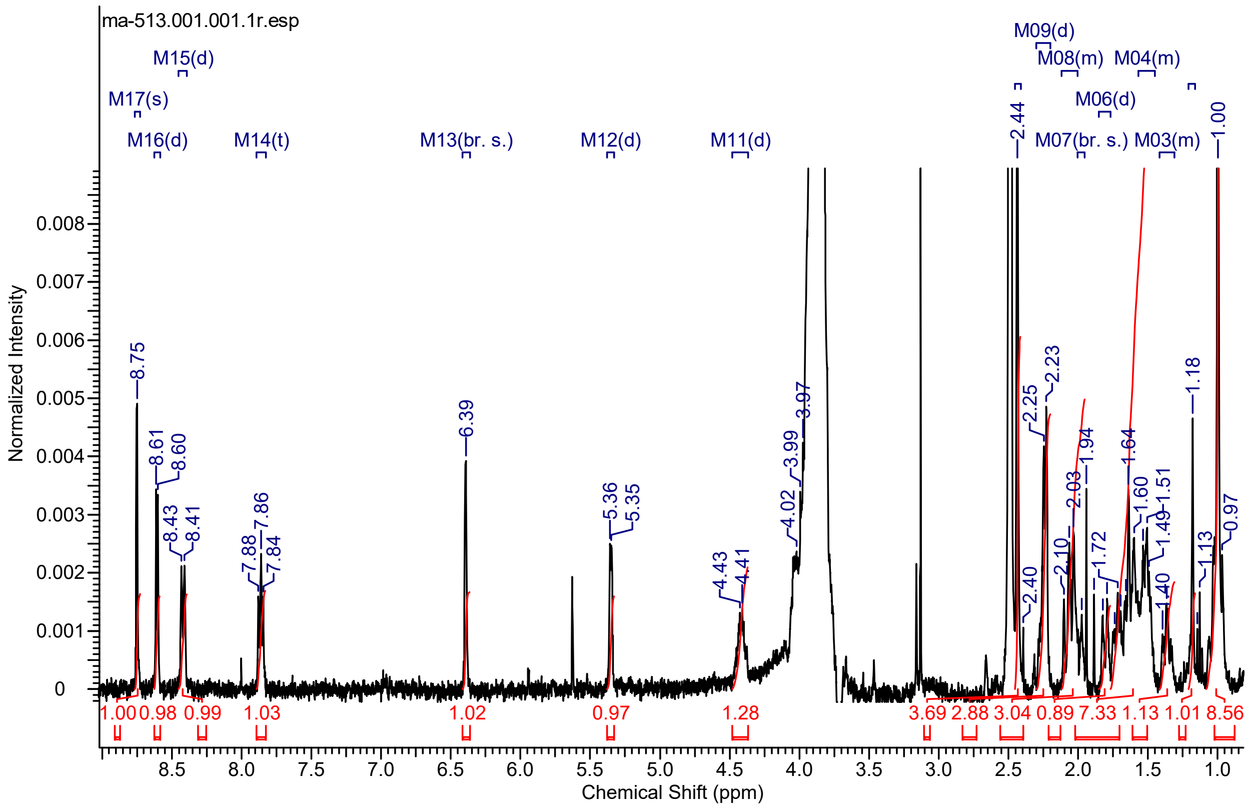

^{1}H NMR spectra for compound **7**

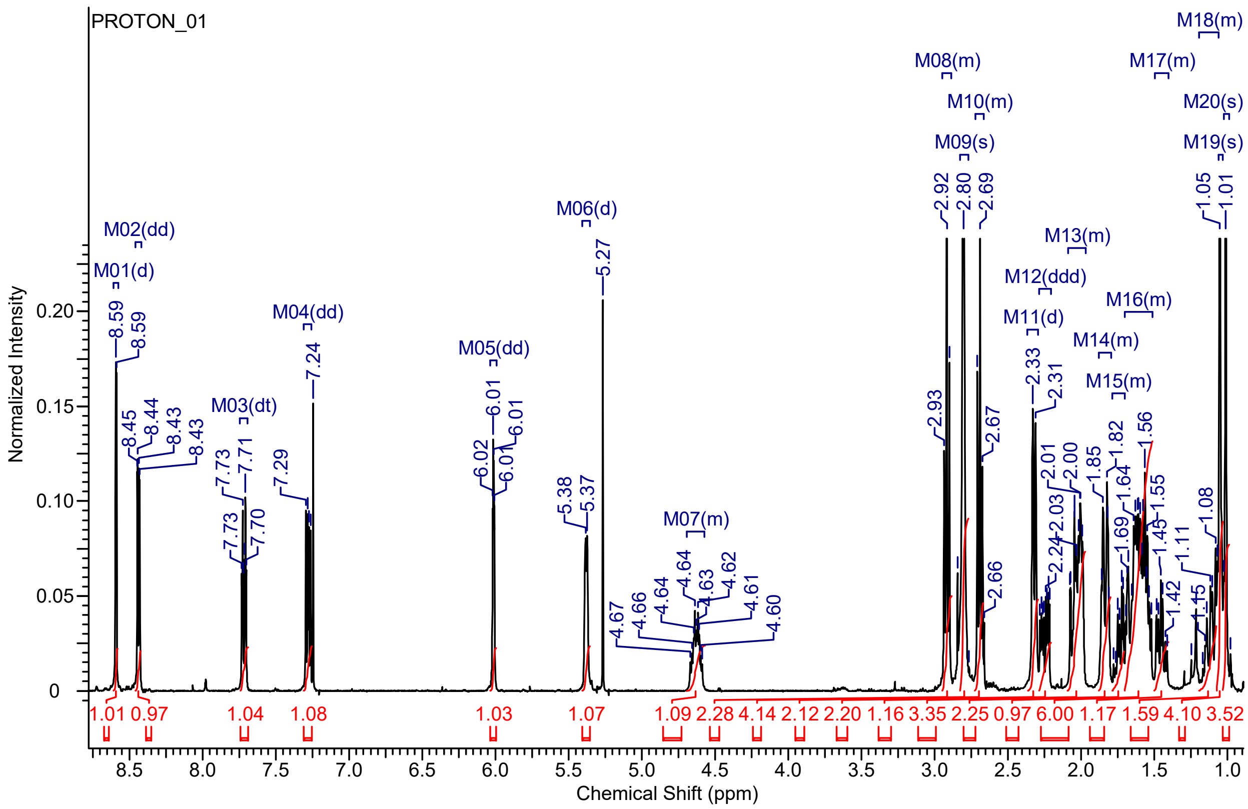

^{13}C NMR spectra for compound **7**

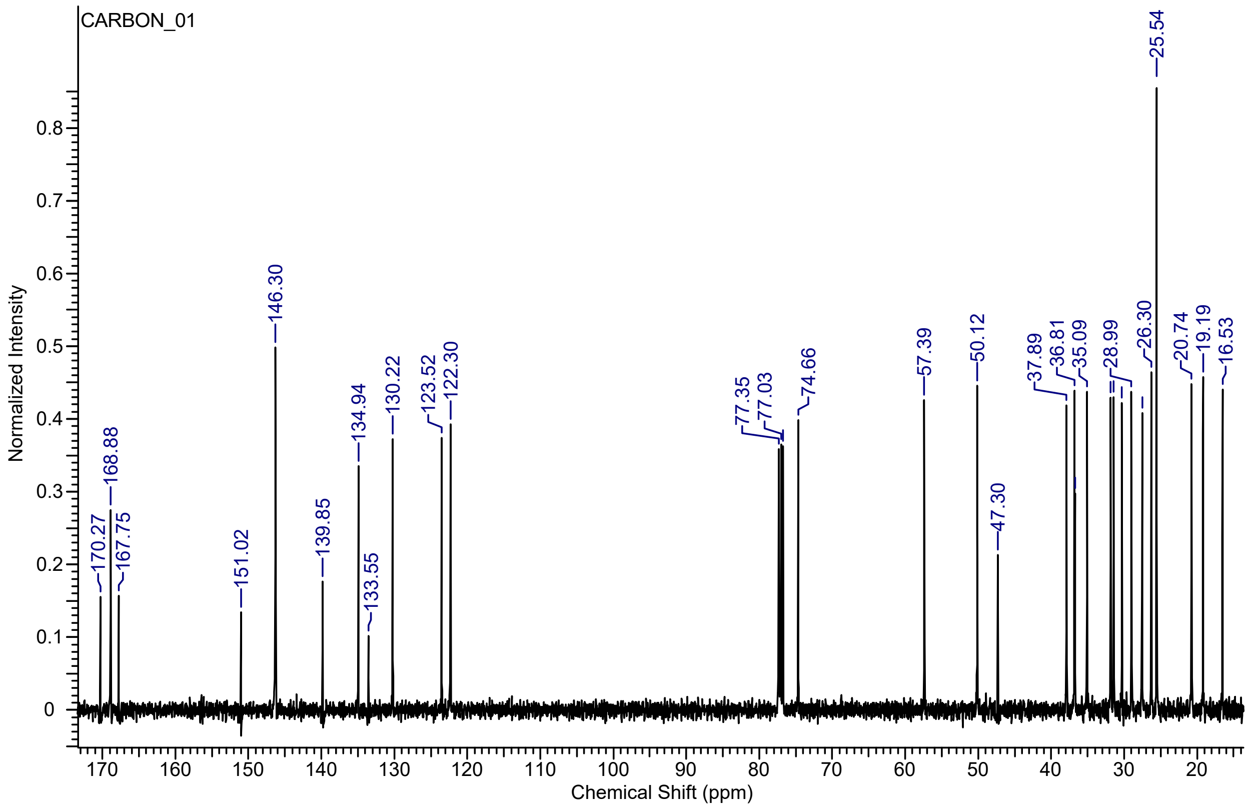

^{1}H NMR spectra for compound **8a**

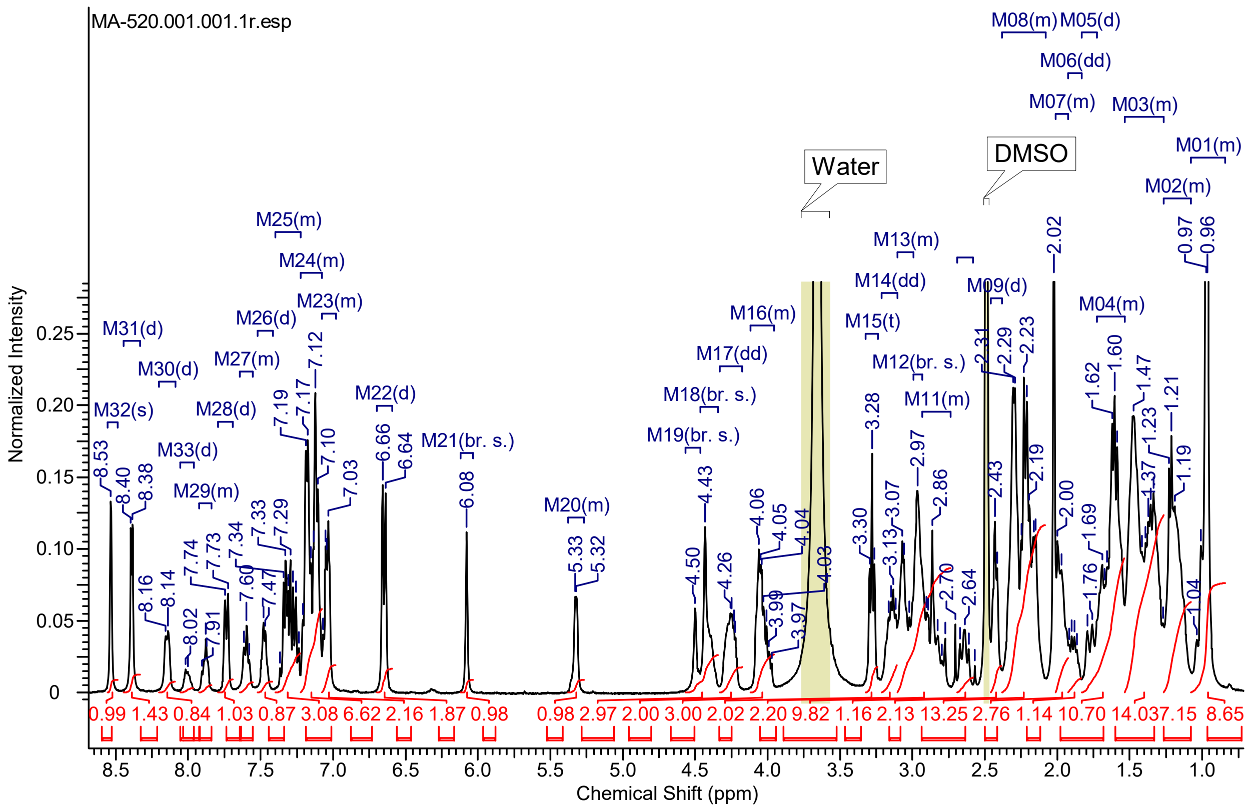

HPLC chromatogram for compound **8a**

Positive ions (chromatogram and blank)

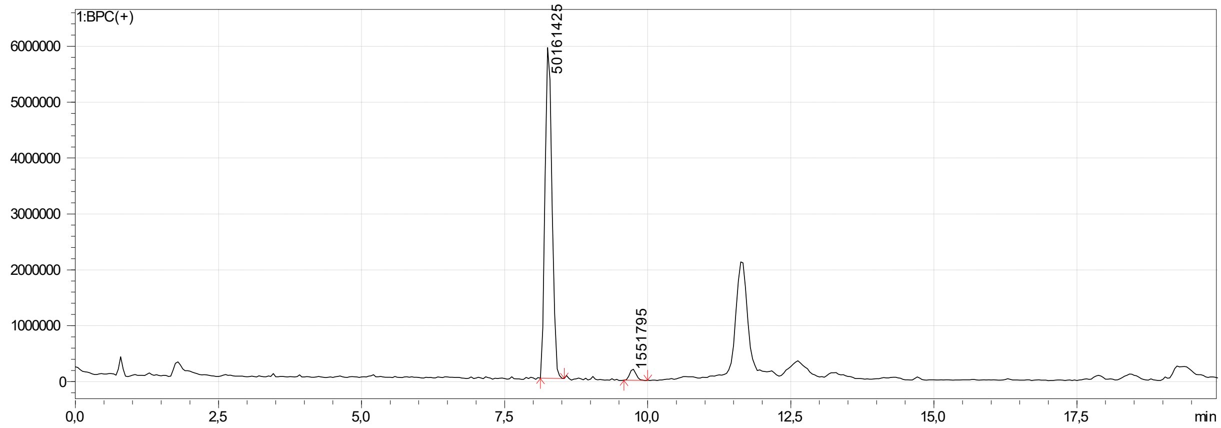

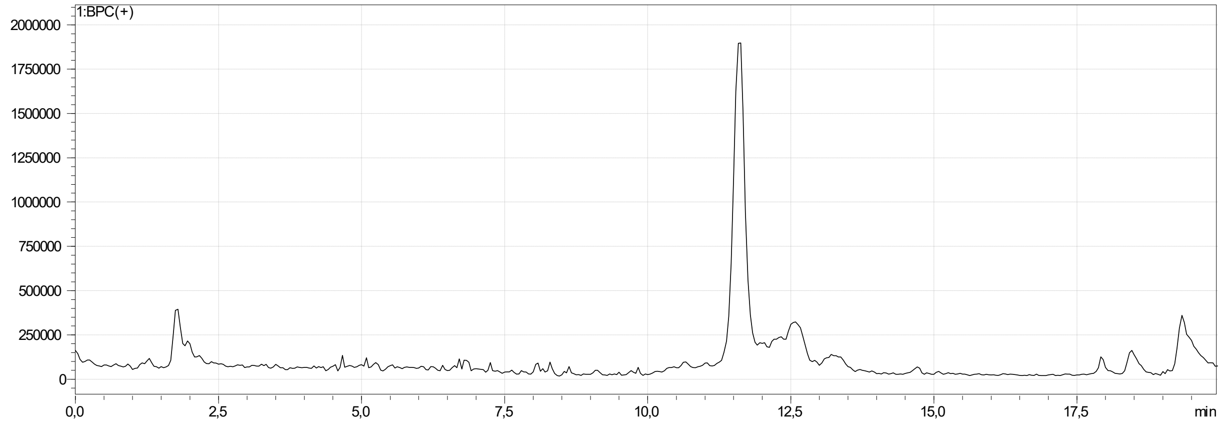

LCMS spectra for compound **8a**

Positive ions spectra for compound **8a**

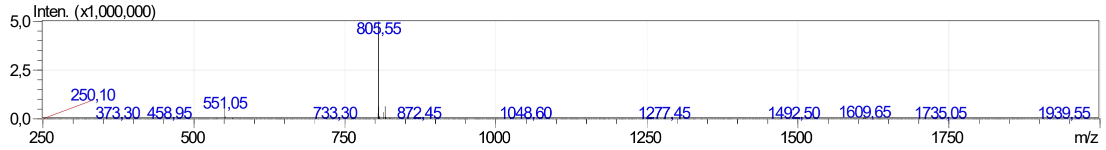

Negative ions spectra for compound **8a**

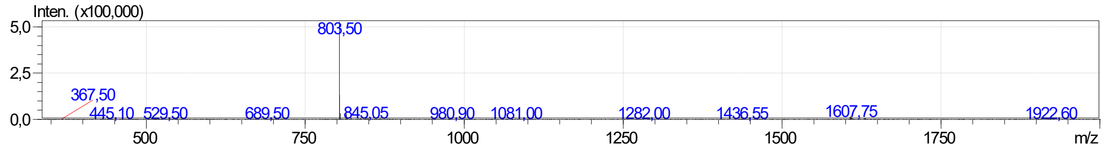

^{1}H NMR spectra for compound **8b**

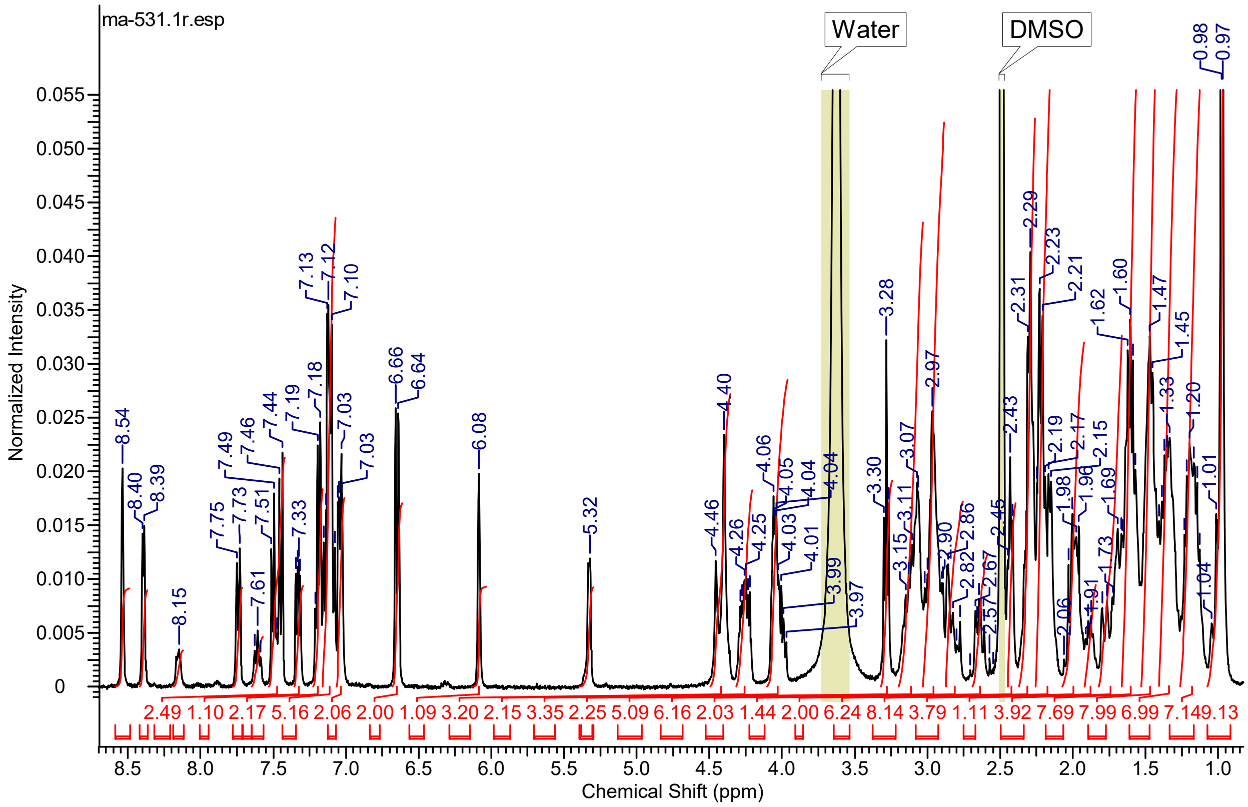

ESI-HRMS for compound **8b**

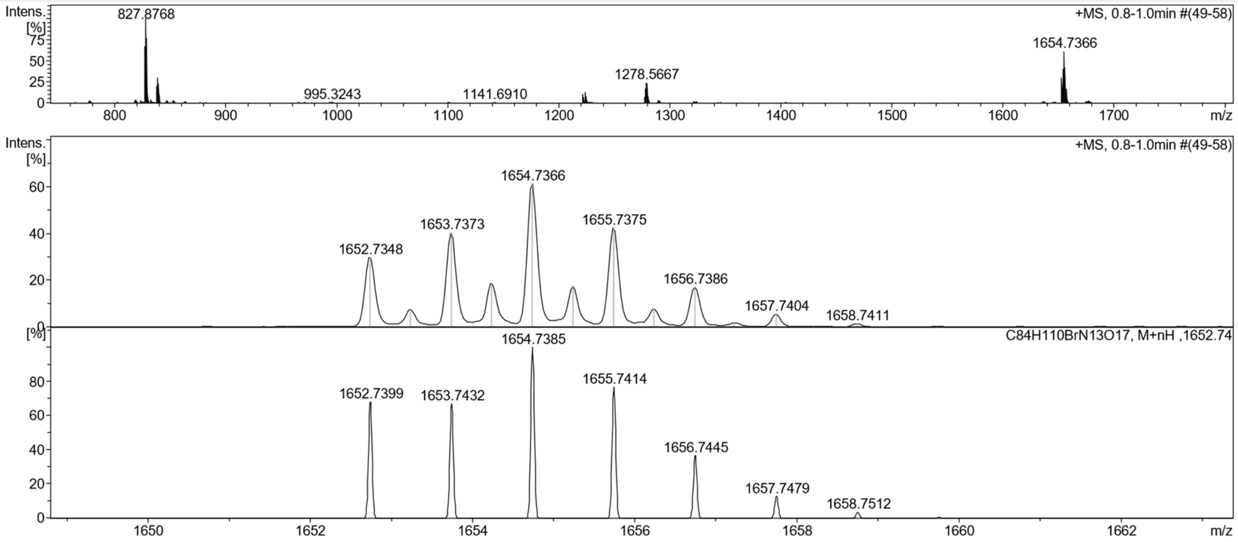

HPLC chromatogram for compound **8b**

Positive ions (chromatogram and blank)

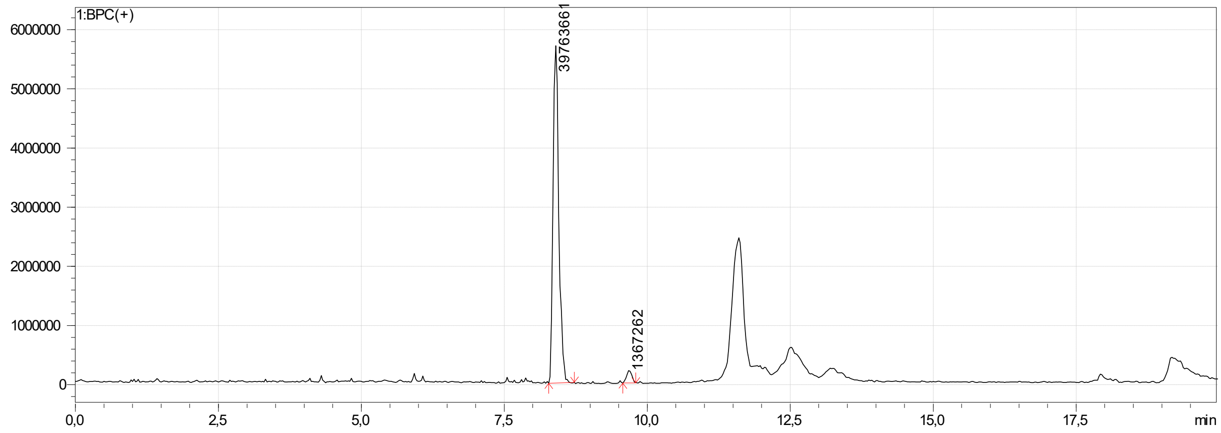

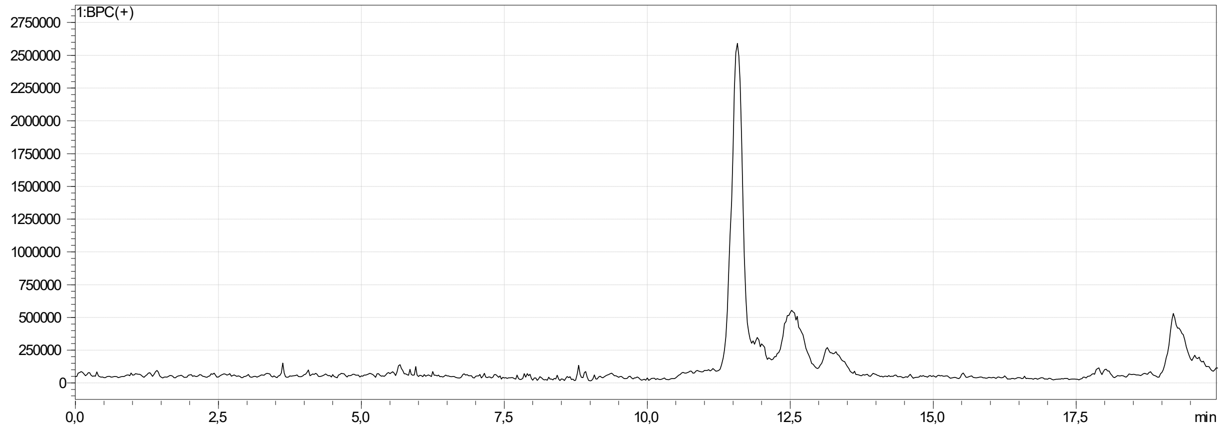

Negative ions (chromatogram and blank)

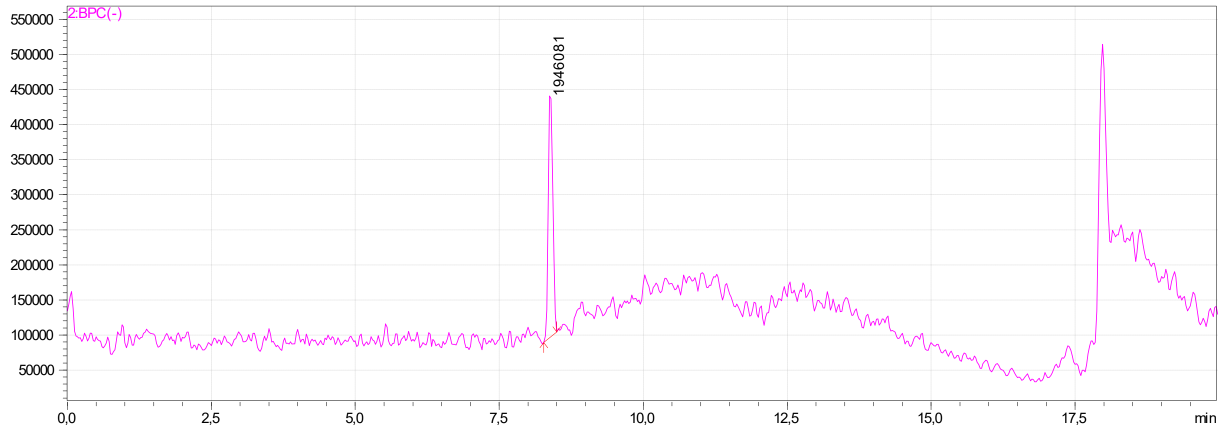

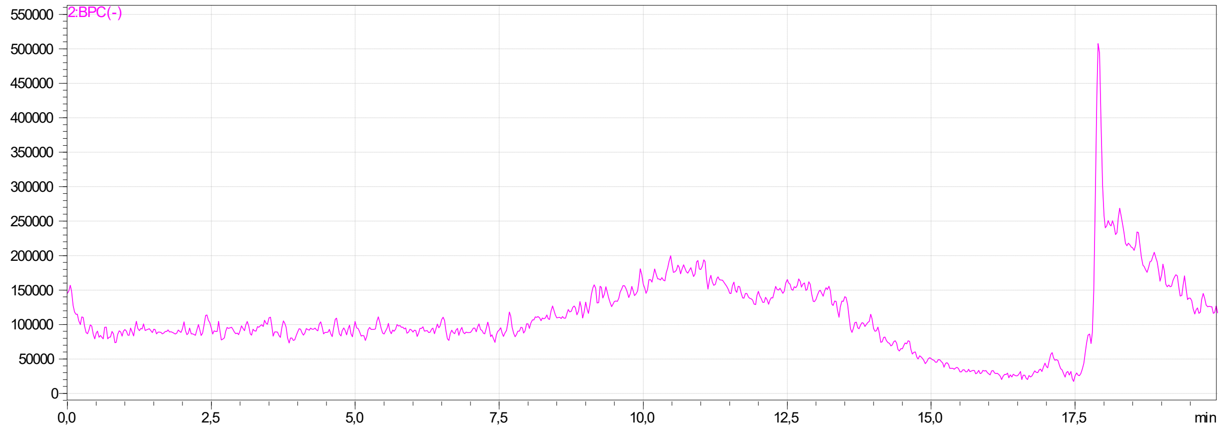

LCMS spectra for compound **8b**

Positive ions spectra for compound **8b**

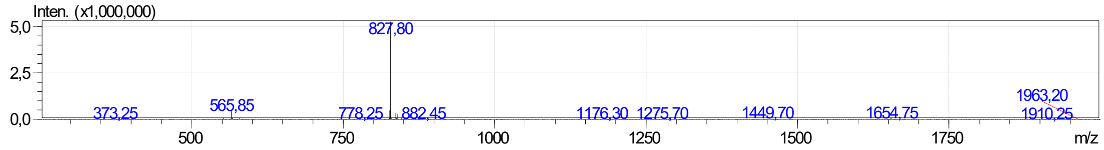

Negative ions spectra for compound **8b**

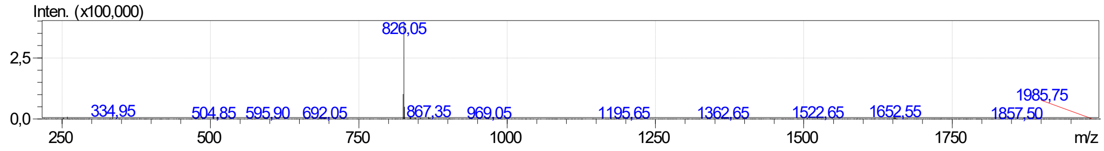

ESI-HRMS for compound **8c**

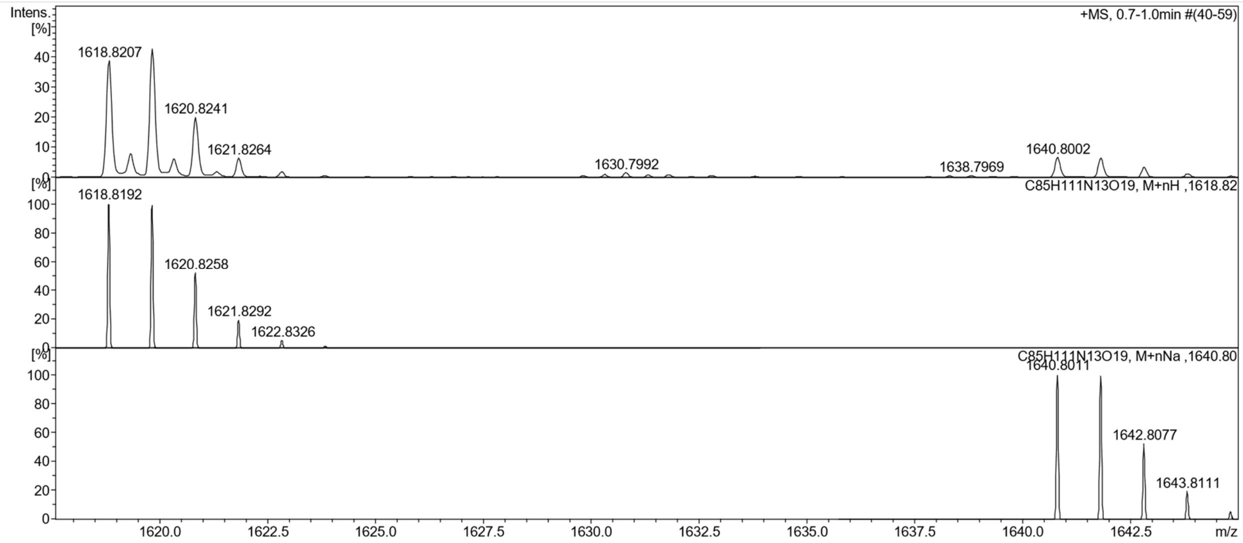

HPLC chromatogram for compound **8c**

Positive ions (chromatogram and blank)

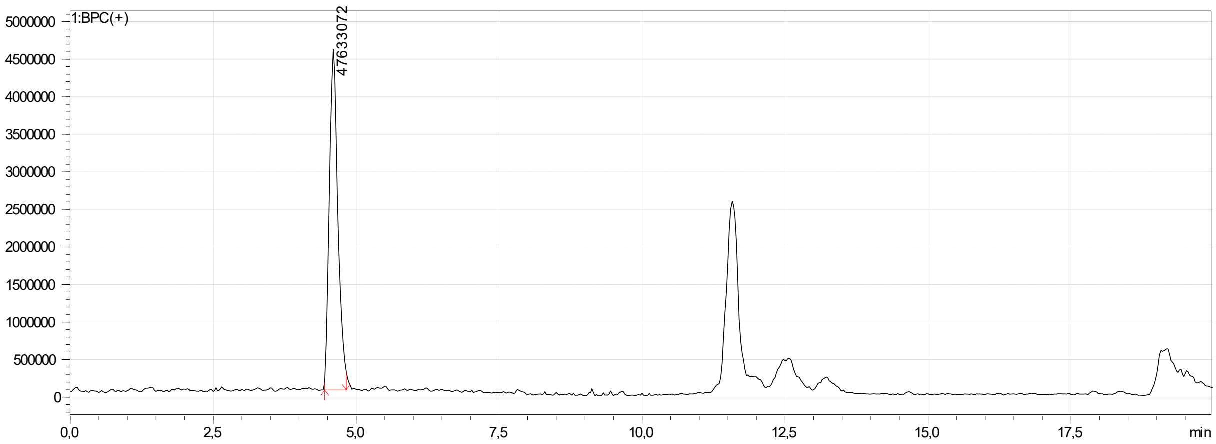

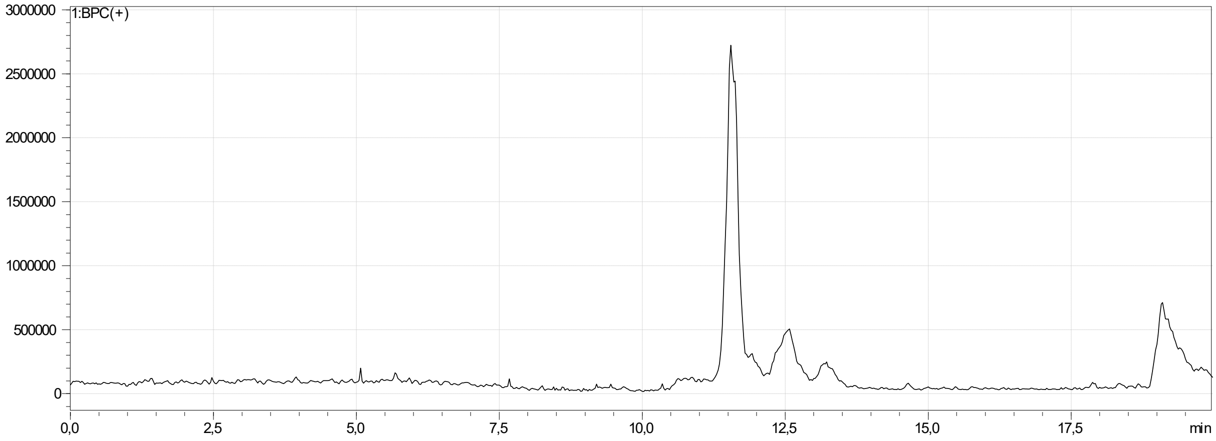

Negative ions (chromatogram and blank)

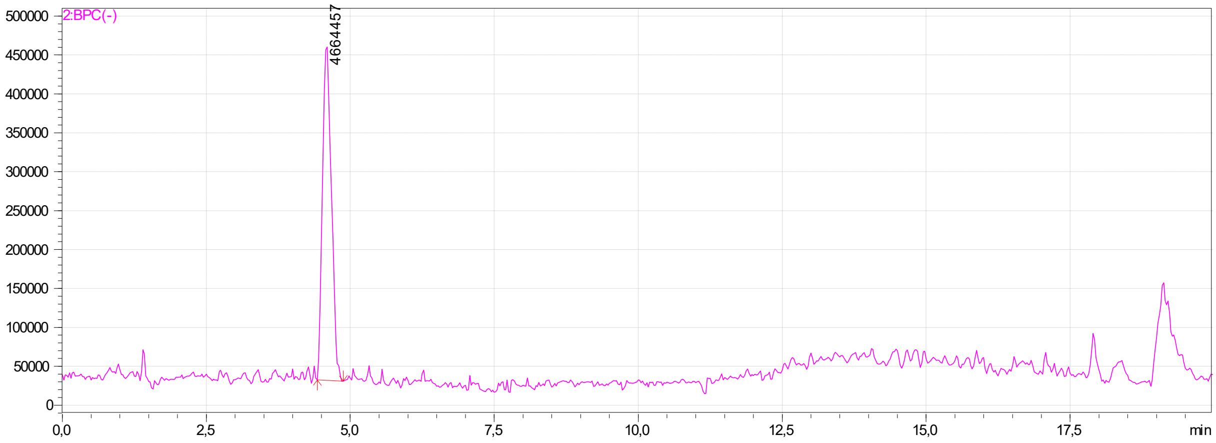

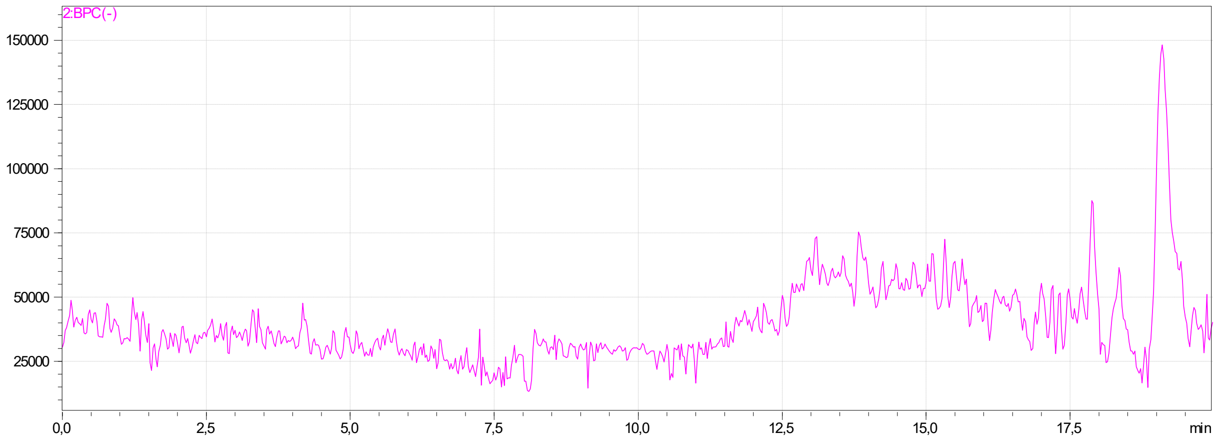

LCMS spectra for compound **8c**

Positive ions spectra for compound **8c**

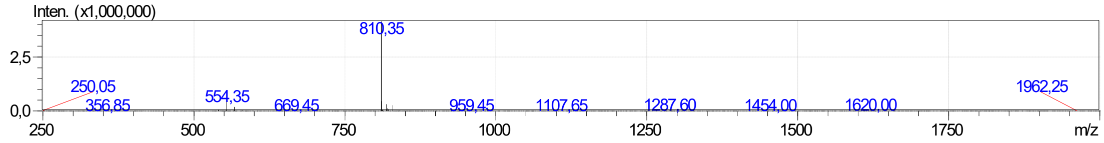

Negative ions spectra for compound **8c**

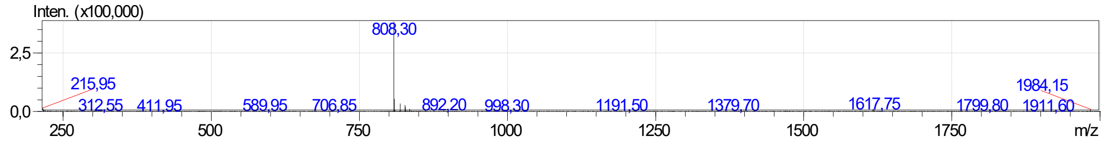

^{1}H NMR spectra for compound **8d**

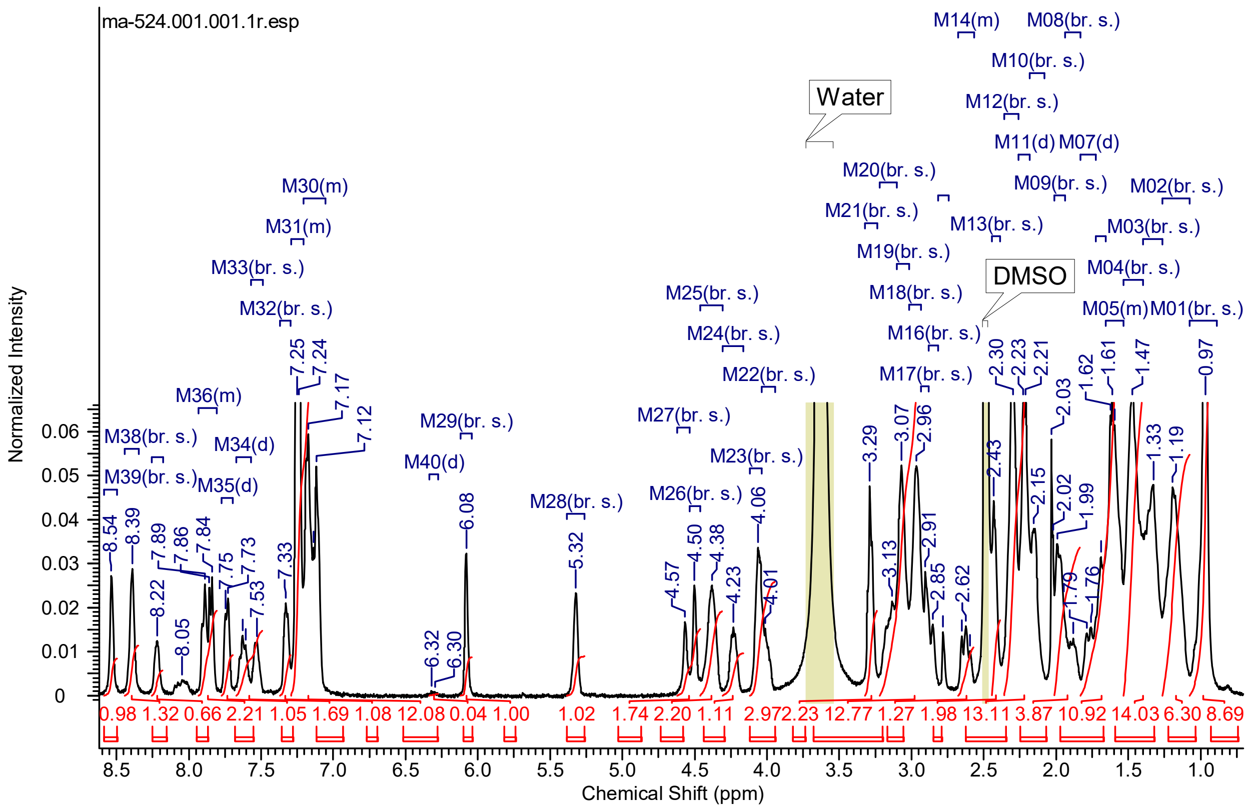

ESI-HRMS for compound **8d**

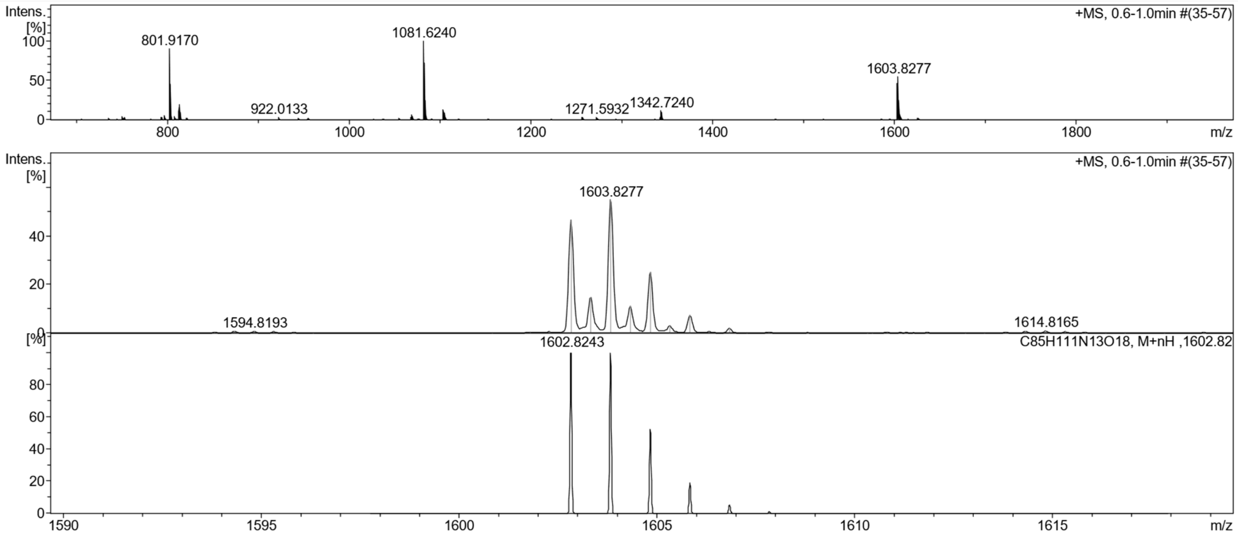

HPLC chromatogram for compound **8d**

Positive ions (chromatogram and blank)

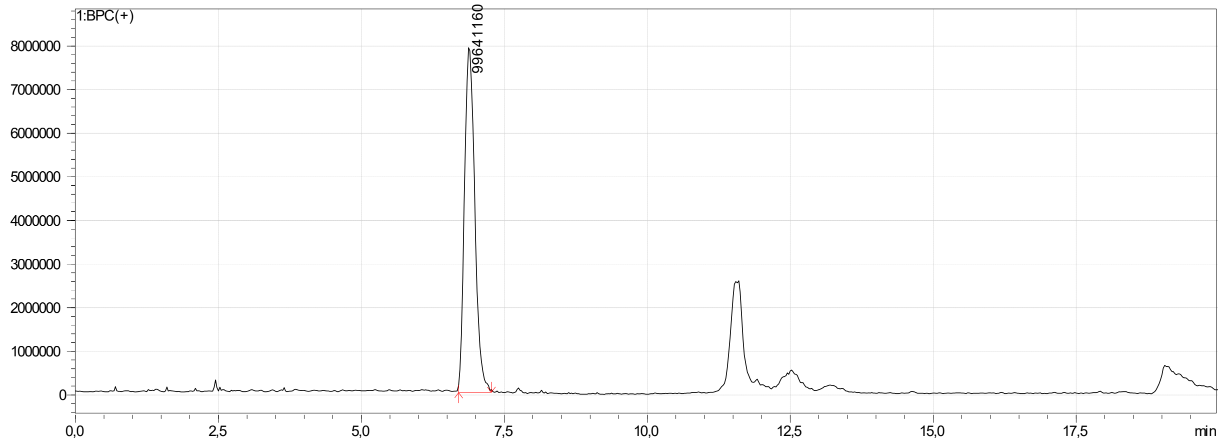

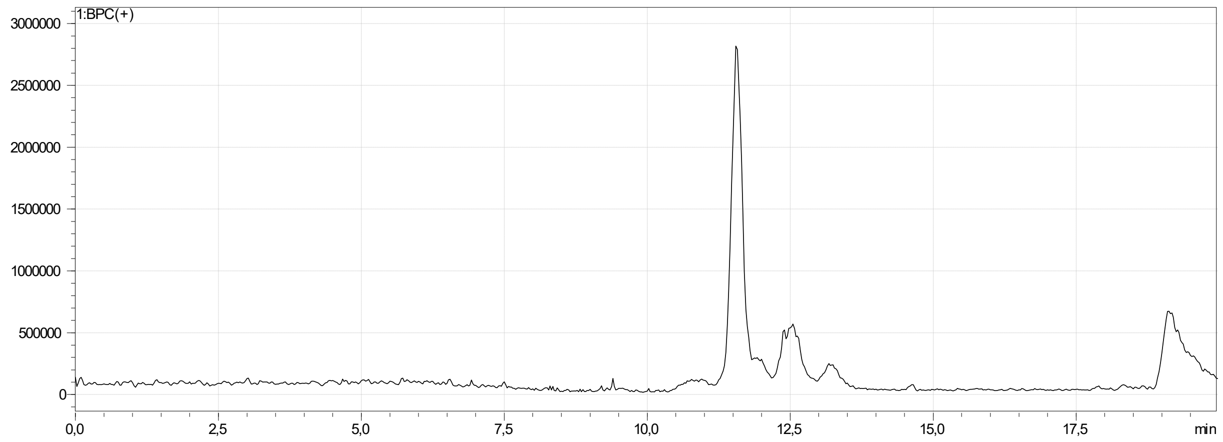

Negative ions (chromatogram and blank)

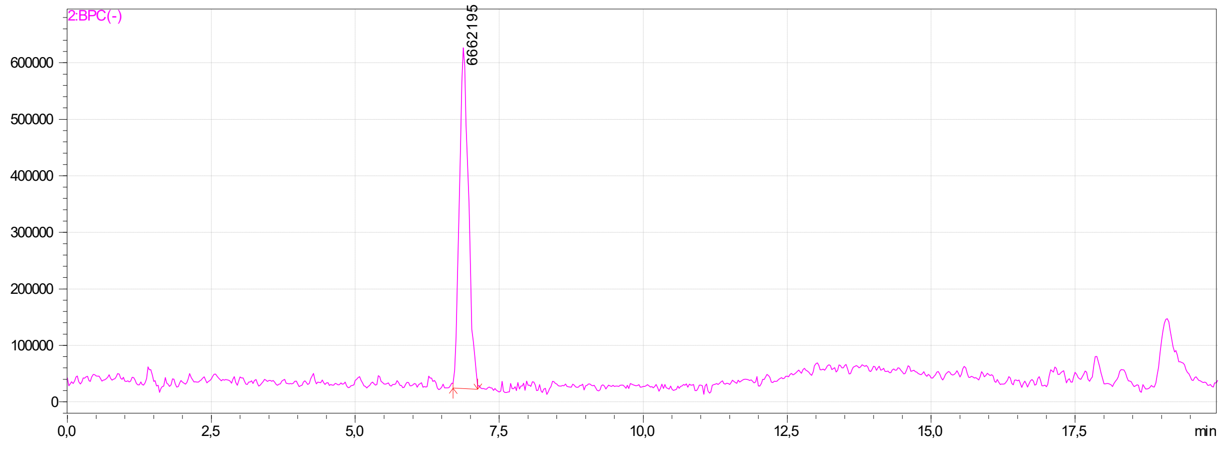

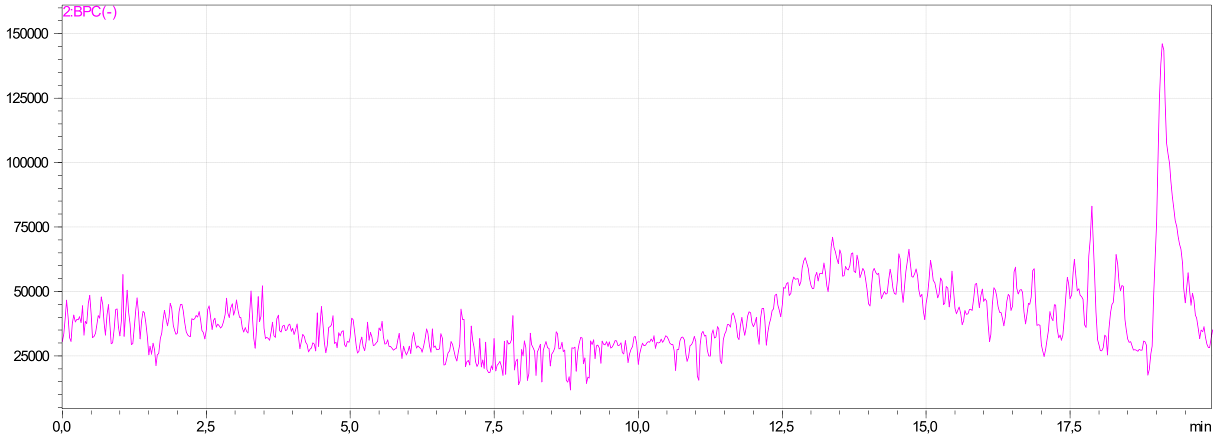

LCMS spectra for compound **8d**

Positive ions spectra for compound **8d**

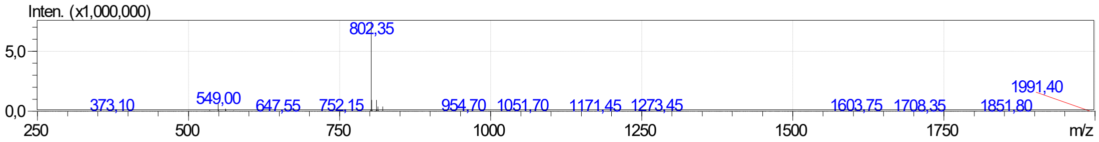

Negative ions spectra for compound **8d**

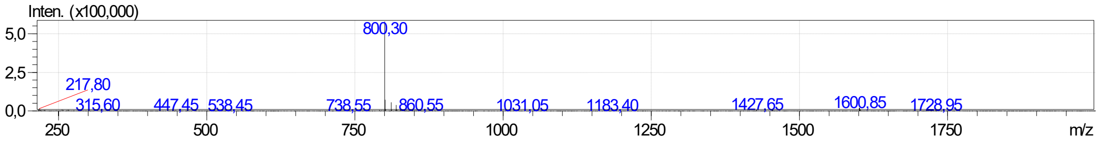

^{1}H NMR spectra for compound **10a**

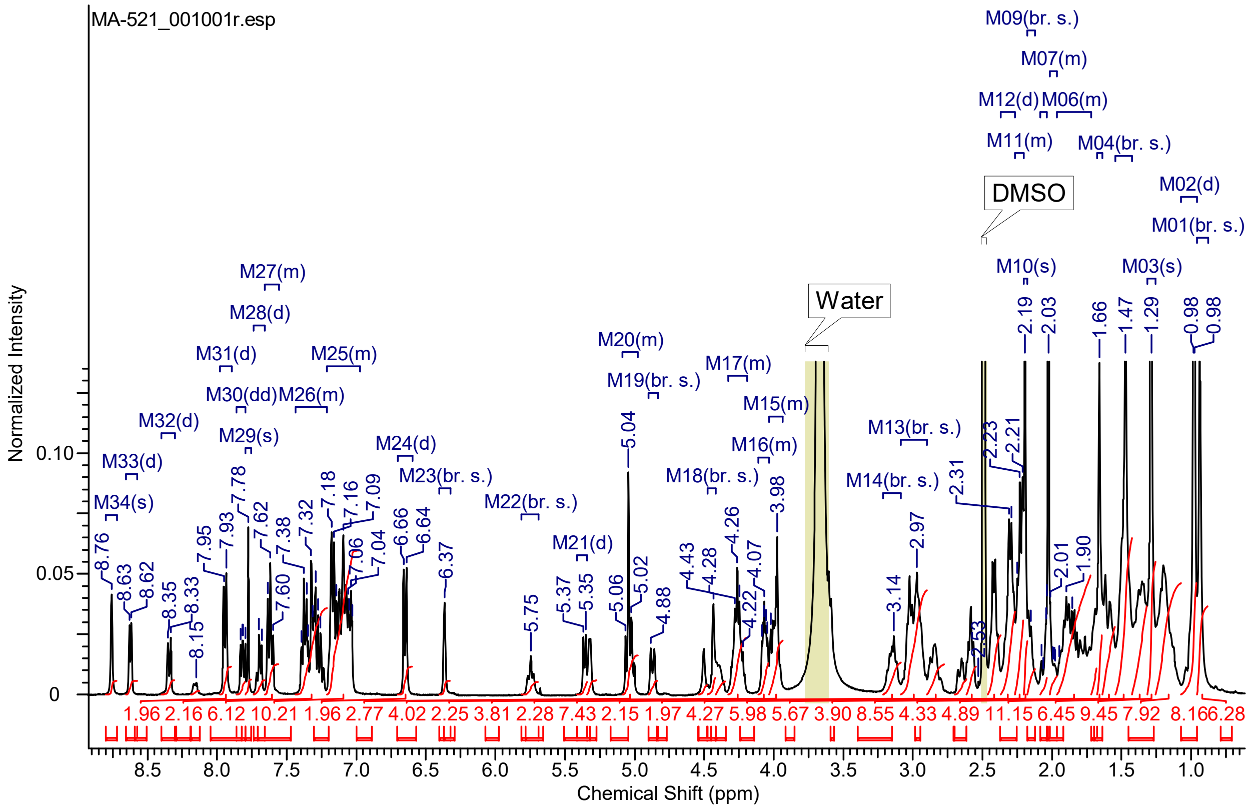

^{13}C NMR spectra for compound **10a**

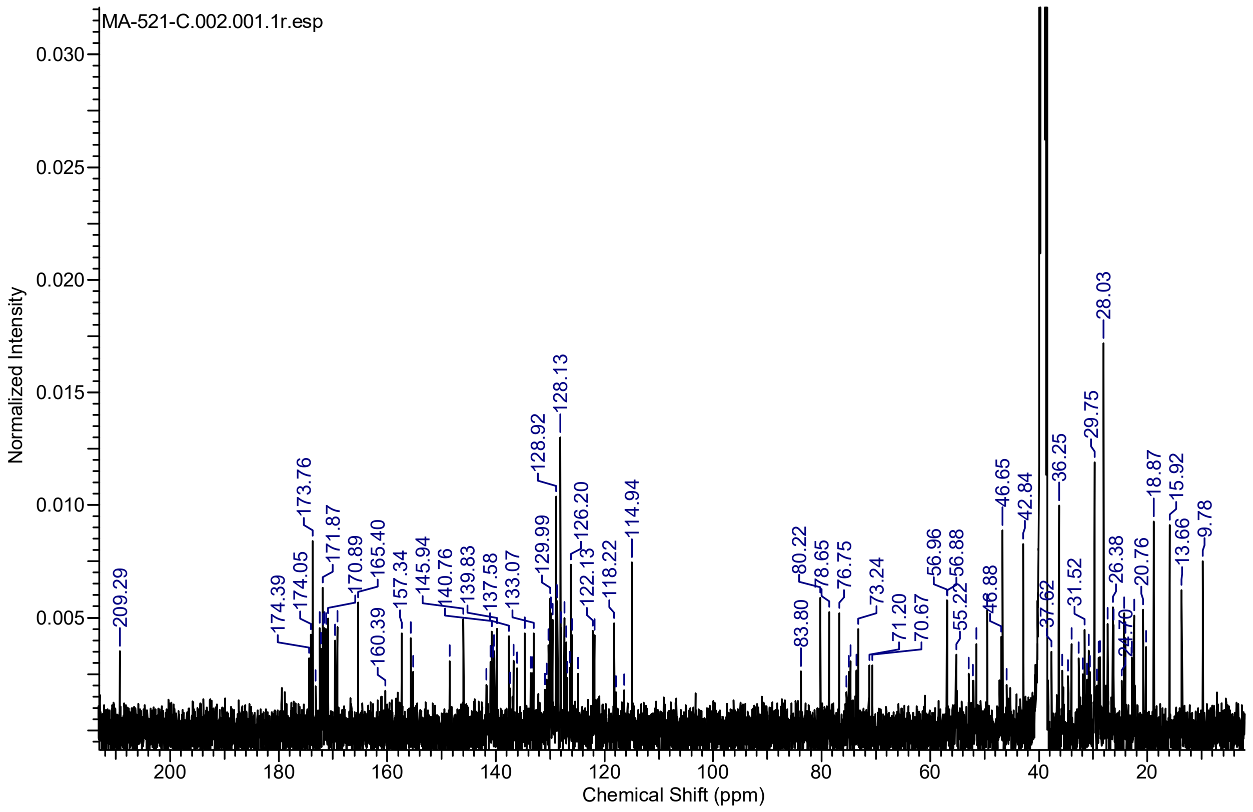

ESI-HRMS for compound **10a**

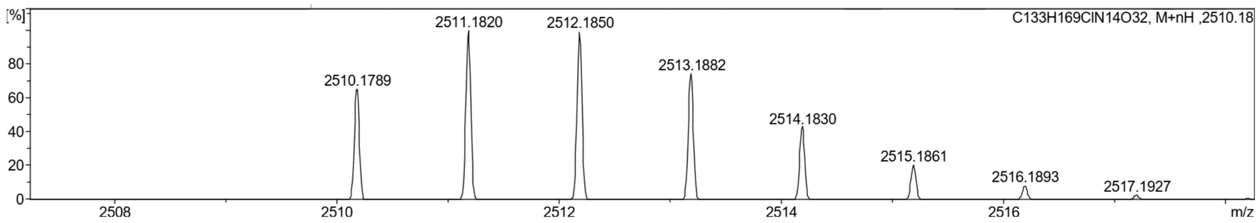

HPLC chromatogram for compound **10a**

Positive ions (chromatogram and blank)

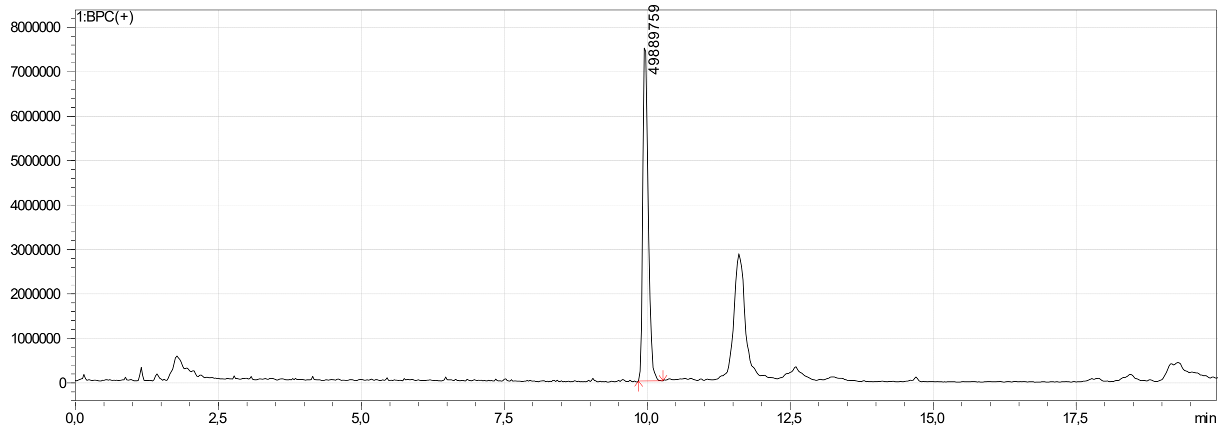

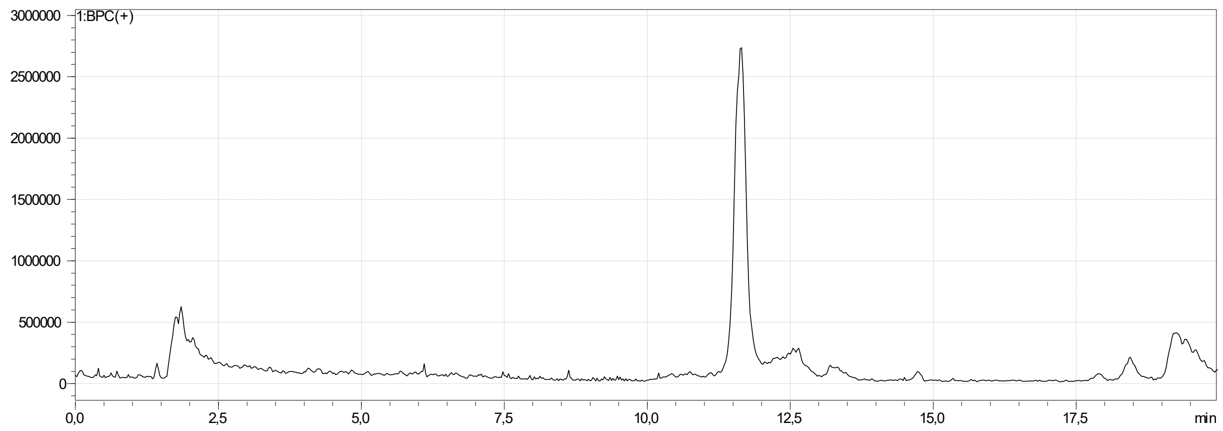

Negative ions (chromatogram and blank)

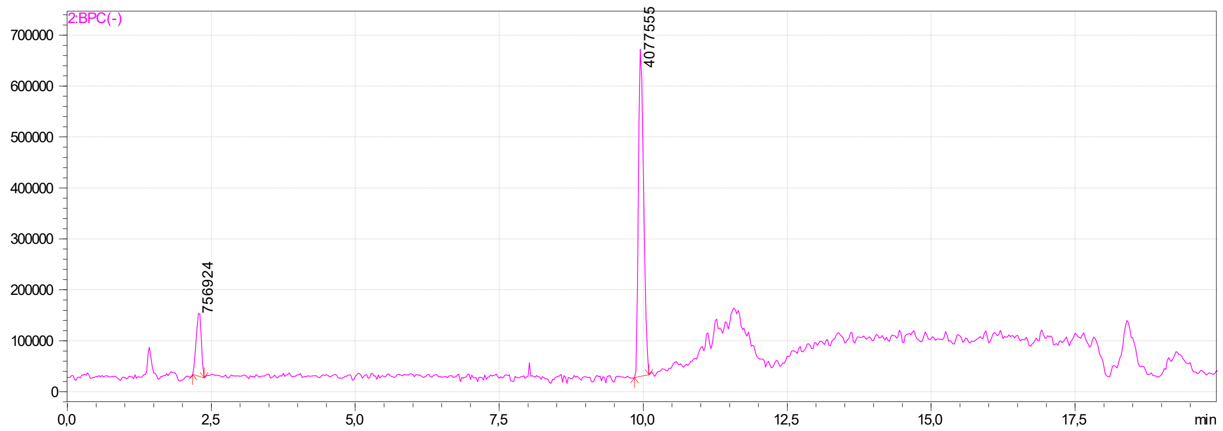

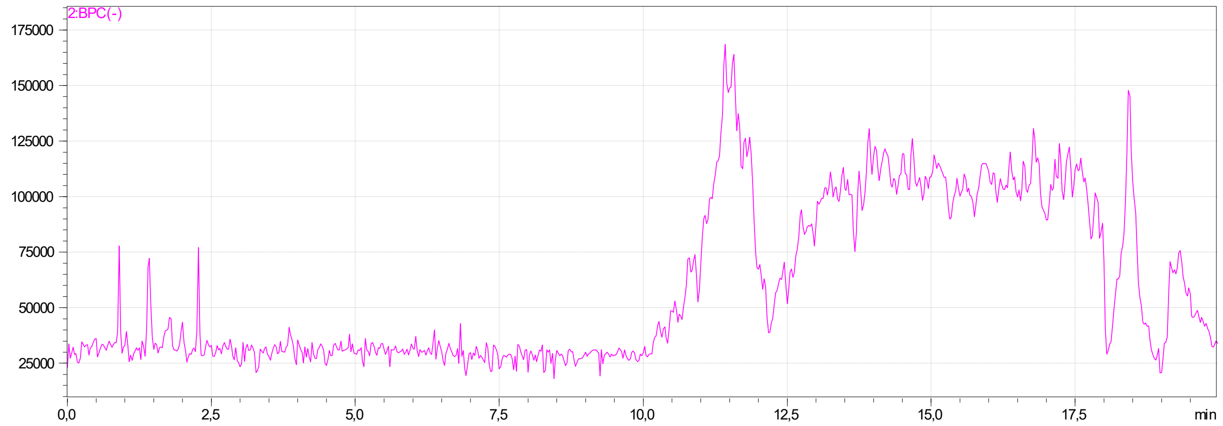

LCMS spectra for compound **10a**

Positive ions spectra for compound **10a**

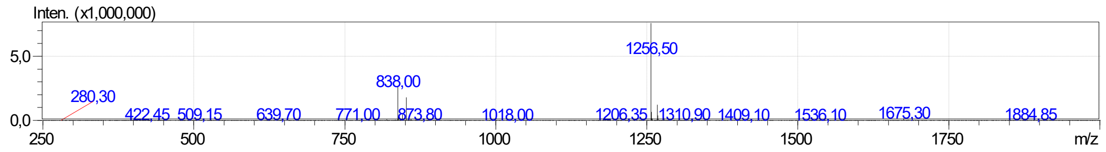

Negative ions spectra for compound **10a**

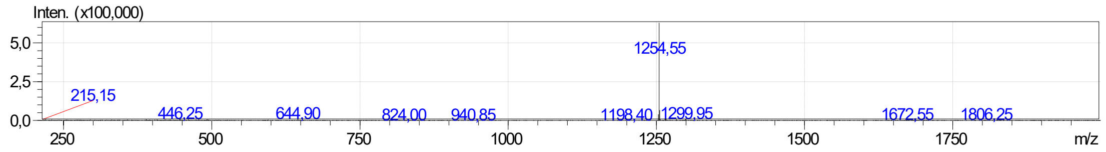

^{1}H NMR spectra for compound **10b**

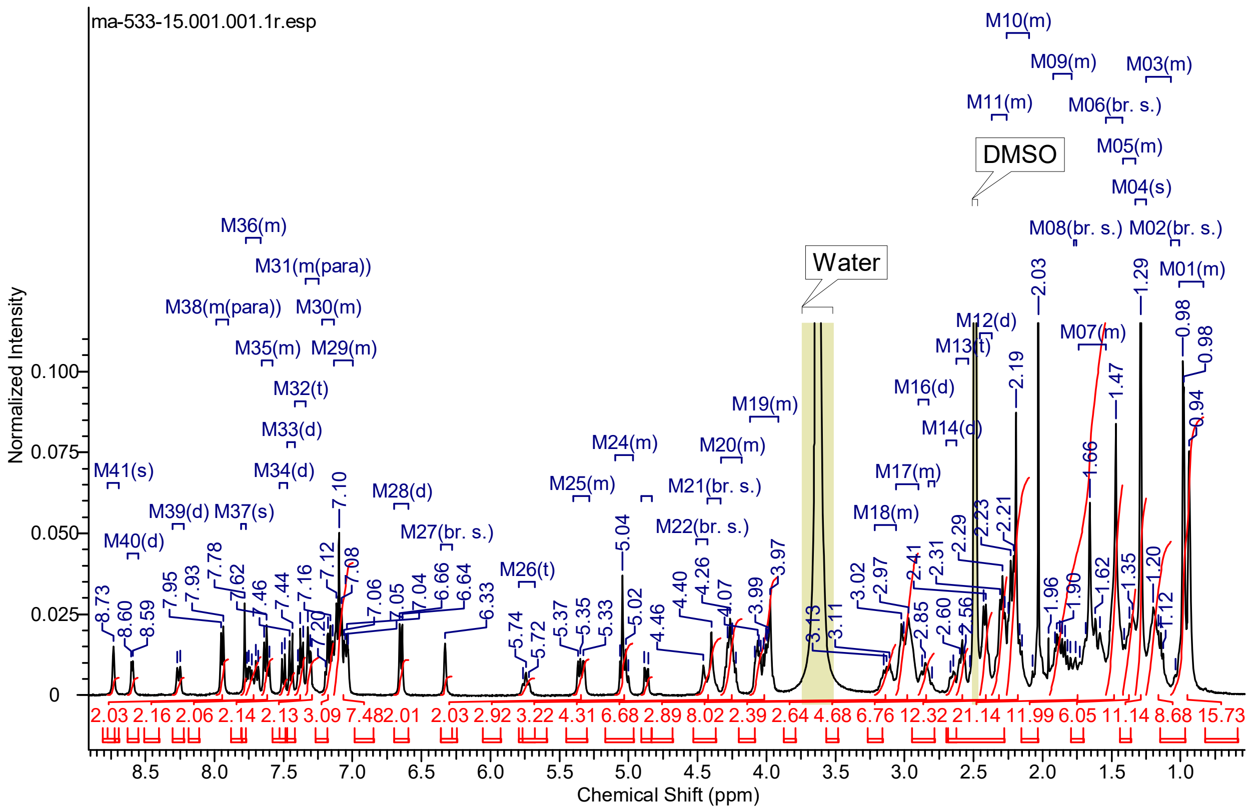

ESI-HRMS for compound **10b**

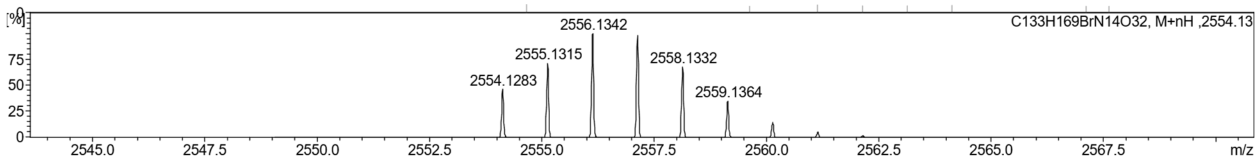

HPLC chromatogram for compound **10b**

Positive ions (chromatogram and blank)

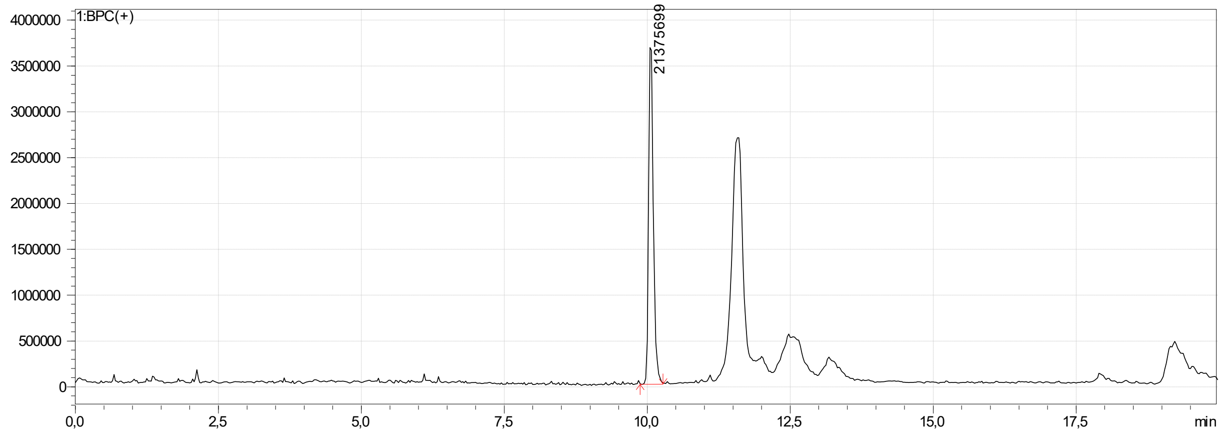

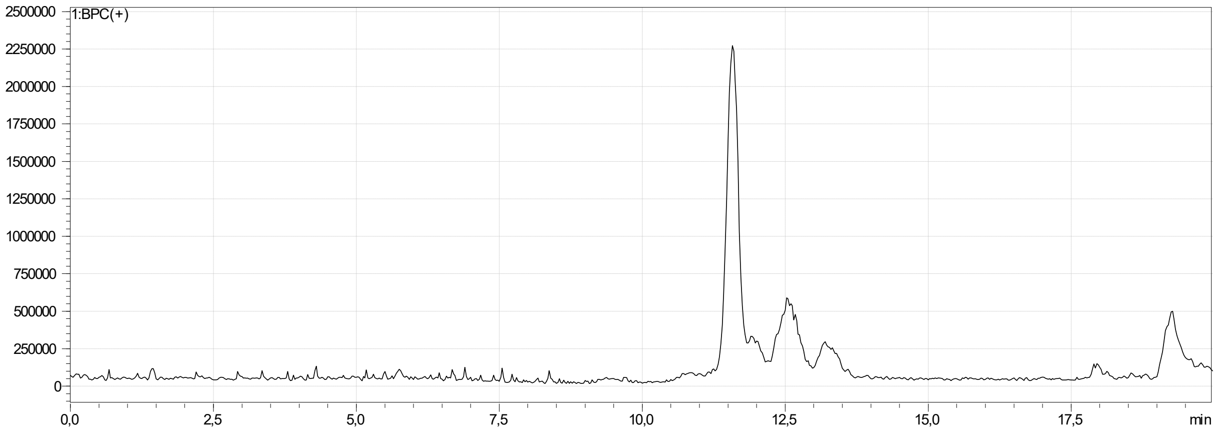

Negative ions (chromatogram and blank)

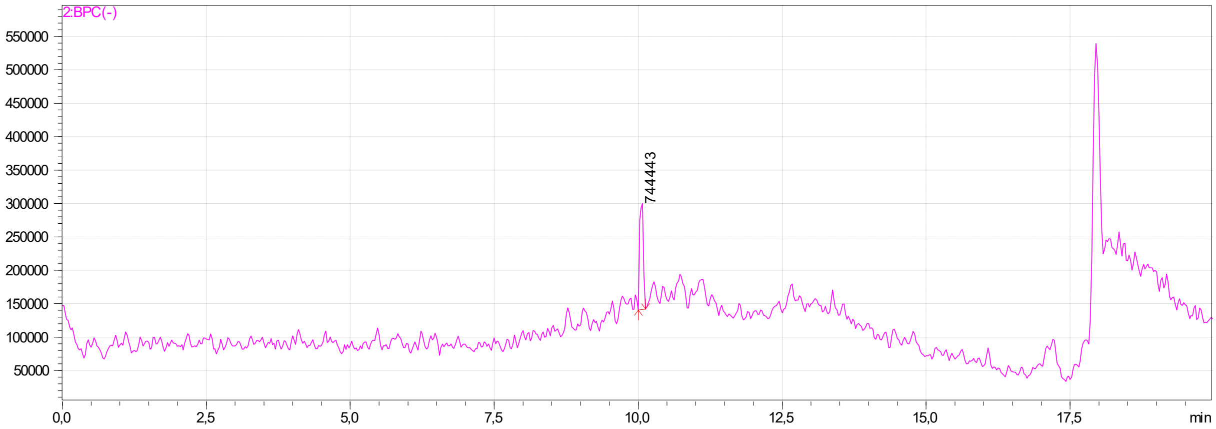

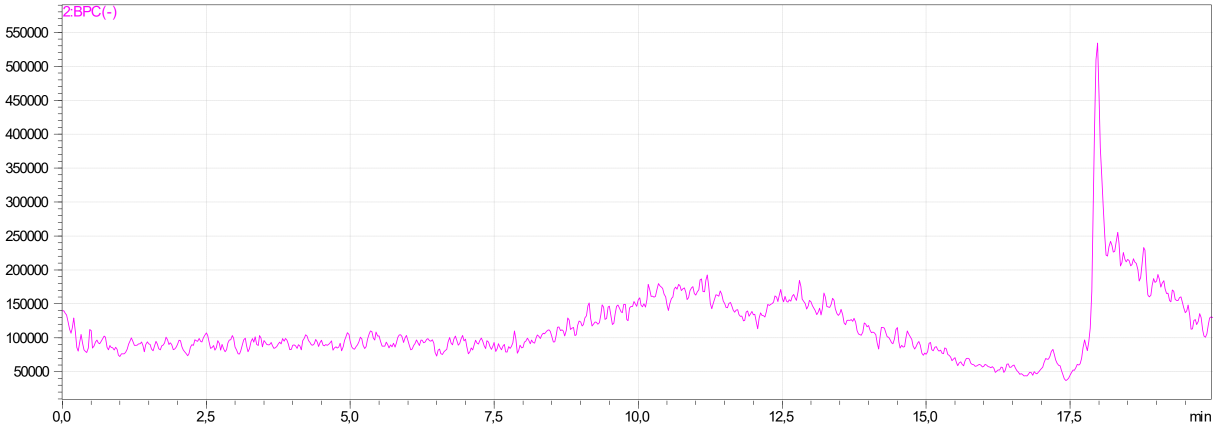

LCMS spectra for compound **10b**

Positive ions spectra for compound **10b**

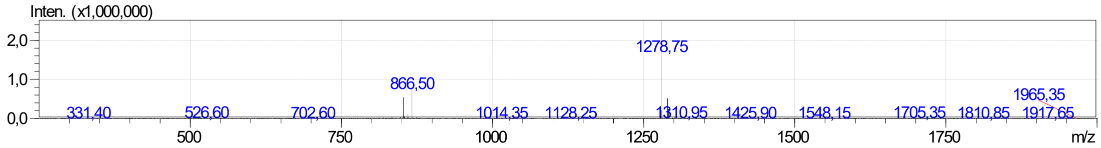

Negative ions spectra for compound **10b**

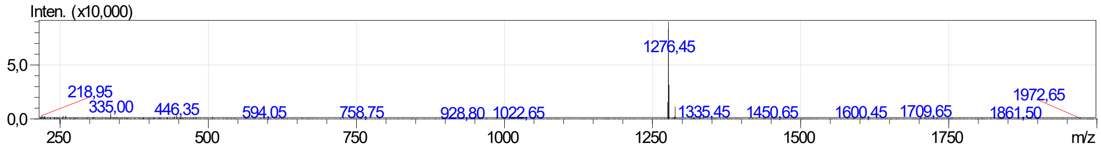

^{1}H NMR spectra for compound **10c**

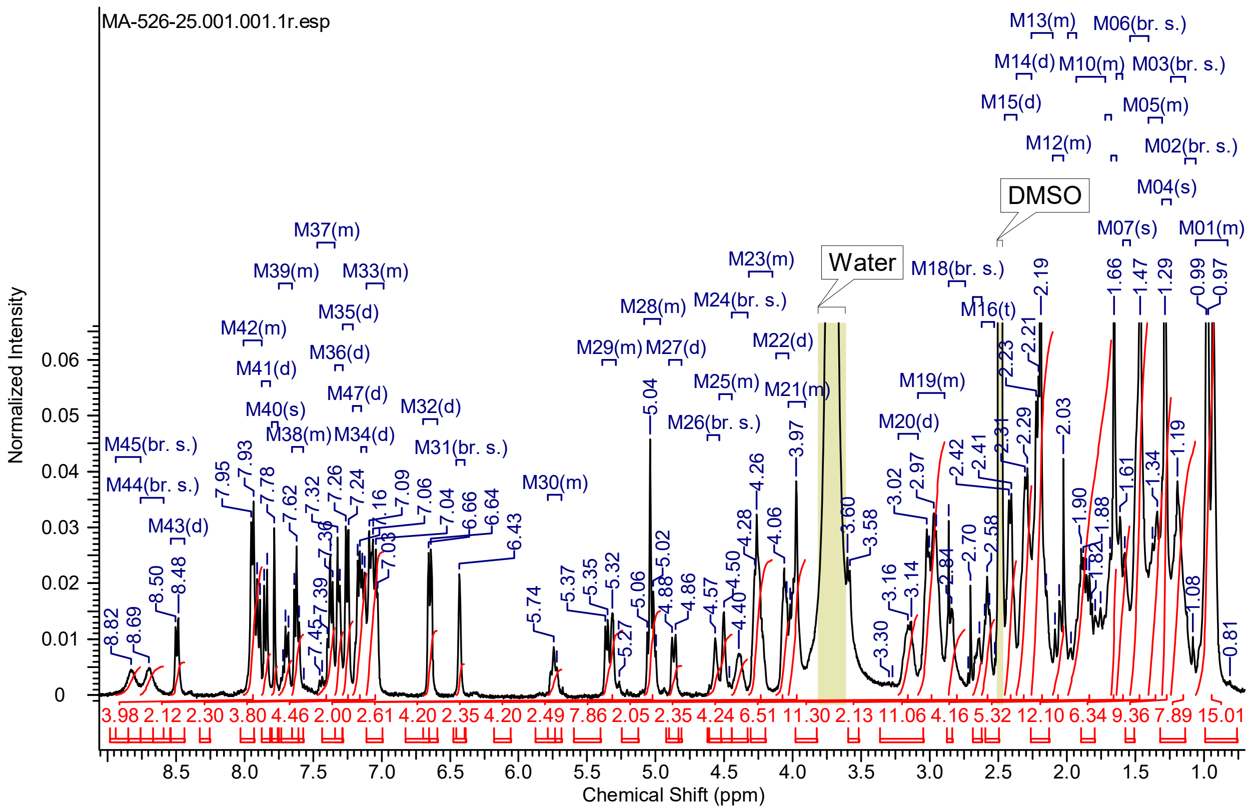

ESI-HRMS for compound **10c**

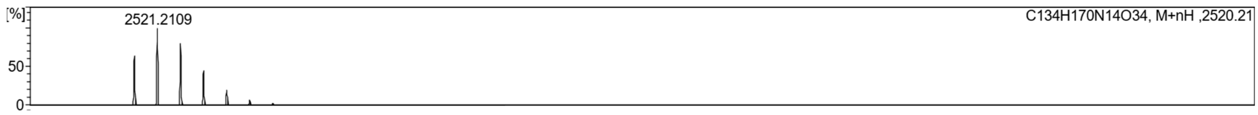

HPLC chromatogram for compound **10c**

Positive ions (chromatogram and blank)

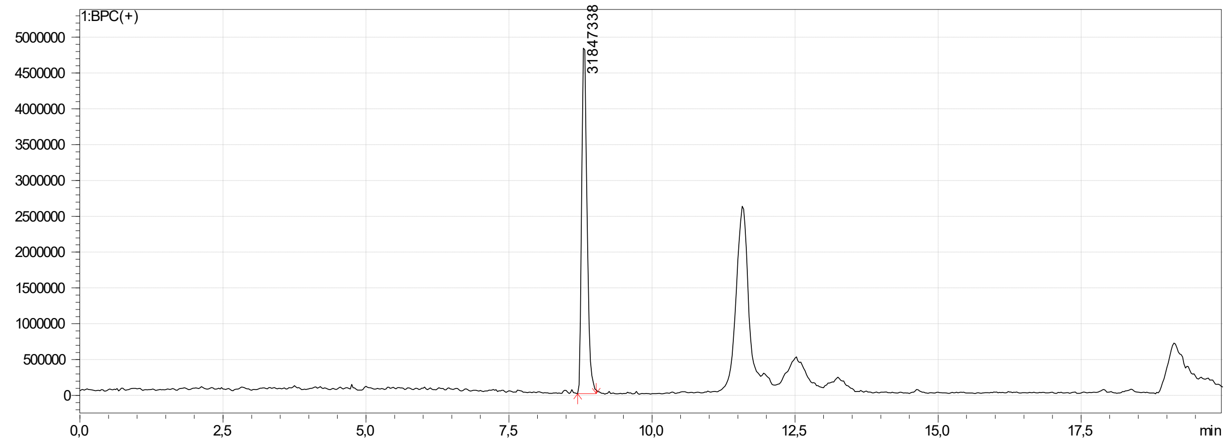

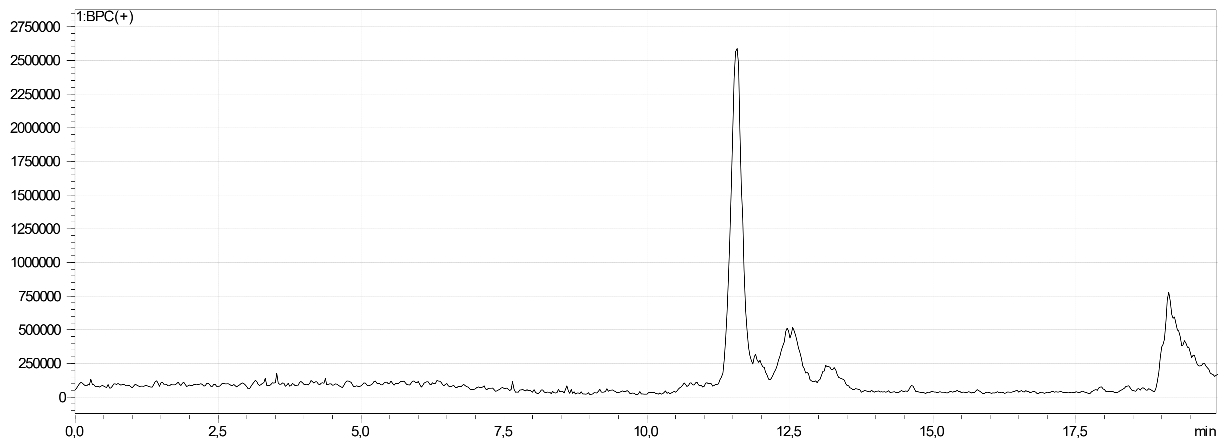

Negative ions (chromatogram and blank)

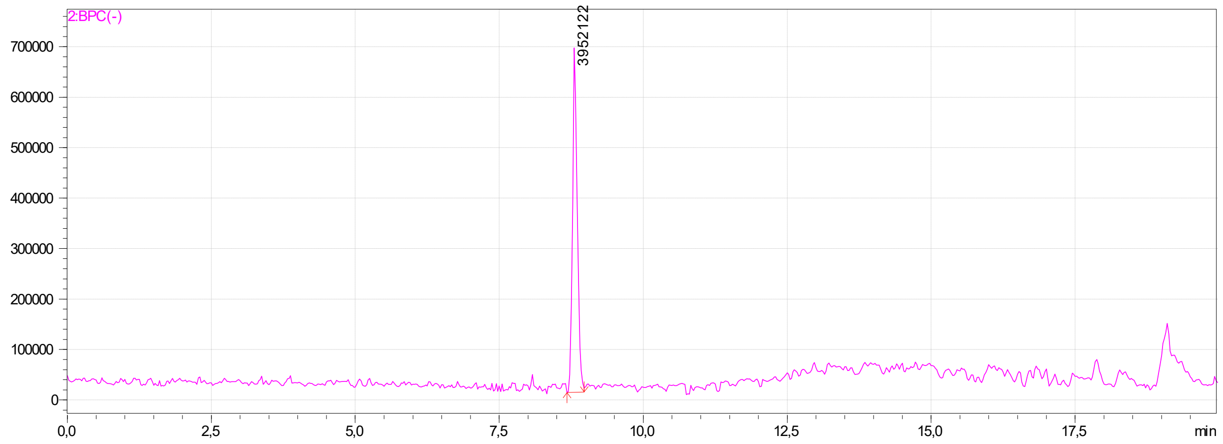

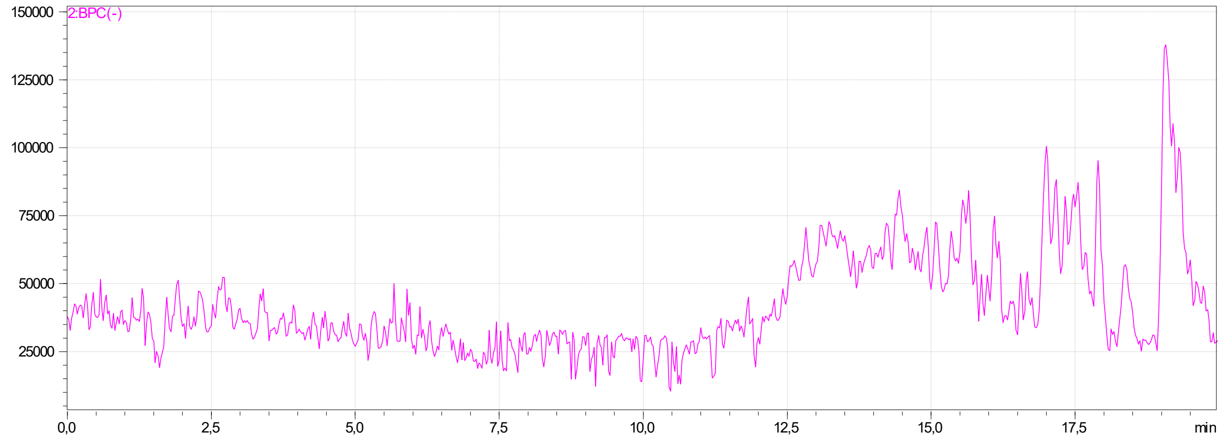

LCMS spectra for compound **10c**

Positive ions spectra for compound **10c**

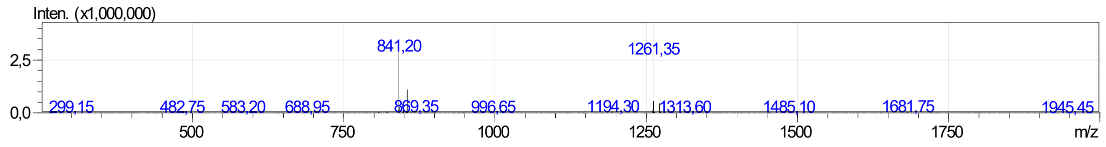

Negative ions spectra for compound **10c**

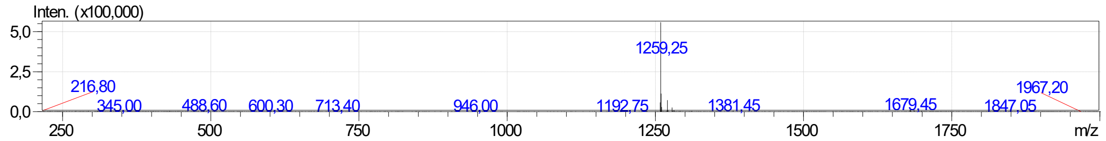

^{1}H NMR spectra for compound **10d**

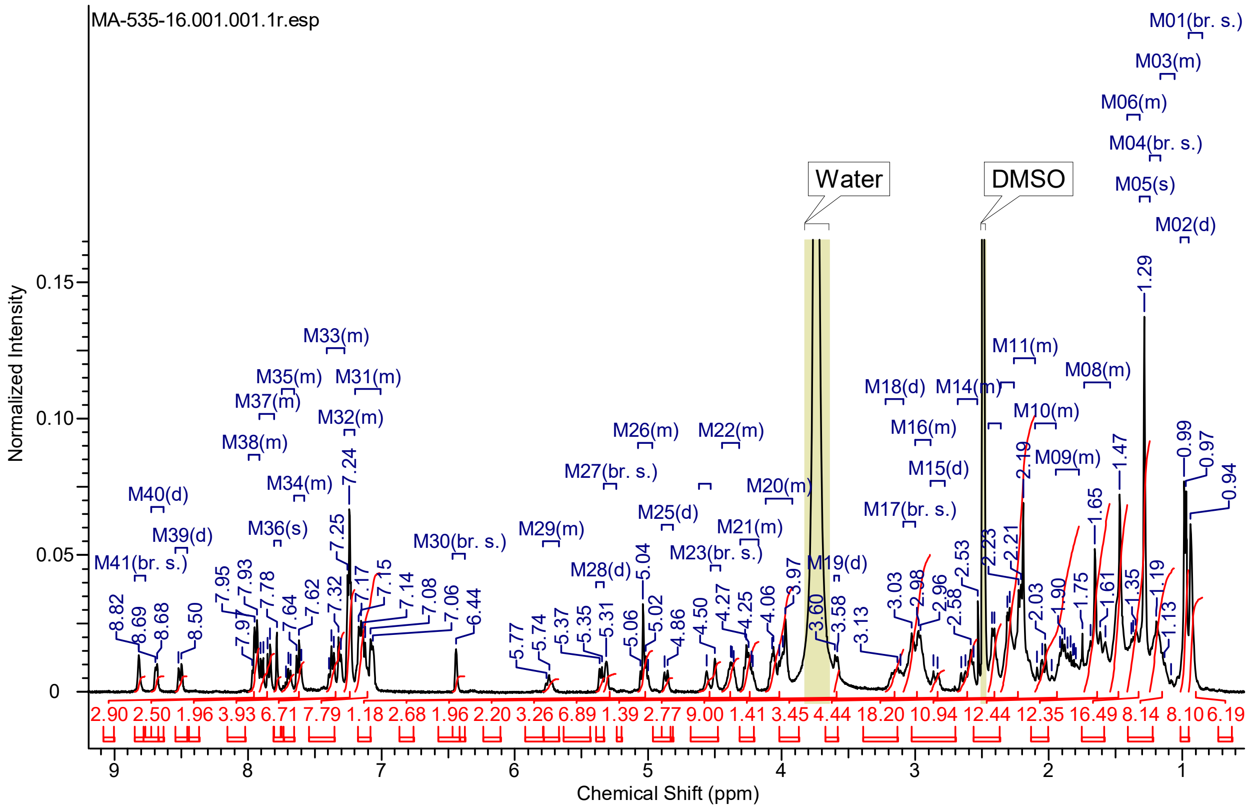

ESI-HRMS for compound **10d**

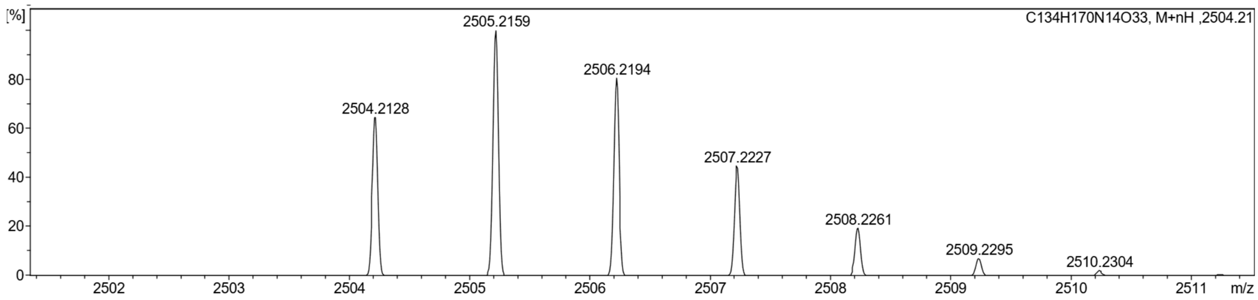

HPLC chromatogram for compound **10d**

Positive ions (chromatogram and blank)

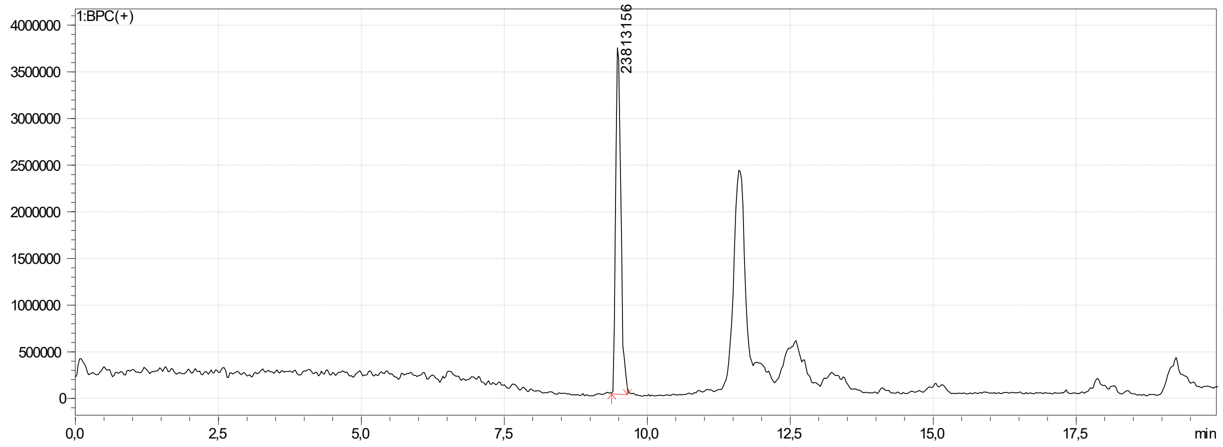

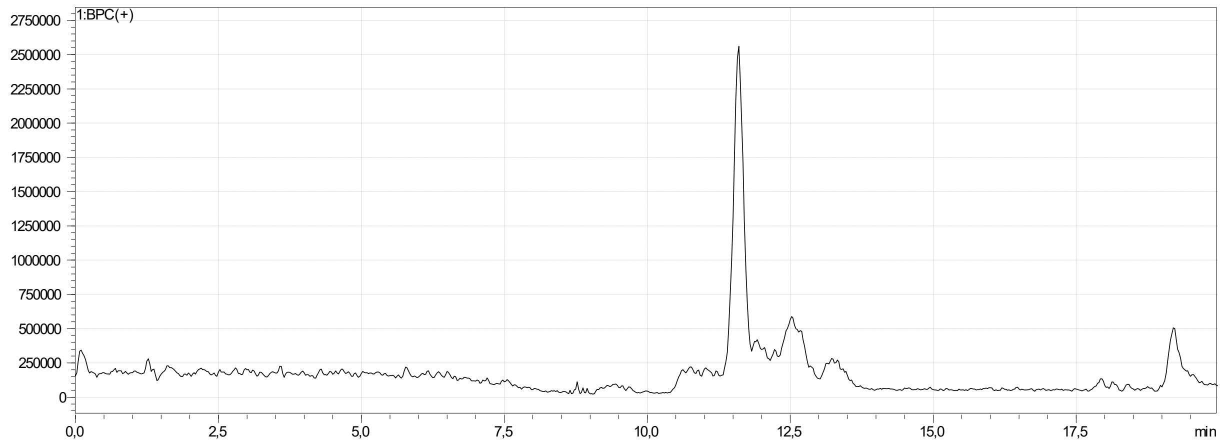

Negative ions (chromatogram and blank)

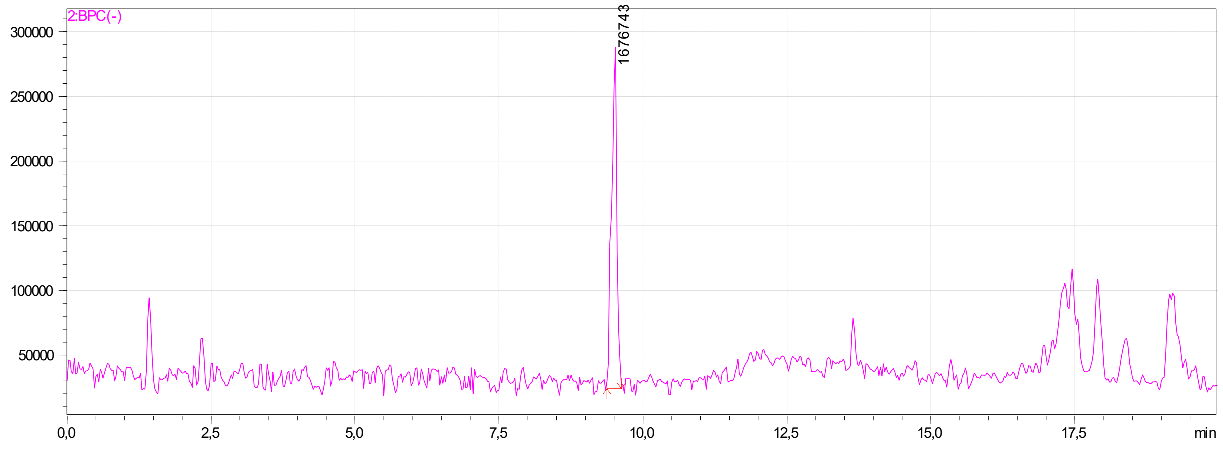

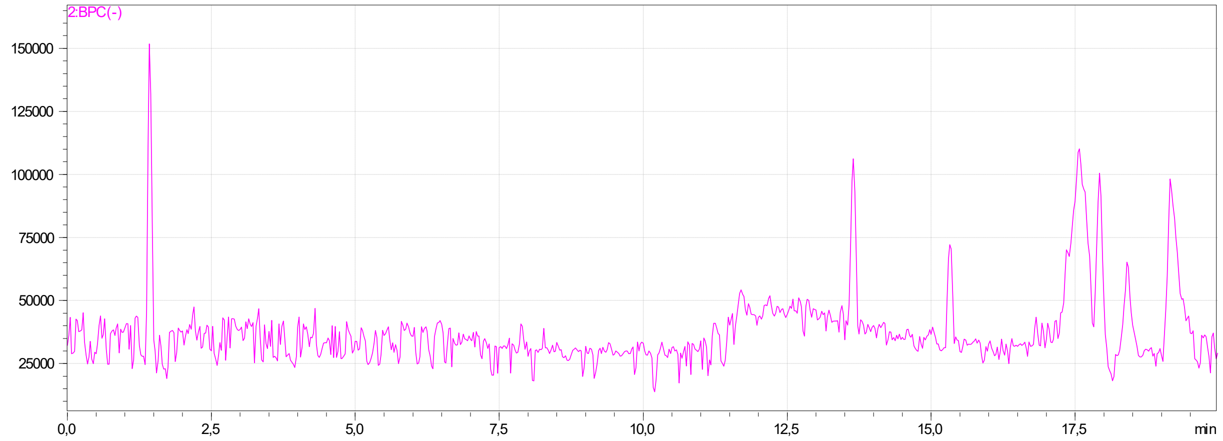

LCMS spectra for compound **10d**

Positive ions spectra for compound **10d**

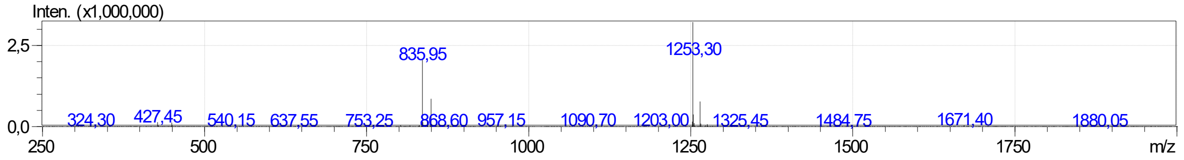

Negative ions spectra for compound **10d**

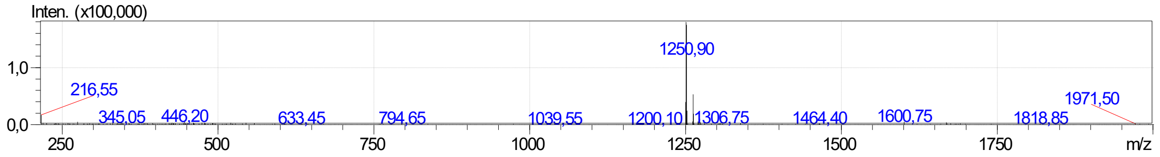

^{1}H NMR spectra for compound **12a**

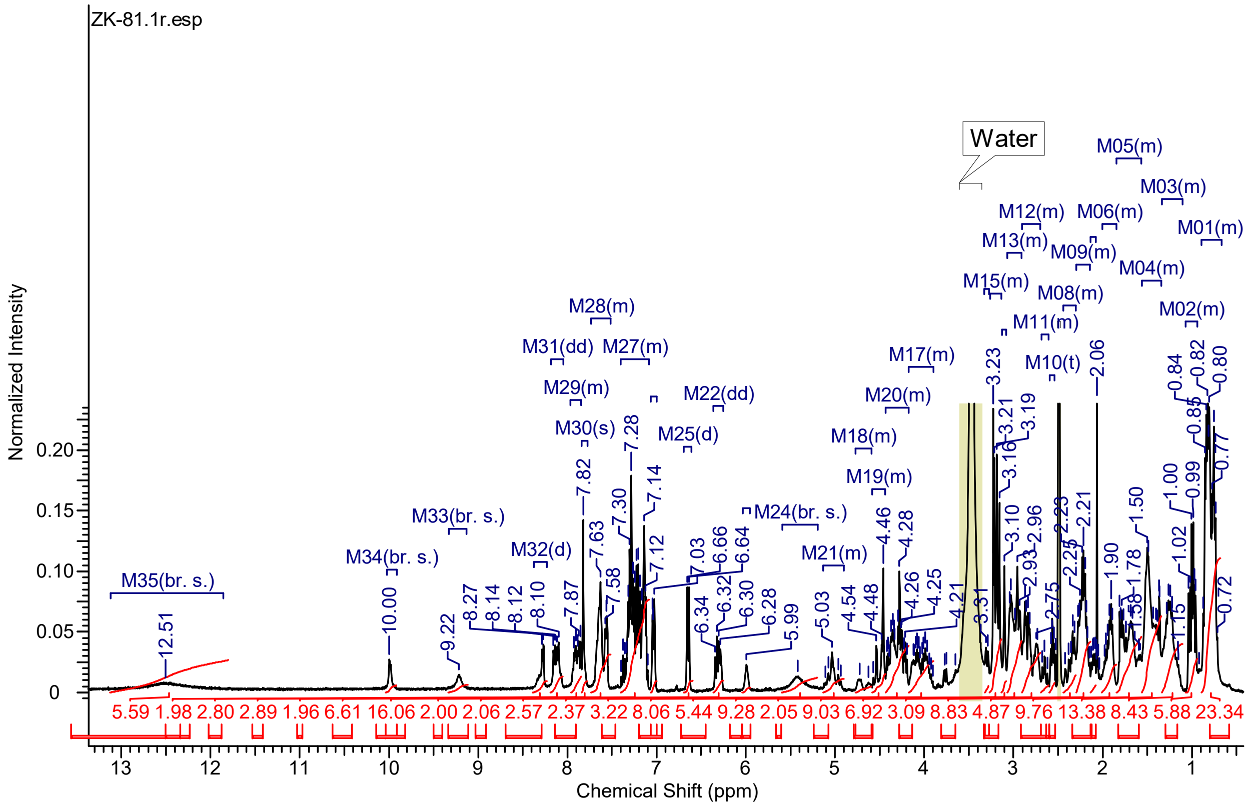

^{13}C NMR spectra for compound **12a**

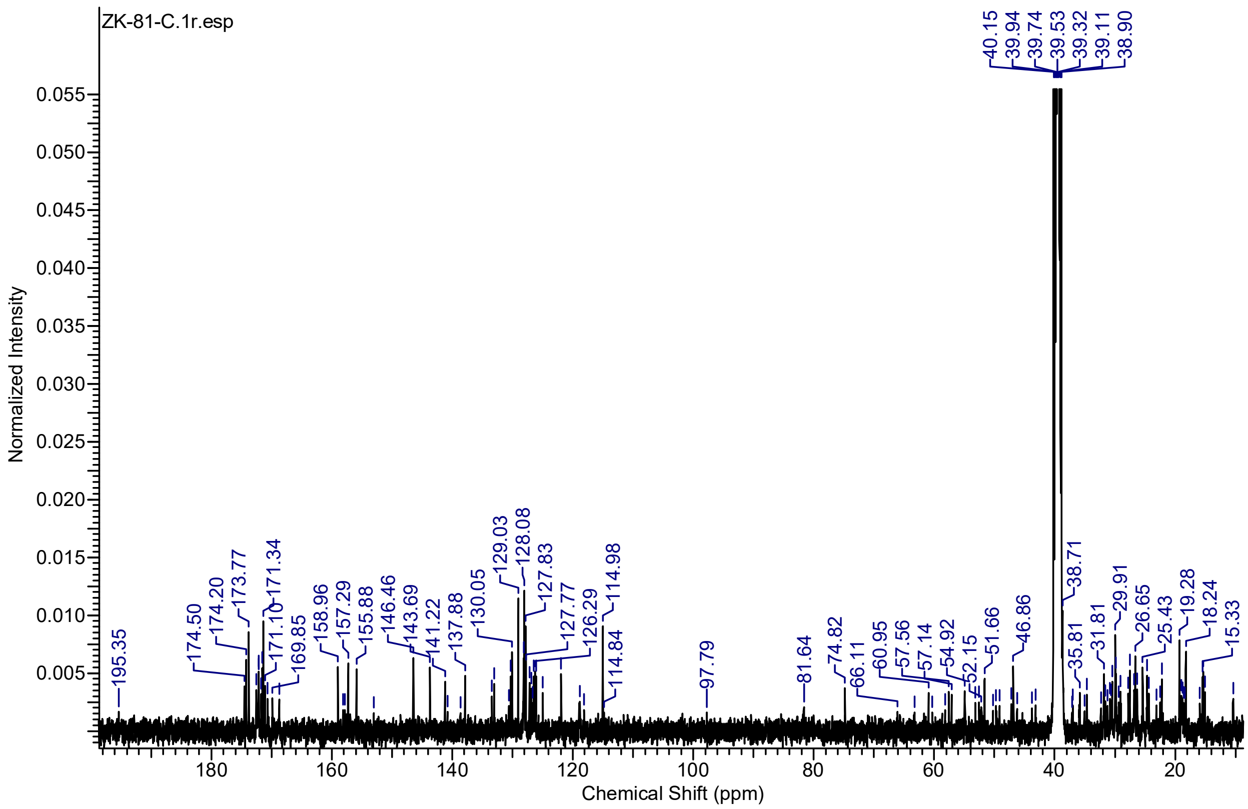

ESI-HRMS for compound **12a**

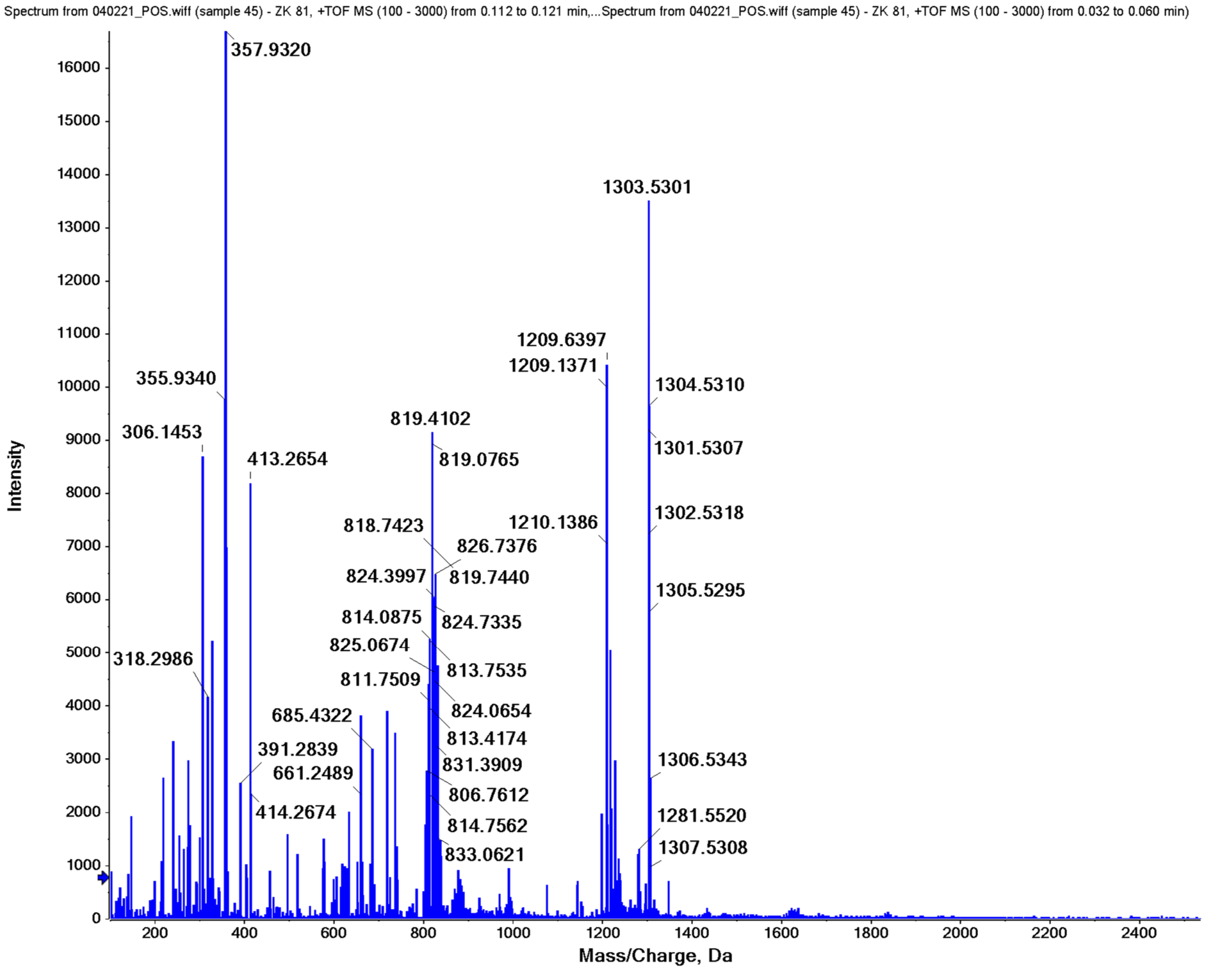

HPLC chromatogram for compound **12a**

Positive ions (chromatogram and blank)

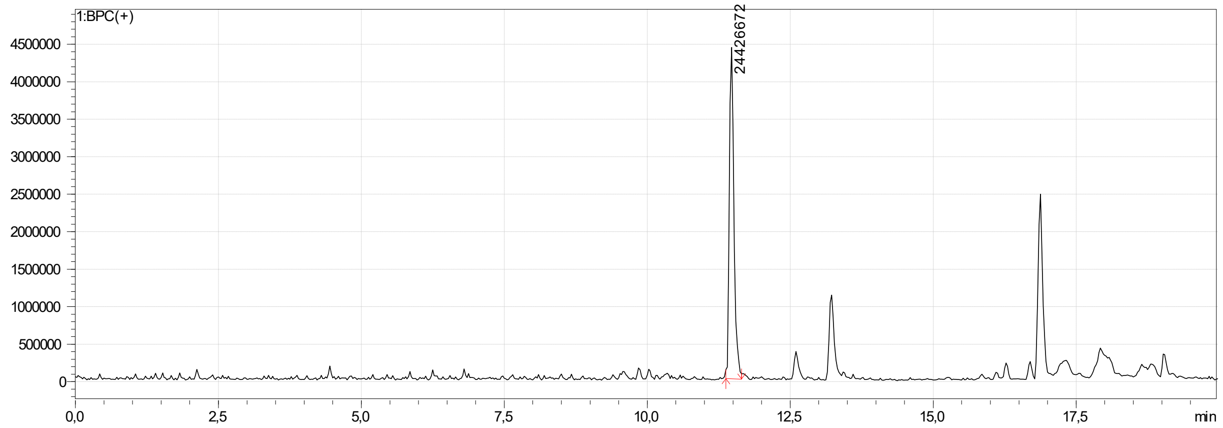

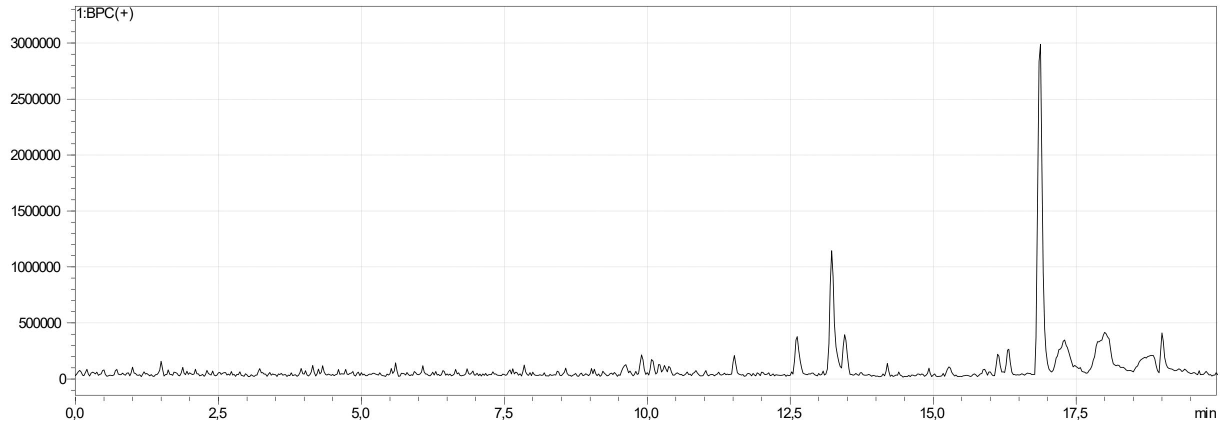

Negative ions (chromatogram and blank)

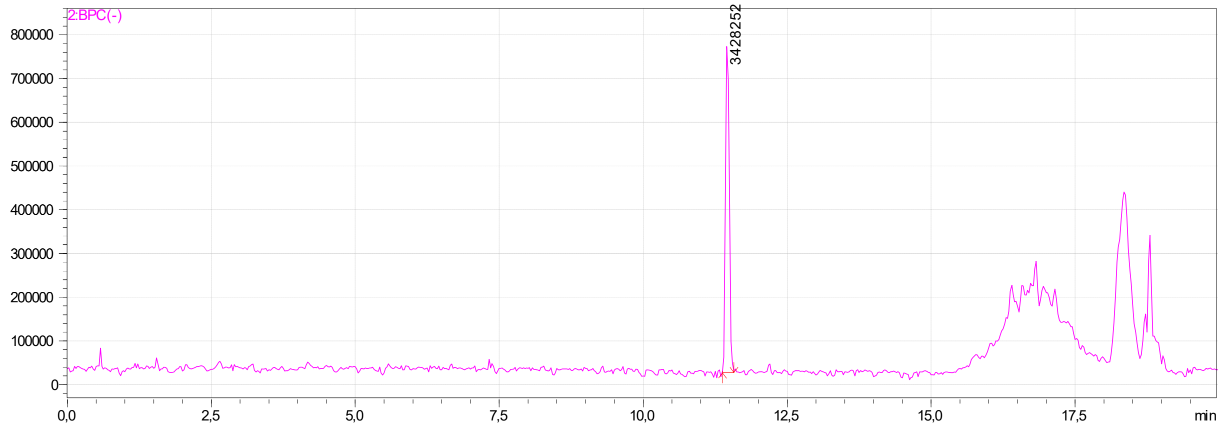

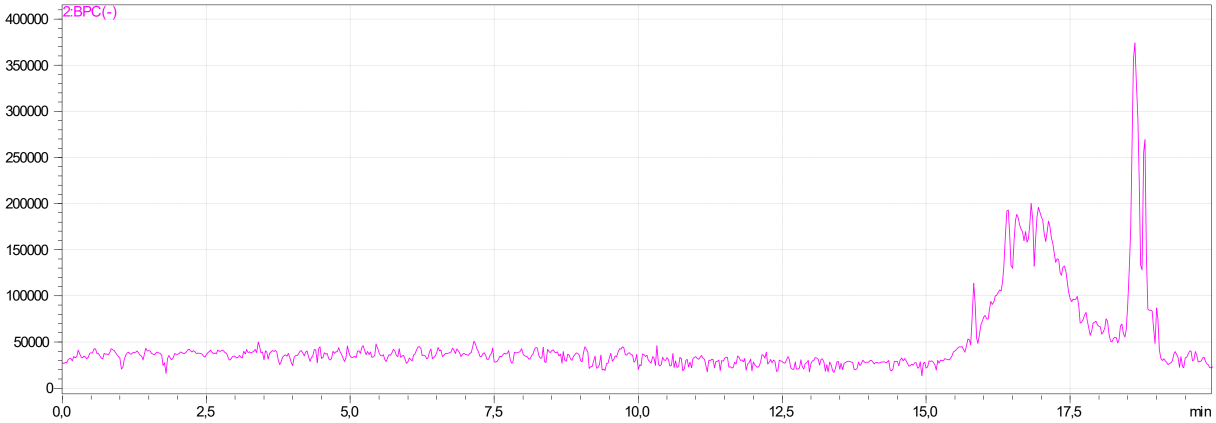

LCMS spectra for compound **12a**

Positive ions spectra for compound **12a**

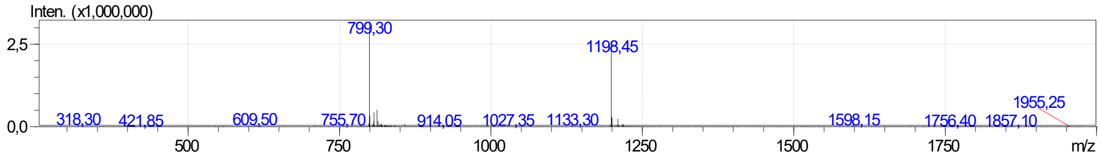

Negative ions spectra for compound **12a**

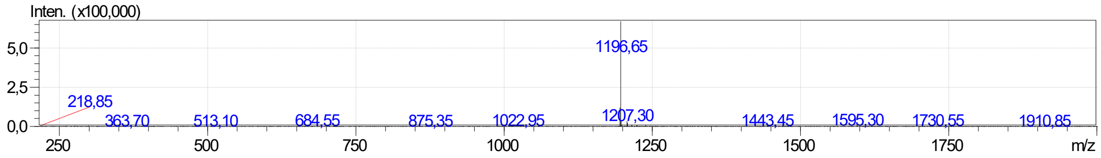

^{1}H NMR spectra for compound **12b**

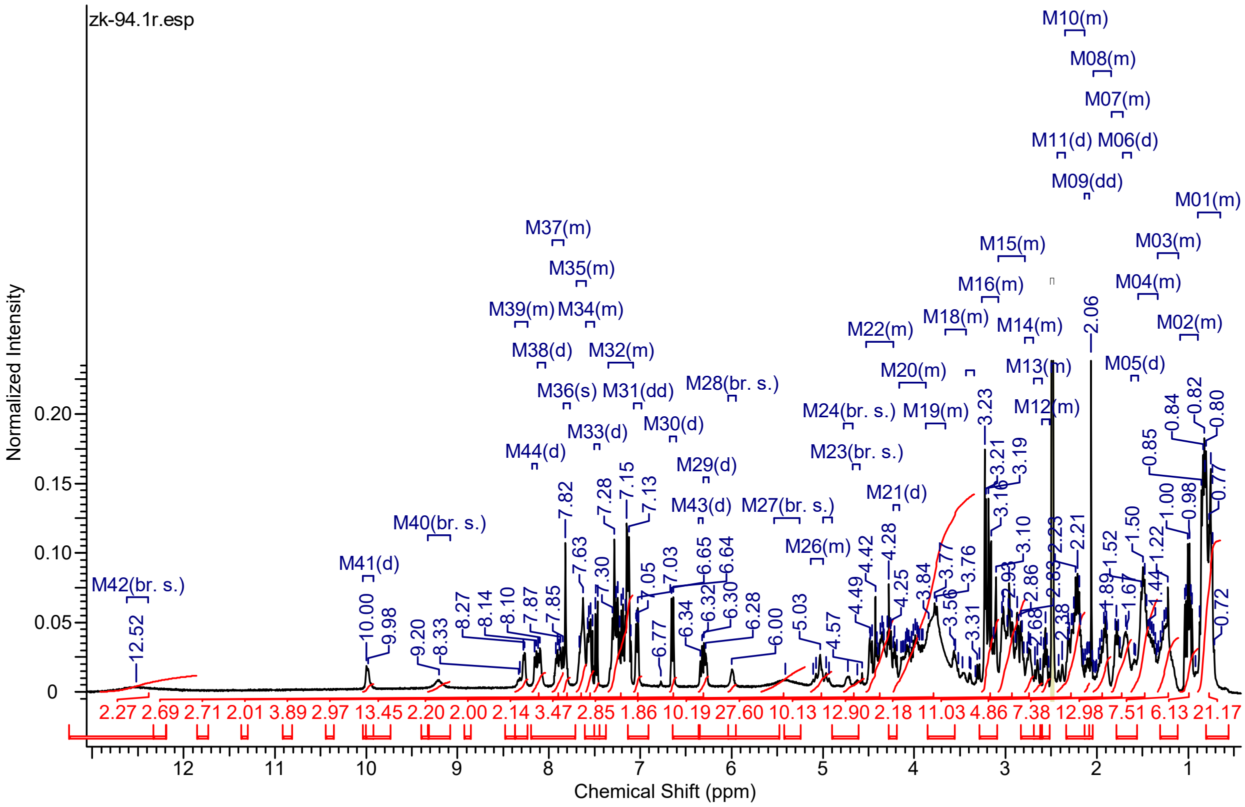

^{13}C NMR spectra for compound **12b**

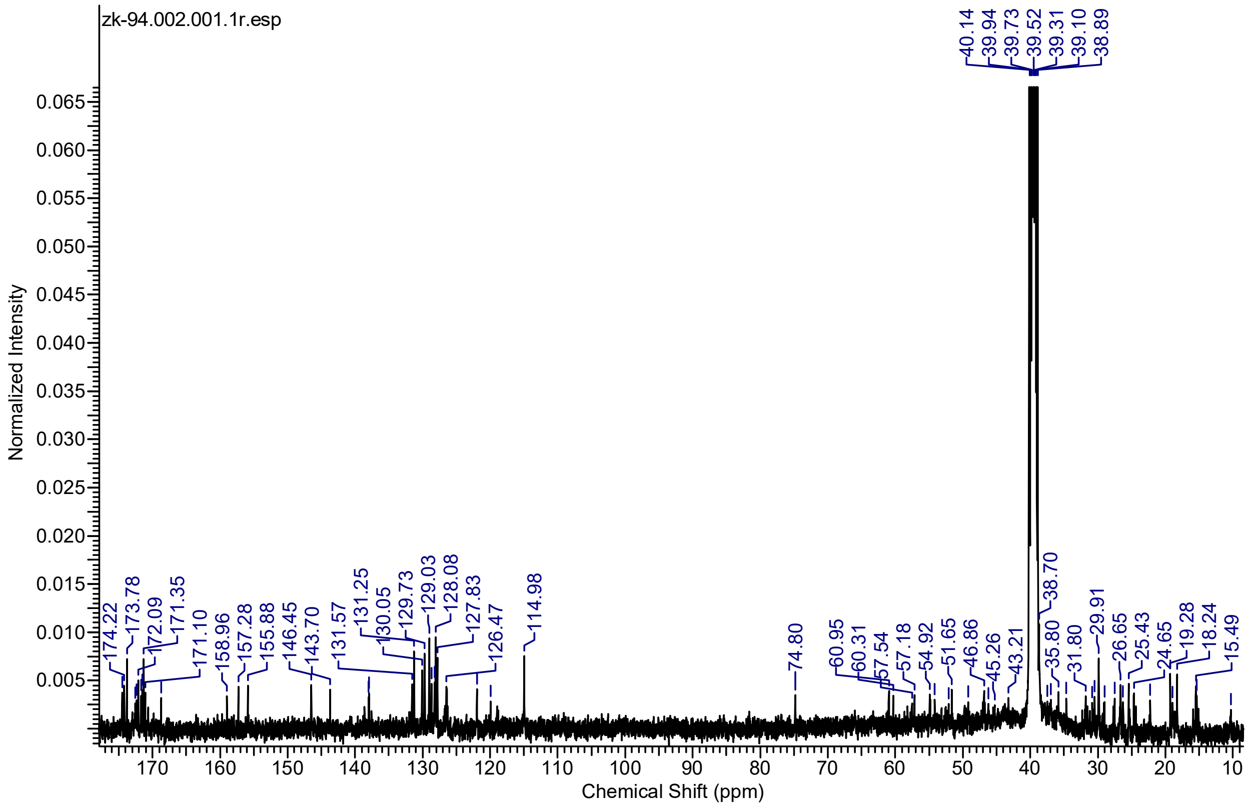

ESI-HRMS for compound **12b**

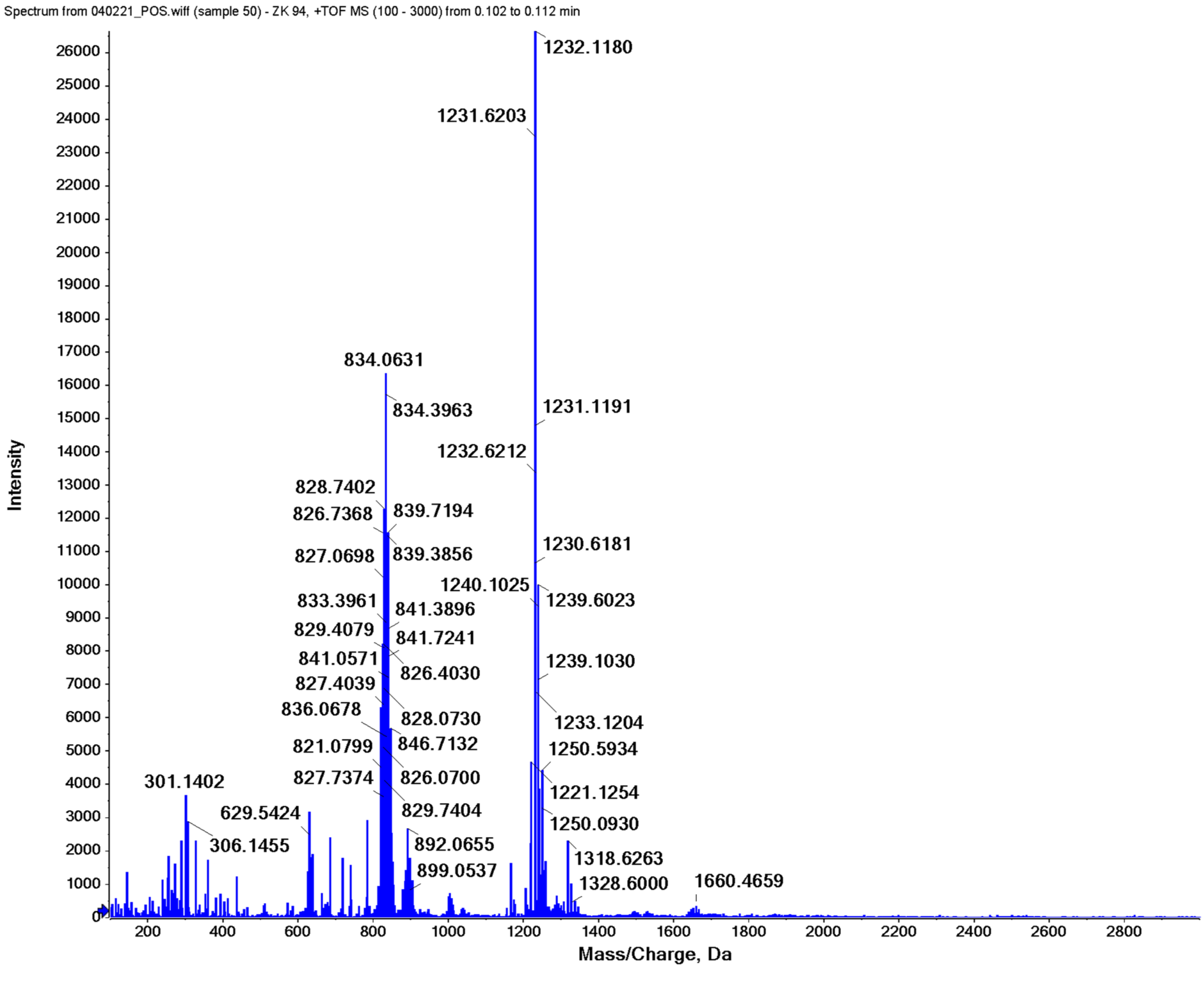

HPLC chromatogram for compound **12b**

Positive ions (chromatogram and blank)

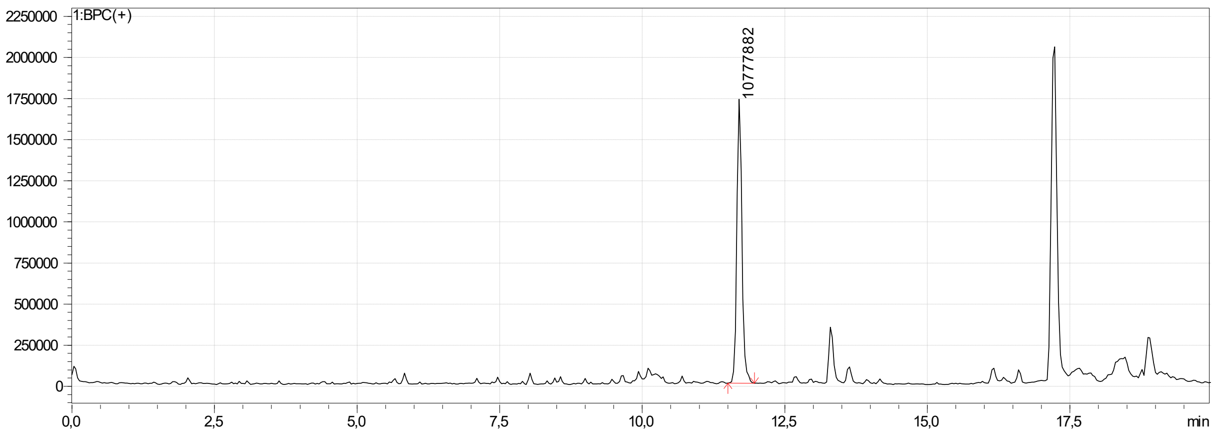

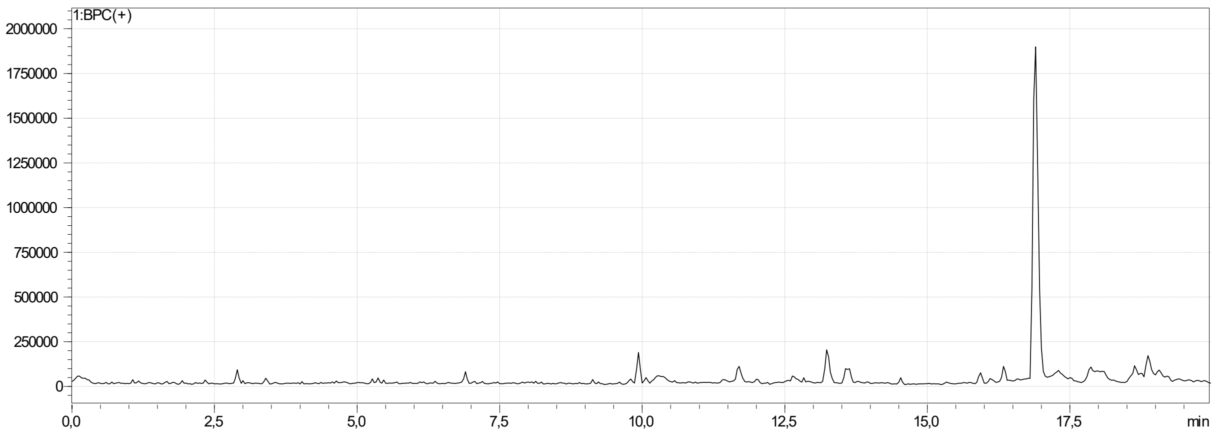

Negative ions (chromatogram and blank)

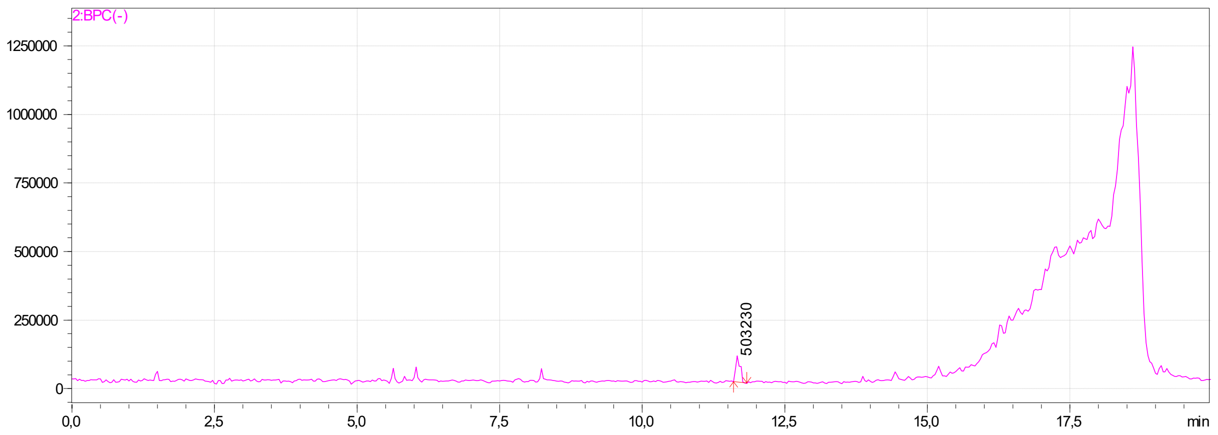

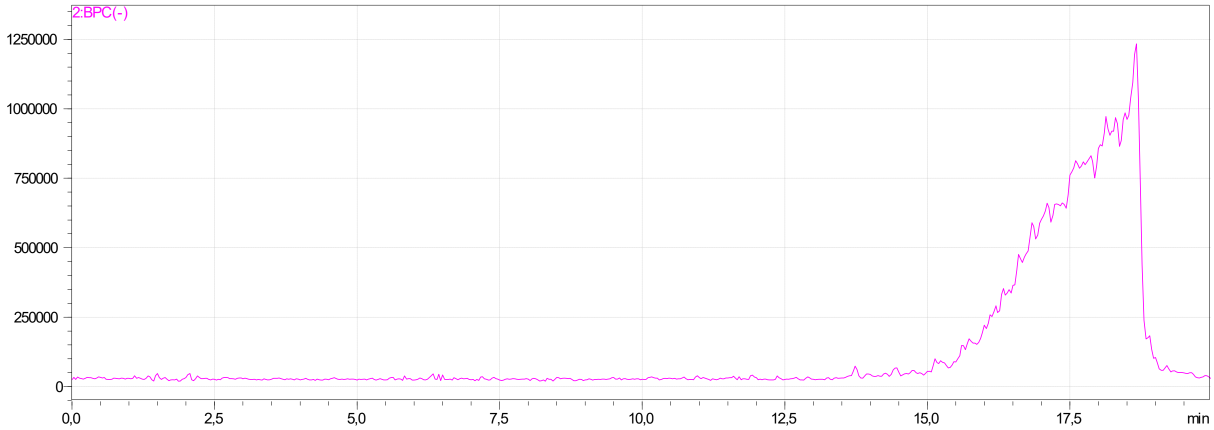

LCMS spectra for compound **12b**

Positive ions spectra for compound **12b**

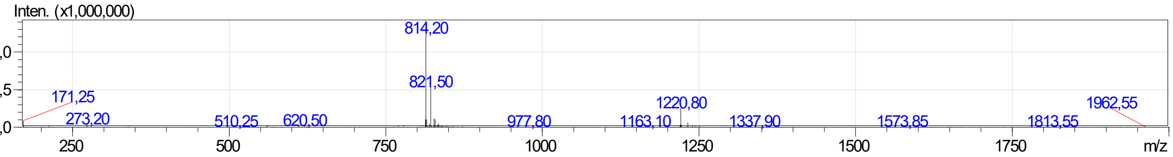

Negative ions spectra for compound **12b**

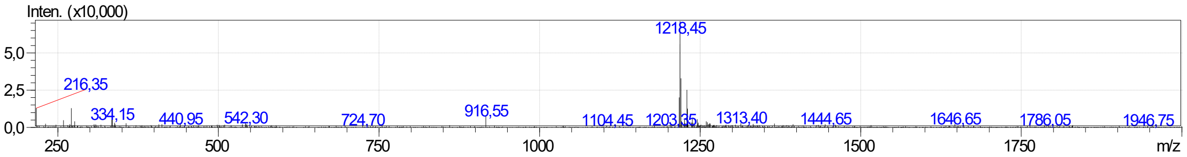

^{1}H NMR spectra for compound **12c**

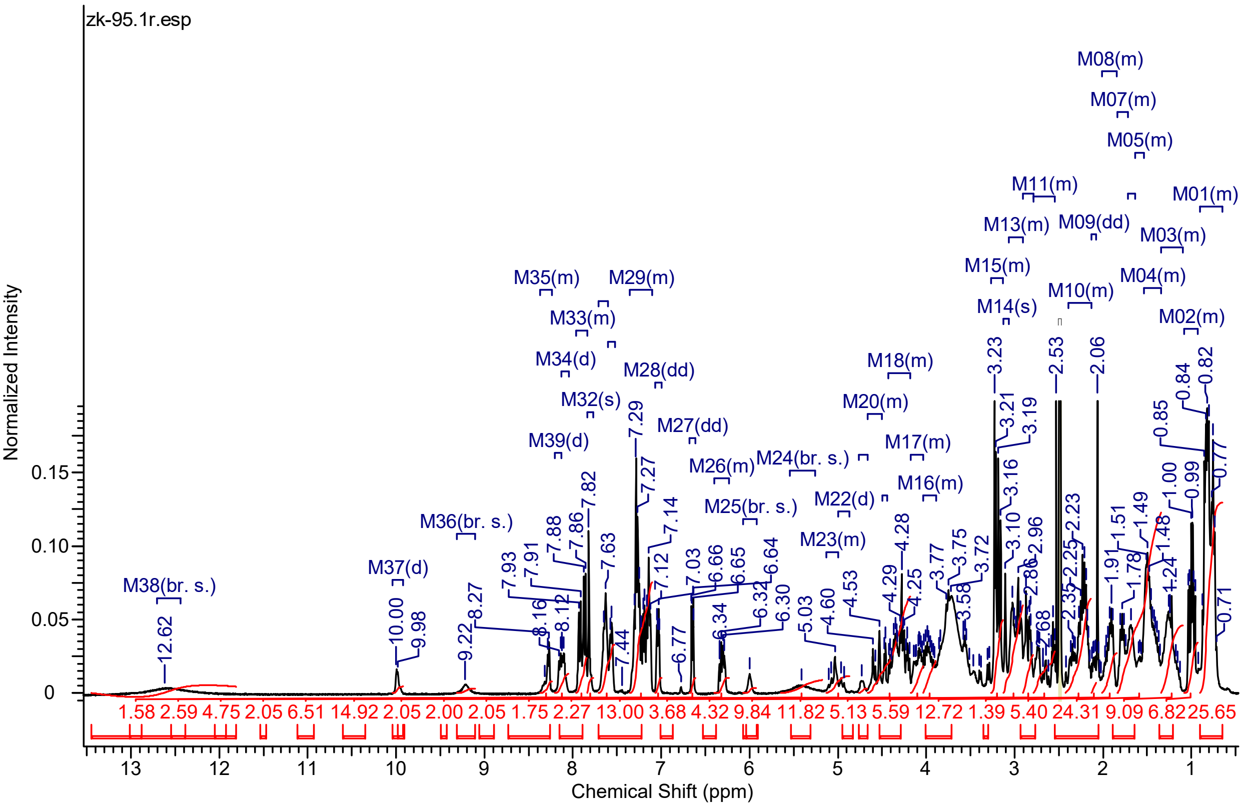

^{13}C NMR spectra for compound **12c**

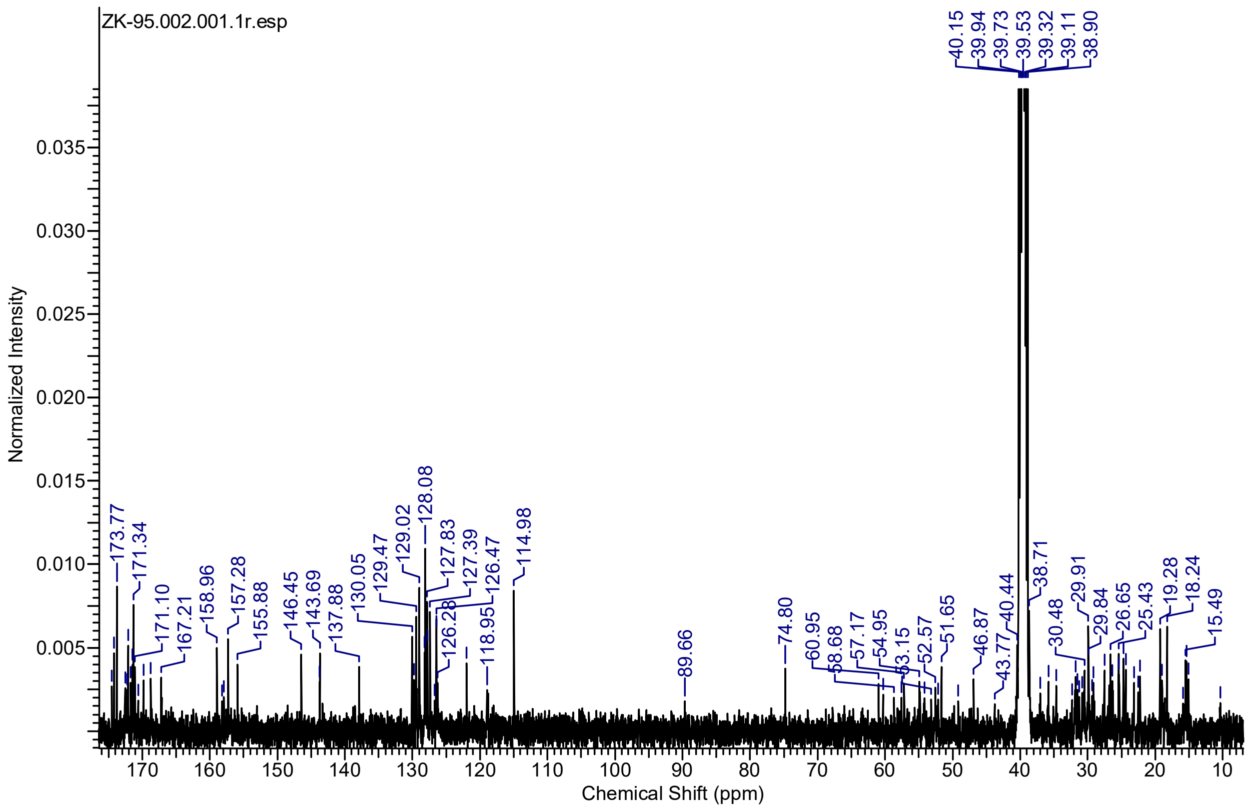

ESI-HRMS for compound **12c**

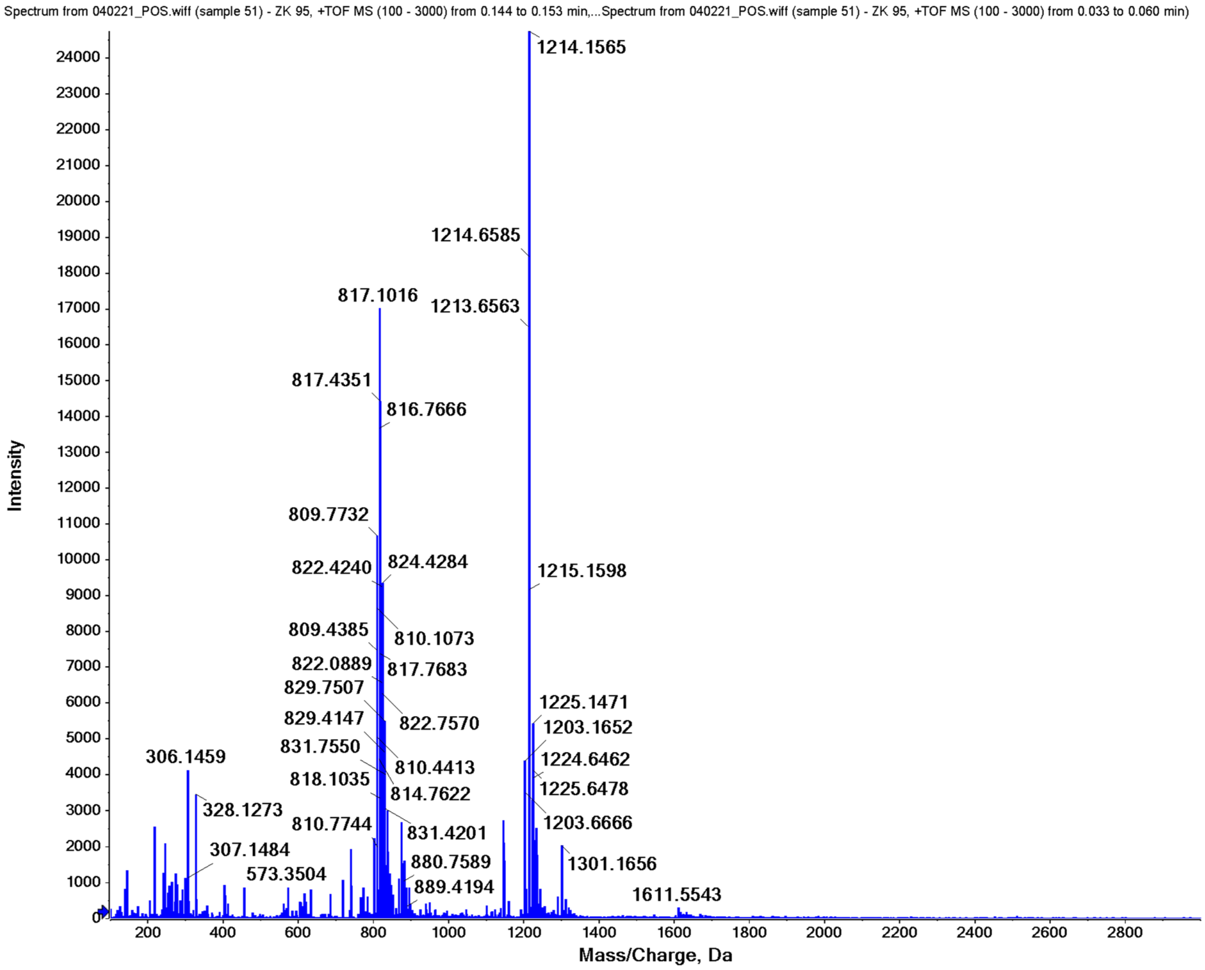

HPLC chromatogram for compound **12c**

Positive ions (chromatogram and blank)

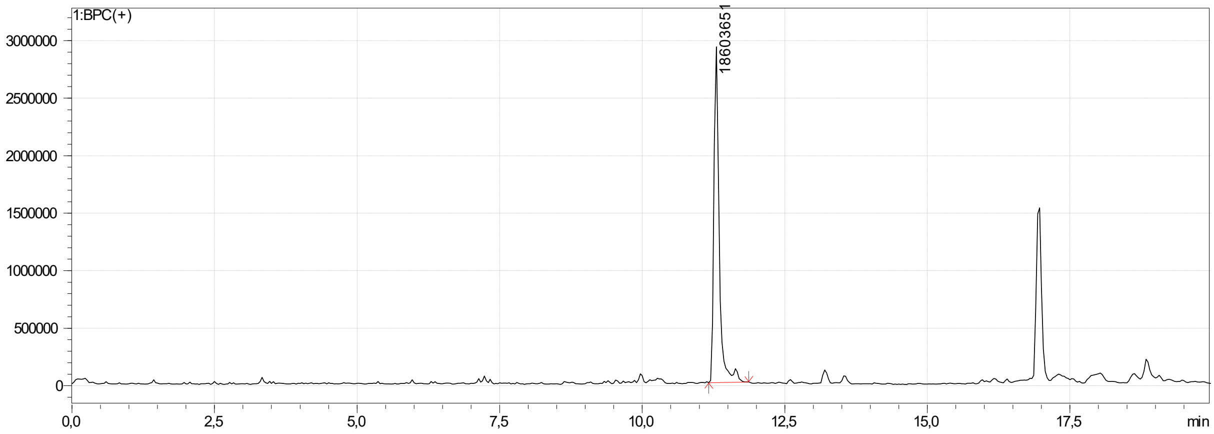

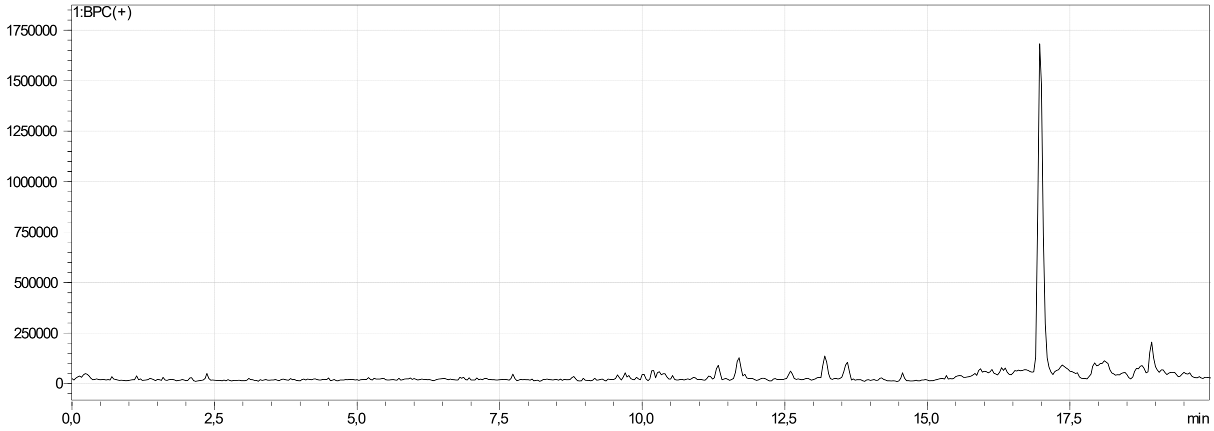

Negative ions (chromatogram and blank)

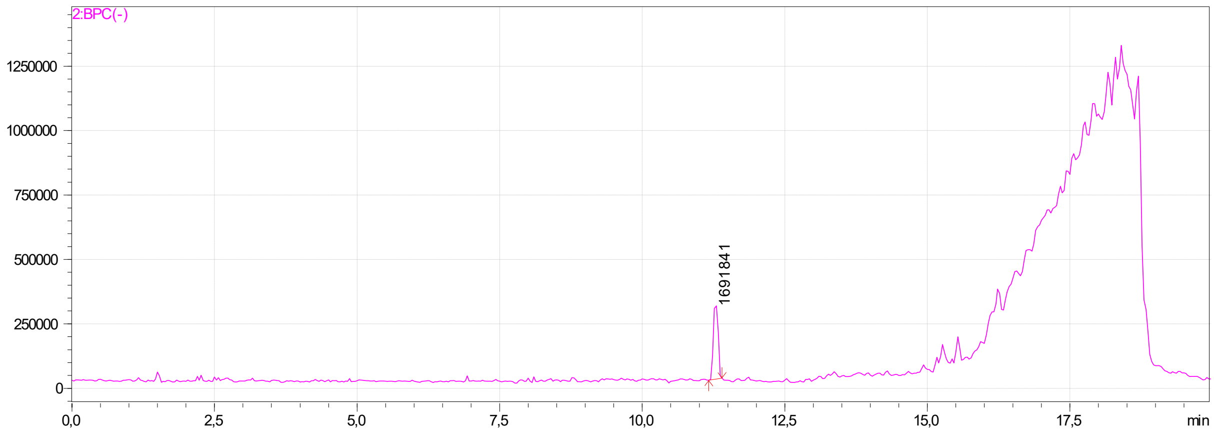

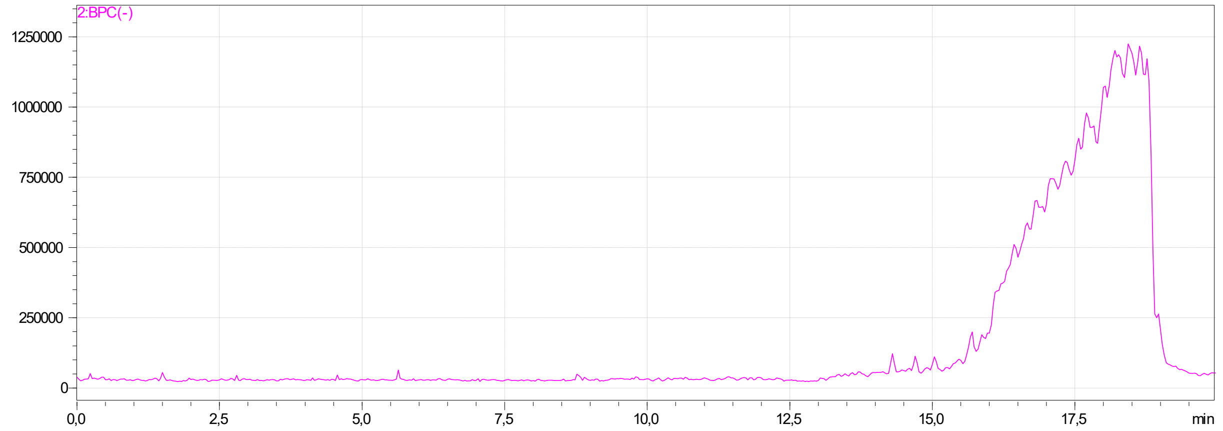

LCMS spectra for compound **12c**

Positive ions spectra for compound **12c**

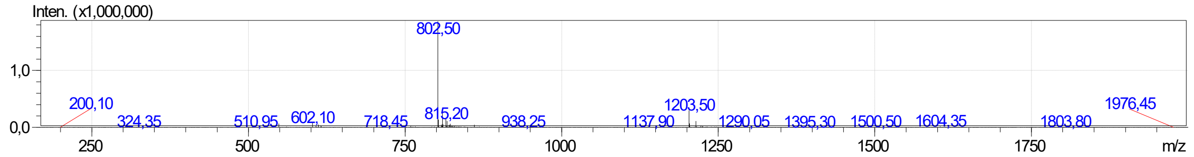

Negative ions spectra for compound **12c**

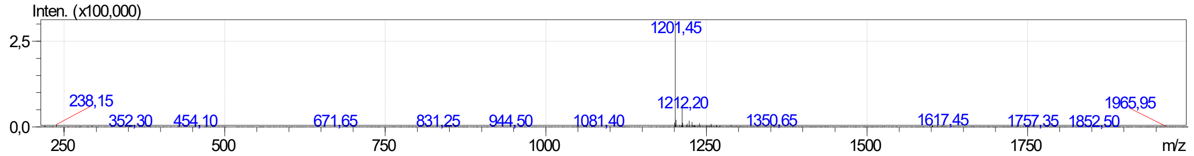

^{1}H NMR spectra for compound **12d**

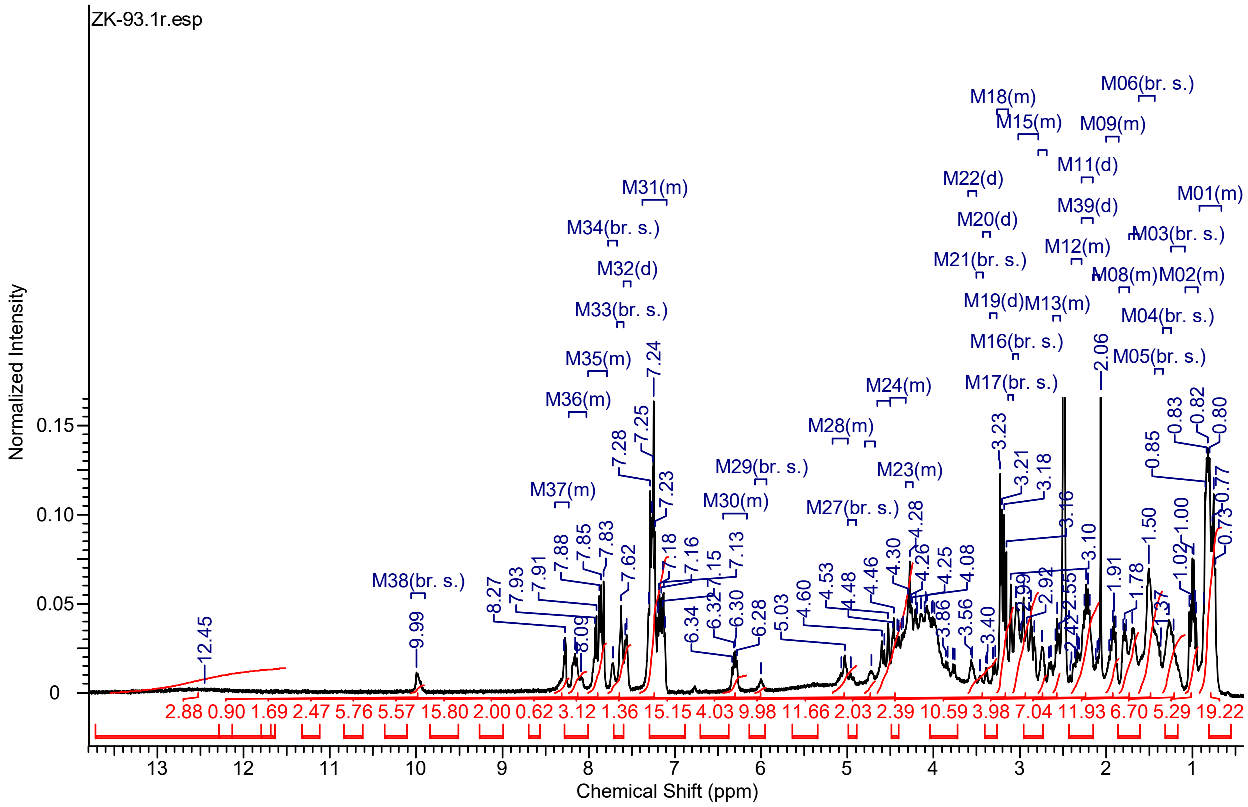

^{13}C NMR spectra for compound **12d**

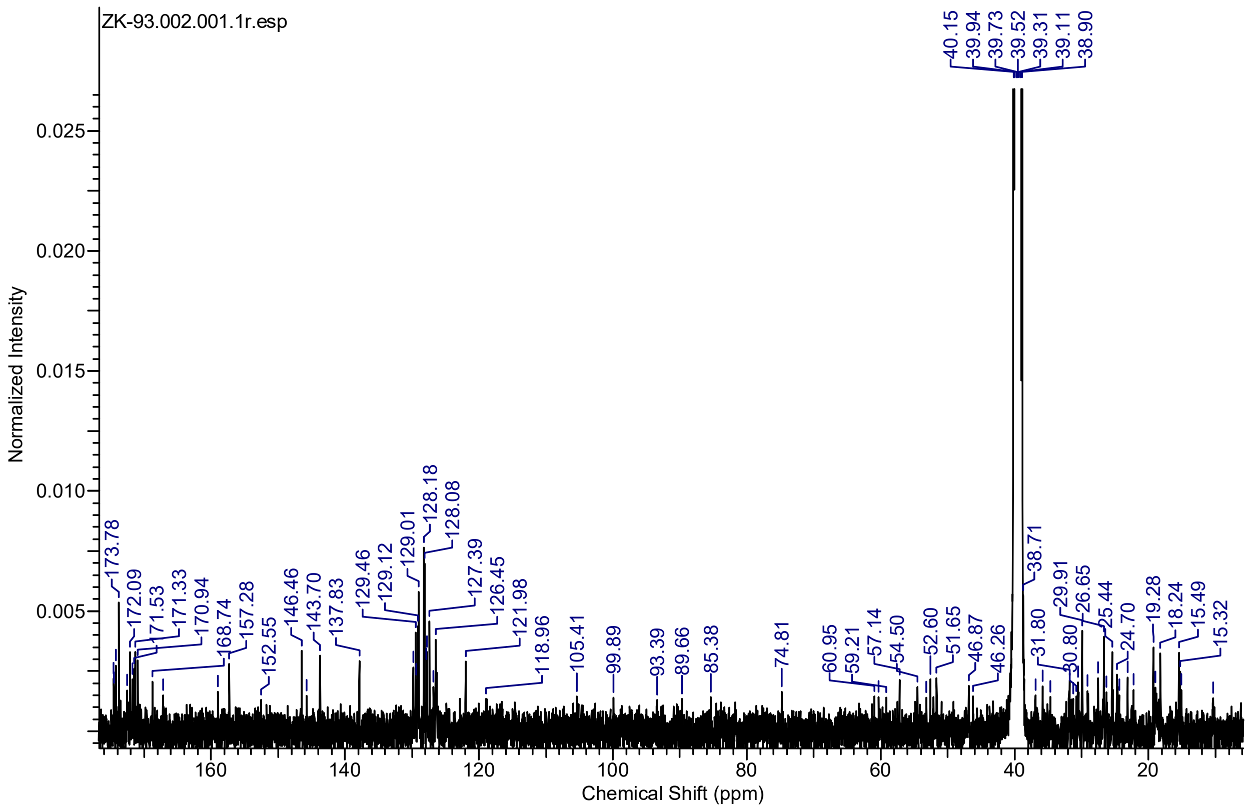

ESI-HRMS for compound **12d**

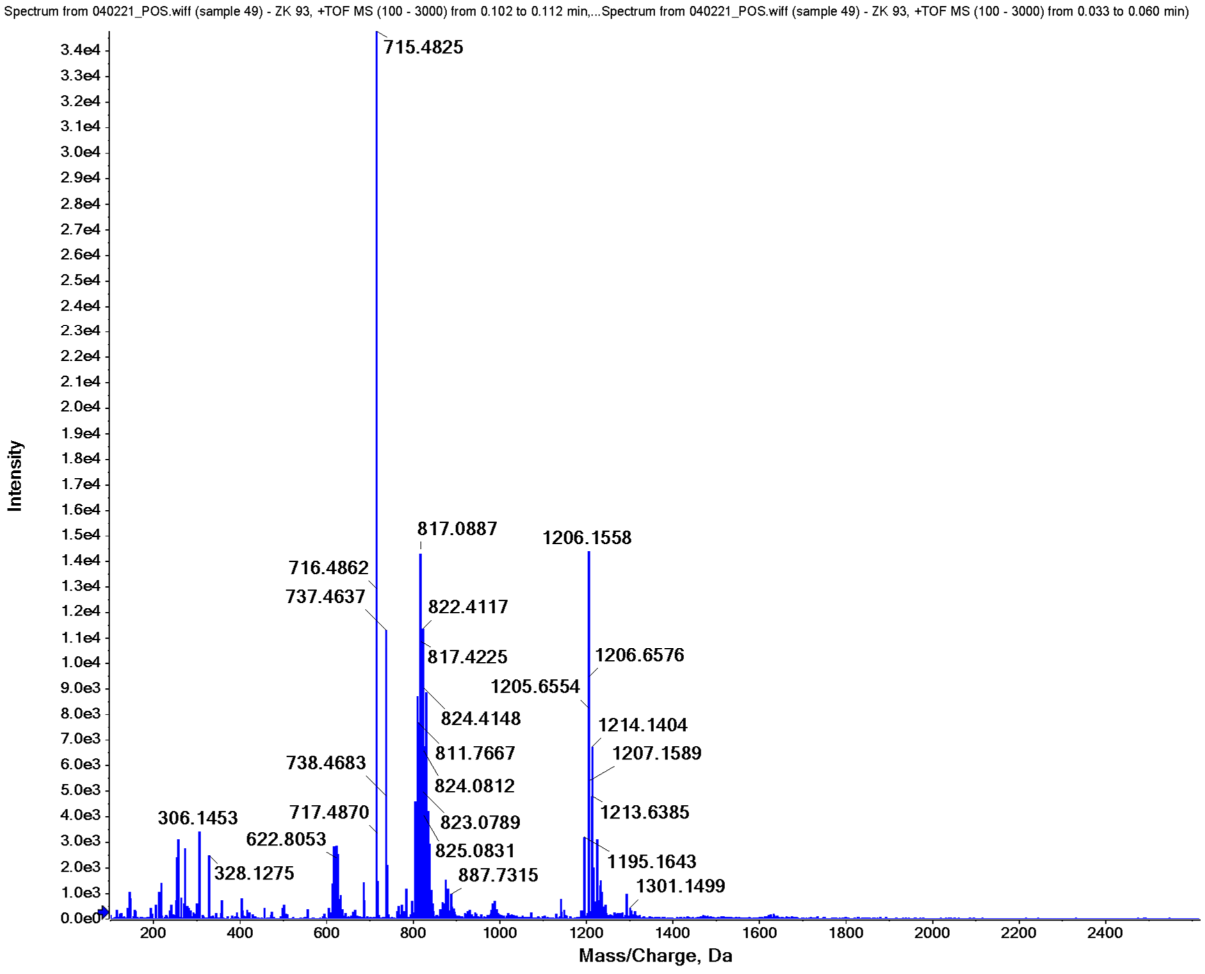

HPLC chromatogram for compound **12d**

Positive ions (chromatogram and blank)

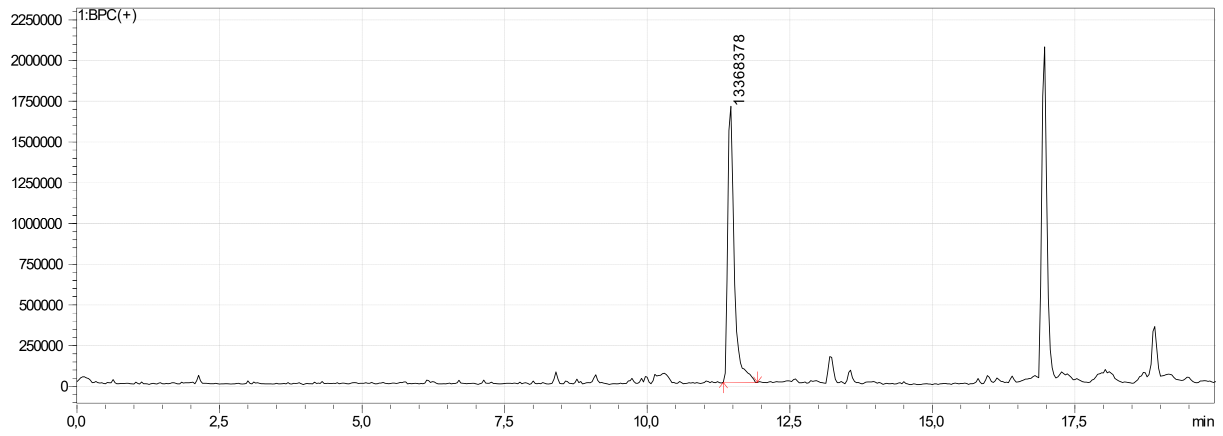

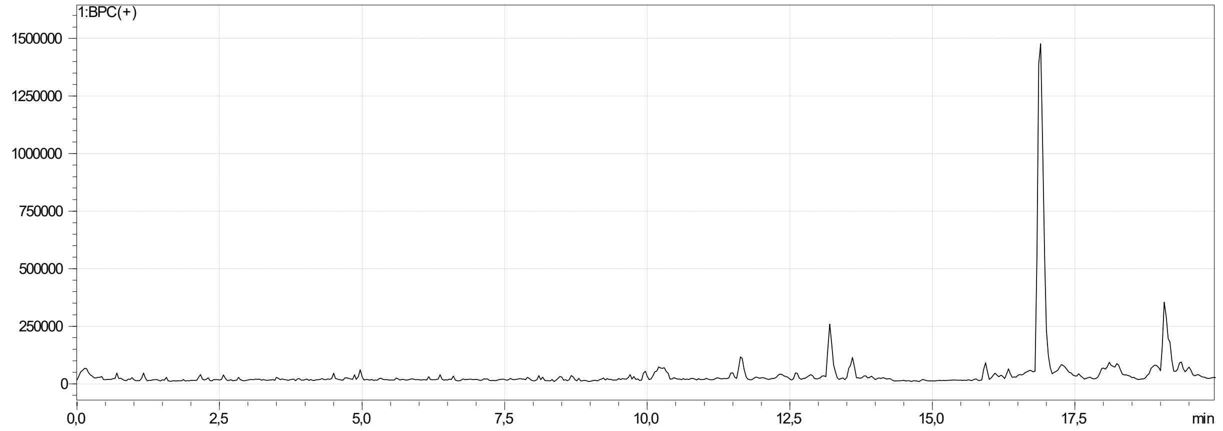

Negative ions (chromatogram and blank)

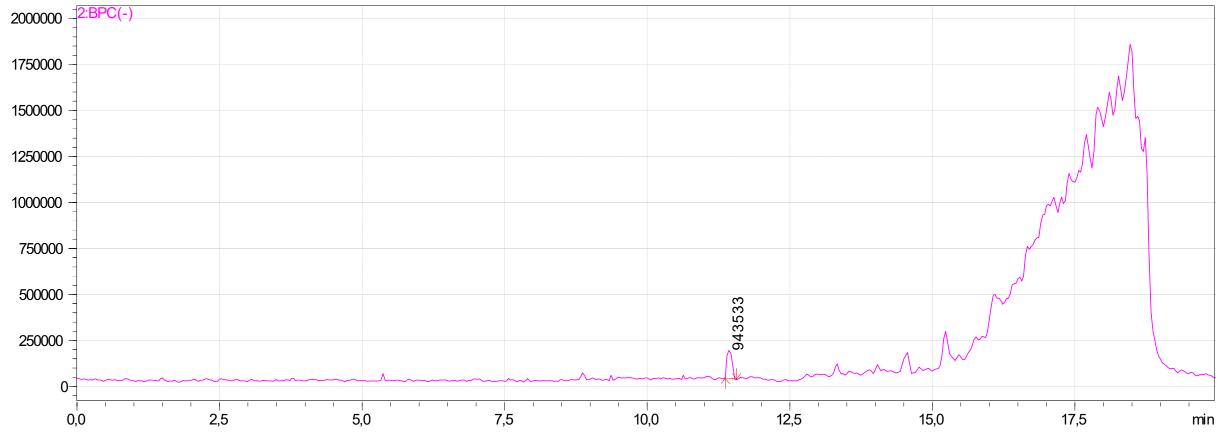

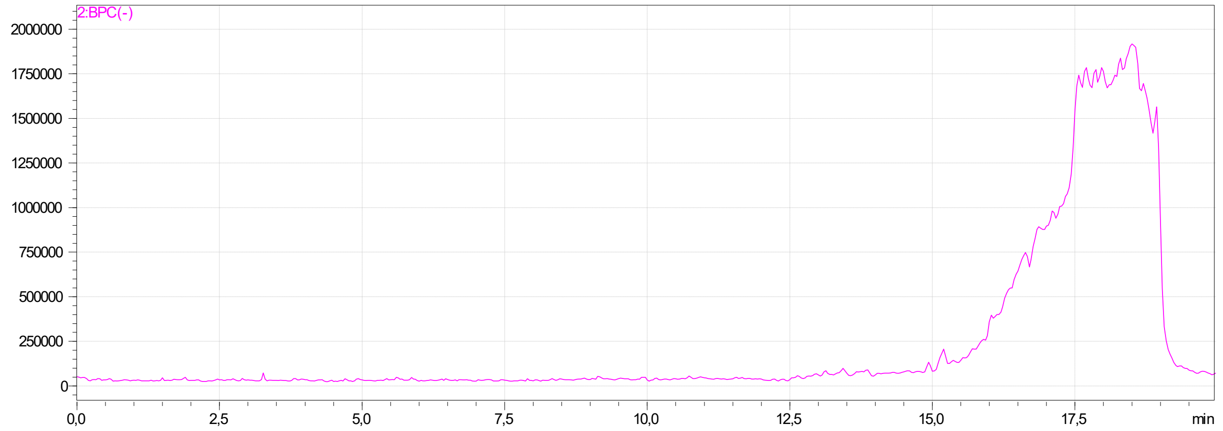

LCMS spectra for compound **12d**

Positive ions spectra for compound **12d**

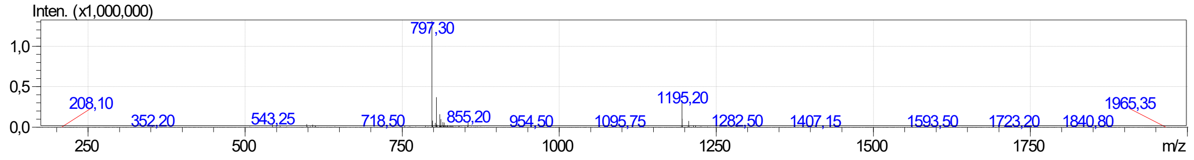

Negative ions spectra for compound **12d**

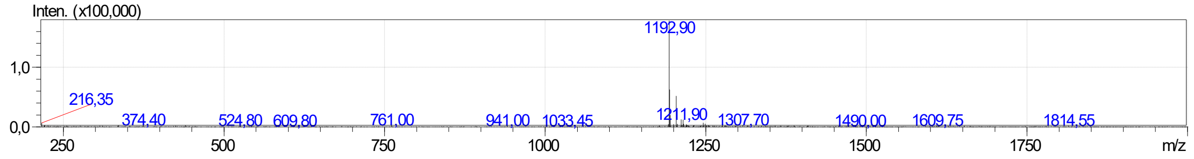

^{1}H NMR spectra for compound **13a**

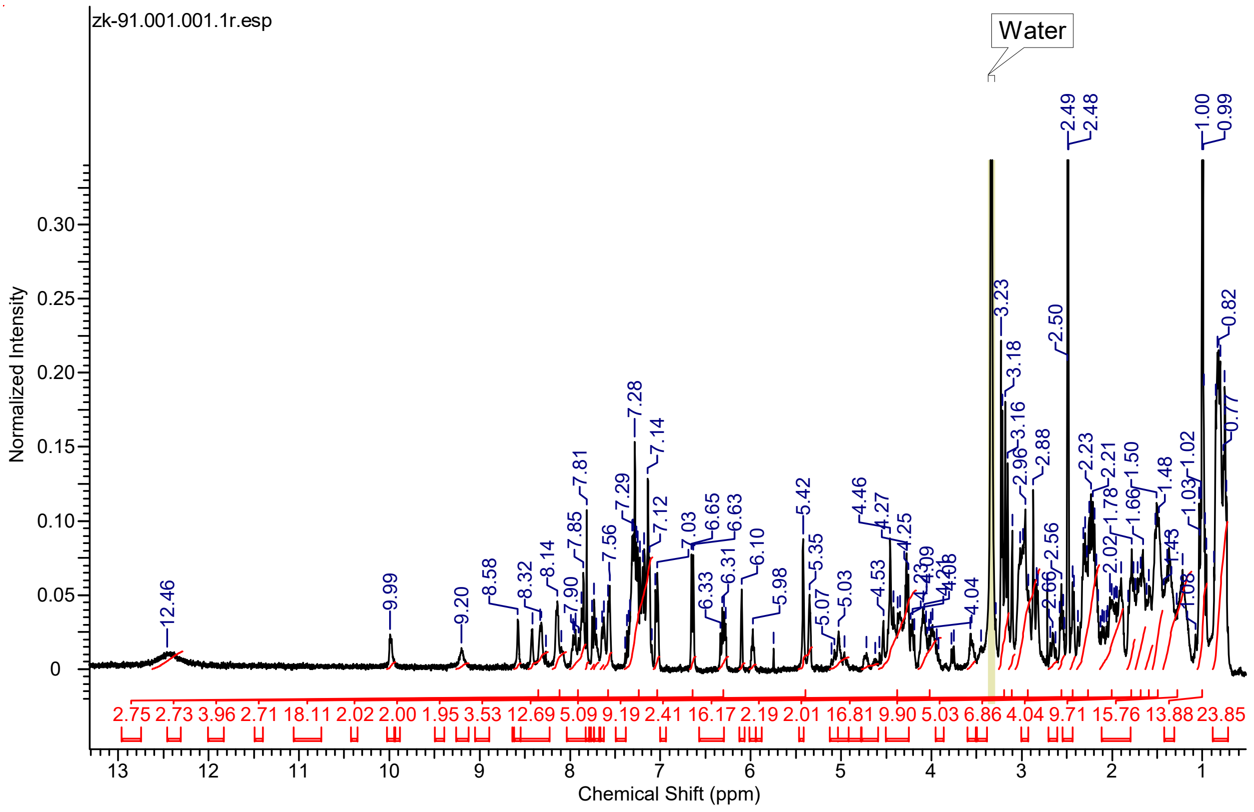

^{13}C NMR spectra for compound **13a**

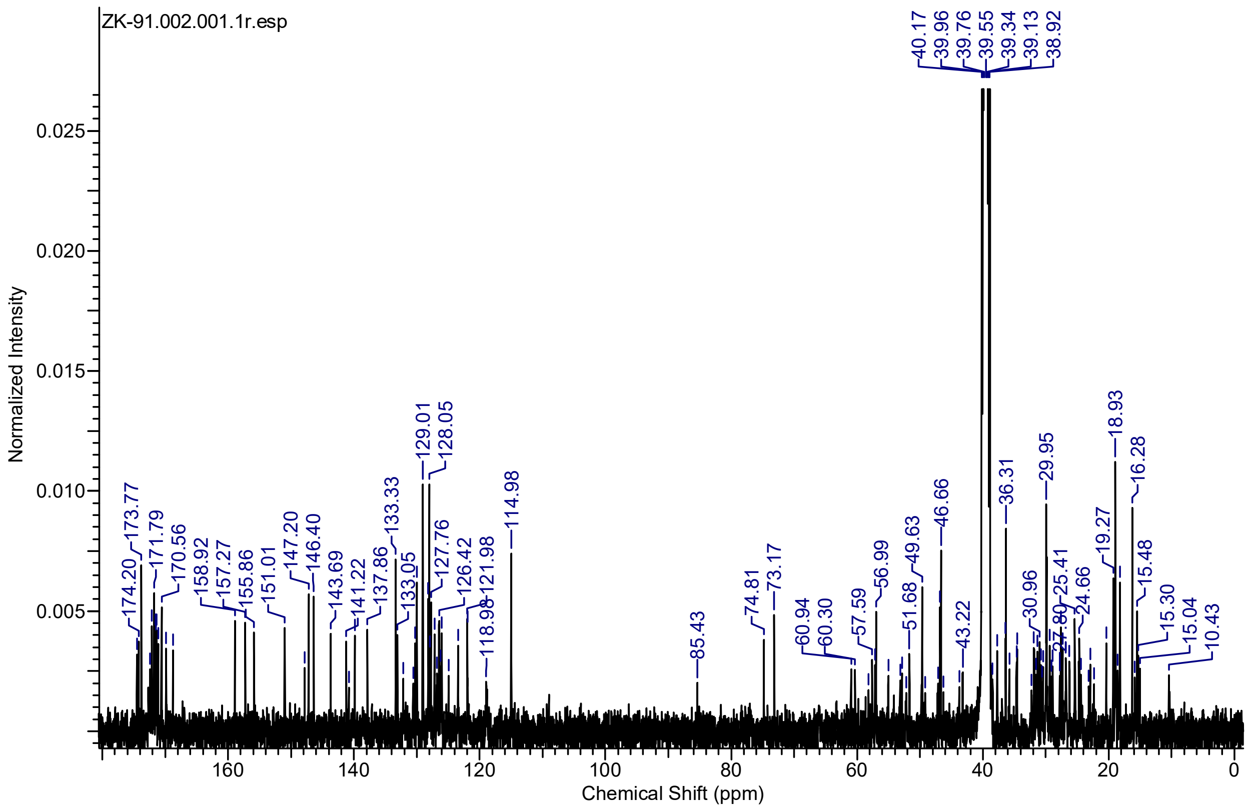

ESI-HRMS for compound **13a**

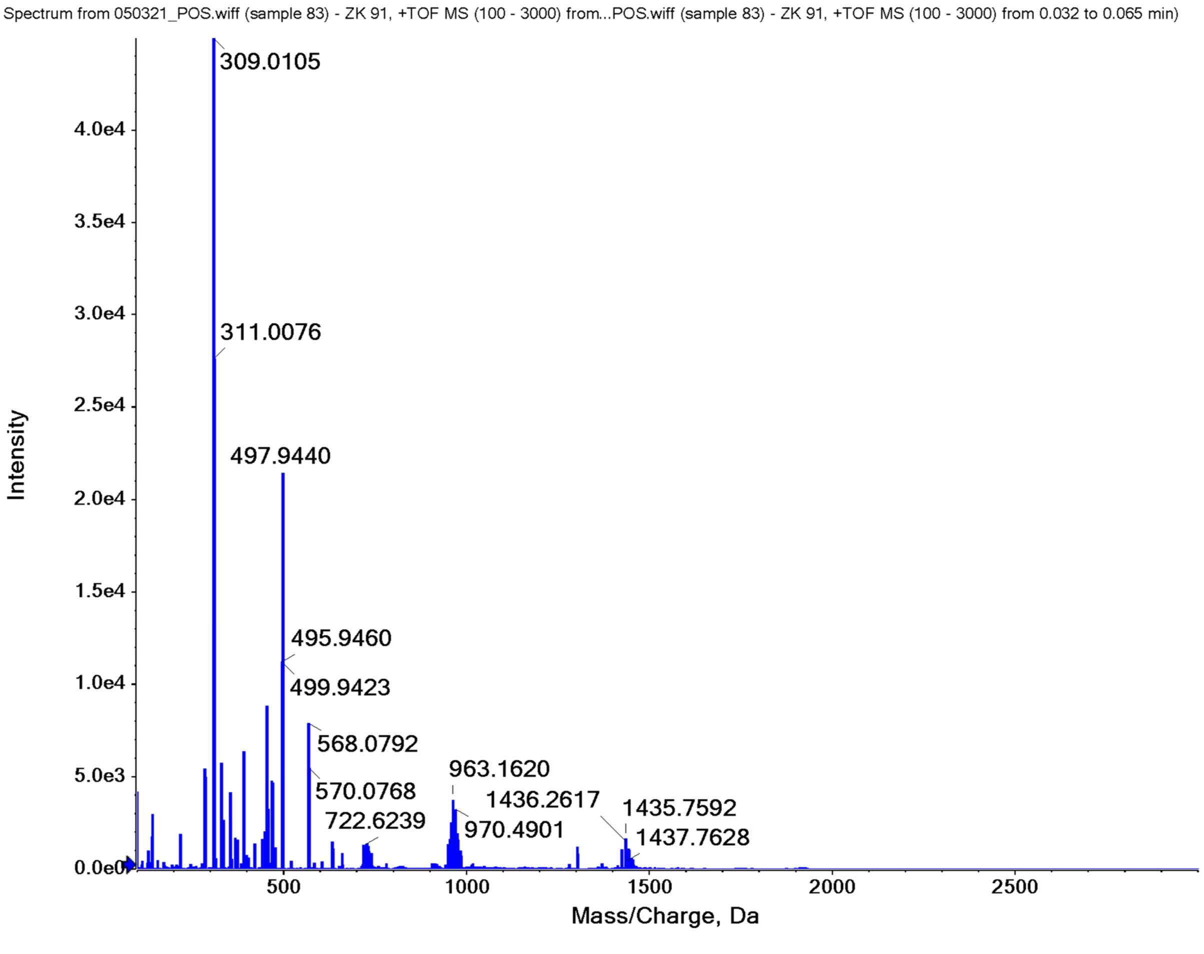

HPLC chromatogram for compound **13a**

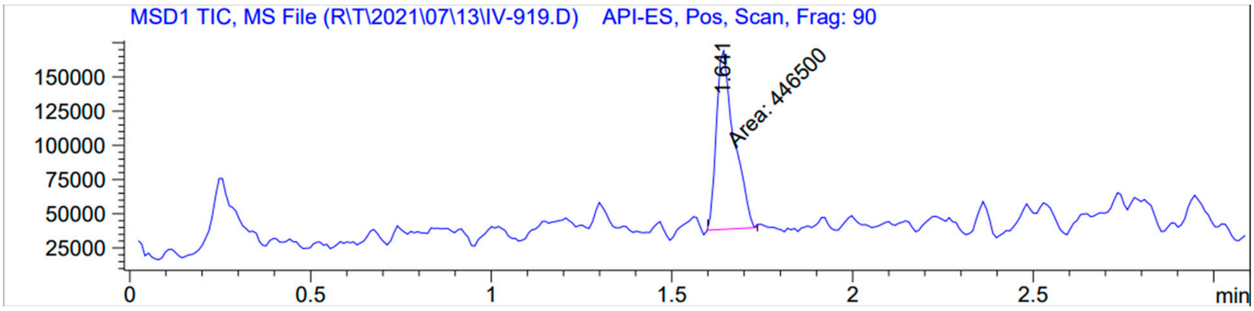

LCMS spectra for compound **13a**

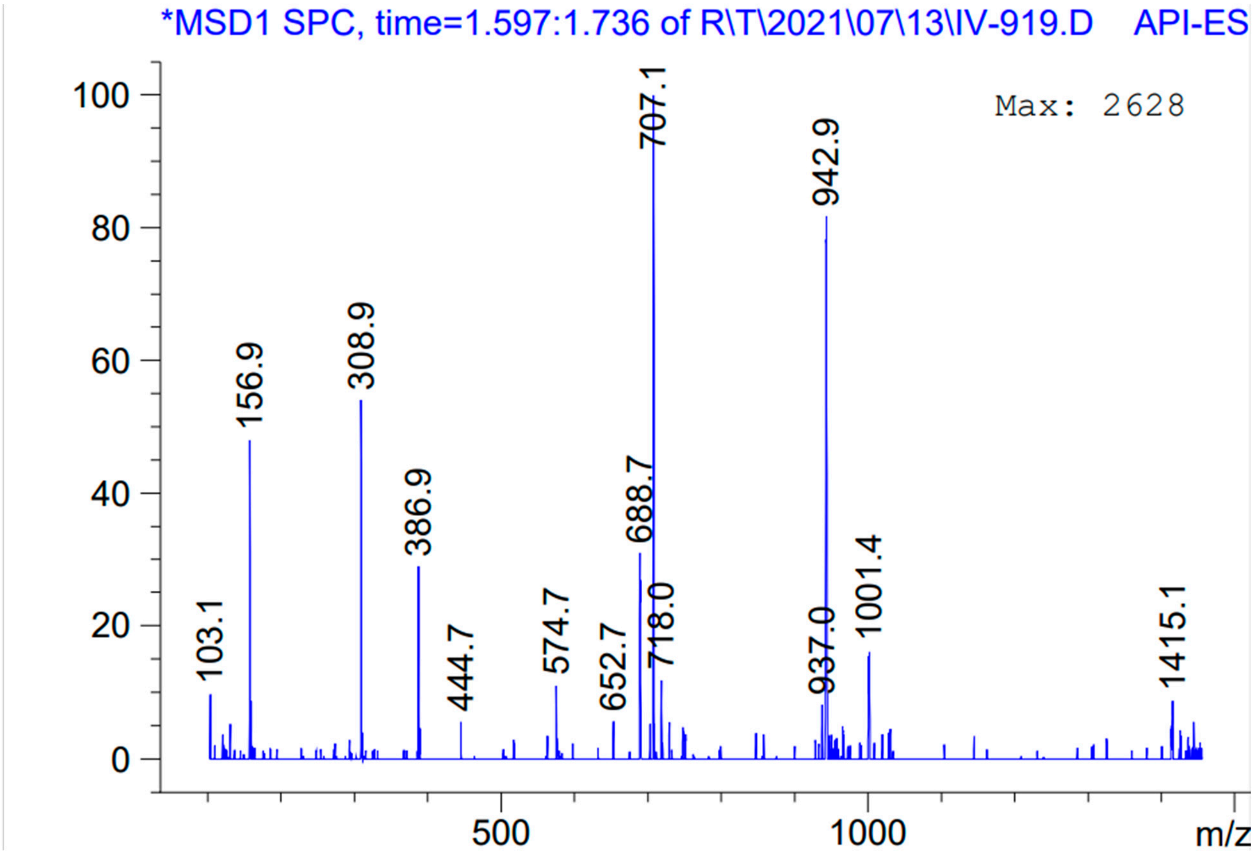

^{1}H NMR spectra for compound **13b**

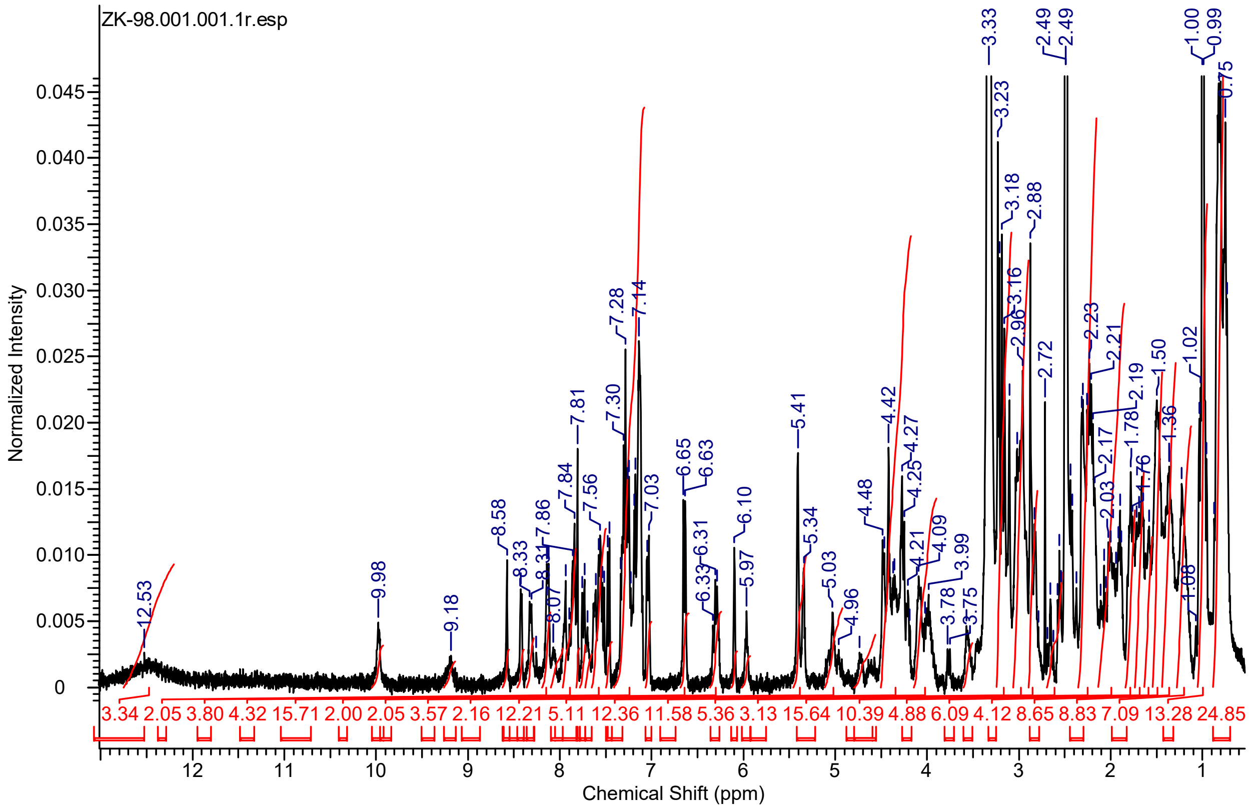

^{13}C NMR spectra for compound **13b**

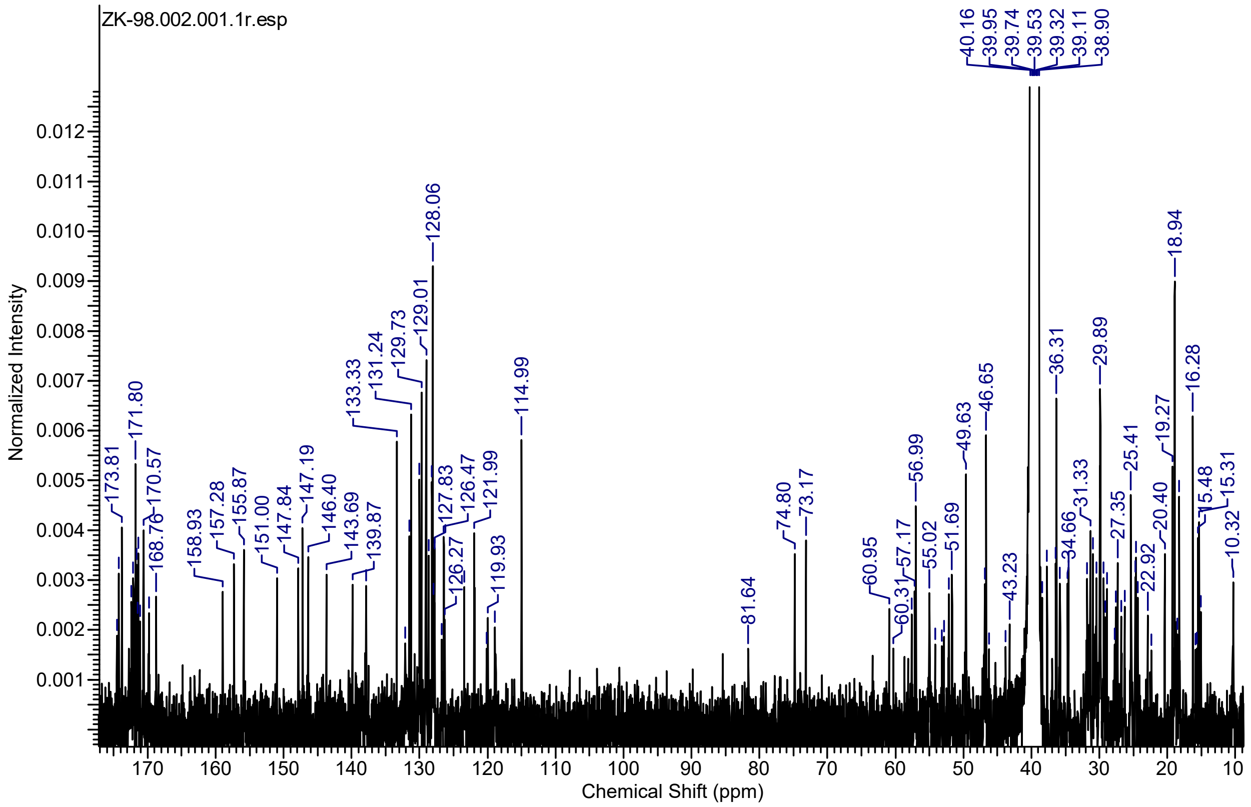

ESI-HRMS for compound **13b**

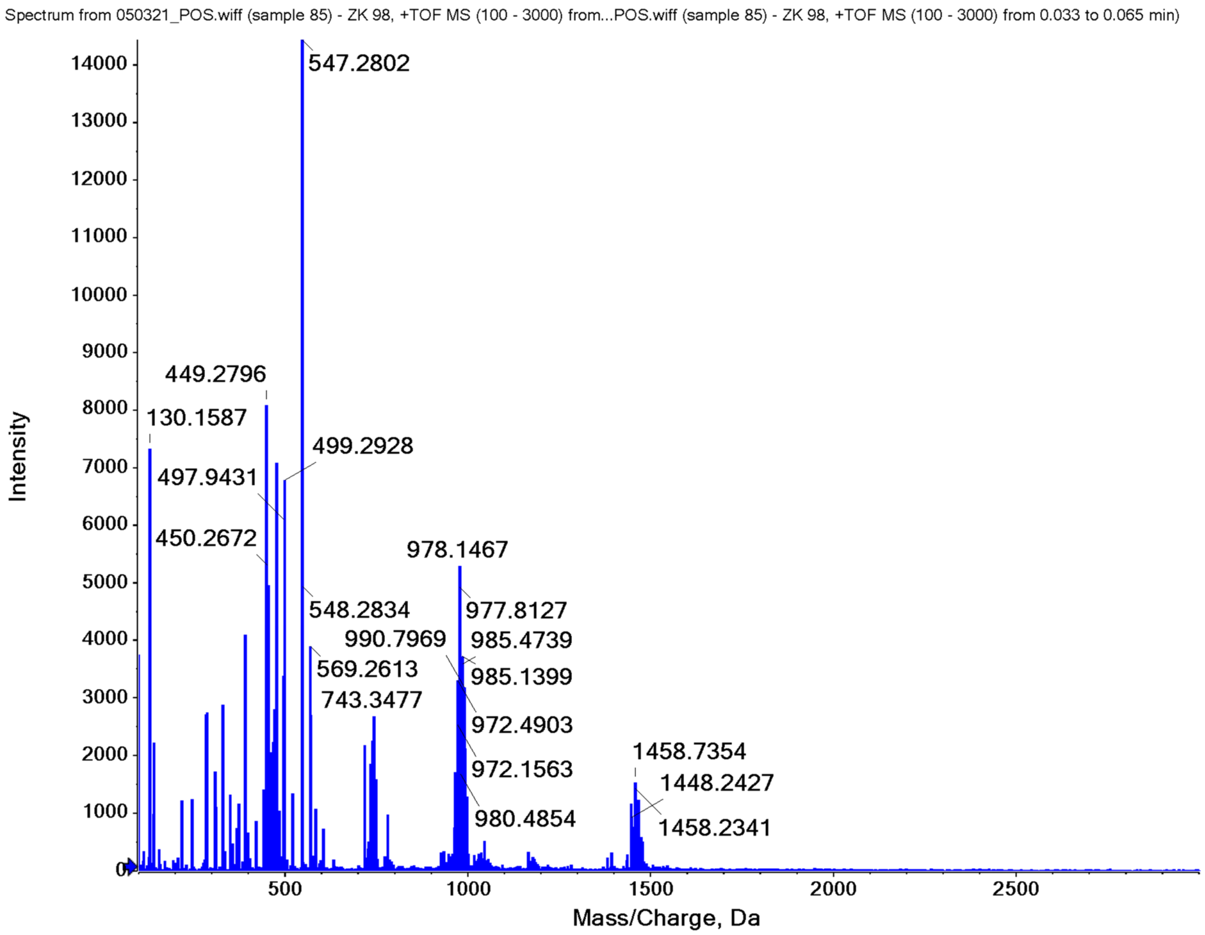

HPLC chromatogram for compound **13b** (chromatogram and blank)

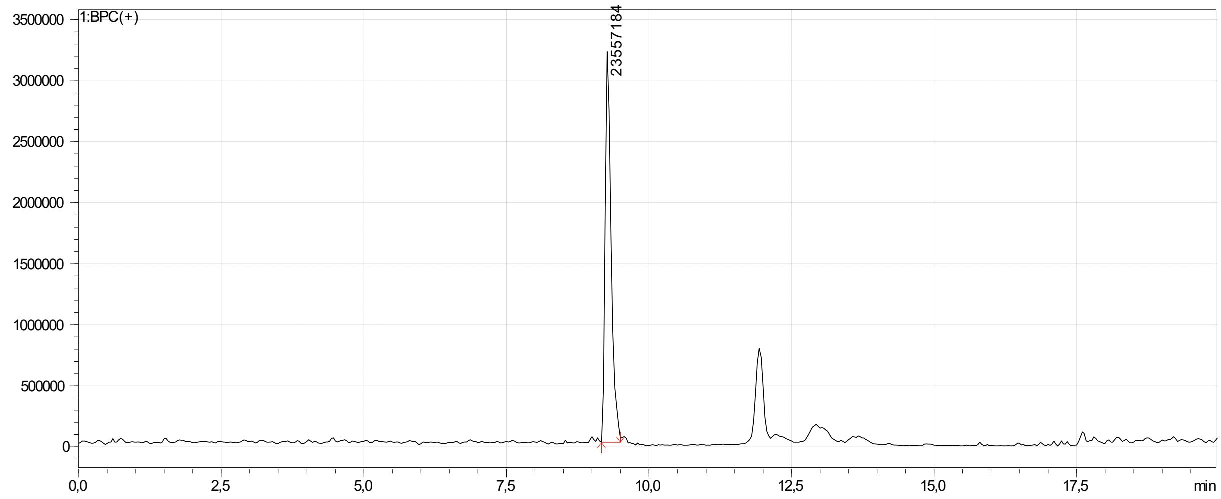

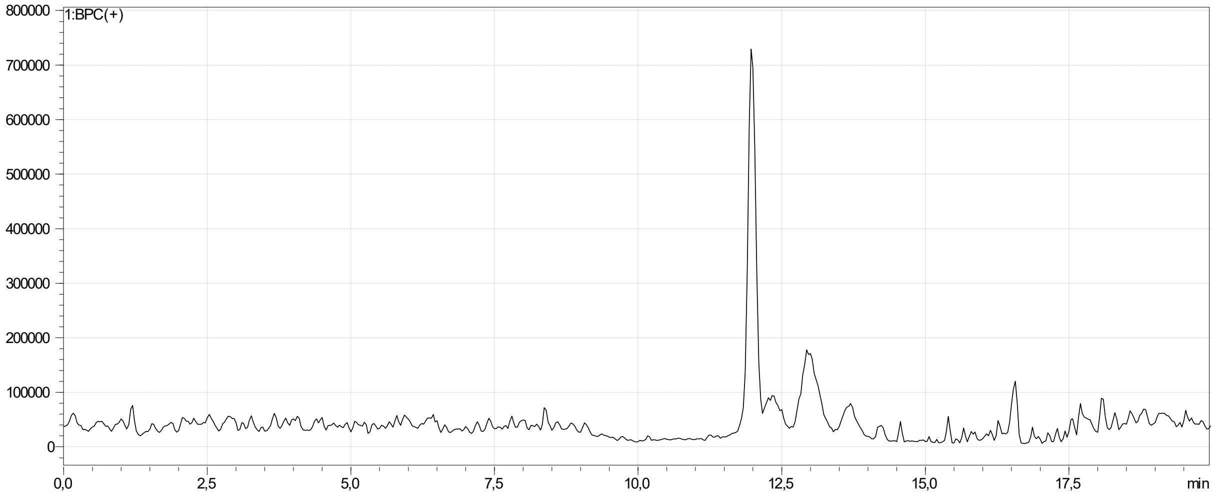

LCMS spectra for compound **13b**

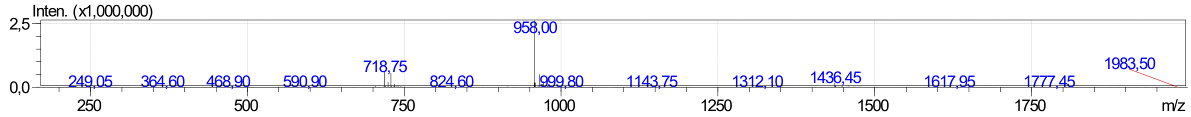

^{1}H NMR spectra for compound **13c**

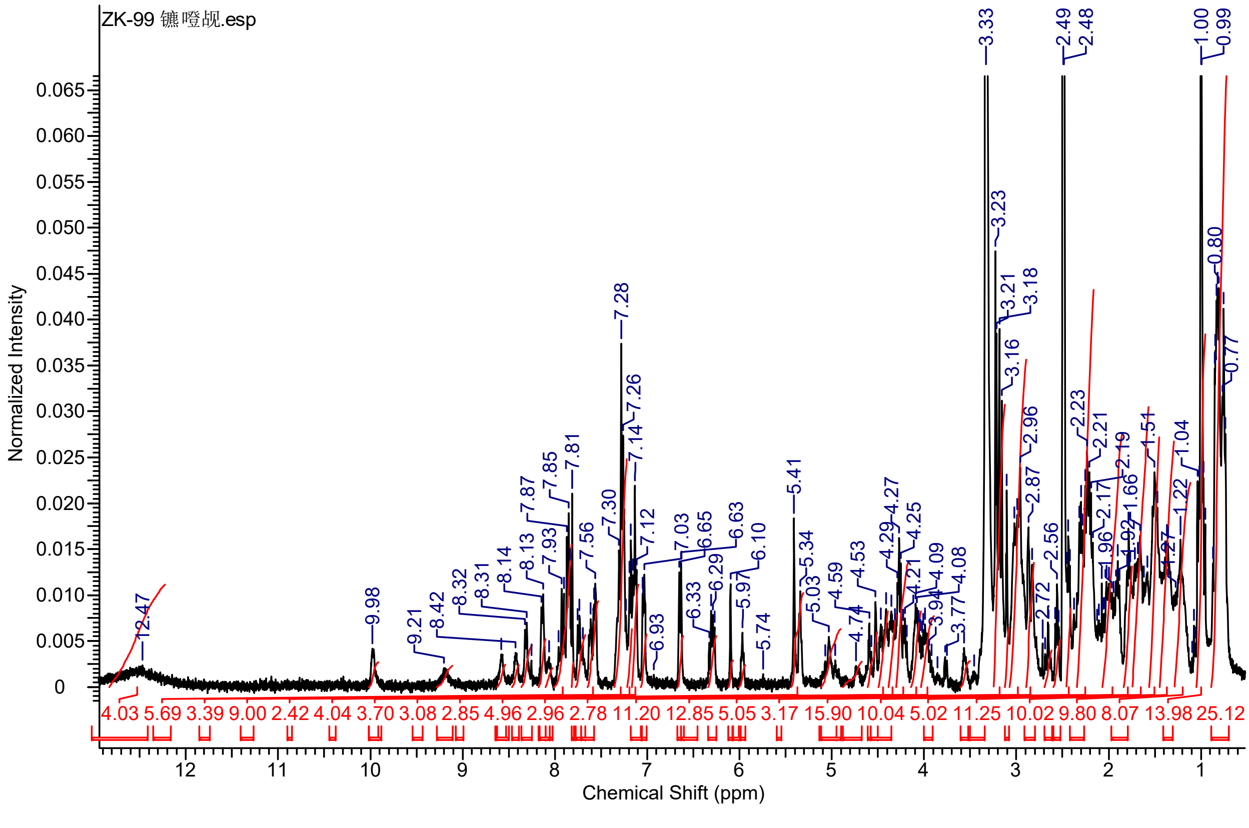

^{13}C NMR spectra for compound **13c**

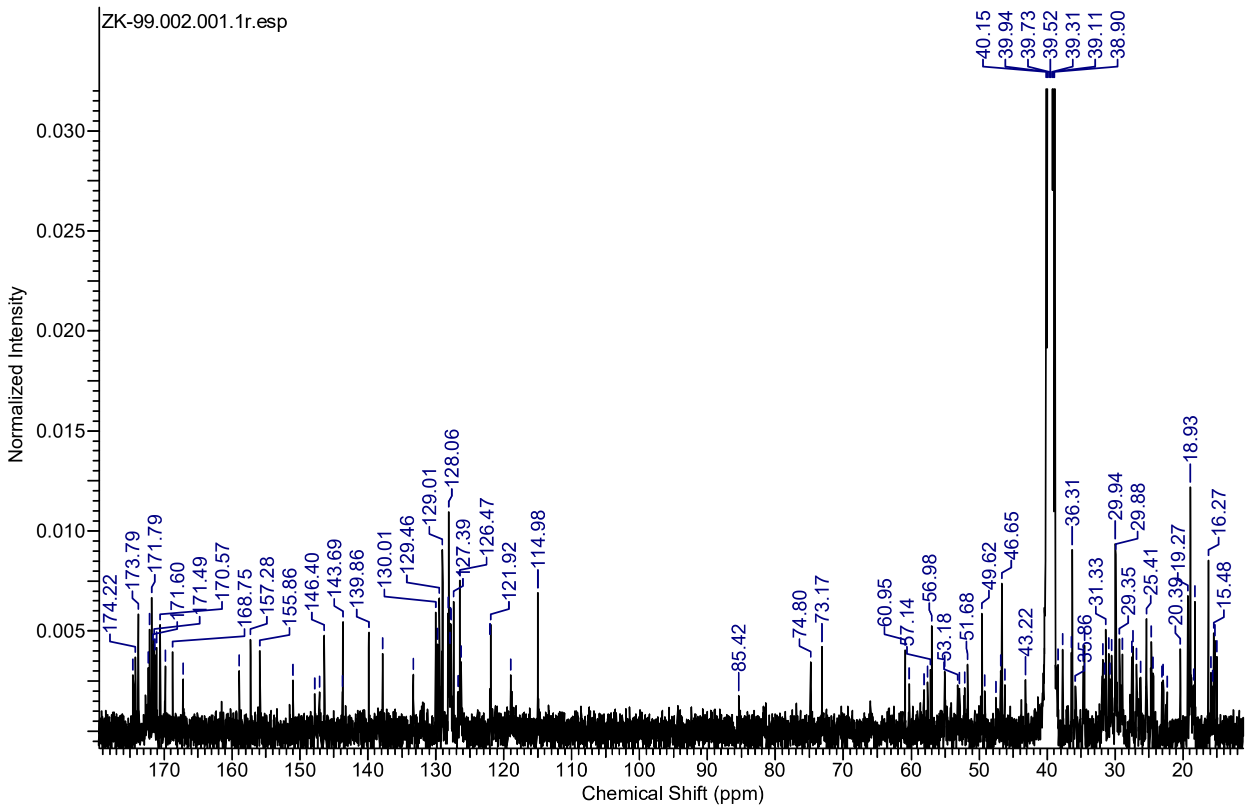

ESI-HRMS for compound **13c**

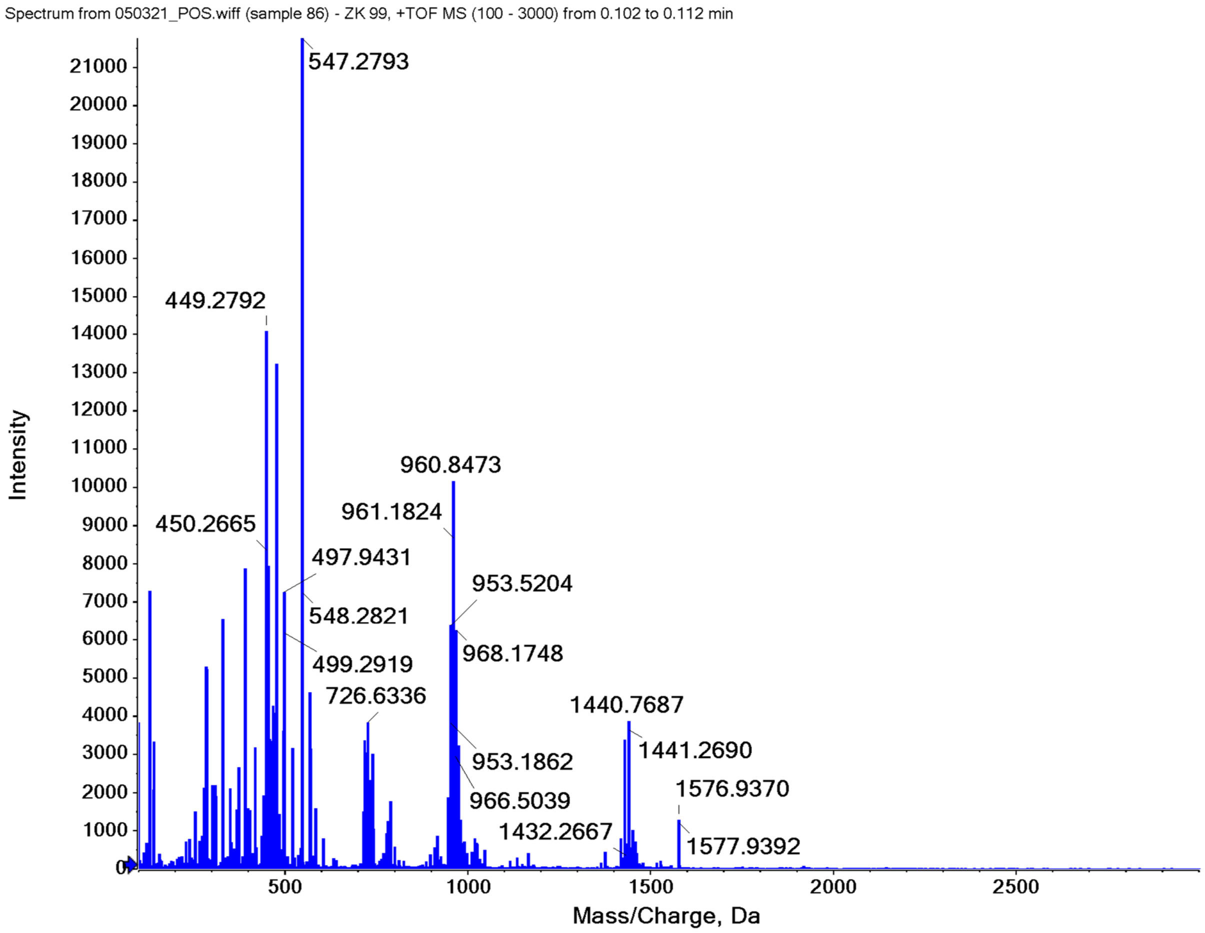

HPLC chromatogram for compound **13c** (chromatogram and blank)

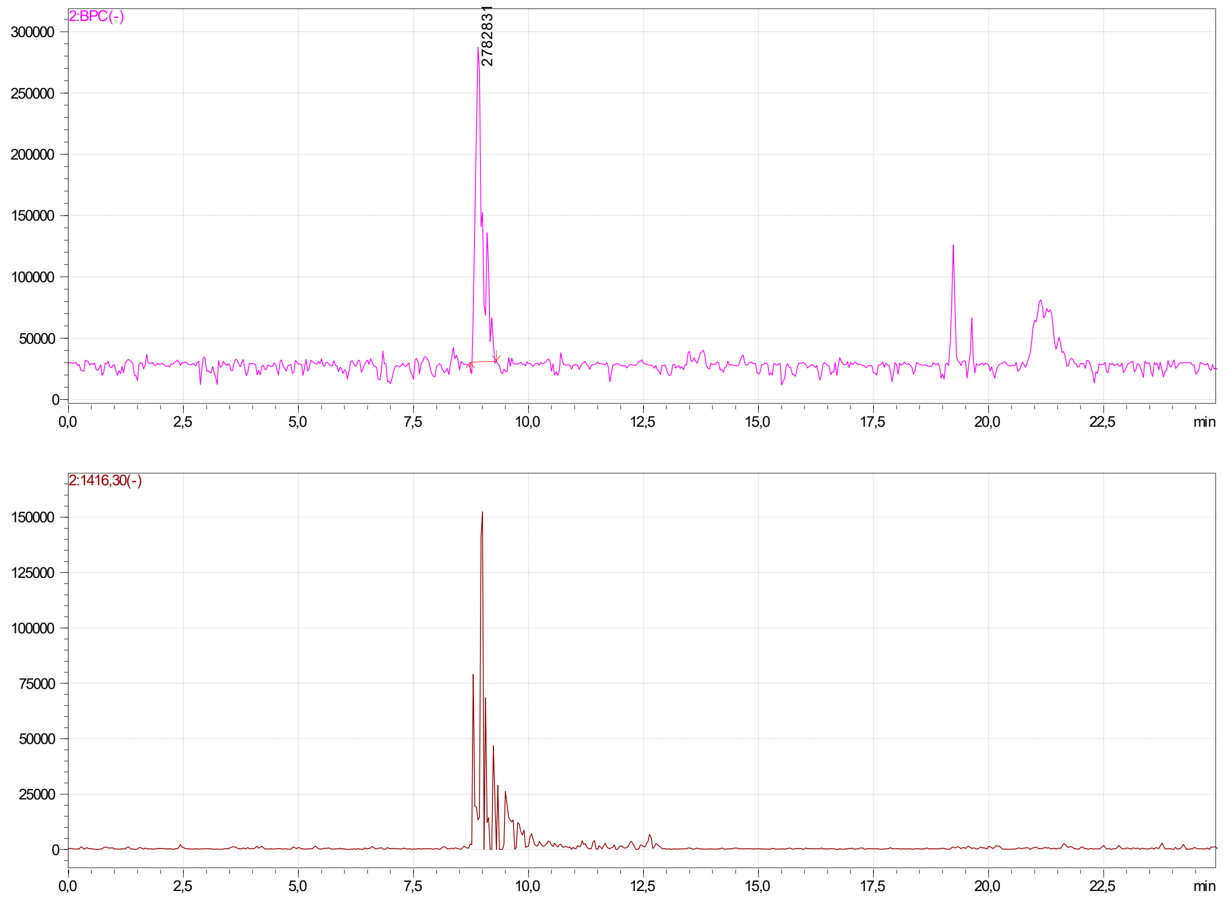

LCMS spectra for compound **13c**

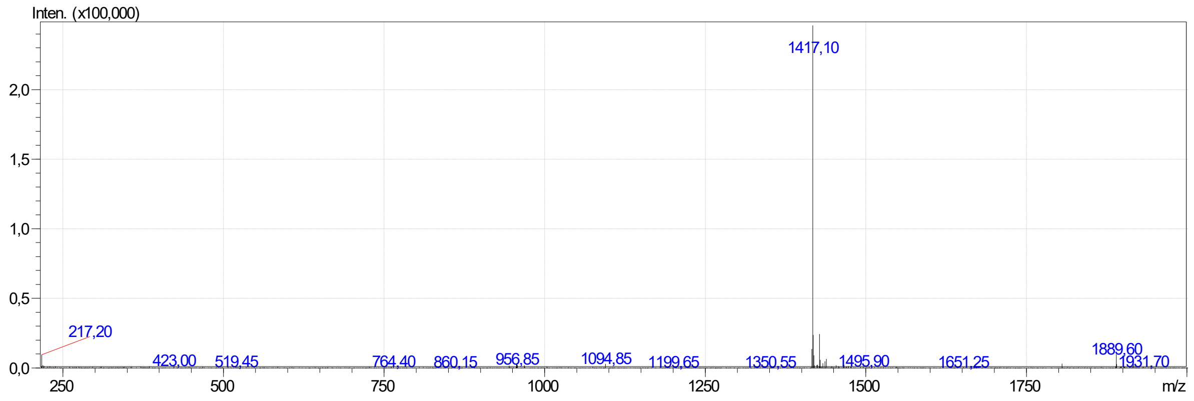

^{1}H NMR spectra for compound **13d**

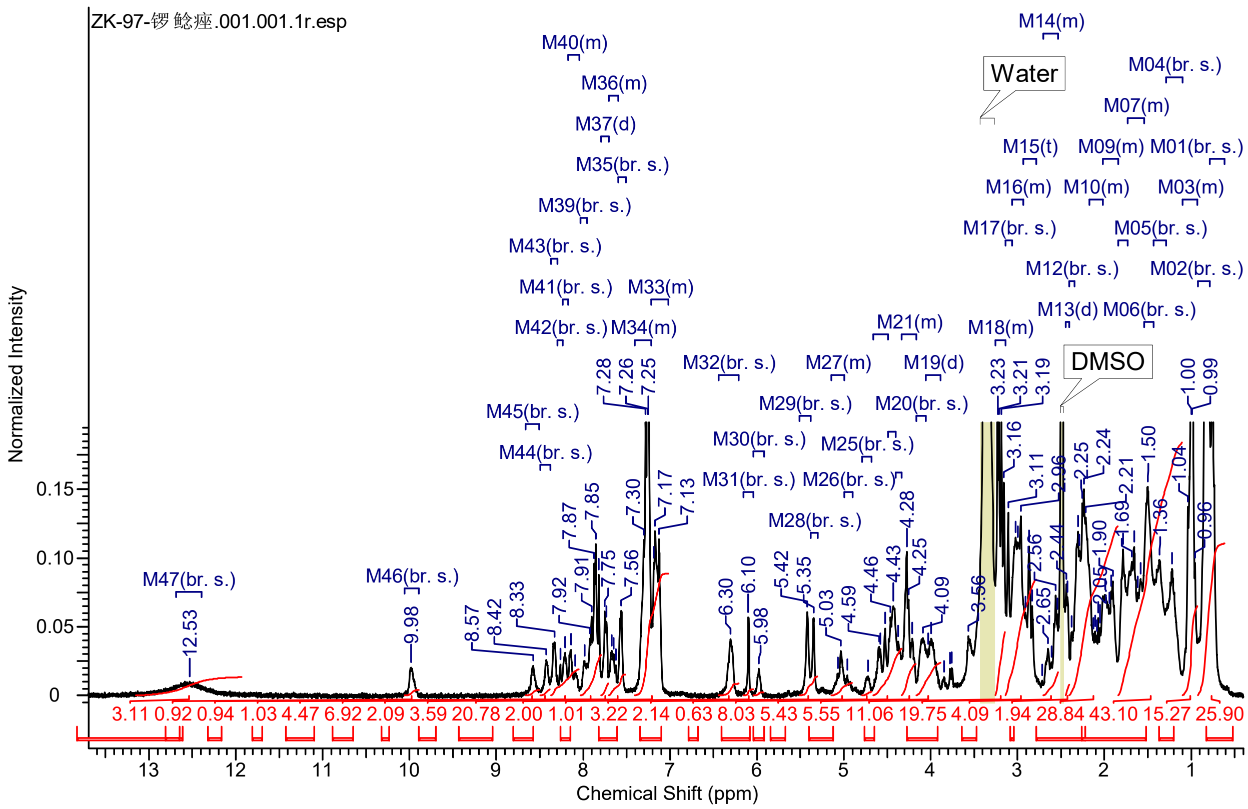

^{13}C NMR spectra for compound **13d**

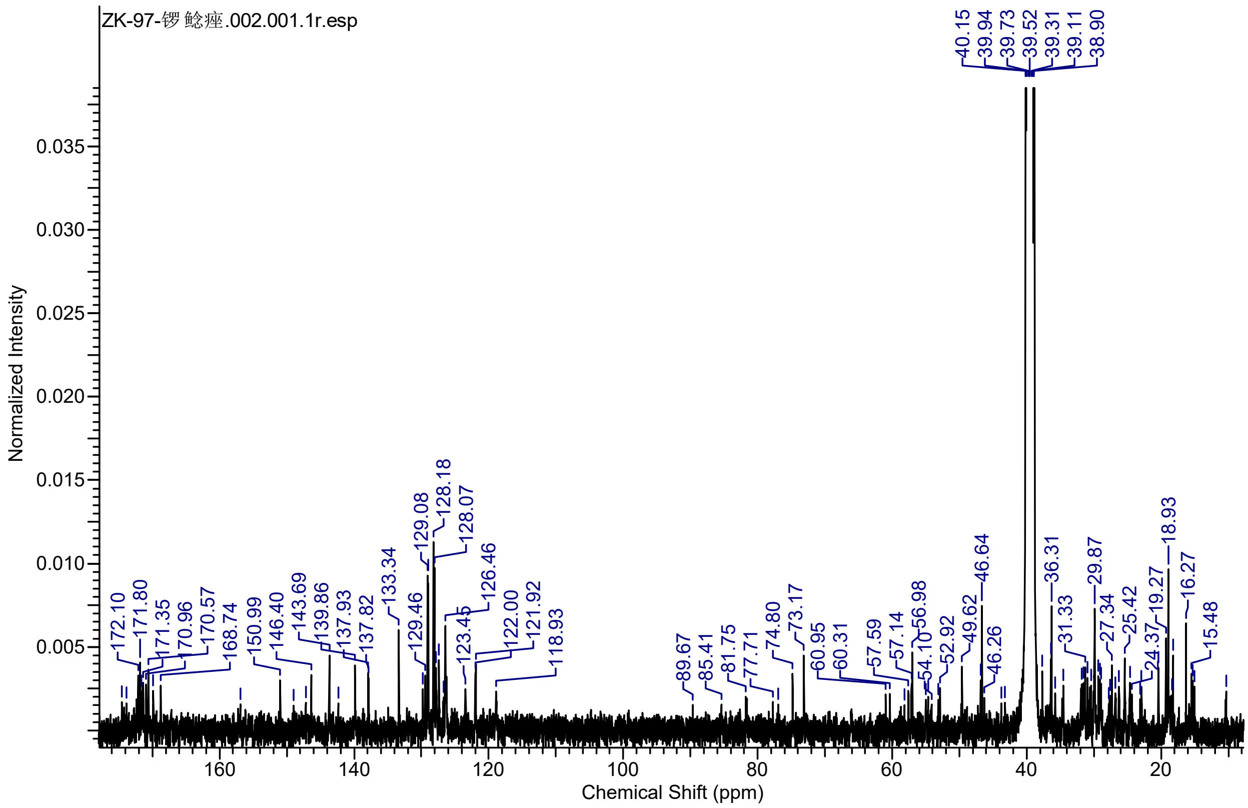

ESI-HRMS for compound **13d**

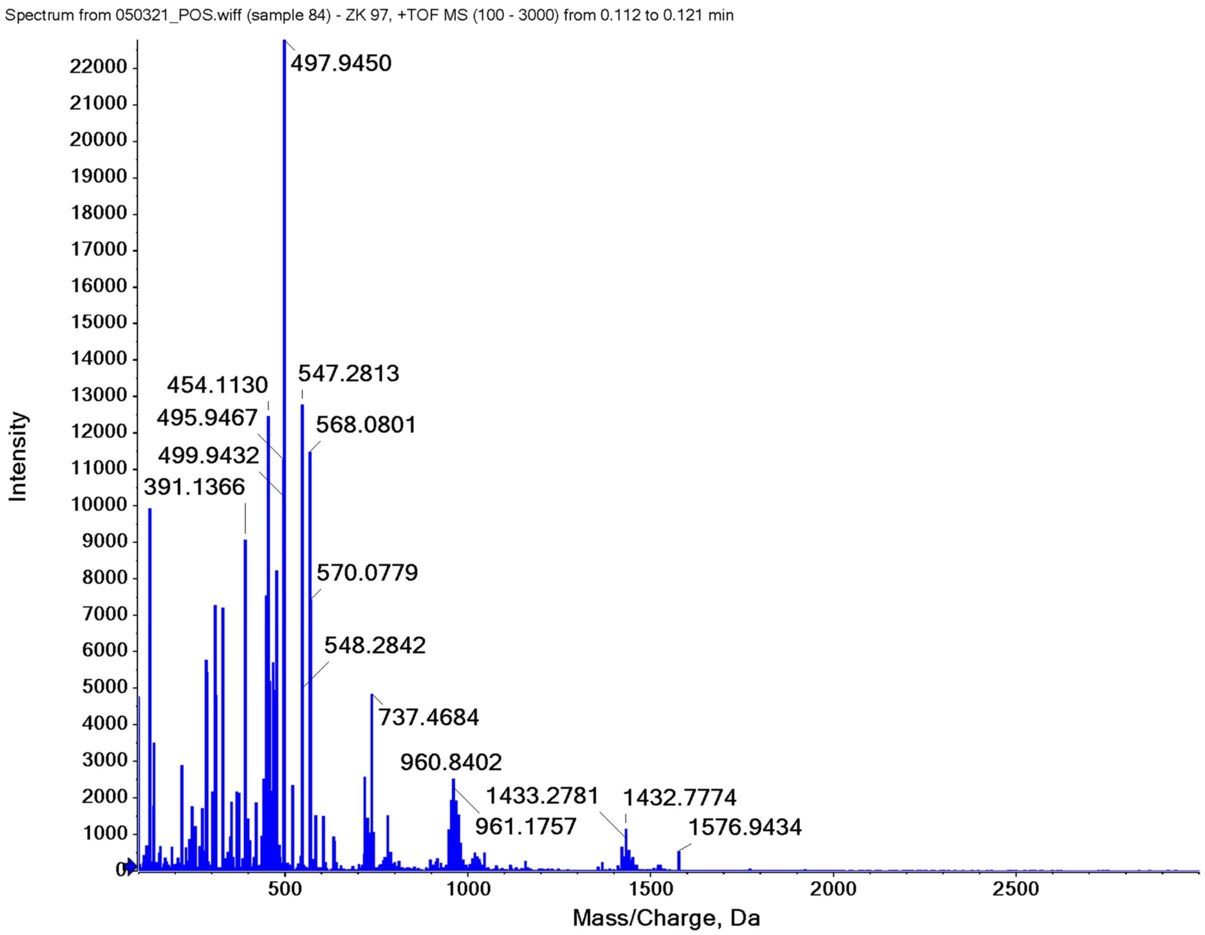

HPLC chromatogram for compound **13d** (chromatogram and blank)

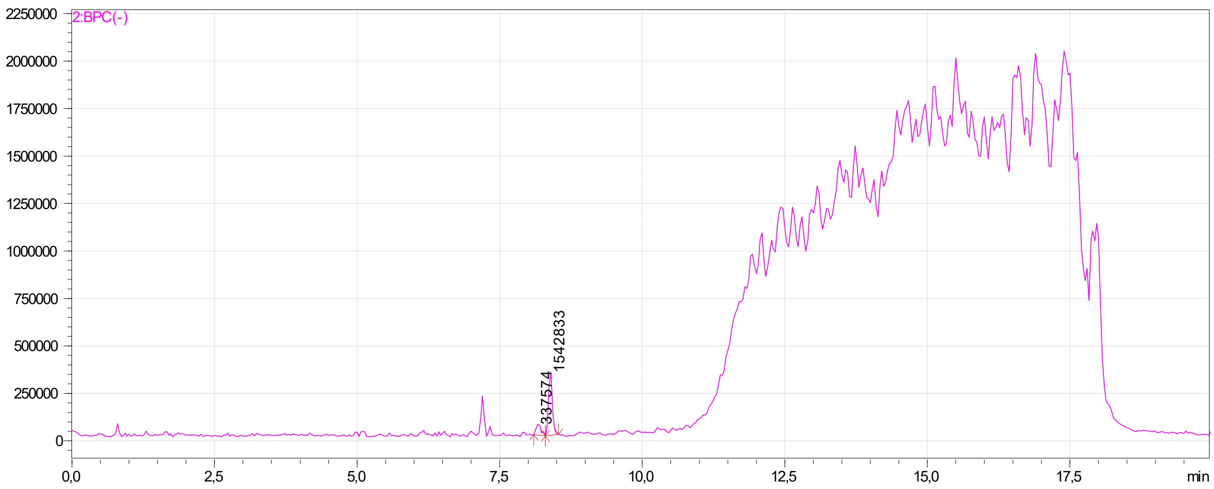

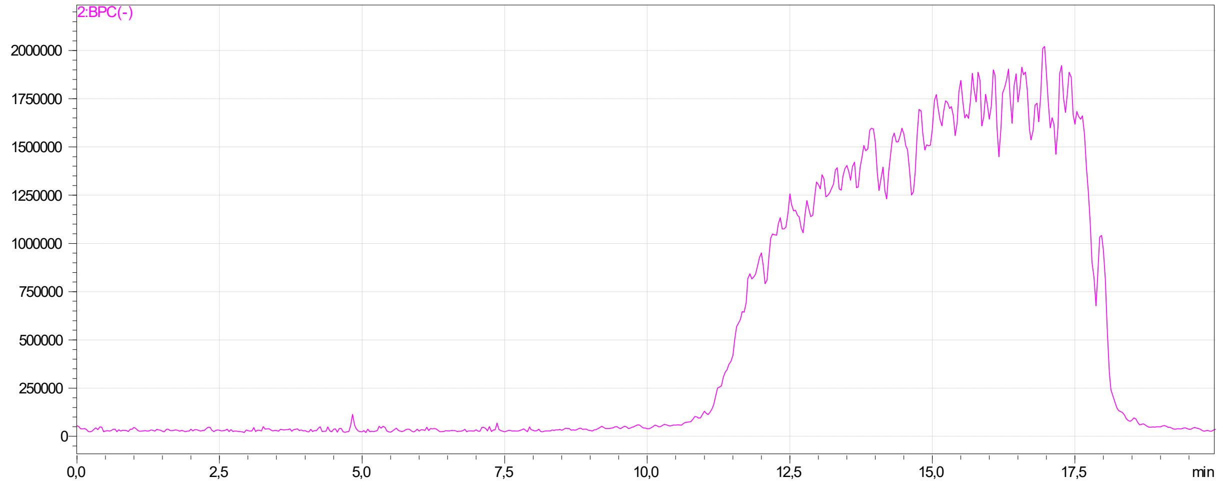

LCMS spectra for compound **13d**

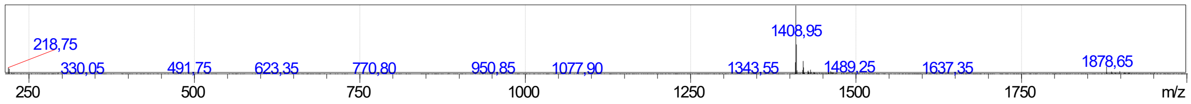

^{1}H NMR spectra for compound **Enz-COOH**

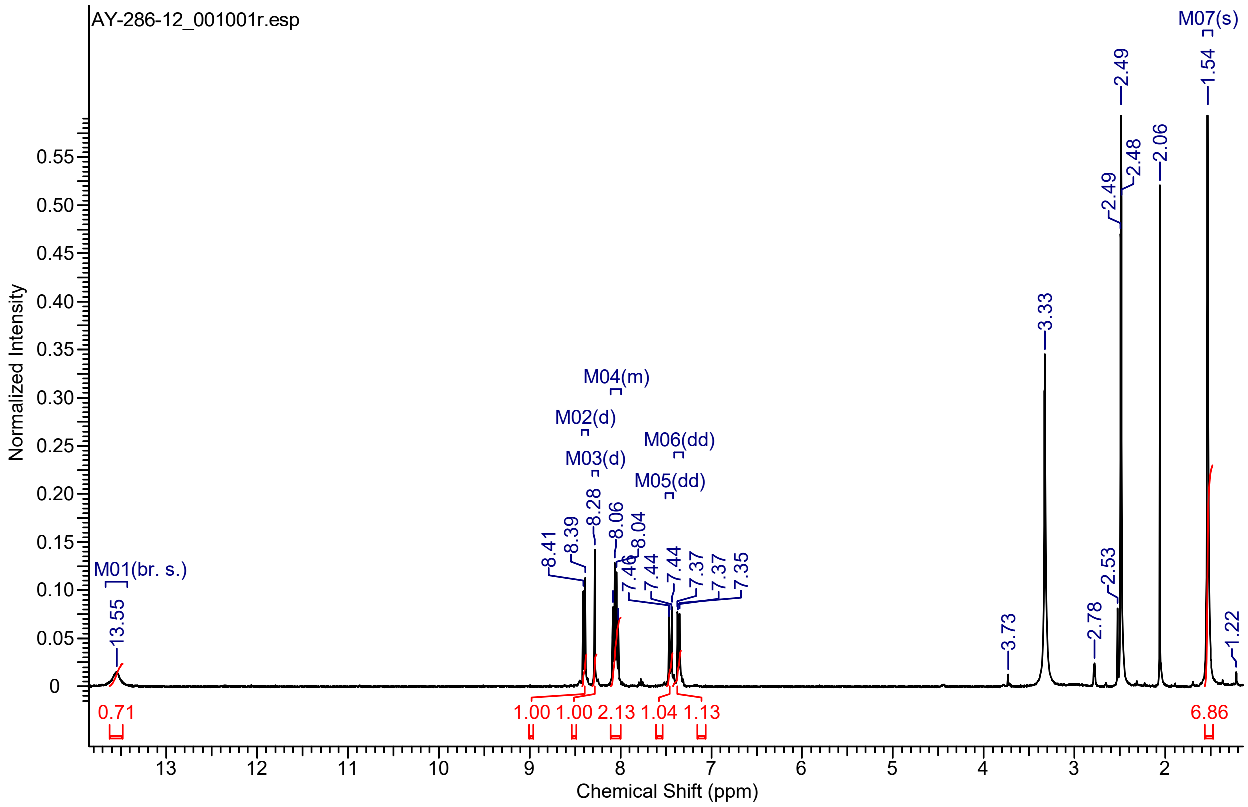

^{1}H NMR spectra for compound **14**

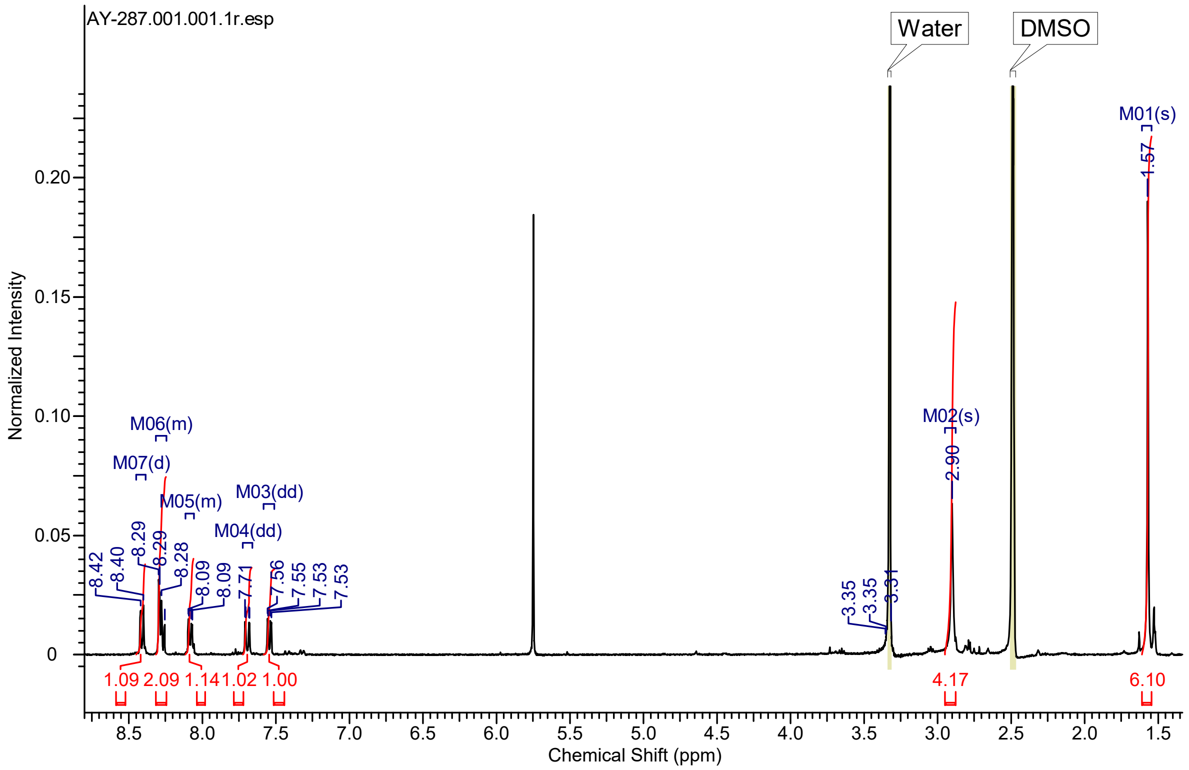

^{1}H NMR spectra for compound **15**

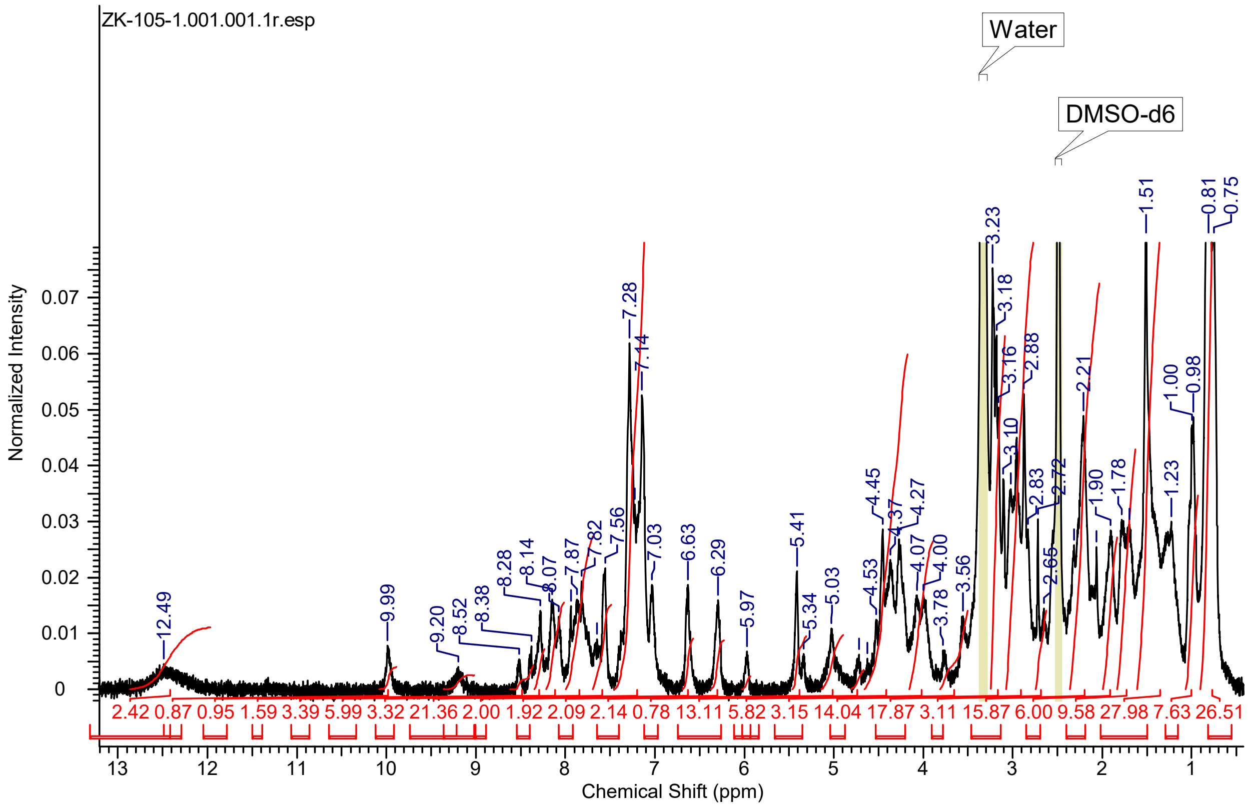

ESI-HRMS for compound **15**

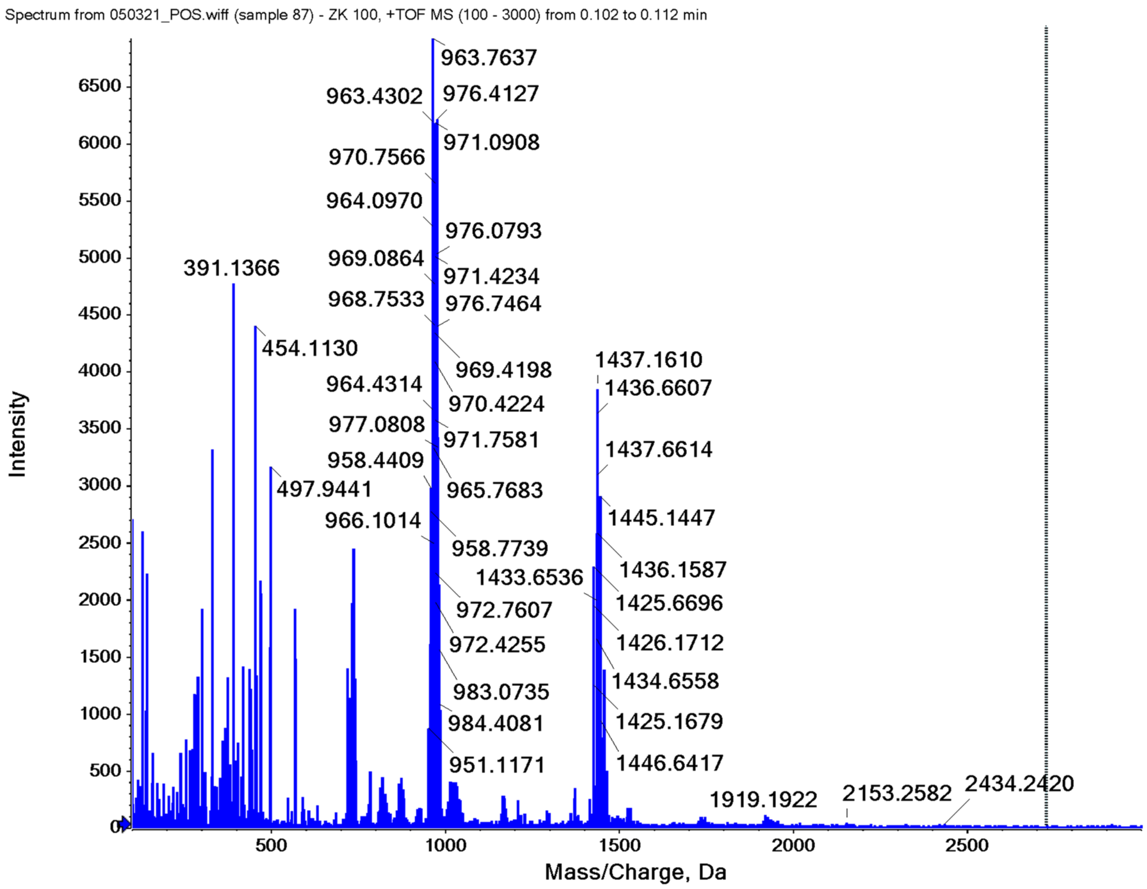

HPLC chromatogram for compound **15**
Positive ions (chromatogram and blank)

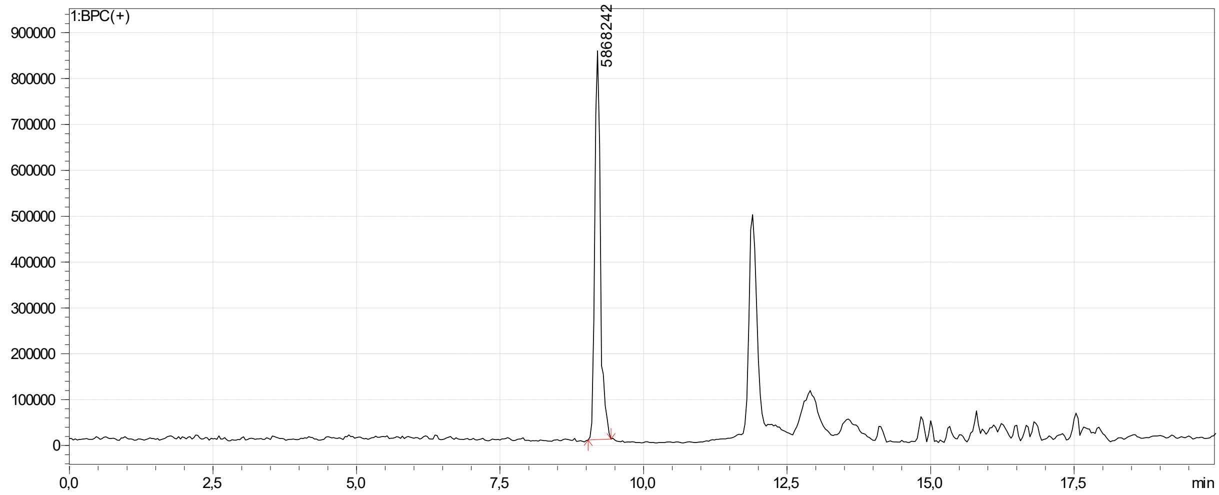

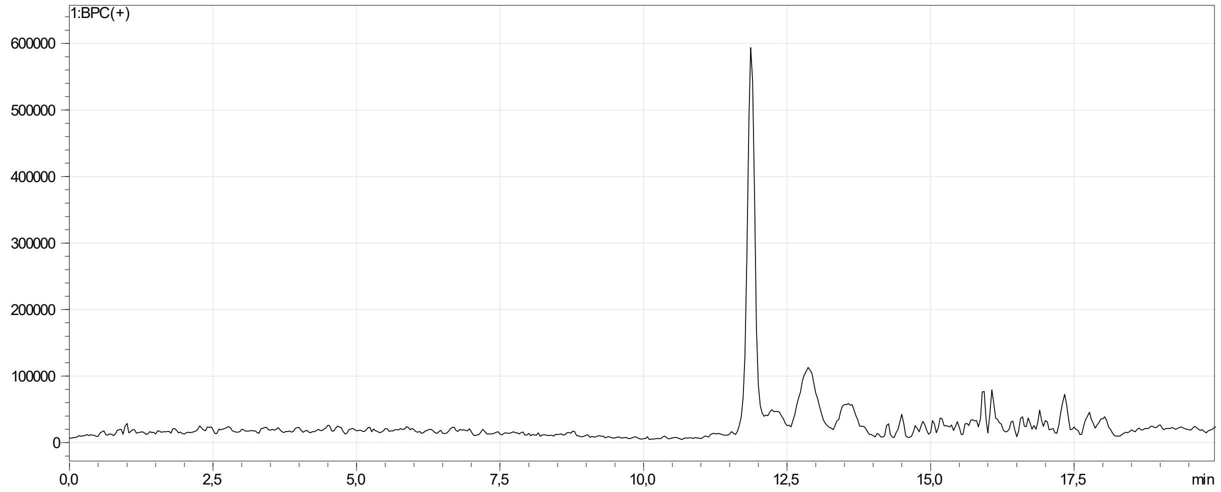

Negative ions (chromatogram and blank)

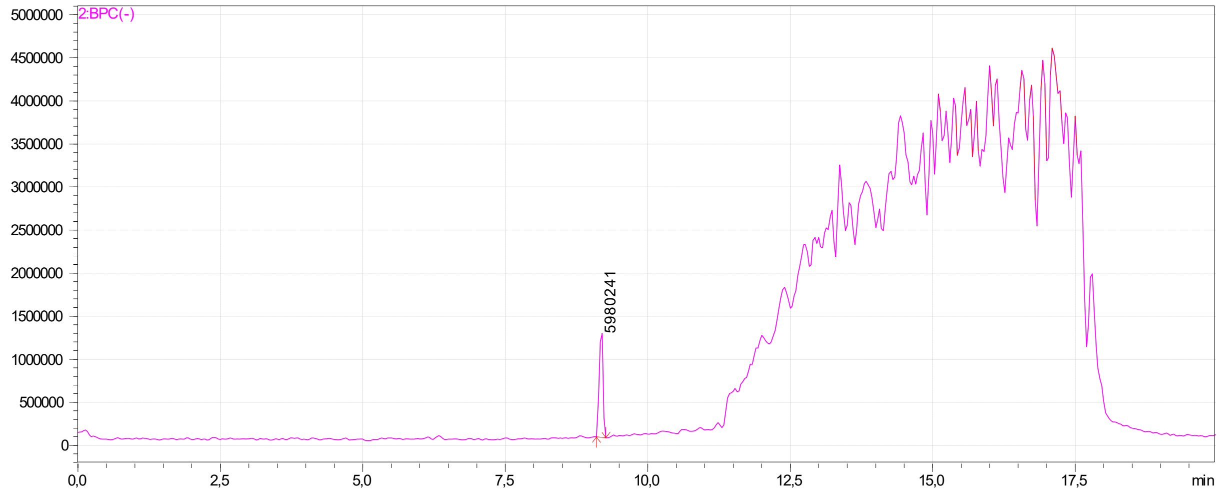

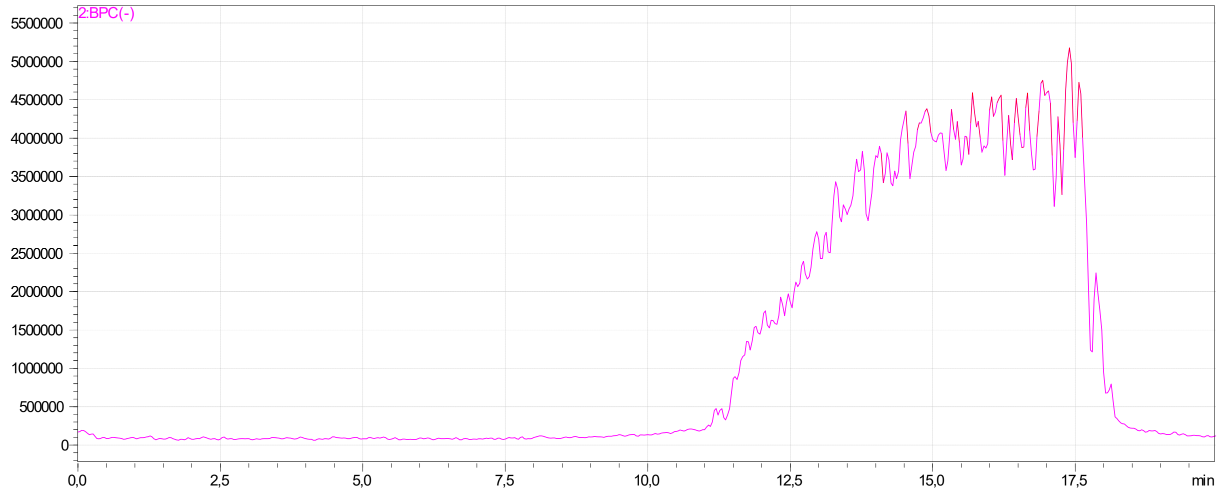

LCMS spectra for compound **15**

Positive ions spectra for compound **15**

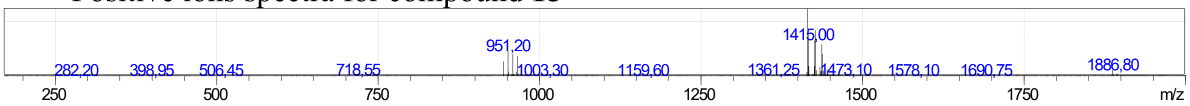

Negative ions spectra for compound **15**

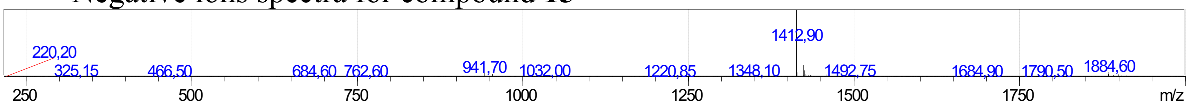

5. References

[1] Tietze, L. F.; Eicher, T.; Diederichsen, U.; Speicher, A.; Schützenmeister, N. *Reactions and Syntheses: In the Organic Chemistry Laboratory, 2nd, Completely Revised and Updated Edition*; Wiley-Vch, 2015.

[2] Zyk, N. Y.; Ber, A. P.; Nimenko, E. A.; Shafikov, R. R.; Evteev, S. A.; Petrov, S. A.; Uspenskaya, A. A.; Dashkova, N. S.; Ivanenkov, Y. A.; Skvortsov, D. A.; et al. Synthesis and Initial in Vitro Evaluation of PSMA-Targeting Ligands with a Modified Aromatic Moiety at the Lysine ε-Nitrogen Atom. *Bioorg. Med. Chem. Lett.*, **2022**, *71*, 128840. https://doi.org/10.1016/j.bmcl.2022.128840.

[3] Machulkin, A. E.; Shafikov, R. R.; Uspenskaya, A. A.; Petrov, S. A.; Ber, A. P.; Skvortsov, D. A.; Nimenko, E. A.; Zyk, N. U.; Smirnova, G. B.; Pokrovsky, V. S.; et al. Synthesis and Biological Evaluation of PSMA Ligands with Aromatic Residues and Fluorescent Conjugates Based on Them. *J. Med. Chem.*, **2021**, *64* (8), 4532–4552. https://doi.org/10.1021/acs.jmedchem.0c01935.

[4] Machulkin, A. E.; Uspenskaya, A. A.; Zyk, N. Y.; Nimenko, E. A.; Ber, A. P.; Petrov, S. A.; Shafikov, R. R.; Skvortsov, D. A.; Smirnova, G. B.; Borisova, Y. A.; et al. PSMA-Targeted Small-Molecule Docetaxel Conjugate: Synthesis and Preclinical Evaluation. *Eur. J. Med. Chem.*, **2022**, *227*, 113936. https://doi.org/10.1016/j.ejmech.2021.113936.

[5] Machulkin, A. E.; Uspenskaya, A. A.; Zyk, N. U.; Nimenko, E. A.; Ber, A. P.; Petrov, S. A.; Polshakov, V. I.; Shafikov, R. R.; Skvortsov, D. A.; Plotnikova, E. A.; et al. Synthesis, Characterization, and Preclinical Evaluation of a Small-Molecule Prostate-Specific Membrane Antigen-Targeted Monomethyl Auristatin E Conjugate. *J. Med. Chem.*, **2021**, *64* (23), 17123–17145. https://doi.org/10.1021/acs.jmedchem.1c01157.
